# Probing Unexpected Reactivity in Radiometal Chemistry: Indium-111-Mediated Hydrolysis of Hybrid Cyclen-Hydroxypyridinone Ligands

**DOI:** 10.1021/acs.inorgchem.3c00353

**Published:** 2023-03-17

**Authors:** Charlotte Rivas, Jessica A. Jackson, Alex Rigby, James A. Jarvis, Andrew J. P. White, Philip J. Blower, Andreas Phanopoulos, Michelle T. Ma

**Affiliations:** † School of Biomedical Engineering and Imaging Sciences, King’s College London, 4th Floor Lambeth Wing, St Thomas’ Hospital, London SE1 7EH, U.K.; ‡ Randall Centre of Cell and Molecular Biophysics and Centre for Biomolecular Spectroscopy, King’s College London, London SE1 9RT, U.K.; § Department of Chemistry, Molecular Sciences Research Hub, Imperial College London, London W12 0BZ, U.K.

## Abstract

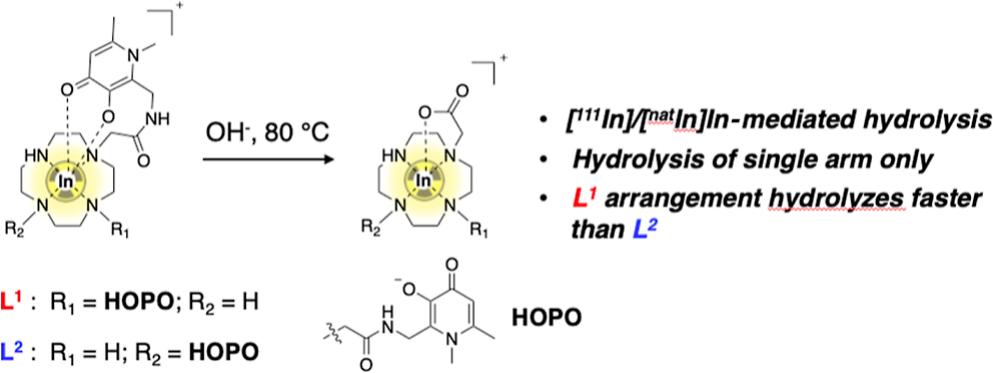

Chelators based on hydroxypyridinones have utility in incorporating radioactive metal ions into diagnostic and therapeutic agents used in nuclear medicine. Over the course of our hydroxypyridinone studies, we have prepared two novel chelators, consisting of a cyclen (1,4,7,10-tetraazacyclododecane) ring bearing two pendant hydroxypyridinone groups, appended via methylene acetamide motifs at either the 1,4-positions (**L**^**1**^) or 1,7-positions (**L**^**2**^) of the cyclen ring. In radiolabeling reactions of **L**^**1**^ or **L**^**2**^ with the γ-emitting radioisotope, [^111^In]In^3+^, we have observed radiometal-mediated hydrolysis of a single amide group of either **L**^**1**^ or **L**^**2**^. The reaction of either [^111^In]In^3+^ or [^nat^In]In^3+^ with either **L**^**1**^ or **L**^**2**^, in aqueous alkaline solutions at 80 °C, initially results in formation of [In(**L**^**1**^)]^+^ or [In(**L**^**2**^)]^+^, respectively. Over time, each of these species undergoes In^3+^-mediated hydrolysis of a single amide group to yield species in which In^3+^ remains coordinated to the resultant chelator, which consists of a cyclen ring bearing a single hydroxypyridinone group and a single carboxylate group. The reactivity toward hydrolysis is higher for the **L**^**1**^ complex compared to that for the **L**^**2**^ complex. Density functional theory calculations corroborate these experimental findings and importantly indicate that the activation energy required for the hydrolysis of **L**^**1**^ is significantly lower than that required for **L**^**2**^. This is the first reported example of a chelator undergoing radiometal-mediated hydrolysis to form a radiometalated complex. It is possible that metal-mediated amide bond cleavage is a source of instability in other radiotracers, particularly those in which radiometal complexation occurs in aqueous, basic solutions at high temperatures. This study highlights the importance of appropriate characterization of radiolabeled products.

## Abstract 2

We have prepared regioisomers of two novel bis(hydroxypyridinone)-cyclen ligands, **L**^**1**^ and **L**^**2**^. Complexation with either [^111^In]In^3+^ or [^nat^In]In^3+^ led to unexpected amide bond hydrolysis of **L**^**1**^ and **L**^**2**^, with a marked difference in reactivity between these two regioisomers toward hydrolysis. Hydrolysis occurs under reaction conditions that are commonly used in radiometal labeling reactions, highlighting the importance of thorough characterization of radiolabeled products.

## Introduction

Radiometal ions are used in nuclear medicine for both diagnostic imaging and systemic, targeted radiotherapy. In receptor-targeted diagnostic imaging and radiotherapy, a radioactive metal is bound via a chelator attached to a peptide or protein, which targets cell-surface receptors of diseased cells.^1^ New multidentate chelators that complex radiometal ions to provide thermodynamically and kinetically stable complexes have been seminal in the development of this field.

**Chart 1:**
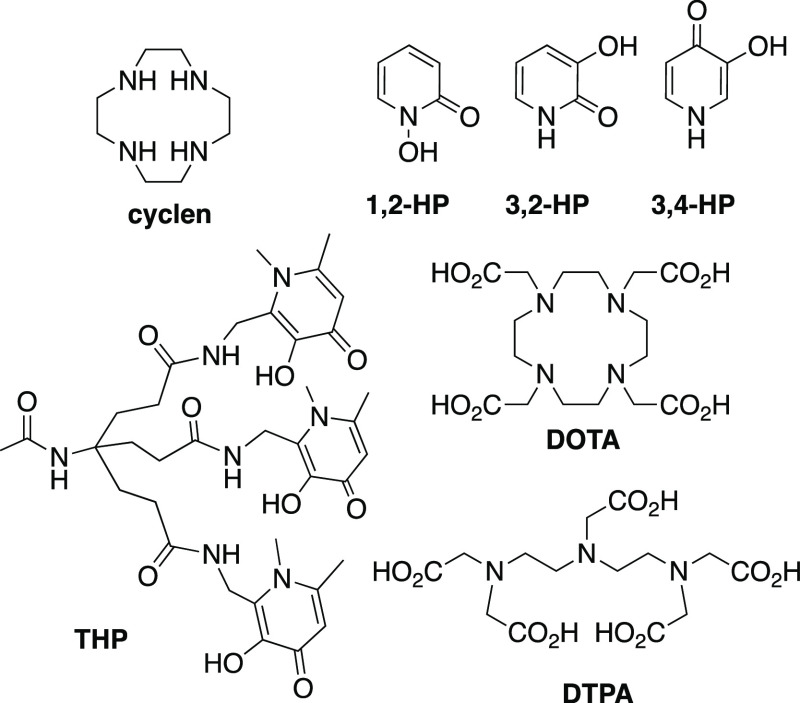


In recent years, macrocyclic derivatives of cyclen (1,4,7,10-tetraazacyclododecane, Chart 1) and crown ethers, all bearing additional pendant coordinating groups, have demonstrated utility for complexing hard metal ions with expanded coordination spheres.^2^ In addition to providing complexes of high thermodynamic stability, the inclusion of the macrocyclic ring imparts high kinetic stability. This kinetic stability is important for in vivo stability of a complex: radiopharmaceuticals are typically administered intravenously, resulting in very high dilutions in the bloodstream, and many endogenous ligands (proteins, peptides, and minerals) have high affinities for metal ions. Without possessing sufficient kinetic stability, most thermodynamically stable complexes will likely undergo some dissociation in vivo.

Hydroxypyridinones have a high affinity for metal ions with a high charge density. Varying the positions of the metal-binding hydroxyl and ketone groups of hydroxypyridinones results in three types of hydroxypyridinones: 1-hydroxy-2-pyridinones (**1,2-HP**), 3-hydroxy-2-pyridinones (**3,2-HP**), and 3-hydroxy-4-pyridinones (**3,4-HP**) (Chart 1).^3^ All three classes of hydroxypyridinones have the ability to sequester metal ions in vivo, including Fe^3+^, in hemochromatosis or iron-overload disease,^3^ and actinide ions, in the event of exposure or contamination in the nuclear industry.^4^ Chelators based on hydroxypyridinones have also been used for complex radiometals used in nuclear medicine, including [^68^Ga]Ga^3+^ and [^89^Zr]Zr^4+^ for positron emission tomography imaging,^5−9^ radioisotopes of lanthanides and actinides for radionuclide imaging and therapy,^10−12^ and in luminescent complexes.^13^ Our prior research on a tris(1,6-dimethyl-3-hydroxy-4-pyridinone) chelator (**THP**, Chart 1) has shown that it is an ideal chelator for rapid, kit-based preparation of [^68^Ga]Ga-labeled peptides,^14,15^ and a [^68^Ga]Ga–THP-peptide targeting prostate cancer has entered clinical trials.^15,16^

Indium-111 (^111^In) is a γ-emitting radioisotope (γ 173 keV, 90.5% and γ 247 keV, 94%, half-life = 67 h) used for γ-scintigraphy and single-photon emission computerized tomography imaging. Several of the first Food and Drug Administration-approved receptor-targeted radiopharmaceuticals incorporated ^111^In, enabling molecular imaging of various types of cancers;^1^ the [^111^In]In-labeled radiopharmaceutical, “octreoscan”, used for imaging neuroendocrine tumors, is still in routine use.^17^ The chelators diethylenetriaminepentaacetic acid (**DTPA**) and 1,4,7,10-tetraazacyclododecane–1,4,7,10-tetraacetic acid (**DOTA**) (Chart 1) are commonly used for incorporating [^111^In]In^3+^ into biomolecules. Derivatives of bidentate 3,4-HP have shown high affinity for In^3+^, with β_3_ stability constants for [In(**3,4-HP**)_3_] complexes of the order of 10^31^–10^33^.^18,19^ Bidentate hydroxypyridinone derivatives have been applied to the complexation of [^111^In]In^3+^ for cell labeling^20^ and incorporating [^111^In]In^3+^ into targeting biomolecules such as carbohydrates.^21^

We postulated that combining two hydroxypyridinone groups with cyclen could lead to an octadentate chelator that (i) possesses a high affinity for hard metal ions with expanded coordination spheres and (ii) provides complexes of high kinetic stability, typical of cyclen derivatives. As part of our investigations, we prepared two cyclen regioisomers, **L**^**1**^ and **L**^**2**^, each bearing two 1,6-dimethyl-3-hydroxy-4-pyridinone groups, in either the N^1^,N^4^-positions (**L**^**1**^) or N^1^,N^7^-positions (**L**^**2**^). There are potential differences in the reactivity of cyclen-based regioisomers. For example, regioisomers of “DO2A”, tetraazacyclododecane-1,4-diacetic acid and tetraazacyclododecane-1,7-diacetic acid, both coordinate with Mn^2+^.^22^ The resulting two Mn^2+^ complexes exhibit similar structural properties; however, the N^1^,N^4^-derivative exhibits superior thermodynamic stability and kinetic inertness. In the first instance, we elected to react the new **L**^**1**^ and **L**^**2**^ chelators with [^111^In]In^3+^.

In the course of these studies, we observed unexpected behavior for both **L**^**1**^ and **L**^**2**^ when reacted with aqueous solutions containing In^3+^: in the presence of either salt of [^nat^In]In^3+^ or radioactive [^111^In]In^3+^, both chelators undergo hydrolysis of a single amide bond, yielding In^3+^ complexes in which a single hydroxypyridinone group and a single carboxylate group are tethered to a cyclen ring. We detail our observations here. To the best of our knowledge, this is the first report of radiometal-mediated hydrolysis in the radiopharmaceutical literature.

## Results

### Synthesis

**Scheme 1:**
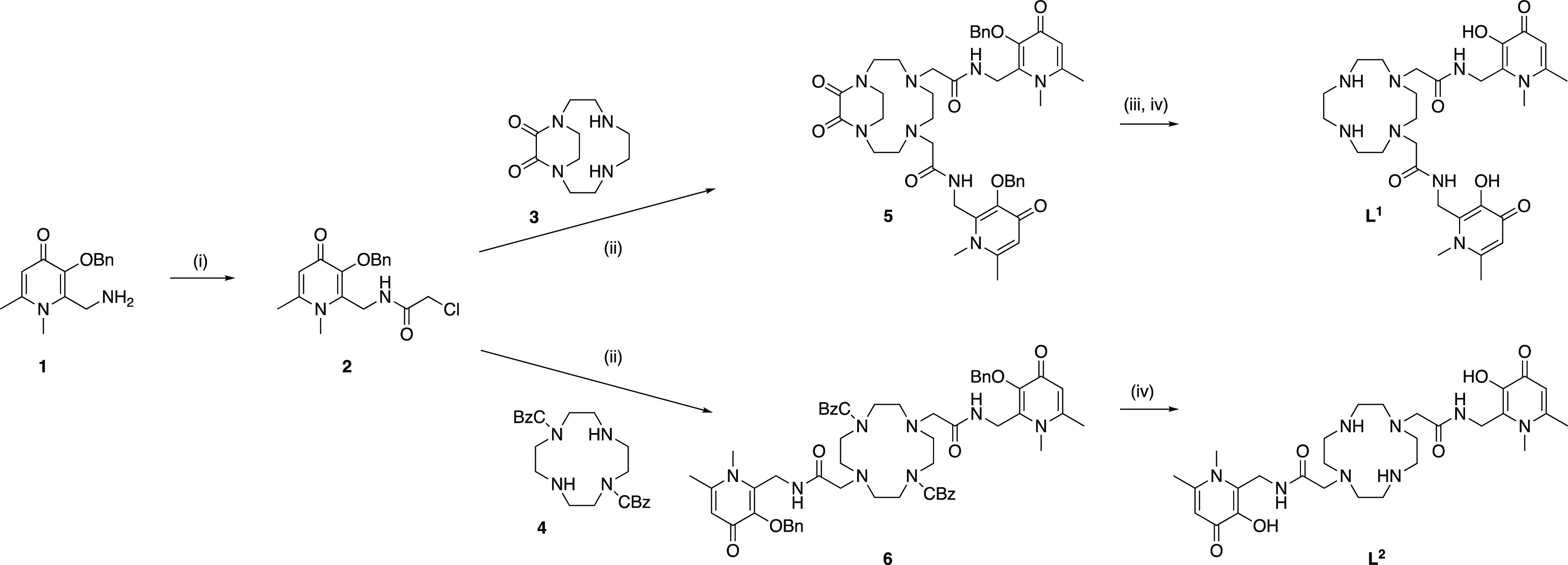
Synthesis of **L**^**1**^ and **L**^**2**^ (i) Chloroacetyl Chloride, K_2_CO_3_, DCM, H_2_O, 0 °C to rt, 16 h; (ii) DIPEA, CH_3_CN, MeOH, 60 °C, 3 days; (iii) NaOH, H_2_O, 90 °C, 16 h; (iv) H_2_, Pd/C, MeOH, rt, 16 h

We elected to append two **3,4-HP** derivatives to protected derivatives of cyclen to obtain target ligands **L**^**1**^ and **L**^**2**^ (Scheme 1). Compound **1**,^23^ cyclenoxamide **3** (N^1^,N^4^-diprotected cyclen),^24^ and diCBz-cyclen **4** (N^1^,N^7^-diprotected cyclen)^25^ were prepared following previously reported syntheses. Compounds **5** and **6** were obtained by alkylation of **3** and **4**, respectively, with the **3,4-HP** chloromethyl derivative (**2**) in acetonitrile in the presence of diisopropylethylamine. **L**^**1**^ was obtained following two deprotection steps, first by hydrolysis under basic conditions to give the N^1^,N^4^-dialkylated cyclen followed by removal of the benzyl groups on the **3,4-HP** moieties by catalytic hydrogenolysis. **L**^**2**^ was conveniently fully deprotected in one step, removing both benzyl and carboxybenzyl protecting groups using catalytic hydrogenolysis only.

### Reaction of **L**^**1**^ with [^111^In]In^3+^ and [^nat^In]In^3+^

**Figure 1:**
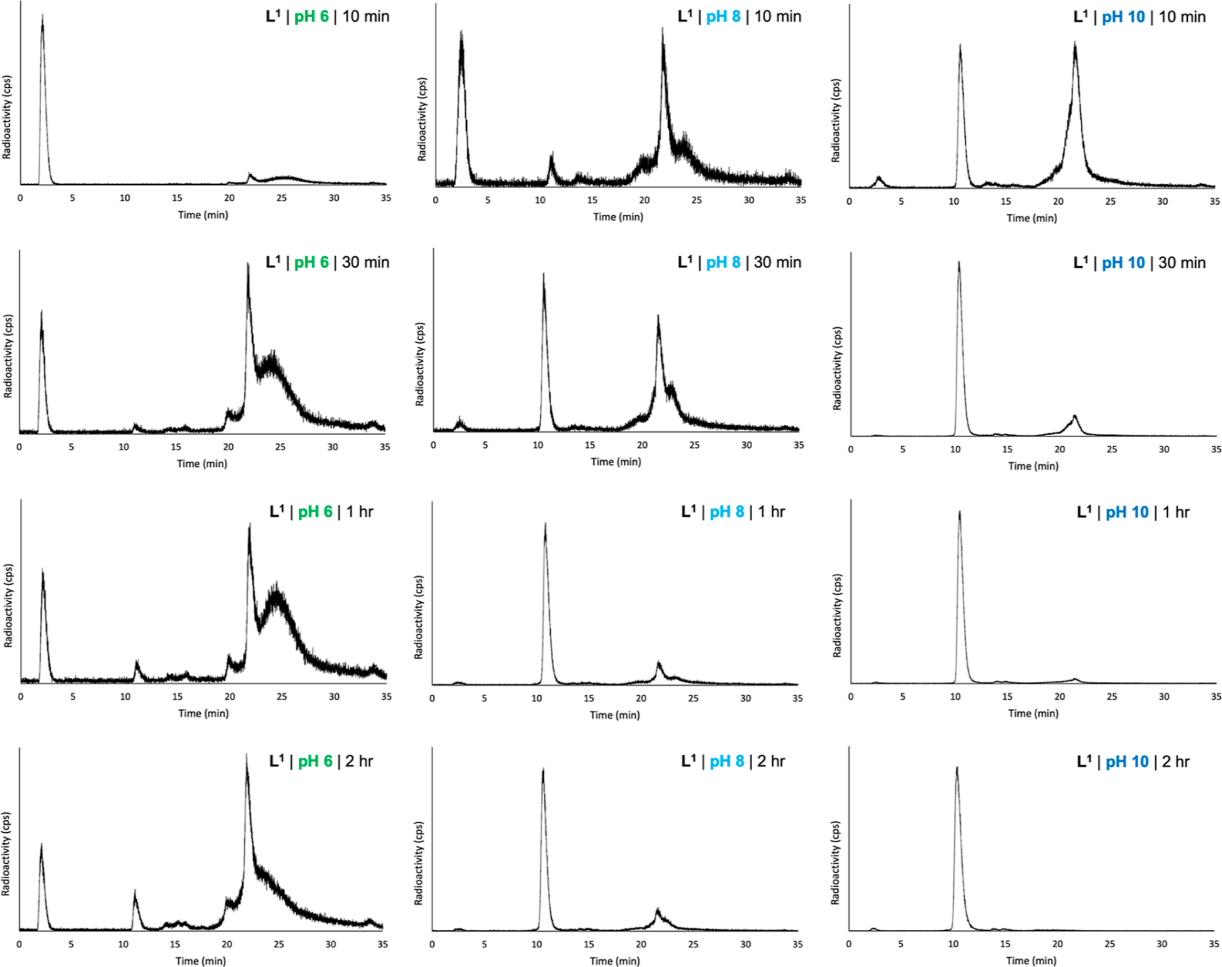
Reverse-phase radio-HPLC chromatograms of reaction mixtures of **L**^**1**^ with [^111^In]In^3+^, undertaken at 80 °C, at either pH 6, 8, or 10, measured at either 10 min, 30 min, 1 h, or 2 h.

To assess the ability of **L**^**1**^ to complex In^3+^, **L**^**1**^ was initially reacted with solutions of [^111^In]In^3+^ at 80 °C and varying pH (pH = 6, 8, or 10). These reactions were monitored by reversed-phase analytical radio-high-performance liquid chromatography (HPLC) over 2 h (Figure 1).

When [^111^In]In^3+^ was reacted with **L**^**1**^ at pH 6 and 80 °C, multiple species were observed, and **L**^**1**^ did not quantitatively bind to all available [^111^In]In^3+^, although the amount of unreacted [^111^In]In^3+^ (retention time of 2.5 min) decreased over time. Notably, the radiochemical yield of the [^111^In]In-bound species with a retention time of 21.5 min ([^111^In]In-**L**^**1A**^) increased over time (Figure 1). A broad signal at 23–28 min was also observed in all radiochromatograms. From 30 min onward, a species with a retention time of 10.5 min ([^111^In]In-**L**^**1B**^) was observed. When the reaction was repeated at pH 8 and 80 °C, [^111^In]In-**L**^**1B**^ increased in yield over time (Figure 1, 31% at 30 min and 72% at 120 min). [^111^In]In-**L**^**1A**^ was also observed at early reaction time points (29% radiochemical yield at 30 min) but decreased over time (8% at 120 min) as the amount of [^111^In]In-**L**^**1B**^ increased. At pH 10 and 80 °C, 80% of the radioactivity was associated with [^111^In]In-**L**^**1B**^ after only 30 min of reaction. By 120 min, the radiochemical yield of [^111^In]In-**L**^**1B**^ measured 98%. Importantly, only low amounts of [^111^In]In-**L**^**1A**^ (11% at 30 min and 0.3% at 120 min) and unreacted [^111^In]In^3+^ (<1% at 30 min onward) were observed. The broad species observed at pH 6 (and to a lesser extent at pH 8), eluting at 23–28 min, was not observed for reactions at pH 10.

These radiolabeling data point to a reaction pathway in which **L**^**1**^ initially reacts with [^111^In]In^3+^ to give an intermediate species, [^111^In]In-**L**^**1A**^, which then reacts further under sufficiently basic conditions to yield [^111^In]In-**L**^**1B**^.

**Figure 2:**
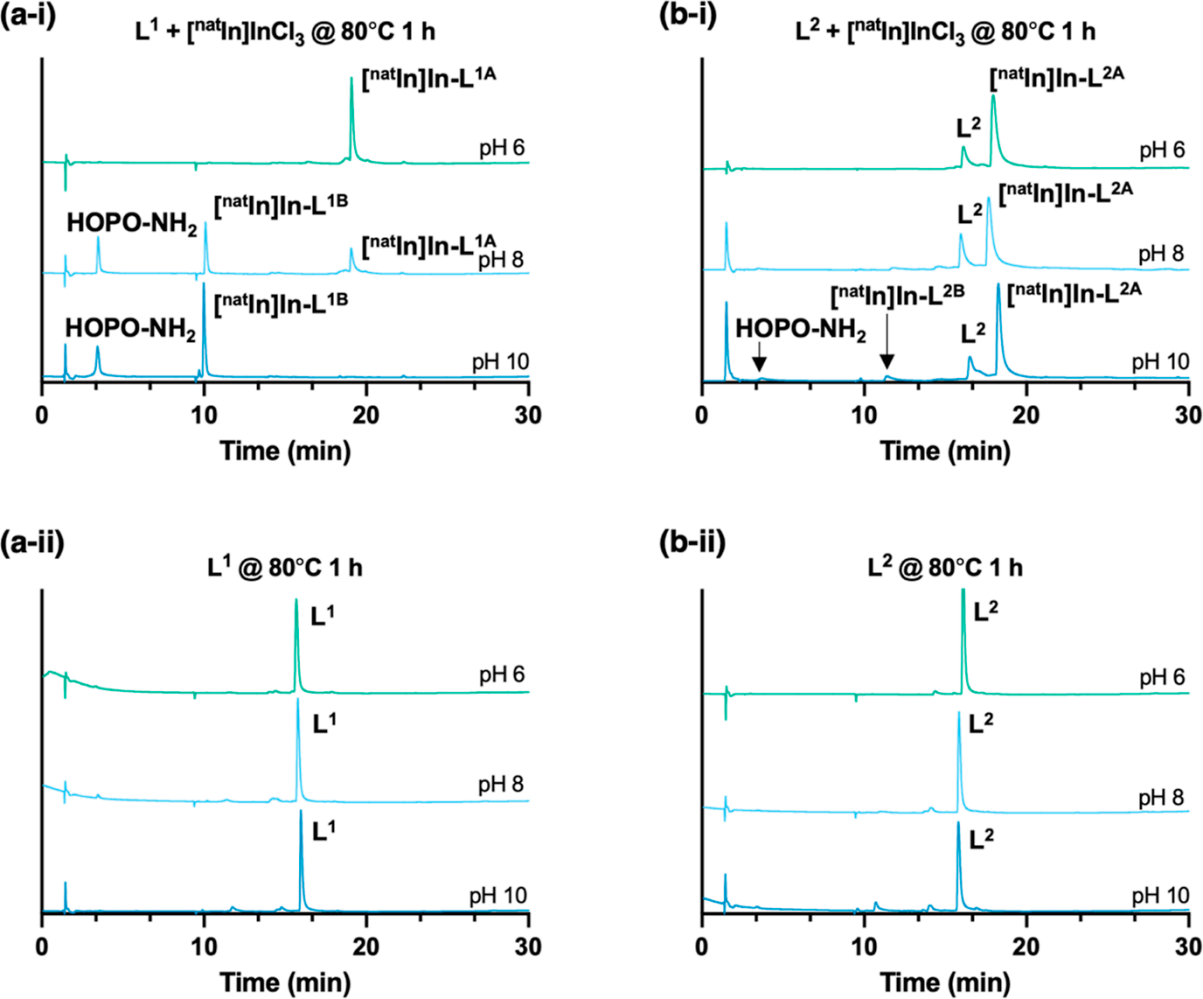
Reverse-phase LC–MS chromatograms (λ = 254 nm) of reaction mixtures of (a–i) **L**^**1**^ or (b–i) **L**^**2**^ with [^nat^In]InCl_3_, undertaken at either pH 6, 8, or 10, after 1 h reaction at 80 °C. Solutions of (a-ii) **L**^**1**^ or (b-ii) **L**^**2**^ at either pH 6, 8, or 10 were also heated for 1 h at 80 °C (without [^nat^In]InCl_3_), prior to LC–MS analysis.

**Scheme 2:**
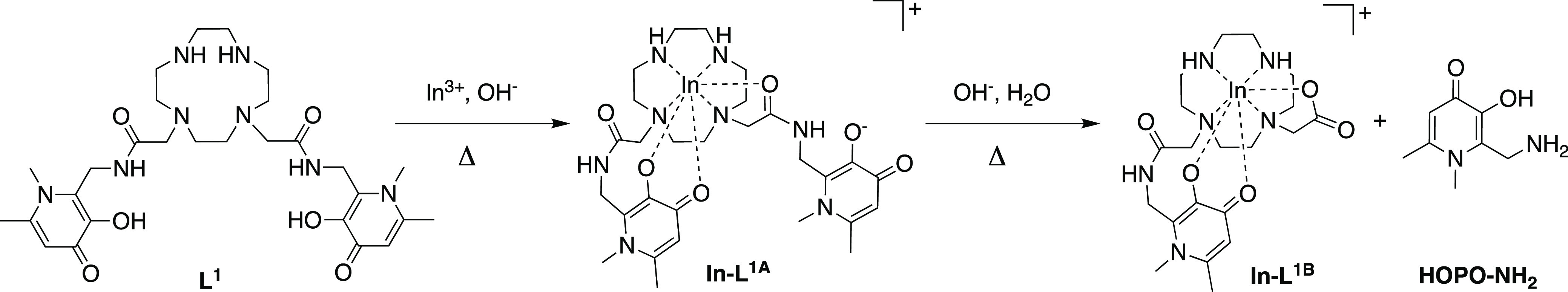
Reaction of **L**^**1**^ with In^3+^, Including a Putative Structure of Intermediate In-**L**^**1A**^

Concurrent with radiolabeling experiments, we prepared [^nat^In]In-**L**^**1A**^ and [^nat^In]In-**L**^**1B**^: an aqueous solution of **L**^**1**^ was reacted at 80 °C with [^nat^In]InCl_3_ (1 equiv) for 1 h, at either pH 6, pH 8, or pH 10, followed by liquid chromatography (LC)–low-resolution mass spectrometry (LRMS) analysis (Figure 2). At pH 6, only a single major species, assigned as [^nat^In]In-**L**^**1A**^ (retention time = 19.1 min), was observed, with an *m*/*z* signal consistent with the stoichiometry of a complex in which a single equivalent of **L**^**1**^ coordinates to [^nat^In]In^3+^ ([M]^+^*m*/*z* = 701.1 obs, 701.2 calc). At pH 8, two additional species, assigned as [^nat^In]In-**L**^**1B**^ (retention time = 10.1 min) and **HOPO–NH**_**2**_ (retention time = 3.5 min, structure in Scheme 2), were observed. The *m*/*z* signal of [^nat^In]In-**L**^**1B**^ is consistent with that of an [^nat^In]In^3+^-bound complex of a cyclen motif appended to a single hydroxypyridinone group and a single carboxylate/carboxylic acid group ([M]^+^*m*/*z* = 550.9 obs, 551.2 calc). Lastly, at pH 10, only [^nat^In]In-**L**^**1B**^ and **HOPO–NH**_**2**_ were observed in the chromatogram.

In combination, these [^111^In]In^3+^ and [^nat^In]In^3+^ complexation studies suggest that **L**^**1**^ reacts with In^3+^ to first furnish species In-**L**^**1A**^ in which **L**^**1**^ is bound to In^3+^ (Scheme 2). Under sufficiently basic conditions, this species undergoes hydrolysis of a single amide bond to yield complex In-**L**^**1B**^, in which In^3+^ is coordinated to “**L**^**1B**^”, likely through cyclen amine groups, hydroxypyridinone groups, and a carboxylate (see DFT Calculations, vide infra). Significantly, this hydrolysis reaction is mediated by In^3+^. When **L**^**1**^ was heated at 80 °C for 1 h at either pH 6, pH 8, or pH 10 in the absence of In^3+^ and analyzed by analytical LC–MS, no changes in the UV chromatogram, relative to that of freshly dissolved **L**^**1**^, were observed: **L**^**1**^ remained intact.

**Figure 3:**
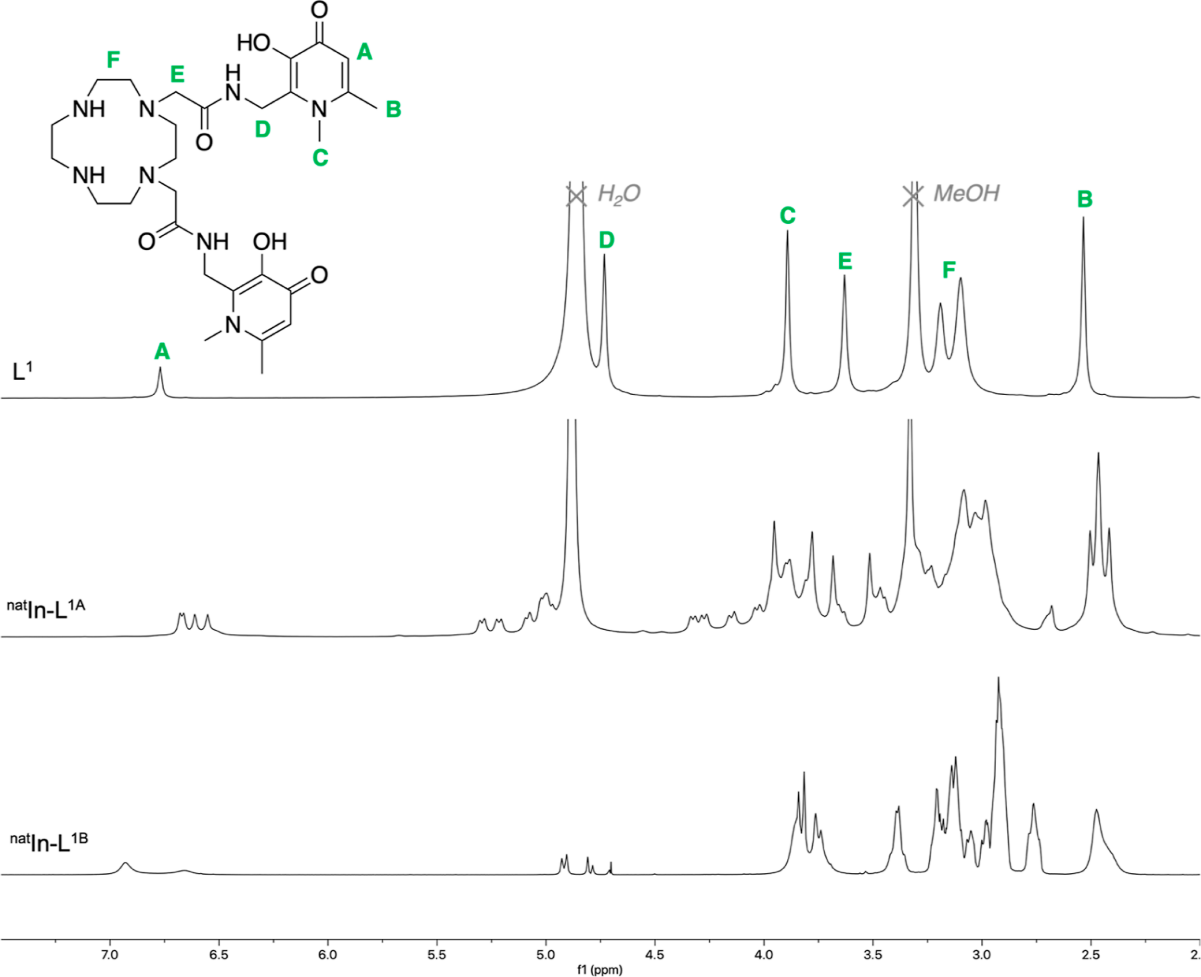
^1^H NMR spectra of **L**^**1**^ (top, CD_3_OD, 700 MHz), [^nat^In]In-**L**^**1A**^ (middle, CD_3_OD, 700 MHz), and [^nat^In]In-**L**^**1B**^ (bottom, D_2_O with water suppression, 700 MHz).

The compounds [^nat^In]In-**L**^**1A**^, [^nat^In]In-**L**^**1B**^, and **HOPO–NH**_**2**_ were subsequently prepared and isolated (with HR–ESI–MS data consistent with LC–LRMS data, see the Supporting Information). Compared to the nuclear magnetic resonance (NMR) spectrum of **L**^**1**^, the NMR spectra of [^nat^In]In-**L**^**1A**^ (acquired in methanol-*d*_4_) exhibited several notable features (Figures 3 and S3).

(i) Four signals for each of the hydroxypyridinone N^1^–CH_3_, C^5^–H, and C^6^–CH_3_ proton environments were observed.
(ii) Signals corresponding to the cyclen CH_2_ ethylene protons of [^nat^In]In-**L**^**1A**^ exhibited more complex splitting/coupling patterns compared to the corresponding signals in **L**^**1**^, which were broad and unresolved.
(iii) Signals corresponding to the CH_2_ methylene groups of [^nat^In]In-**L**^**1A**^ exhibited more complex splitting/coupling, compared to the sharp singlets of **L**^**1**^.
(iv) Integration of ^1^H signals of [^nat^In]In-**L**^**1A**^ was consistent with the **L**^**1**^ chelator remaining intact when coordinated to [^nat^In]In^3+^.
(v) ^13^C NMR (Figure S3) of [^nat^In]In-**L**^**1A**^ similarly revealed an increase in the number of ^13^C signals relative to the free ligand.

These ^1^H NMR data are consistent with **L**^**1**^ coordinating to [^nat^In]In^3+^ through at least one hydroxypyridinone group in [^nat^In]In-**L**^**1A**^ and cyclen amine groups or amide carbonyl groups. The increased complexity of cyclen CH_2_ signals is consistent with cyclen amine ligands’ participation in In^3+^ coordination. It is possible that [^nat^In]In-**L**^**1A**^ is highly fluxional and that multiple species exist in solution that interconvert via transient dissociation and recoordination of **L**^**1**^ donor atoms.

There were several significant features in the NMR spectra of [^nat^In]In-**L**^**1B**^ (acquired in D_2_O) that suggest that both the single hydroxypyridinone group and the cyclen ring participate in the coordination of [^nat^In]In^3+^ (Figures 3 and S4).

(i) Integration of the ^1^H NMR spectrum of [^nat^In]In-**L**^**1B**^ indicated that only a single hydroxypyridinone group is appended to the cyclen ring, and an additional, chemically distinct, methylene group is present.
(ii) ^1^H NMR signals corresponding to the ethylene groups of the cyclen ring in [^nat^In]In-**L**^**1B**^ were sharper and exhibited more complex splitting/coupling patterns compared to the corresponding signals in **L**^**1**^. These patterns are typical for cyclen derivatives in which ring amine groups are complexed to a diamagnetic metal ion.
(iii) ^1^H NMR signals corresponding to hydroxypyridinone substituents were broadened, and more complex splitting was observed, relative to those of **L**^**1**^.
(iv) The ^1^H–^13^C HSQC NMR spectrum of [^nat^In]In-**L**^**1B**^ (Figure S4) revealed an increase in the number of chemically inequivalent cyclen CH_2_ groups (eight distinct cyclen cross-peaks) relative to that of **L**^**1**^ (four chemically distinct cyclen CH_2_ signals). In the 1D ^13^C{H} spectrum, cyclen signals were discernible, but signals for hydroxypyridinone C atoms were not discernible or were very weak, presumably due to significant broadening.

### Reaction of **L**^**2**^ with [^111^In]In^3+^ and [^nat^In]In^3+^

**Figure 4:**
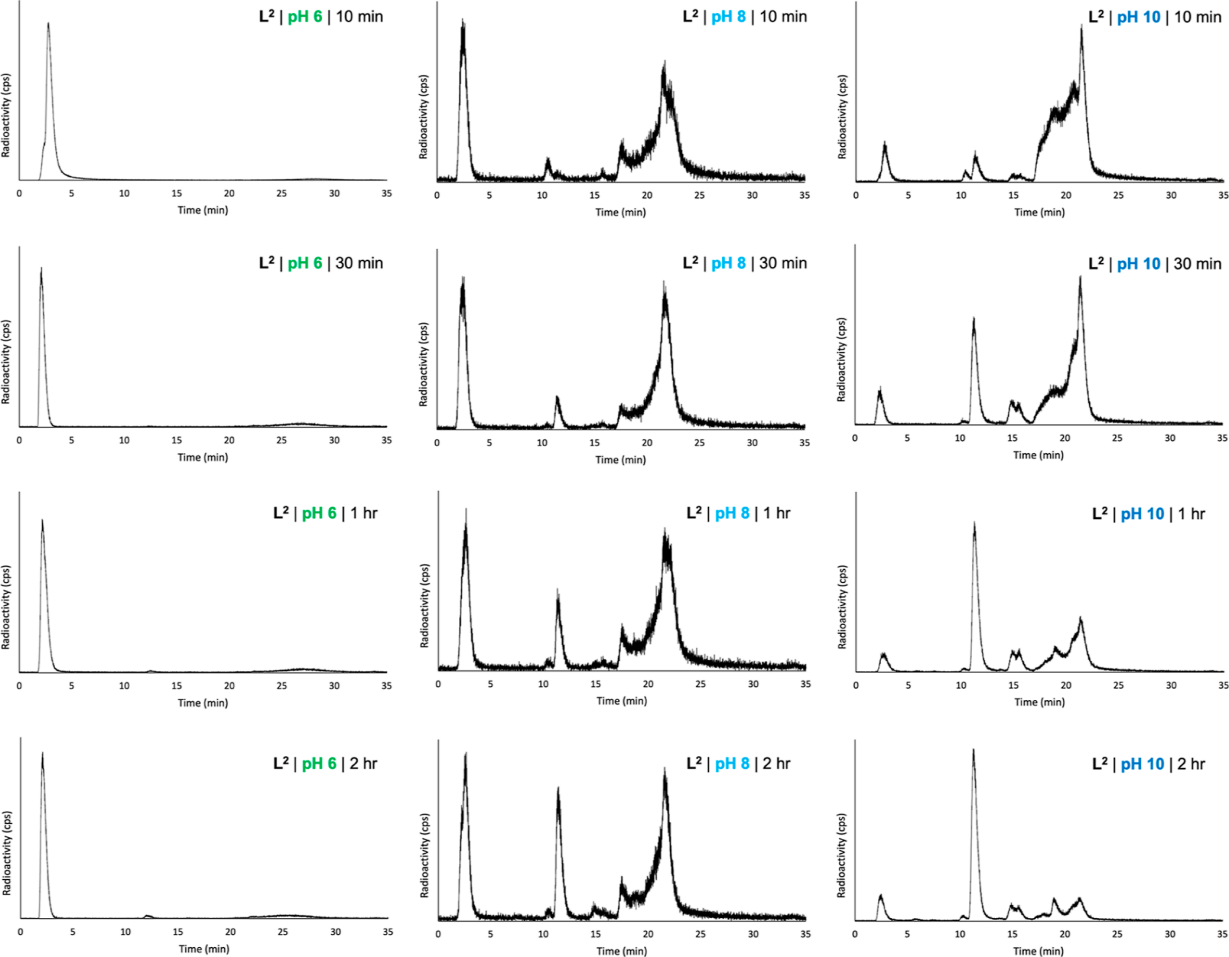
Reverse-phase radio-HPLC chromatograms of reaction mixtures of **L**^**2**^ with [^111^In]In^3+^, undertaken at 80 °C, at either pH 6, 8, or 10, measured at either 10 min, 30 min, 1 h, or 2 h.

We then studied the reaction of [^nat^In]In^3+^ with **L**^**2**^ (in which pendant hydroxypyridinone groups are attached at the N^1^ and N^7^ positions of the cyclen ring). Similar to our strategy for elucidating the reactivity of **L**^**1**^ with [^111^In]In^3+^, we reacted **L**^**2**^ with solutions of [^111^In]In^3+^ and [^nat^In]In^3+^ at pH 6, pH 8, and pH 10 at 80 °C and analyzed these reactions using reversed-phase analytical radio-HPLC (Figure 4).

At pH 6, even after 2 h of reaction, the majority of radioactivity signal corresponded to unreacted [^111^In]In^3+^. This contrasts the reactivity of **L**^**1**^ with [^111^In]In^3+^ at pH 6. However, at pH 8, **L**^**2**^ reacted with [^111^In]In^3+^ to yield a species with a retention time of 21.5 min ([^111^In]In-**L**^**2A**^) and a second species with a retention time of 11.3 min ([^111^In]In-**L**^**2B**^). Compound [^111^In]In-**L**^**2A**^ was present in a >25% radiochemical yield at all measured time points; the radiochemical yield of [^111^In]In-**L**^**2B**^ increased over the course of the reaction, from 1% at 10 min to 17% at 120 min. Other radioactive species, including unreacted [^111^In]In^3+^, were also present.

The reaction of **L**^**2**^ with [^111^In]In^3+^ at pH 10 and 80 °C yielded multiple species; however, after 2 h of reaction, the major radioactive product corresponded to [^111^In]In-**L**^**2B**^. Similar to the reaction at pH 8, signals corresponding to [^111^In]In-**L**^**2A**^ and other species were discernible over the course of the 2 h reaction but decreased as the radiochemical yield of [^111^In]In-**L**^**2B**^ increased from 4% at 10 min to 56% at 2 h.

**Scheme 3:**
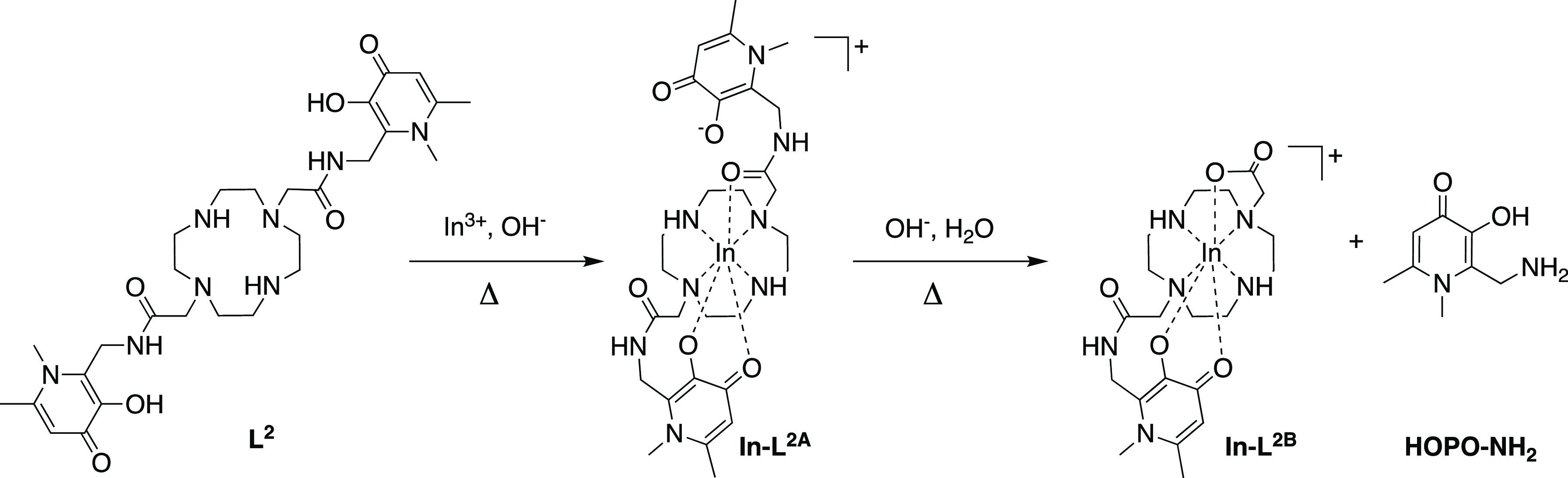
Reaction of **L**^**2**^ with In^3+^, Including a Putative Structure of Intermediate In-**L**^**2A**^

The reaction of **L**^**2**^ with [^nat^In]InCl_3_ (for 1 h at 80 °C), at either pH 6, pH 8, or pH 10, followed by LC–MS analysis, revealed a similar reaction pathway to that of **L**^**1**^ (Figure 2 and Scheme 3). **L**^**2**^ reacts with [^nat^In]In^3+^ to yield an intermediate complex, [^nat^In]In-**L**^**2A**^ (retention time = 18.3 min, [M]^+^*m*/*z* = 701.4 obs, 701.2 calc), which undergoes hydrolysis of a single amide bond to furnish [^nat^In]In-**L**^**2B**^ (retention time = 11.9 min, [M]^+^*m*/*z* = 551.2 obs, 551.2 calc). In the case of **L**^**1**^, [^nat^In]In^3+^ complexation and subsequent hydrolysis were complete within 1 h. However, in the case of **L**^**2**^, some unreacted ligand was observed (retention time = 16.6 min, [M + H]^+^*m*/*z* = 589.5 obs, 589.3 calc), and only small amounts of [^nat^In]In-**L**^**2B**^ and **HOPO–NH**_**2**_ were detected after 1 h of reaction at 80 °C at pH 10. At all pH values, the major species in the solution corresponded to [^nat^In]In-**L**^**2A**^.

The [^nat^In]In-**L**^**2A**^ and [^nat^In]In-**L**^**2B**^ complexes were isolated and analyzed by NMR (Figures S7 and S8) and HR-MS, which indicated that [^nat^In]In-**L**^**2A**^ and [^nat^In]In-**L**^**2B**^ were isomeric with [^nat^In]In-**L**^**1A**^ and [^nat^In]In-**L**^**1B**^, respectively.

In the ^1^H NMR of [^nat^In]In-**L**^**2A**^, signals of hydroxypyridinone and cyclen exhibited increased splitting compared to those of **L**^**2**^. Similar to [^nat^In]In-**L**^**1A**^, we postulate that this is consistent with **L**^**2**^ coordinating to In^3+^ through at least one hydroxypyridinone group in In-**L**^**2A**^ and possibly also through cyclen amine groups and/or amide carbonyl groups. ^1^H NMR and HSQC spectral features for [^nat^In]In-**L**^**2B**^ were very similar to those of [^nat^In]In-**L**^**1B**^. Importantly, in combination, MS and NMR data indicated that ligand “**L**^**2B**^” consists of a cyclen ring bearing a single hydroxypyridinone group and a single carboxylate/carboxylic acid group in the N^1^ and N^7^ positions.

### DFT Calculations

To further understand the possible coordination environments and reactivity of this series of In^3+^ complexes, DFT calculations were performed. Full details of the computational analysis can be found in the Supporting Information. Initially, the coordination of In^3+^ to **L**^**1A**^ and **L**^**2A**^ was probed. As the reactions were carried out in buffered aqueous solutions, with the highest reactivity observed at the most basic pH (10), each ligand was modeled with both hydroxypyridinone moieties being deprotonated (for similar motifs,^3,26^ p*K*_a_(1) = 3.2–3.6, p*K*_a_(2) = 9.4–9.8). To charge-balance with the metal, an additional hydroxide ligand was modeled as coordinating to the In^3+^ center, which was strongly thermodynamically favored over the charge-separated analogues (see the Supporting Information for discussion of [In-**L**^**B**^][OH] species).

**Figure 5:**
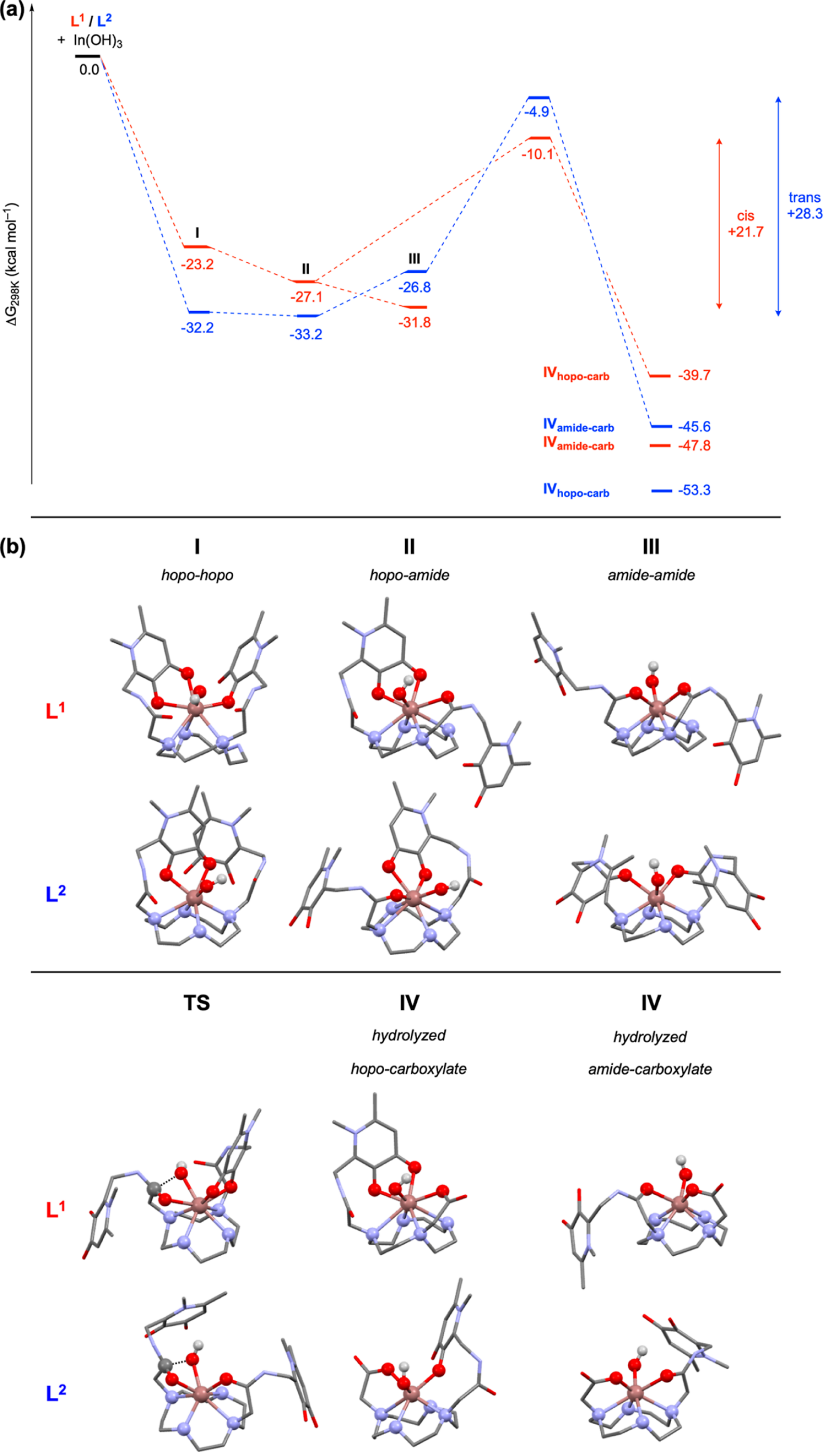
(a) Calculated pathway for intramolecular hydrolysis of In-**L**^**A**^ to In-**L**^**B**^; (b) structures of **I–IV** and lowest energy TS for In^3+^ complexes with both **L**^**1**^ and **L**^**2**^ derivatives.

Each ligand **L**^**A**^ has two pendant arms, each with two possible metal coordination sites (hydroxypyridinone (hopo) and amide moieties). Therefore, there are three possible combinations assuming both arms and the cyclen ring coordinate to In^3+^ (vide infra): hopo–hopo (**I**), hopo–amide (**II**), and amide–amide (**III**), with the resulting complexes exhibiting coordination numbers of either seven or eight. Optimized stationary points were found for each conformation **I–III** for both In-**L**^**1A**^ and In-**L**^**2A**^ (Figure 5). For both ligands, coordination to In^3+^ is thermodynamically favored (**L**^**1**^ = −23.2––31.8 kcal mol^–1^; **L**^**2**^ = −26.8––33.2 kcal mol^–1^), and all three conformations for each ligand set are separated by <10 kcal mol^–1^ (**L**^**1**^ = 8.6 kcal mol^–1^; **L**^**2**^ = 6.4 kcal mol^–1^), suggesting that each conformation should be thermodynamically accessible. In solution, In-**L**^**1A**^ and In-**L**^**2A**^ likely exist as mixtures of **I–III**, and In-**L**^**2A**^ appears more thermodynamically stable than In-**L**^**1A**^. In all cases, In^3+^ is also coordinated to three or four of the nitrogen atoms within the cyclen ring with various strengths (WBIs: 0.08–0.42 but typically 0.10–0.20). Interestingly, for In-**L**^**1A**^, the lowest energy conformation was found to be **III** (amide–amide), while for In-**L**^**2A**^, the lowest energy conformation was **II** (hopo–amide). For the lowest energy conformations of **I** (for both In-**L**^**1A**^ and In-**L**^**2A**^), only one hopo group coordinated in a bidentate fashion, with the second hopo group either coordinating via only one O atom or remaining uncoordinated.

Next, the coordination of hydrolyzed **L**^**B**^ to In^3+^ was investigated. Again, the hydroxypyridinone moiety and carboxylic acid were deprotonated, and a bound hydroxide was included. Various starting conformers were deliberately picked as input geometries (see the Supporting Information), and the resultant complex was allowed to freely optimize. In all cases, the carboxylate arm was found to coordinate with In^3+^ (WBIs: 0.20–0.47). For each ligand, two different conformers were found, in which either hopo or amide from the nonhydrolyzed arm coordinate to In^3+^, **IV**_**hopo-carb**_ and **IV**_**amide-carb**_, respectively (Figure 5). These species therefore represent In-**L**^**B**^ as discussed above. For In-**L**^**1B**^, the **IV**_**amide-carb**_ conformer is more stable than **IV**_**hopo-carb**_ by 8.1 kcal mol^–1^, while the opposite conformer is more stable for In-**L**^**2B**^ by 7.7 kcal mol^–1^. As with In-**L**^**A**^, **L**^**2**^ forms more stable hydrolyzed complexes than **L**^**1**^.

For each conformer **I–III**, with both ligand sets, an associated transition state (TS) for conversion to **IV** was found. The lowest energy TS for **L**^**1**^ was from **II**_**L1**_ to **IV**, while for **L**^**2**^, the lowest energy TS was from **III**_**L2**_. This results in an overall activation energy barrier (from the lowest energy conformer of In-**L**^**A**^ to the lowest TS) for **L**^**1**^ of +21.7 kcal mol^–1^, while **L**^**2**^ has a larger barrier of +28.3 kcal mol^–1^ (Figure 5). With a difference of 6.6 kcal mol^–1^ between the two ligands, the rate of hydrolysis using **L**^**1**^ is >12,000 times faster than with **L**^**2**^ at 80 °C.

The lowest energy TS for **L**^**1**^ has a hopo–amide coordination geometry, with the coordinated amide undergoing hydrolysis. This In^3+^-bound group is more activated toward hydrolysis than the corresponding amide moiety on the noncoordinated arm. The former is also closer to the In-bound hydroxide. The calculations reflect this asymmetry in the two amide groups of **L**^**1**^. The bound amide has a lower order C=O bond (WBIs: 1.52 vs 1.59) and a lower calculated C=O stretching frequency (ν_calc._ = 1716 vs 1729 cm^–1^). Due to its starting coordination environment, the likely initial hydrolysis product for **L**^**1**^ will be **IV**_**hopo-carb**_. On the other hand, the lowest energy TS for **L**^**2**^ has an amide–amide coordination geometry. In this case, either carbonyl may undergo hydrolysis (C=O WBIs: 1.41 and 1.43; one coupled C=O stretch at ν_calc._ = 1699 cm^–1^) and the likely hydrolysis product will be **IV**_**amide-carb**_. For both In-**L**^**1B**^ and In-**L**^**2B**^, the initial hydrolysis product is not in its most thermodynamically stable form, and both may rearrange to **IV**_**amide-carb**_ and **IV**_**hopo-carb**_, respectively (Figure 5). For **IV**_**hopo-carb**_ structures for **L**^**1B**^, the hopo group coordinates in a bidentate fashion. For **IV**_**hopo-carb**_ structures for **L**^**2B**^, the hopo group coordinates via only one hydroxy O^3^ atom. This energy landscape shape is consistent across multiple functionals, both with and without added dispersion (see the Supporting Information).

Overall, the DFT conclusions corroborate the experimental results. Coordination of **L**^**A**^ to In^3+^ leads to a mixture of conformers, which may explain the apparent multiple species observed by NMR spectroscopy and HPLC. The lower overall TS barrier for the hydrolysis of **L**^**1**^ compared to that of **L**^**2**^ is consistent with the observation of rapid generation of In-**L**^**1B**^ from In-**L**^**1A**^, while the corresponding conversion of In-**L**^**2A**^ is slower, despite both being thermodynamically accessible.

## Discussion and Concluding Remarks

Metal ions and their complexes are widely known to catalyze or mediate hydrolysis of amide/peptide bonds, through either (i) coordination of an amide carbonyl group, and concurrent activation of the carbonyl toward nucleophilic attack, (ii) activation of a coordinated water or hydroxide ion to enable nucleophilic attack of an amide, or (iii) a combination of both of the above, in which an amide carbonyl and hydroxide are both coordinated to a metal center.^27^ Complexes of cyclen and oxacyclen derivatives with metal ions, significantly Cu^2+^ and Co^3+^, have demonstrated hydrolytic activity toward peptides.^28−30^

In our ligand systems, In^3+^-mediated hydrolysis is not simply a result of the presence of In^3+^ in solution or transient coordination of In^3+^ to these ligand systems. Experimental data strongly points to a hydrolysis pathway in which hydrolysis is mediated by the coordination of In^3+^ to **L**^**1**^ and **L**^**2**^ ligand systems.

- In^3+^ complexes of **L**^**1**^ and **L**^**2**^, [^nat^In]In-**L**^**1A**^ and [^nat^In]In-**L**^**2A**^, have been isolated and characterized, alongside their hydrolysis products, [^nat^In]In-**L**^**1B**^ and [^nat^In]In-**L**^**2B**^. Given that these complexes have been isolated, we hypothesize that it is unlikely that In^3+^ dissociates from these respective ligand systems during a hydrolysis process.
- At later time points in the reaction of **L**^**1**^ with ^111^In^3+^ at pH 8 and pH 10, minimal or negligible “unchelated” ^111^In^3+^ remains in solution—initial ^111^In^3+^ complexation is rapid. However, there continues to be an increase in the amount of [^111^In]In-**L**^**1B**^ over time, while simultaneously, there is a decrease in the amount of [^111^In]In-**L**^**1A**^.
- There is no evidence of “free” **L**^**1B**^ or **L**^**2B**^ ligand in studies with either ^nat^In^3+^ or ^111^In^3+^.
- DFT calculations indicate that In^3+^ coordination of an intramolecular amide carbonyl, as well as coordination of a hydroxide ion are likely, enabling an intramolecular hydrolysis mechanism (Figures 5 and S20), similar to that previously reported for cyclen and oxacyclen complexes.^27−30^

In developing new chelators based on cyclen, we have exemplified the importance of investigating the relative reactivities of different regioisomers with alternative amine substitution patterns.^22^ There are stark differences in the reactivity of the two different cyclen derivatives, **L**^**1**^ and **L**^**2**^, which differ only in the relative position of the two pendant amide-containing hydroxypyridinone arms. Formation of In-**L**^**2A**^ from solutions containing In^3+^ and **L**^**2**^ requires more basic reaction conditions than that required for the formation of In-**L**^**1A**^ (see Supporting Information, Section 7). Additionally, the reactivity of In-**L**^**2A**^ toward amide bond hydrolysis is lower than that of In-**L**^**1A**^. As a result, [^111^In]In-**L**^**1B**^ could be prepared in a near-quantitative radiochemical yield after 2 h of reaction at 80 °C and pH 10, while [^111^In]In-**L**^**2B**^ could only be prepared in a 56% radiochemical yield under the same conditions. In studying the reaction of both **L**^**1**^ and **L**^**2**^, under the reaction conditions employed, we found no experimental evidence of formation of “DO2A” complexes or derivatives, which would arise if both amide bonds of **L**^**1**^/**L**^**2**^ underwent hydrolysis. In addition, in preliminary studies, we have assessed the reaction of **L**^**1**^ and Ga^3+^: **L**^**1**^ complexes [^68/nat^Ga^3+^]Ga^3+^; however, no hydrolytic activity was observed (see the Supporting Information, Section 6).

Overall, the DFT conclusions corroborate experimental results. DFT suggests that coordination of **L**^**1**^ and **L**^**2**^ to In^3+^ leads to a mixture of species in which cyclen coordinates with In^3+^, alongside a combination of HOPO and/or amide O atoms. These multiple energetically accessible DFT structures are consistent with ^1^H NMR spectra and radio-HPLC data for both In-**L**^**1A**^ and In-**L**^**1B**^: multiple species are observed by NMR spectroscopy and radio-HPLC. In the case of In-**L**^**1A**^, the mixture of low energy structures is within 9 kcal mol^–1^ of each other, and for In-**L**^**2A**^, within 6 kcal mol^–1^. It is likely that these species interconvert with each other and that there is significant lability in this system.

Significantly, the calculated lower overall TS barrier for the hydrolysis of In-**L**^**1A**^ (21.7 kcal mol^–1^) compared to that of In-**L**^**2A**^ (28.3 kcal mol^–1^) is consistent with experimental observations: the conversion of In-**L**^**1A**^ to In-**L**^**1B**^ is relatively rapid compared to the conversion of In-**L**^**2A**^ to In-**L**^**2B**^, despite both reactions being thermodynamically accessible.

Multiple small radioactive ^111^In signals were observed in radio-HPLC chromatograms at early time points. Similar to other cyclen-based macrocycles including DOTA, it is likely that “out-of-cage” complexes^31^ are formed en route to [^111^In]In-**L**^**1A**^ and [^111^In]In-**L**^**2A**^, and it is also possible that these low-intensity signals correspond to “out-of-cage” species. However, the presence of these species decreased over time, concurrent with a decrease in [^111^In]In-**L**^**1A**^ and [^111^In]In-**L**^**2A**^, and we did not observe or isolate nonradioactive isotopologues of these species.

To the best of our knowledge, this report describes the first example of a chelator undergoing radiometal-mediated hydrolysis to yield a radiometalated complex. The documented behavior of **L**^**1**^ and **L**^**2**^ ligands and their resulting In^3+^ complexes is therefore unique in the radiochemical literature. It is possible that metal-mediated amide bond cleavage is a source of instability in some other radiopharmaceuticals or radiotracers, particularly as there are many bioconjugates in which a chelator is attached to a targeting biomolecule via an amide bond. A bisphosphonate conjugate of NOTA (1,4,7-triazacyclononane-*N*,*N*′,*N*″-triacetic acid), in which the bisphosphonate is appended to the macrocycle via an amide bond, has previously been developed for [^68^Ga^3+^]Ga^3+^ complexation. In studies with nonradioactive [^nat^Ga^3+^]Ga^3+^, hydrolysis of this amide bond was observed; preliminary radiolabeling data suggested that it is possible that hydrolysis occurs during radiochemical reactions.^32^ While there are instances of cyclen and cyclam derivatives possessing coordinating primary amide groups that enable stable chelation of large main-group metal ions, notably radioactive isotopes of Pb^2+^,^33,34^ there are also examples of chelator derivatives that contain coordinating secondary amides. X-ray diffraction (XRD) analysis has shown that secondary amide carbonyl groups in DOTA^35^ and DTPA derivatives^36^ coordinate with In^3+^ and other metal ions. These species are often radiolabeled by heating the bioconjugate with a radioactive metal ion under aqueous conditions, followed by radiochromatographic analysis, including radio-HPLC to assess whether or not a chelator binds to a particular radiometal. It is possible that such reactions have led to the hydrolysis of amide bonds in the past but that this reactivity has been unreported or overlooked. Our study highlights the importance of appropriate characterization of radiolabeled products, including isolation and characterization of nonradioactive isotopologues, and careful chromatographic correlation with their radioactive forms.

## Experimental Section

### Synthesis

Compound **2**: **1** (0.28 g, 1.1 mmol) was dissolved in DCM (8 mL), cooled to 0 °C, and stirred. K_2_CO_3_ (0.6 g, 10.7 mmol) dissolved in water (7 mL) and 2-chloroacetyl chloride (0.13 mL, 2.2 mmol) dissolved in DCM (3 mL) were both added simultaneously to the reaction mixture and stirred at 0 °C for 2 h and then overnight at room temperature. The organic layer was separated, and the aqueous layer was extracted with DCM (2 × 30 mL). The organic layers were combined, dried over anhydrous Na_2_SO_4_, filtered, and evaporated to dryness. The residue was purified by SiO_2_ column chromatography (MeOH/CHCl_3_, 5:95) to give **2** as a white solid (0.25 g, 70%). ^1^H NMR (400 MHz, CDCl_3_): δ 2.26 (s, 3H), 3.47 (s, 3H), 3.97 (s, 2H), 4.41 (s, 2H), 5.25 (s, 2H), 6.33 (s, 1H), 7.34 (m, 5H); ^13^C NMR (100 MHz, CDCl_3_): δ 21.0, 35.3, 36.2, 42.3, 73.1, 119.0, 128.4, 128.6, 129.0, 137.2, 139.8, 146.3, 147.3, 166.3, 173.2. Single crystals suitable for XRD analysis were obtained by slow evaporation from a solution of **2** in chloroform. Single-crystal XRD analysis was consistent with the proposed structure and spectroscopic data for **2** (see the Supporting Information).

Compound **L**^**1**^: to **3** (0.22 g, 1 mmol) dissolved in CH_3_CN (10 mL) were added **2** (0.65 g, 1.9 mmol), DIPEA (0.34 mL, 1.9 mmol), and MeOH (1 mL) to fully solubilize all reactants. The reaction mixture was stirred at 60 °C for 3 days. After this time, the reaction was cooled and evaporated to dryness before purifying by reversed-phase column chromatography (H_2_O/0.1% TFA (A)/CH_3_CN/0.1% TFA (B), 0–100% B over 20 CV). Fractions were analyzed by LC–MS, and pure fractions were combined and freeze-dried to give **5**. The resultant solid was dissolved in H_2_O (5 mL) and 10 M NaOH (5 mL) and stirred at 90 °C overnight. After cooling, the product was extracted with DCM (3 × 20 mL), dried over Na_2_SO_4_, and evaporated to dryness. This residue was finally dissolved in MeOH (10 mL), and Pd/C (10%, 10 mg) was added. The reaction flask was evacuated and refilled with H_2_ (2 × balloon) before being left under a H_2_ atmosphere and stirred overnight. After this time, the reaction was filtered over Celite and the solvent was removed. Purification was carried out by preparative reversed-phase HPLC (H_2_O/0.1% TFA (A)/CH_3_CN/0.1% TFA (B), 0–95% B over 20 min, 10 mL/min). Fractions were analyzed by LC–MS, and pure fractions were combined and freeze-dried to give **L**^**1**^ as a hygroscopic solid (68 mg, 12% yield over three steps). ^1^H NMR (400 MHz, CDCl_3_): δ 2.60 (s, 6H), 3.00–3.26 (br m, 16 H), 3.65 (s, 4H), 3.98 (s, 6H), 4.79 (s, 4H), 6.99 (s, 2H); ^13^C NMR (100 MHz, CDCl_3_): δ 21.1, 36.1, 39.0, 43.9, 44.9, 52.4, 52.6, 56.2, 114.3, 137.3, 145.9, 149.9, 164.9, 171.5; HR–ESI–MS: calcd for [C_28_H_44_N_8_O_6_ + H]^+^, 589.3457; found, 589.3455.

Compound **L**^**2**^: to **4** (0.19 g, 0.4 mmol) dissolved in CH_3_CN (10 mL) were added **2** (0.28 g, 0.8 mmol), DIPEA (0.15 mL, 0.8 mmol), and MeOH (1 mL) to fully solubilize all reactants. The reaction mixture was stirred at 60 °C for 3 days. After this time, the reaction was cooled and evaporated to dryness before purifying by reversed-phase column chromatography (H_2_O/0.1% TFA (A)/CH_3_CN/0.1% TFA (B), 0–100% B over 20 CV). Fractions were analyzed by LC–MS and pure fractions were combined and freeze-dried to give **6**. The resultant solid was dissolved in MeOH (10 mL), and Pd/C (10%, 10 mg) was added. The reaction flask was evacuated and refilled with H_2_ (2 × balloon) before being left under a H_2_ atmosphere and stirred overnight. After this time, the reaction was filtered over Celite and the solvent was removed. Purification was carried out by preparative reversed-phase HPLC (H_2_O/0.1% TFA (A)/CH_3_CN/0.1% TFA (B), 0–95% B over 20 min, 10 mL/min). Fractions were analyzed by LC–MS, and pure fractions were combined and freeze-dried to give **L**^**2**^ as a hygroscopic solid (145 mg, 59% yield over two steps). ^1^H NMR (400 MHz, CDCl_3_): δ 2.61 (s, 6H), 2.96–3.21 (br m, 16H), 3.53 (s, 4H), 3.99 (s, 6H), 4.81 (s, 4H), 7.05 (s, 2H); ^13^C NMR (100 MHz, CDCl_3_): δ 21.1, 36.1, 39.8, 44.3, 51.2, 56.2, 114.0, 139.9, 145.2, 150.4, 173.8; HR–ESI–MS: calcd for [C_28_H_44_N_8_O_6_ + H]^+^, 589.3457; found, 589.3454.

Compound **[**^**nat**^**In]In-L**^**1A**^: **L**^**1**^ (5 mg, 8.5 μmol) was dissolved in an ammonium acetate solution (0.2 M, pH 6, 2 mL), and InCl_3_ (2.1 mg, 9.5 μmol) was added. The reaction mixture was stirred and heated to 80 °C for 2 h. The complex was purified by semipreparative reversed-phase HPLC (H_2_O/0.1% TFA (A)/CH_3_CN/0.1% TFA (B), 0–25% B over 30 min, 4 mL/min). Pure fractions were combined and freeze-dried to give [^nat^In]In-**L**^**1A**^ as a white solid (2.9 mg, 50%). Retention time of the desired compound: 20.1 min. HR–ESI–MS: calcd for [C_28_H_42_N_8_O_6_In + H]^+^, 701.2266; found, 701.2275.

Compound **[**^**nat**^**In]In-L**^**1B**^: **L**^**1**^ (5 mg, 8.5 μmol) was dissolved in a sodium carbonate/sodium hydrogen carbonate solution (0.2 M, pH 10, 2 mL), and InCl_3_ (2.1 mg, 9.5 μmol) was added. The reaction mixture was stirred and heated to 80 °C for 2 h. The complex was purified by semipreparative reversed-phase HPLC (H_2_O/0.1% TFA (A)/CH_3_CN/0.1% TFA (B), 0–25% B over 30 min, 4 mL/min). Pure fractions were combined and freeze-dried to give **[**^nat^In]In-**L**^**1B**^ as a white solid (2.4 mg, 52%). Retention time of the desired compound: 12.1 min. HR–ESI–MS: calcd for [C_20_H_32_N_6_O5In + H]^+^, 551.1473; found, 551.1472.

Compound **[**^**nat**^**In]In-L**^**2A**^: **L**^**2**^ (13 mg, 22 μmol) was dissolved in a sodium hydrogen carbonate solution (0.2 M, pH 8, 5 mL) and InCl_3_ (5.4 mg, 24 μmol) was added. The reaction mixture was stirred and heated to 80 °C for 2 h. The complex was purified by semipreparative reversed-phase HPLC (H_2_O/0.1% TFA (A)/CH_3_CN/0.1% TFA (B), 0–25% B over 30 min, 4 mL/min). Pure fractions were combined and freeze-dried to give [^nat^In]In-**L**^**2A**^ as a white solid (3.2 mg, 20%). Retention time of the desired compound: 20.1 min. HR–ESI–MS: calcd for [C_28_H_42_N_8_O_6_In + H]^+^, 701.2266; found, 701.2281.

Compound **[**^**nat**^**In]In-L**^**2B**^: **L**^**2**^ (15 mg, 25 μmol) was dissolved in a sodium carbonate/sodium hydrogen carbonate solution (0.2 M, pH 10, 2 mL), and InCl_3_ (6.2 mg, 28 μmol) was added. The reaction mixture was stirred and heated to 80 °C for 16 h. The complex was purified by semipreparative reversed-phase HPLC (H_2_O/0.1% TFA (A)/CH_3_CN/0.1% TFA (B), 0–25% B over 30 min, 4 mL/min). Pure fractions were combined and freeze-dried to give [^nat^In]In-**L**^**2B**^ as a white solid (3.7 mg, 26%). Retention time of the desired compound: 12.1 min. HR–ESI–MS: calcd for [C_20_H_32_N_6_O5In + H]^+^, 551.1473; found, 551.1463.

### [^111^In]In^3+^ Radiolabeling

To an aqueous buffered solution (186 μL, 0.2 M of pH 6 ammonium acetate or pH 8 sodium bicarbonate or pH 10 sodium carbonate/sodium bicarbonate) were added either **L**^**1**^ or **L**^**2**^ (10 μL, 2 mM) and [^111^In]InCl_3_ (4 μL, 2–4 MBq, Mallinckrodt Medical B.V., Petten, the Netherlands) to give a final ligand concentration of 100 μM. Reactions were heated to 80 °C, and aliquots were analyzed by reverse-phase radio-HPLC at 10 min, 30 min, 1 h, and 2 h time points (H_2_O/0.1% TFA (A)/CH_3_CN/0.1% TFA (B), 0–25% B over 30 min, 25–95% over 5 min, 1 mL/min).

### [^nat^In]In^3+^ LC–MS Reaction Monitoring

To an aqueous buffered solution (186 μL, 0.2 M of pH 6 ammonium acetate or pH 8 sodium hydrogen carbonate or pH 10 sodium carbonate/sodium hydrogen carbonate) were added either **L**^**1**^ or **L**^**2**^ (10 μL, 2 mM) and [^nat^In]InCl_3_ (4 μL, 2.5 mM in 0.1 M HCl) to give a final ligand concentration of 100 μM. Reaction mixtures were heated to 80 °C and aliquots analyzed by reverse-phase LC–MS analysis after 1 h of reaction time (H_2_O/0.1% TFA (A)/CH_3_CN/0.1% TFA (B), 0–25% B over 30 min, 25–95% over 5 min 1 mL/min).

## Supporting Information Available

General materials and instrumentation; NMR and MS characterization data for ligands and complexes; crystal structure analysis of precursor **2**; hydrolysis mechanism pathway; gallium complexation and analysis; kinetic analysis; and details of computation modeling (PDF)
Coordinate data for computed structures (XYZ)

## Supplementary Material

ic3c00353_si_001.pdf

ic3c00353_si_002.xyz

## References

[ref1] Jackson J. A.; Hungnes I. N.; Ma M. T.; Rivas C. Bioconjugates of Chelators with Peptides and Proteins in Nuclear Medicine: Historical Importance, Current Innovations, and Future Challenges. Bioconjugate Chem. 2020, 31, 483–491. 10.1021/acs.bioconjchem.0c00015. 31990543

[ref2] Rivas C.; Jackson J. A.; Hungnes I. N.; Ma M. T. Radioactive Metals in Imaging and Therapy. Compr. Coord. Chem. III 2021, 9, 706–740. 10.1016/b978-0-08-102688-5.00010-6.

[ref3] Cusnir R.; Imberti C.; Hider R.; Blower P.; Ma M. Hydroxypyridinone Chelators: From Iron Scavenging to Radiopharmaceuticals for PET Imaging with Gallium-68. Int. J. Mol. Sci. 2017, 18, 116 10.3390/ijms18010116. 28075350 PMC5297750

[ref4] Gorden A. E. V.; Xu J.; Raymond K. N.; Durbin P. Rational Design of Sequestering Agents for Plutonium and Other Actinides. Chem. Rev. 2003, 103, 4207–4282. 10.1021/cr990114x. 14611263

[ref5] Imberti C.; Terry S. Y. A.; Cullinane C.; Clarke F.; Cornish G. H.; Ramakrishnan N. K.; Roselt P.; Cope A. P.; Hicks R. J.; Blower P. J.; Ma M. T. Enhancing PET Signal at Target Tissue in vivo: Dendritic and Multimeric Tris(Hydroxypyridinone) Conjugates for Molecular Imaging of αvβ3 Integrin Expression with Gallium-68. Bioconjugate Chem. 2017, 28, 481–495. 10.1021/acs.bioconjchem.6b00621. PMC5314429 27966893

[ref6] Ma M. T.; Cullinane C.; Imberti C.; Baguña Torres J.; Terry S. Y. A.; Roselt P.; Hicks R. J.; Blower P. J. New Tris(Hydroxypyridinone) Bifunctional Chelators Containing Isothiocyanate Groups Provide a Versatile Platform for Rapid One-Step Labeling and PET Imaging with ^68^Ga^3+^. Bioconjugate Chem. 2016, 27, 309–318. 10.1021/acs.bioconjchem.5b00335. PMC4759618 26286399

[ref7] Ma M. T.; Meszaros L. K.; Paterson B. M.; Berry D. J.; Cooper M. S.; Ma Y.; Hider R. C.; Blower P. J. Tripodal Tris(Hydroxypyridinone) Ligands for Immunoconjugate PET Imaging with ^89^Zr^4+^: Comparison with Desferrioxamine-B. Dalton Trans. 2015, 44, 4884–4900. 10.1039/c4dt02978j. 25351250 PMC4357251

[ref8] Deri M. A.; Ponnala S.; Kozlowski P.; Burton-Pye B. P.; Cicek H. T.; Hu C.; Lewis J. S.; Francesconi L. C. P-SCN-Bn-HOPO: A Superior Bifunctional Chelator for ^89^Zr ImmunoPET. Bioconjugate Chem. 2015, 26, 2579–2591. 10.1021/acs.bioconjchem.5b00572. PMC4962612 26550847

[ref9] Tinianow J. N.; Pandya D. N.; Pailloux S. L.; Ogasawara A.; Vanderbilt A. N.; Gill H. S.; Williams S. P.; Wadas T. J.; Magda D.; Marik J. Evaluation of a 3-Hydroxypyridin-2-one (2,3-HOPO) based Macrocyclic Chelator for ^89^Zr^4+^ and its use for ImmunoPET Imaging of HER2 Positive Model of Ovarian Carcinoma in Mice. Theranostics 2016, 6, 511–521. 10.7150/thno.14261. 26941844 PMC4775861

[ref10] Deblonde G. J. P.; Lohrey T. D.; Booth C. H.; Carter K. P.; Parker B. F.; Larsen Å.; Smeets R.; Ryan O. B.; Cuthbertson A. S.; Abergel R. J. Solution Thermodynamics and Kinetics of Metal Complexation with a Hydroxypyridinone Chelator Designed for Thorium-227 Targeted Alpha Therapy. Inorg. Chem. 2018, 57, 14337–14346. 10.1021/acs.inorgchem.8b02430. 30372069

[ref11] Bailey T. A.; Mocko V.; Shield K. M.; An D. D.; Akin A. C.; Birnbaum E. R.; Brugh M.; Cooley J. C.; Engle J. W.; Fassbender M. E.; Gauny S. G.; Lakes A. L.; Nortier F. M.; O’Brien E. M.; Thiemann S. L.; White F. D.; Vermeulen C.; Kozimor S. A.; Abergel R. J. Developing the ^134^Ce and ^134^La Pair as Companion Positron Emission Tomography Diagnostic Isotopes for ^225^Ac and ^227^Th Radiotherapeutics. Nat. Chem. 2020, 13, 284–289. 10.1038/s41557-020-00598-7. 33318671

[ref12] Carter K. P.; Shield K. M.; Smith K. F.; Jones Z. R.; Wacker J. N.; Arnedo-Sanchez L.; Mattox T. M.; Moreau L. M.; Knope K. E.; Kozimor S. A.; Booth C. H.; Abergel R. J. Structural and Spectroscopic Characterization of an Einsteinium Complex. Nature 2021, 590, 85–88. 10.1038/s41586-020-03179-3. 33536647

[ref13] Dai L.; Lo W. S.; Gu Y.; Xiong Q.; Wong K. L.; Kwok W. M.; Wong W. T.; Law G. L. Breaking the 1,2-HOPO Barrier with a Cyclen Backbone for More Efficient Sensitization of Eu(III) Luminescence and Unprecedented Two-Photon Excitation Properties. Chem. Sci. 2019, 10, 4550–4559. 10.1039/c9sc00244h. 31123564 PMC6498141

[ref14] Ma M. T.; Cullinane C.; Waldeck K.; Roselt P.; Hicks R. J.; Blower P. J. Rapid Kit-Based ^68^Ga-Labelling and PET Imaging with THP-Tyr^3^-Octreotate: A Preliminary Comparison with DOTA-Tyr^3^-Octreotate. EJNMMI Res. 2015, 5, 52 10.1186/s13550-015-0131-1. 26452495 PMC4600075

[ref15] Young J. D.; Abbate V.; Imberti C.; Meszaros L. K.; Ma M. T.; Terry S. Y. A.; Hider R. C.; Mullen G. E.; Blower P. J. 68Ga-THP-PSMA: A PET Imaging Agent for Prostate Cancer Offering Rapid, Room-Temperature, 1-Step Kit-Based Radiolabeling. J. Nucl. Med. 2017, 58, 1270–1277. 10.2967/jnumed.117.191882. 28408532 PMC6175039

[ref16] Kulkarni M.; Hughes S.; Mallia A.; Gibson V.; Young J.; Aggarwal A.; Morris S.; Challacombe B.; Popert R.; Brown C.; Cathcart P.; Dasgupta P.; Warbey V. S.; Cook G. J. R. The Management Impact of ^68^Gallium-Tris(Hydroxypyridinone) Prostate-Specific Membrane Antigen (^68^Ga-THP-PSMA) PET-CT Imaging for High-Risk and Biochemically Recurrent Prostate Cancer. Eur. J. Nucl. Med. Mol. Imaging 2020, 47, 674–686. 10.1007/s00259-019-04643-7. 31872280 PMC7005085

[ref17] Shi W.; Johnston C. F.; Buchanan K. D.; Ferguson W. R.; Laird J. D.; Crothers J. G.; McIlrath E. M. Localization of neuroendocrine tumours with [111In] DTPA-octreotide scintigraphy (Octreoscan): a comparative study with CT and MR imaging. QJM 1998, 91, 295–301. 10.1093/qjmed/91.4.295. 9666953

[ref18] Zhang Z.; Rettig S. J.; Orvig C. Lipophilic Coordination Compounds: Aluminum, Gallium, and Indium Complexes of 1-Aryl-3-Hydroxy-2-Methyl-4-Pyridinones. Inorg. Chem. 1991, 30, 509–515. 10.1021/ic00003a031.

[ref19] Clevette D. J.; Lyster D. M.; Nelson W. O.; Rihela T.; Webb G. A.; Orvig C. Solution Chemistry of Gallium and Indium 3-Hydroxy-4-Pyridinone Complexes in Vitro and in Vivo. Inorg. Chem. 1990, 29, 667–672. 10.1021/ic00329a021.

[ref20] Ellis B. L.; Duhme A. K.; Hider R. C.; Bilayet Hossain M.; Rizvi S.; van der Helm D. Synthesis, Physicochemical Properties, and Biological Evaluation of Hydroxypyranones and Hydroxypyridinones: Novel Bidentate Ligands for Cell-Labeling. J. Med. Chem. 1996, 39, 3659–3670. 10.1021/jm960220g. 8809155

[ref21] Green D. E.; Ferreira C. L.; Stick R. V.; Patrick B. O.; Adam M. J.; Orvig C. Carbohydrate-Bearing 3-Hydroxy-4-Pyridinonato Complexes of Gallium(III) and Indium(III). Bioconjugate Chem. 2005, 16, 1597–1609. 10.1021/bc0501808. 16287260

[ref22] Garda Z.; Forgács A.; Do Q. N.; Kálmán F. K.; Timári S.; Baranyai Z.; Tei L.; Tóth I.; Kovács Z.; Tircsó G. Physico-chemical properties of MnII complexes formed with cis- and trans-DO2A: thermodynamic, electrochemical and kinetic studies. J. Inorg. Biochem. 2016, 163, 206–213. 10.1016/j.jinorgbio.2016.07.018. 27567150

[ref23] Zhang M. X.; Zhu C. F.; Zhou Y. J.; Kong X. L.; Hider R. C.; Zhou T. Design, Synthesis, and Antimicrobial Evaluation of Hexadentate Hydroxypyridinones with High Iron(III) Affinity. Chem. Biol. Drug Des. 2014, 84, 659–668. 10.1111/cbdd.12358. 24890019

[ref24] Bellouard F.; Chuburu F.; Kervarec N.; Toupet L.; Triki S.; Le Mest Y.; Handel H. Cis-Diprotected Cyclams and Cyclens: A New Route to Symmetrically or Asymmetrically 1,4-Disubstituted Tetraazamacrocycles and to Asymmetrically Tetrasubstituted Derivatives. J. Chem. Soc., Perkin Trans. 1 1999, 23, 3499–3505. 10.1039/a905701c.

[ref25] De León-Rodríguez L. M.; Kovacs Z.; Esqueda-Oliva A. C.; Miranda-Olvera A. D. Highly Regioselective N-Trans Symmetrical Diprotection of Cyclen. Tetrahedron Lett. 2006, 47, 6937–6940. 10.1016/j.tetlet.2006.07.135.

[ref26] Imberti C.; Chen Y. L.; Foley C. A.; Ma M. T.; Paterson B. M.; Wang Y.; Young J. D.; Hider R. C.; Blower P. J. Tuning the properties of tris(hydroxypyridinone) ligands: efficient 68Ga chelators for PET imaging. Dalton Trans. 2019, 48, 4299–4313. 10.1039/c8dt04454f. 30860215 PMC6469224

[ref27] Grant K.; Kassai M. Major Advances in the Hydrolysis of Peptides and Proteins by Metal Ions and Complexes. Curr. Org. Chem. 2006, 10, 1035–1049. 10.2174/138527206777435535.

[ref28] Norjmaa G.; Solé-Daura A.; Besora M.; Ricart J. M.; Carbó J. J. Peptide Hydrolysis by Metal (Oxa)Cyclen Complexes: Revisiting the Mechanism and Assessing Ligand Effects. Inorg. Chem. 2021, 60, 807–815. 10.1021/acs.inorgchem.0c02859. 33411534

[ref29] Zhang T.; Zhu X.; Prabhakar R. Peptide Hydrolysis by Metal-Cyclen Complexes and Their Analogues: Insights from Theoretical Studies. Organometallics 2014, 33, 1925–1935. 10.1021/om400903r.

[ref30] Perera-Bobusch C.; Hormann J.; Weise C.; Wedepohl S.; Dernedde J.; Kulak N. Significantly Enhanced Proteolytic Activity of Cyclen Complexes by Monoalkylation. Dalton Trans. 2016, 45, 10500–10504. 10.1039/c6dt00681g. 27277522

[ref31] Moreau J.; Guillon E.; Pierrard J. C.; Rimbault J.; Port M.; Aplincourt M. Complexing Mechanism of the Lanthanide Cations Eu3+, Gd3+, and Tb3+ with 1,4,7,10-Tetrakis(carboxymethyl)-1,4,7,10-tetraazacyclododecane (dota)-Characterization of Three Successive Complexing Phases: Study of the Thermodynamic and Structural Properties of the Complexes by Potentiometry, Luminescence Spectroscopy, and EXAFS. Chem.—Eur. J. 2004, 10, 5218–5232. 10.1002/chem.200400006. 15372580

[ref32] Holub J.; Meckel M.; Kubíček V.; Rösch F.; Hermann P. Gallium(III) complexes of NOTA-bis (phosphonate) conjugates as PET radiotracers for bone imaging. Contrast Media Mol. Imaging 2015, 10, 122–134. 10.1002/cmmi.1606. 24801892

[ref33] Cuenot F.; Meyer M.; Espinosa E.; Guilard R. Synthesis, Characterization, and X-ray Crystal Structures of Cyclam Derivatives. 8. Thermodynamic and Kinetic Appraisal of Lead(II) Chelation by Octadentate Carbamoyl-Armed Macrocycles1. Inorg. Chem. 2005, 44, 7895–7910. 10.1021/ic0508019. 16241139

[ref34] Chappell L. L.; Dadachova E.; Milenic D. E.; Garmestani K.; Wu C.; Brechbiel M. W. Synthesis, Characterization, and Evaluation of a Novel Bifunctional Chelating Agent for the Lead Isotopes ^203^Pb and ^212^Pb. Nucl. Med. Biol. 2000, 27, 93–100. 10.1016/s0969-8051(99)00086-4. 10755652

[ref35] Liu S.; He Z.; Hsieh W. Y.; Fanwick P. E. Synthesis, Characterization, and X-Ray Crystal Structure of In(DOTA-AA) (AA = p-Aminoanilide): A Model for ^111^In-Labeled DOTA-Biomolecule Conjugates. Inorg. Chem. 2003, 42, 8831–8837. 10.1021/ic0349914. 14686864

[ref36] Hsieh W. Y.; Liu S. Synthesis, Characterization, and Structures of Indium In(DTPA-BA_2_) and Yttrium Y(DTPA-BA_2_)(CH_3_OH) Complexes (BA = Benzylamine): Models for ^111^In- and ^90^Y-Labeled PTPA-Biomolecule Conjugates. Inorg. Chem. 2004, 43, 6006–6014. 10.1021/ic049973g. 15360250

