# Supplementary material for: Probing Unexpected Reactivity in Radiometal Chemistry: Indium-111-Mediated Hydrolysis of Hybrid Cyclen-Hydroxypyridinone Ligands
Source: Inorg Chem. 2023 Mar 17;62(13):5270–81. doi: 10.1021/acs.inorgchem.3c00353 (PMC10074387; doi:10.1021/acs.inorgchem.3c00353)
Supplement: Supplementary file 1 — ic3c00353_si_001.pdf [file ic3c00353_si_001.pdf]

Probing unexpected reactivity in radiometal chemistry: indium-111 mediated hydrolysis of hybrid cyclen-hydroxypyridinone ligands

Supporting Information

Charlotte Rivas[1], Jessica A. Jackson[1], Alex Rigby[1], James A. Jarvis[2], Andrew J. P. White[3], Philip J. Blower[1], Andreas Phanopoulos*[3], Michelle T. Ma*[1]*

[1] King's College London, School of Biomedical Engineering and Imaging Sciences, 4th Floor Lambeth Wing, St Thomas' Hospital, London, United Kingdom, SE1 7EH

[2] Randall Centre of Cell and Molecular Biophysics and Centre for Biomolecular Spectroscopy, King's College London, London, United Kingdom, SE1 9RT

[3] Department of Chemistry, Imperial College London, Molecular Sciences Research Hub, London, United Kingdom, W12 0BZ

*Corresponding authors: Charlotte Rivas, King's College London, charlotte.rivas@kcl.ac.uk; Andreas Phanopoulos, Imperial College London, a.phanopoulos11@imperial.ac.uk; Michelle Ma, King's College London, michelle.ma@kcl.ac.uk

Contents

1. General Experimental......S1
2. NMR Characterisation...... S2
3. MS Characterisation......S9
4. X-ray Crystallography......S15
5. Hydrolysis Mechanism......S16
6. Gallium Complexation......S16
7. Kinetic Analysis......S20
8. Computational Modelling......S22

1. General Experimental

All chemicals and solvents were purchased from commercial suppliers and used without purification unless stated (Merck, Fisher Scientific, Fluorochem). Flash chromatography purification was performed on a Biotage Isolera 4 system using Sfar chromatography columns (silica and C18). Semi-preparative HPLC purification was performed on an Agilent Prostar system using a Zorbax Eclipse C18 column (21.2 x 250 mm, 5μm). ^{1}H, $^{13}C\{^{1}H\}$, HSQC NMR data were acquired on Bruker 400 or 700 MHz machines with data being processed using MestReNova (700 MHz machine equipped with an AVIII console and a quadruple-resonance QCI cryoprobe). LC-ESI-MS data were acquired on an Agilent 1200 Series Liquid Chromatograph with UV spectroscopic detection at 254 nm, interfaced with an Advion Expression LC-MS mass spectrometer with electrospray ionisation source. The mobile phase used for LC-MS composed of H_2O (0.1% formic acid): A and CH_3CN (0.1% formic acid): B running on an Eclipse XDB-C18 column (4.6 x 150 mm, 5 μm). High resolution electrospray mass spectrometry was carried out by Dr Lisa Haigh (MS service at Imperial College London). X-ray diffraction analysis were carried out by Dr. Andrew White of the Department of Chemistry at Imperial College London. Data for compound **2** was collected using an Agilent Xcalibur PX Ultra A diffractometer, and the structure was refined using the SHELXTL and SHELX-2013 program systems.[1,2] Radioactive samples were measured using a Capintec CRC25R dose calibrator. Radio-analytical HPLC was performed on an Agilent 1260 LC system with Laura software (UV spectroscopic detection at 254 nm), a Rheodyne sample loop (200 μL) and Lablogic Flow-Count detector with a sodium iodide probe (B-FC-3200) for radiation detection using an Eclipse XDB-C18 column (4.6 x 150 mm, 5 μm).

2. NMR characterisation

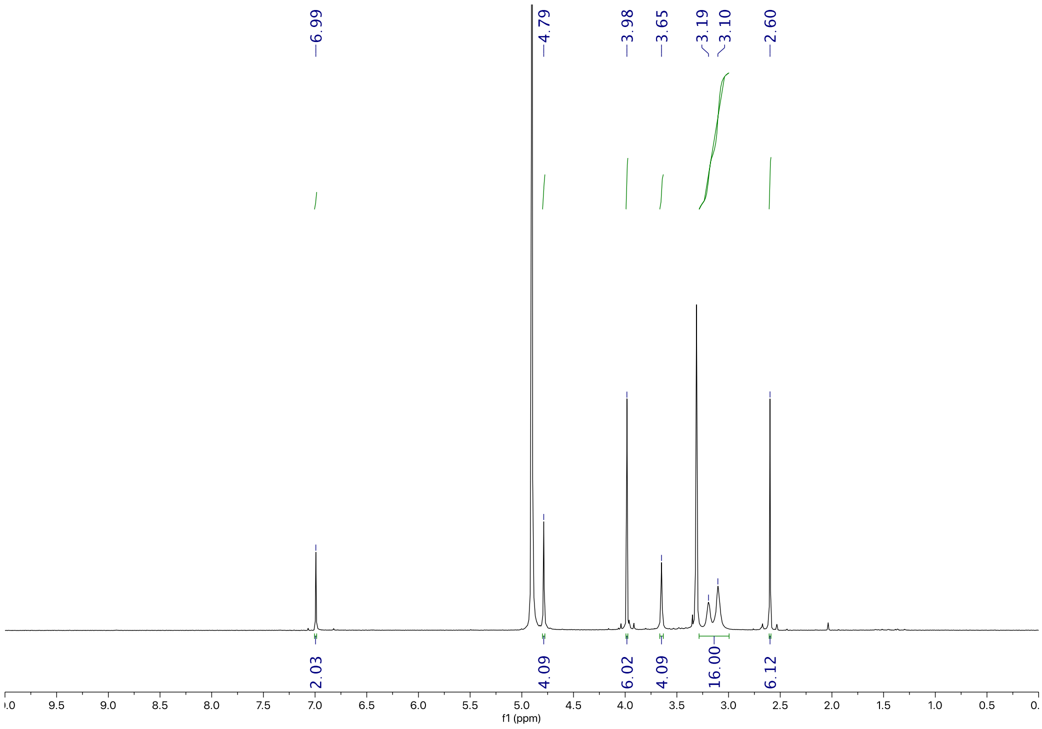

Figure S1. Compound ***L¹*** *^{1}H NMR spectrum (400 MHz, 298 K, MeOD)*

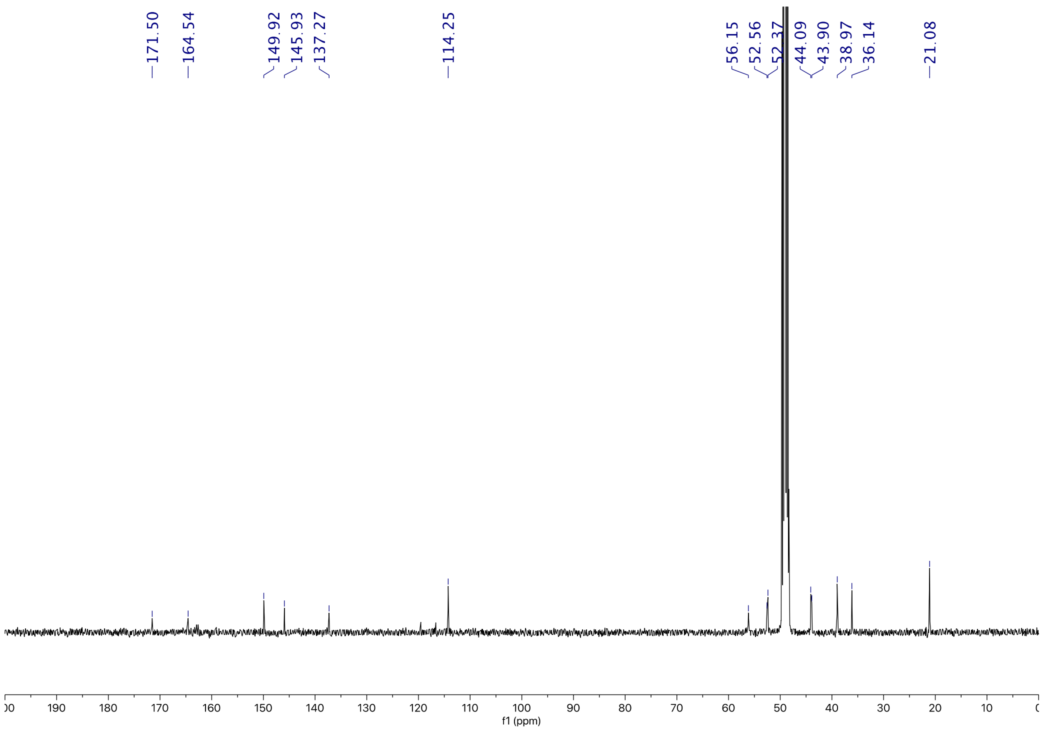

Figure S2. Compound ***L¹*** *^{13}C{^{1}H} NMR spectrum (101 MHz, 298 K, MeOD)*

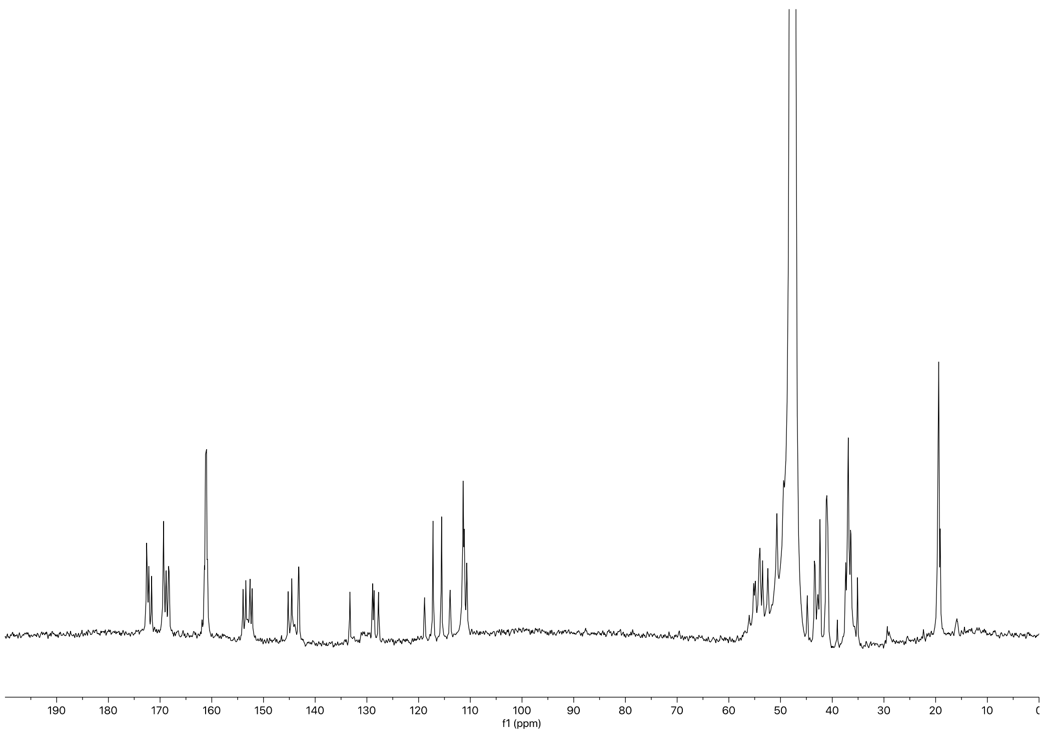

*Figure S3. Compound In-**L^{1A}** $^{13}C\{^1H\}$ NMR spectrum (175 MHz, 298 K, MeOD)*

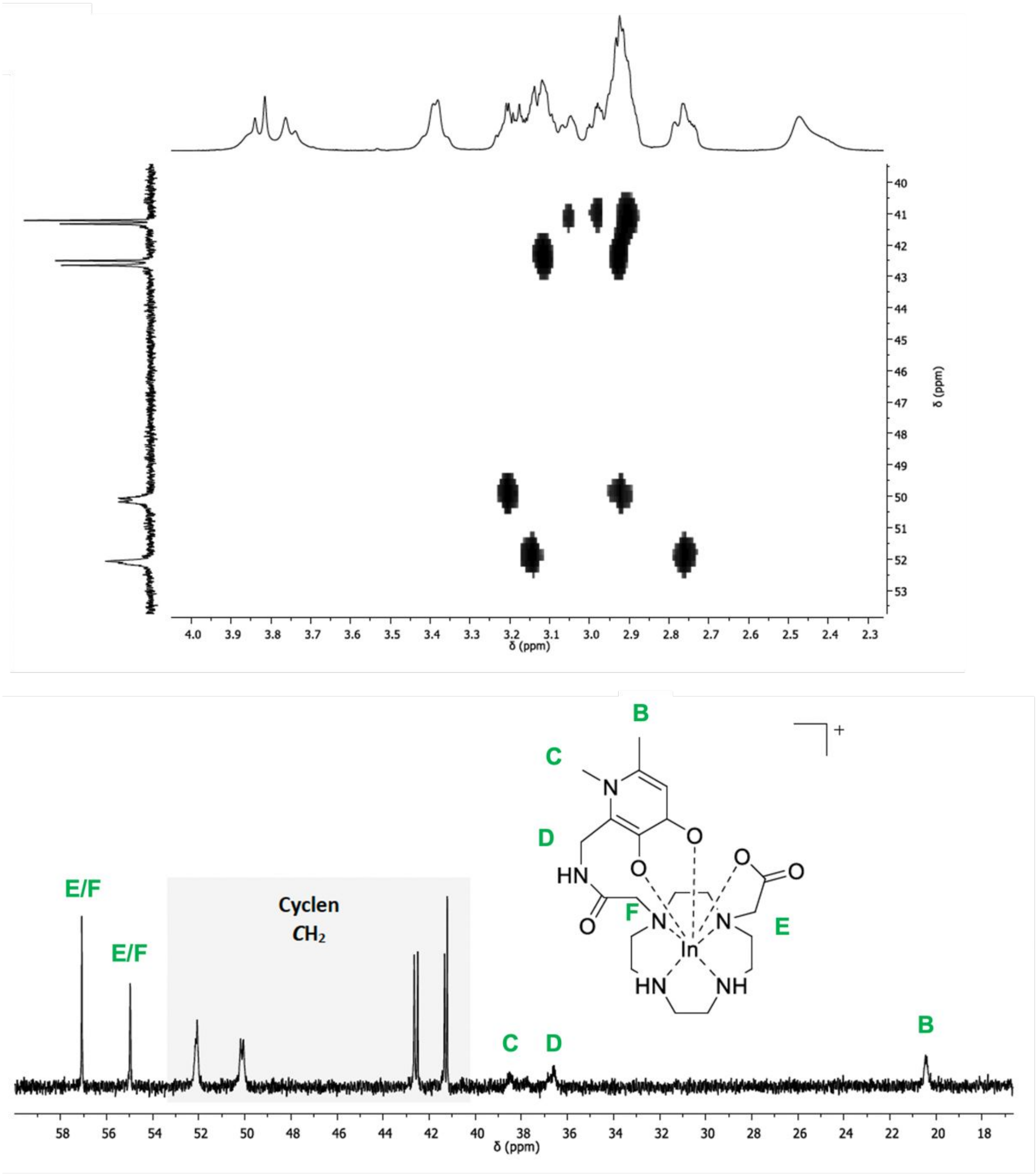

*Figure S4. Compound In-**L**[1B]: ^{1}H-^{13}C HSQC NMR spectrum of cyclen ring resonances (top); $^{13}C\{^{1}H\}$ NMR spectrum (bottom) (175 MHz, 298 K, D_2O).*

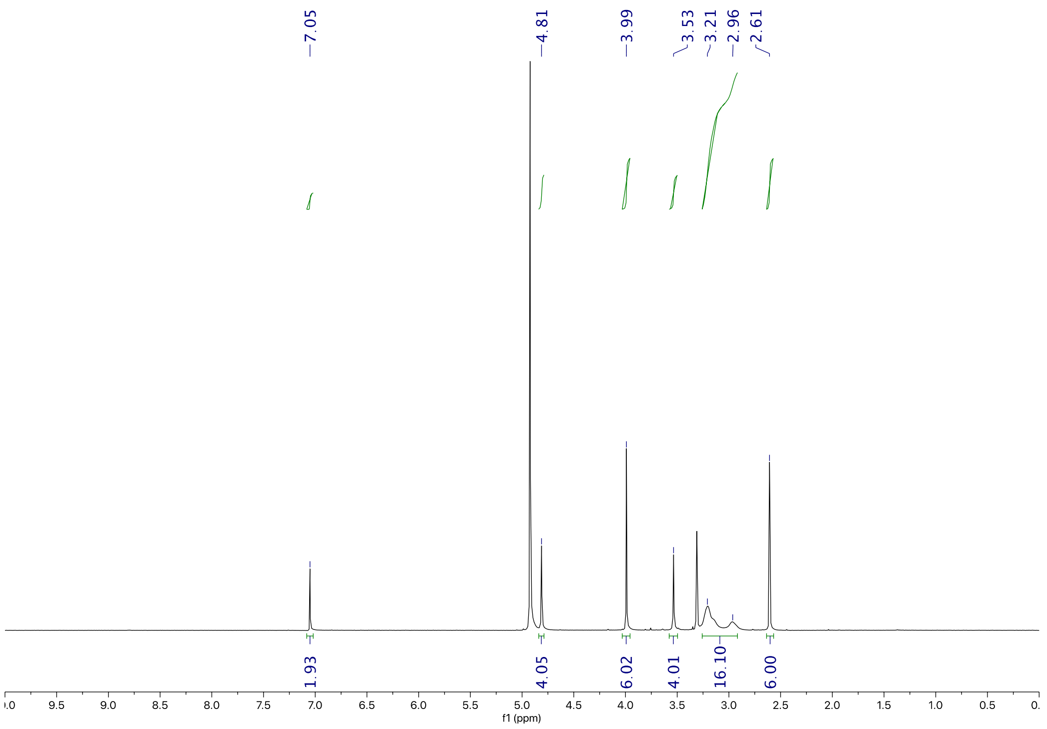

*Figure S5. Compound **L²** ^{1}H NMR spectrum (400 MHz, 298 K, MeOD)*

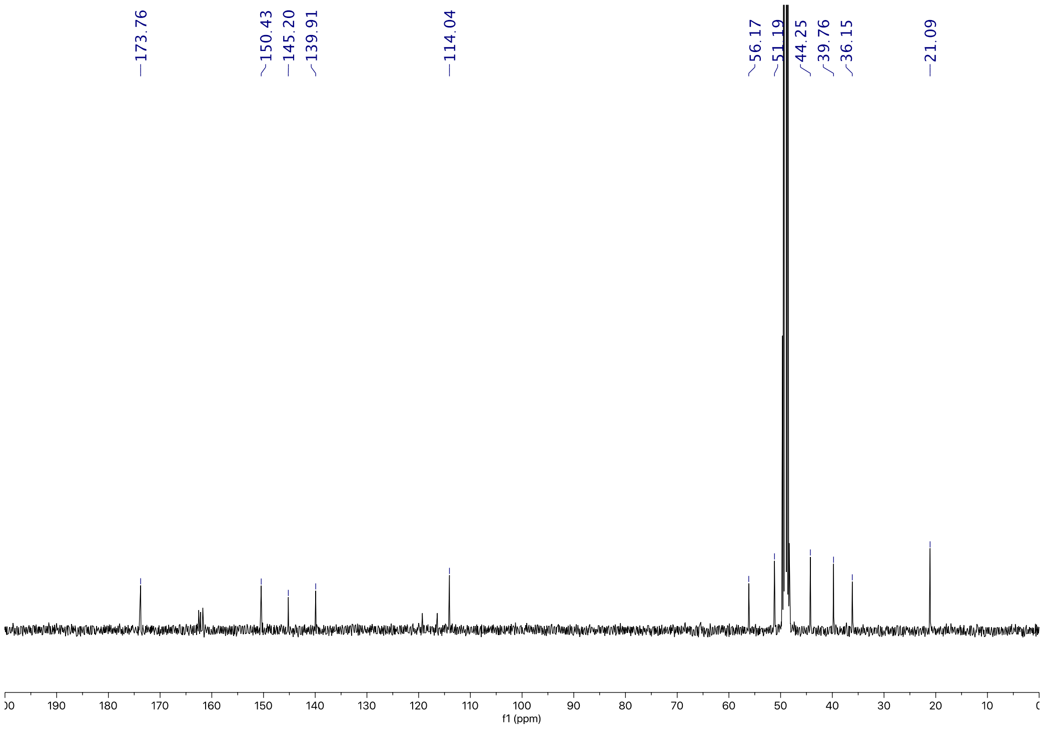

*Figure S6. Compound **L²** ^{13}C{^{1}H} NMR spectrum (101 MHz, 298 K, MeOD)*

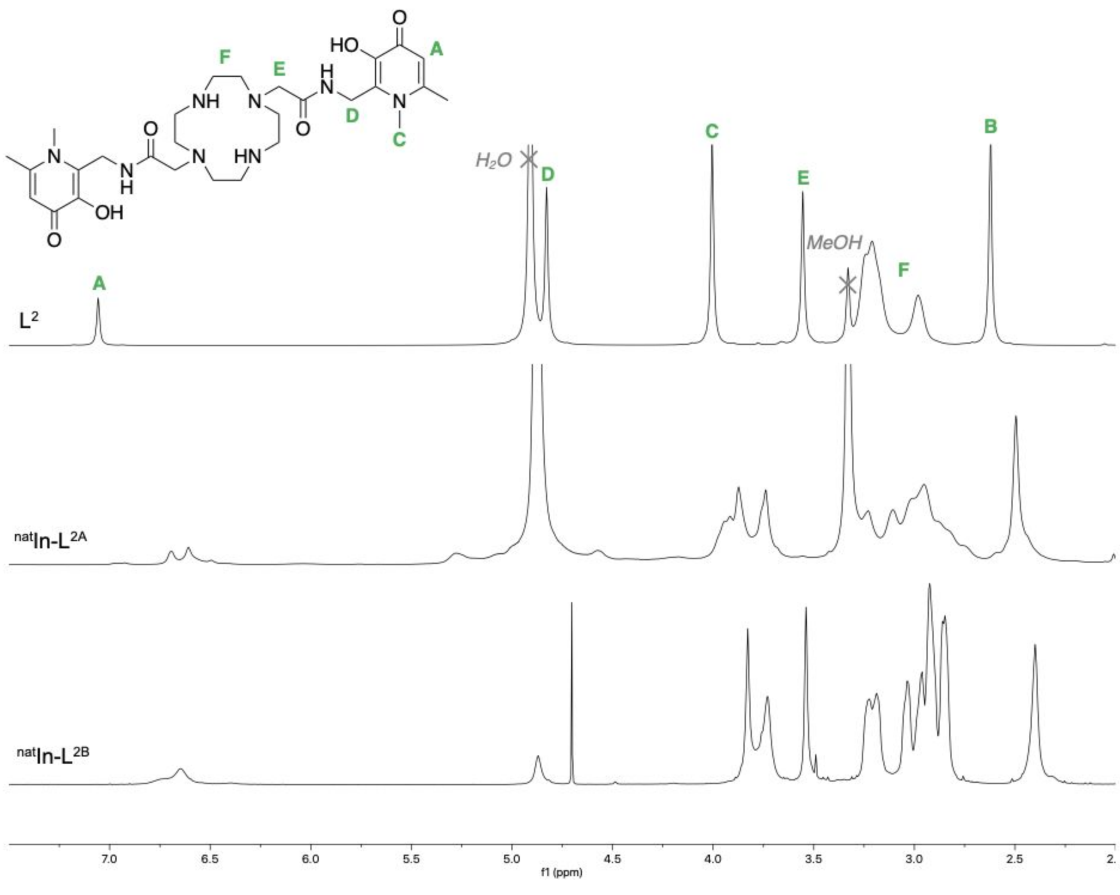

*Figure S7. ^{1}H NMR spectrum (700 MHz, 298 K) of **L^2** (MeOD), **^{nat}In-L^{2A}** (MeOD) and **^{nat}In-L^{2B}** (D_2O).*

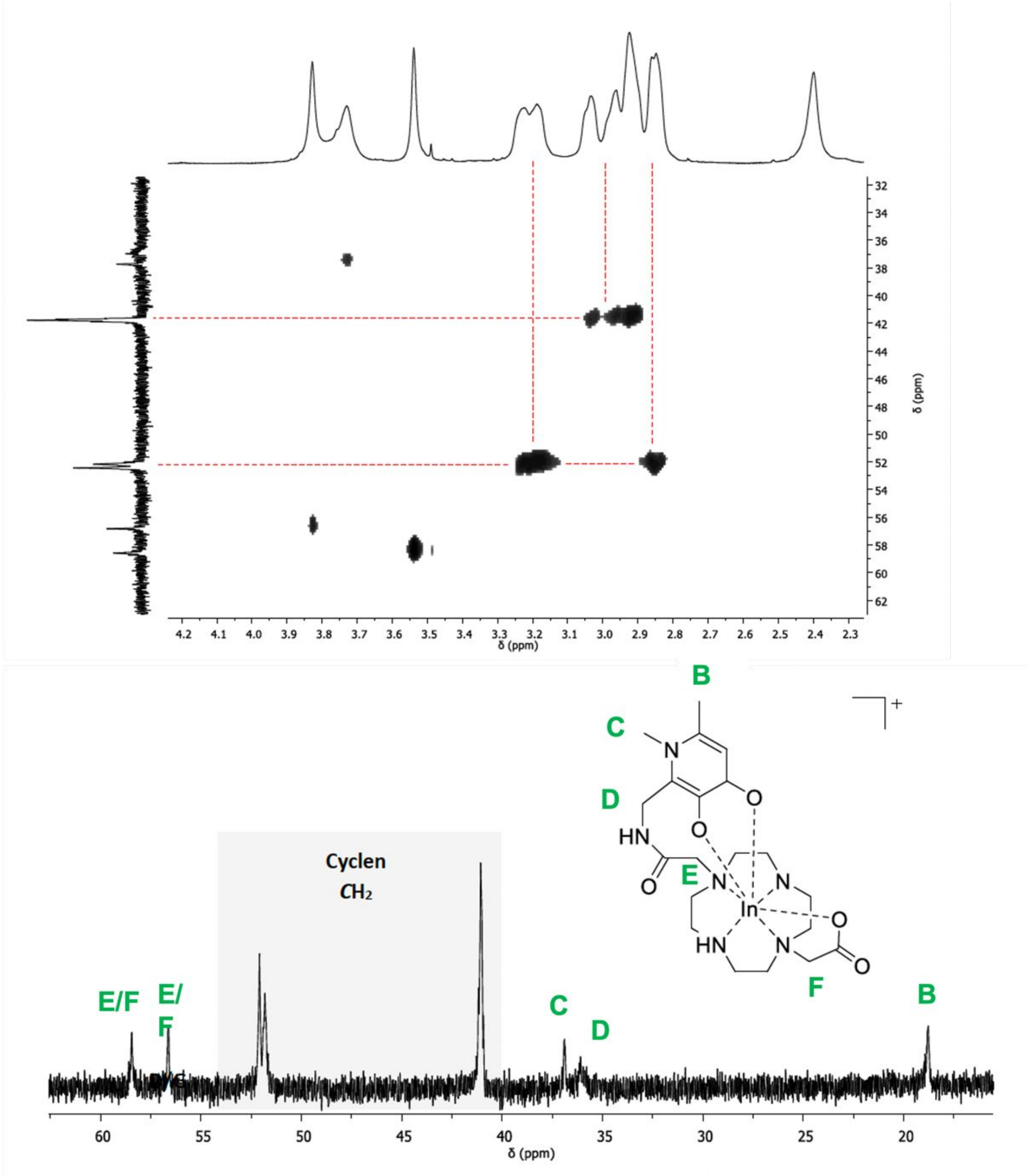

*Figure S8. Compound In-**L^1B**: ^{1}H-^{13}C HSQC NMR spectrum of cyclen ring resonances (top); ^{13}C{^{1}H} NMR spectrum (bottom) (175 MHz, 298 K, D_2O).*

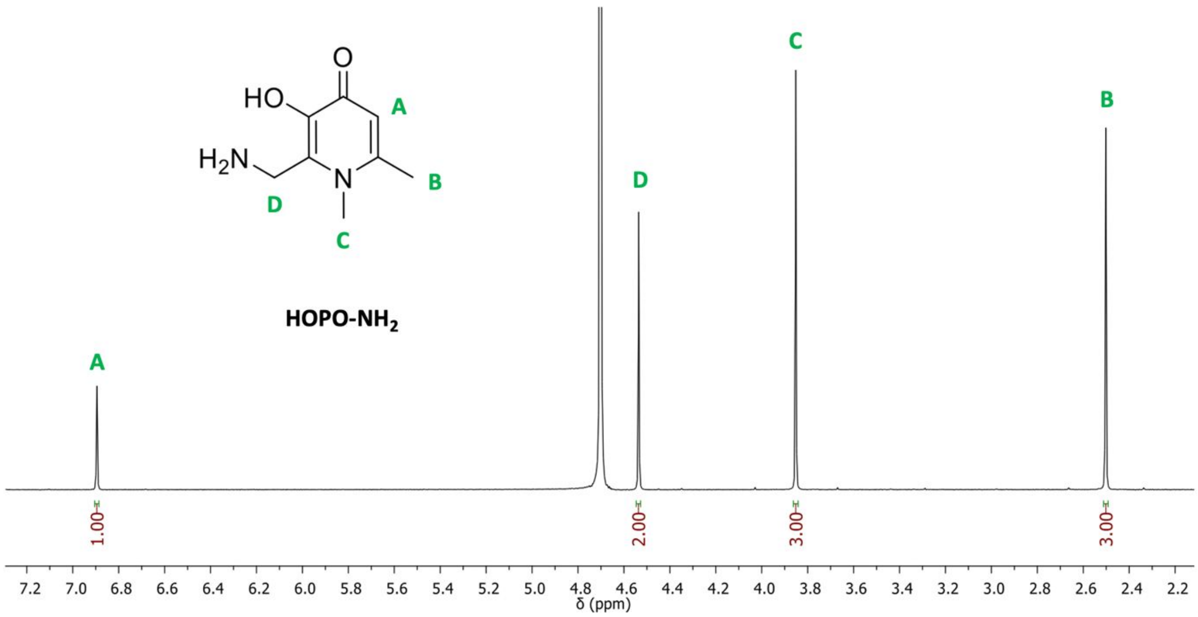

*Figure S9. Compound **$HOPO-NH_2$**: ^1H NMR spectrum (400 MHz, 298 K, D_2O). This sample of $HOPO-NH_2$ was isolated from a reaction in which an aqueous solution of **L^1** was reacted at 80 °C with $[^{nat}In]InCl_3$ at pH 10.*

3. MS characterisation

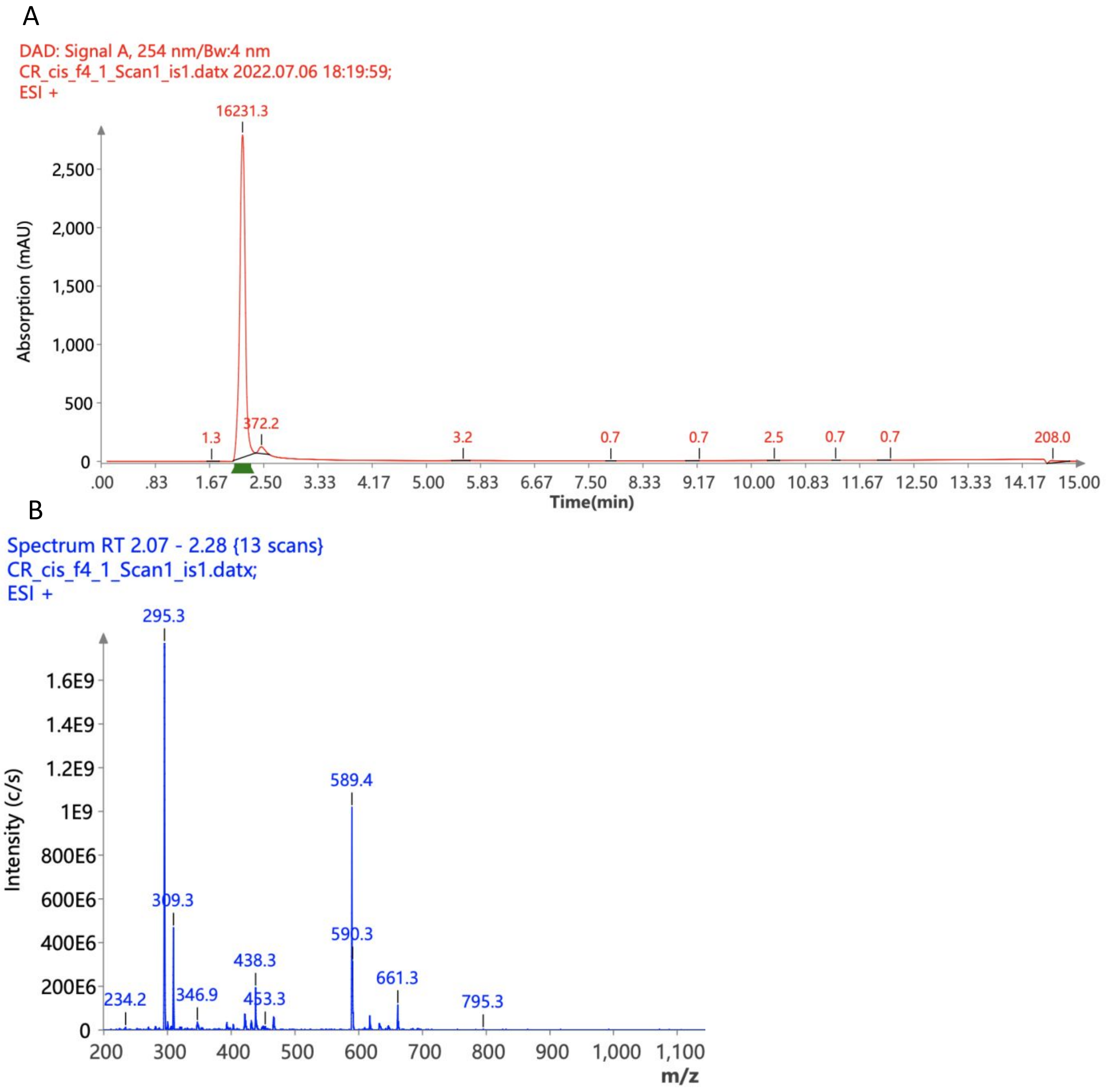

Figure S10. (A) LC-MS UV trace of **L^1**; *(B) MS associated with LC-MS trace at 2.07-2.28 min corresponding to m/z of* **L^1**.

A

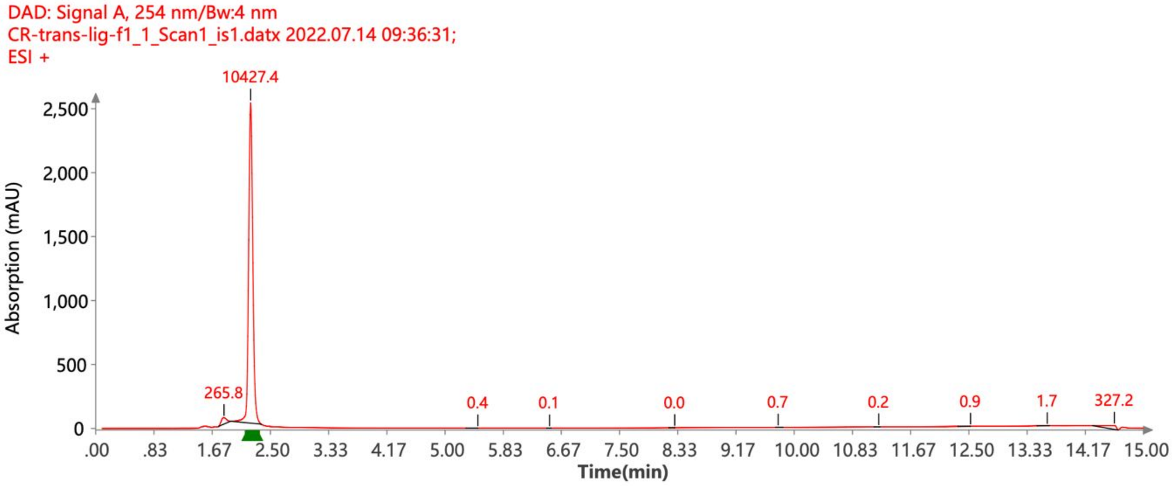

B

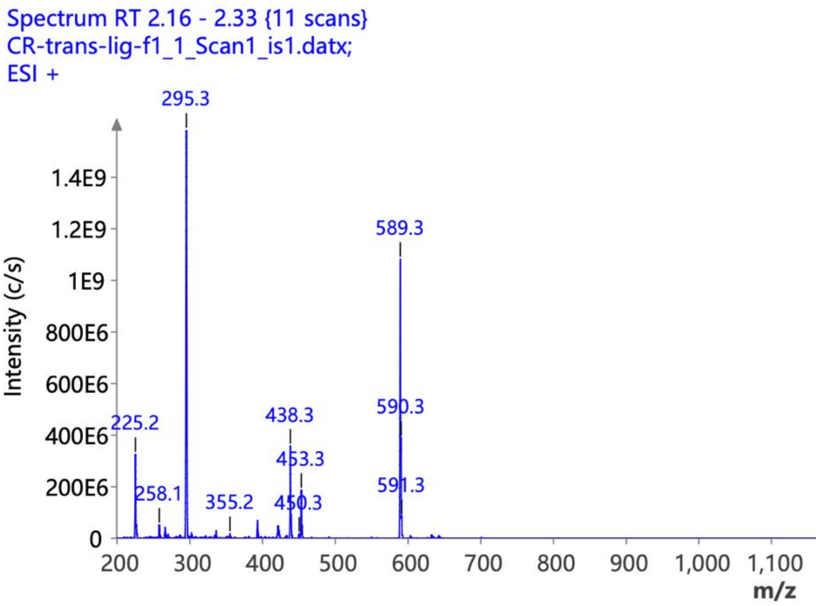

Figure S11. (A) LC-MS UV trace of $\boldsymbol{L^2}$*; (B) MS associated with LC-MS trace at 2.07-2.28 min corresponding to m/z of* $\boldsymbol{L^2}$*.*

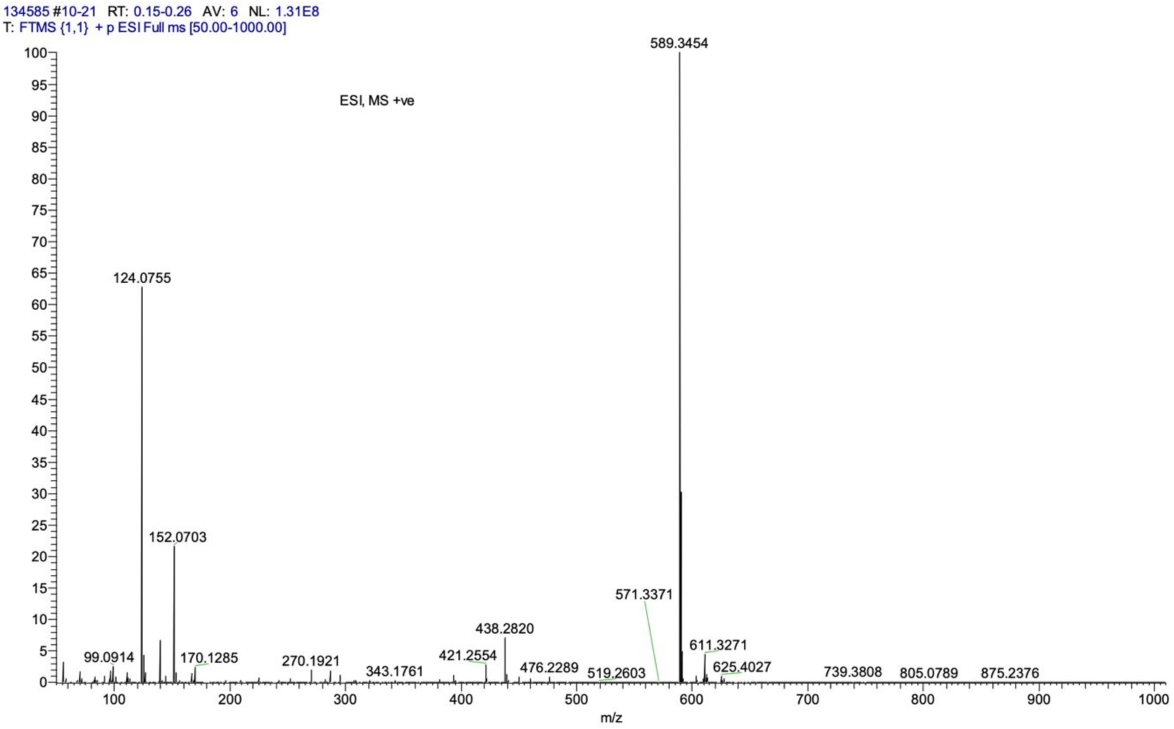

*Figure S12. HR-MS of compound **L1**.*

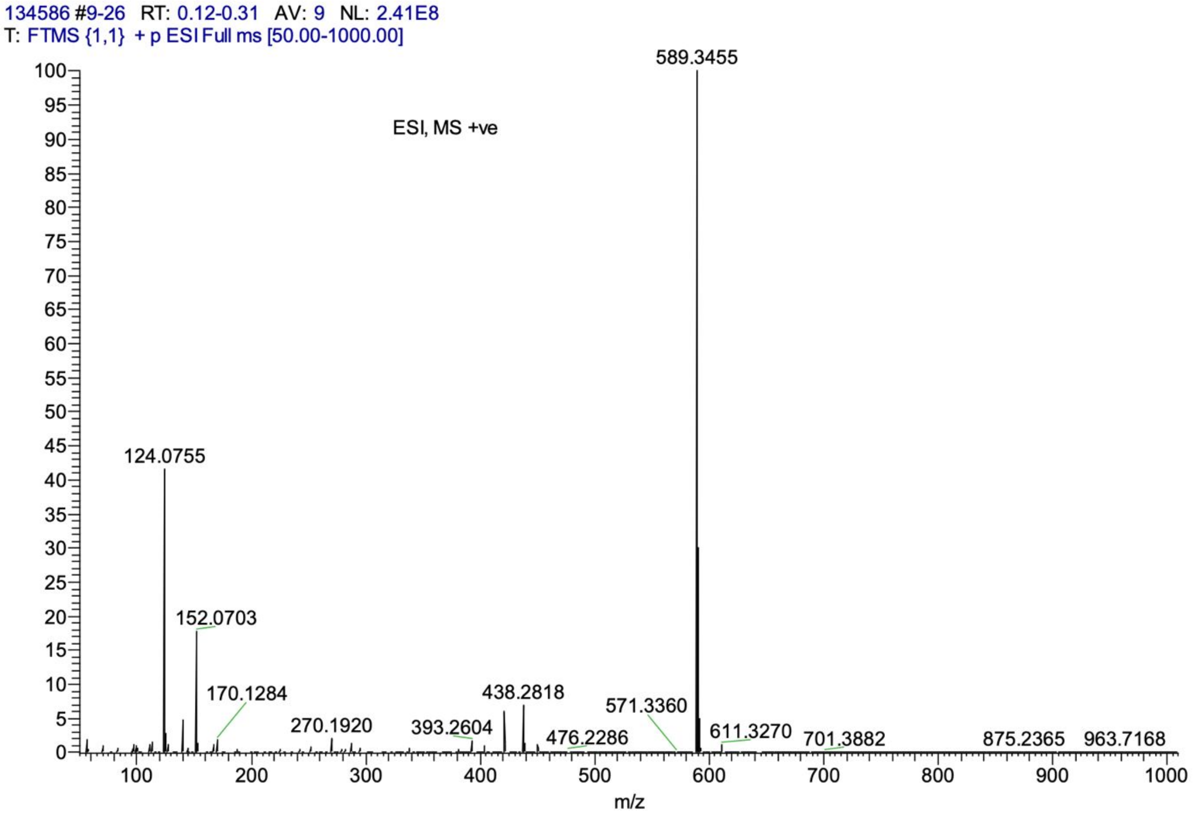

*Figure S13. HR-MS of compound **L2**.*

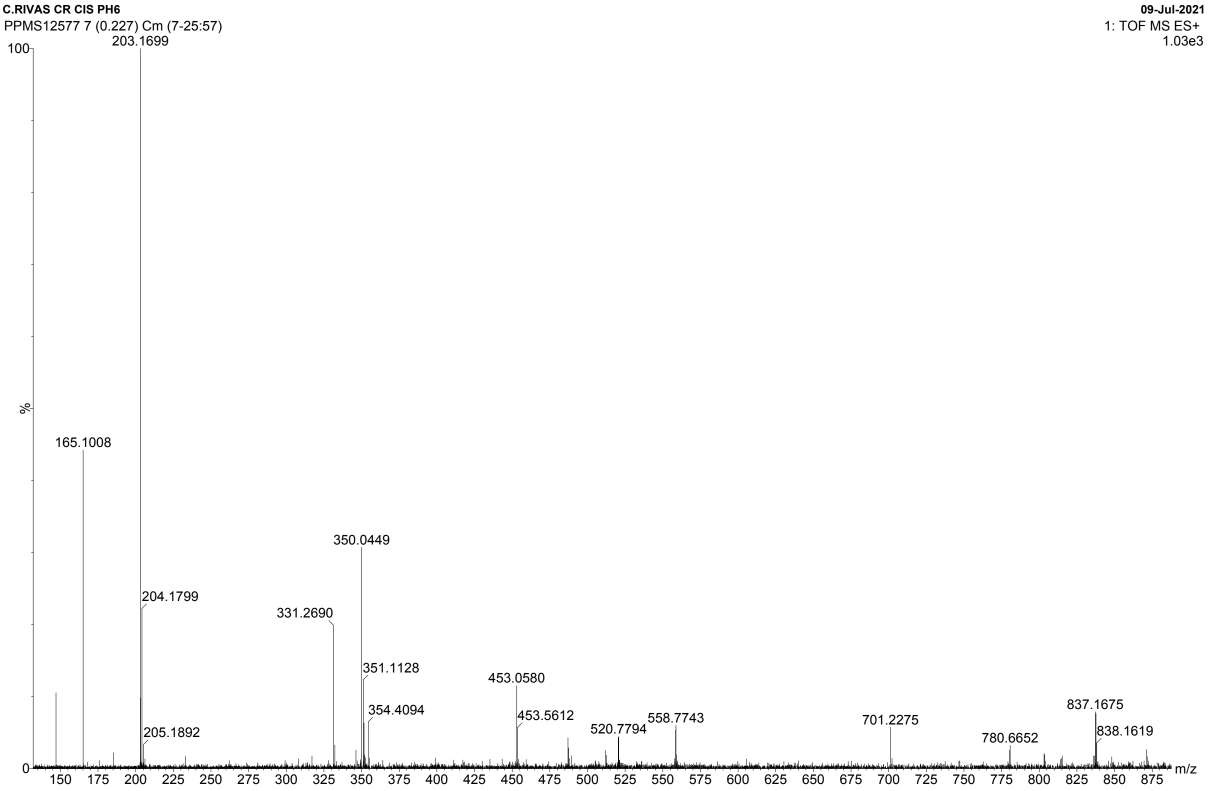

Figure S14. HR-MS of compound ***In-L**[1A]*.

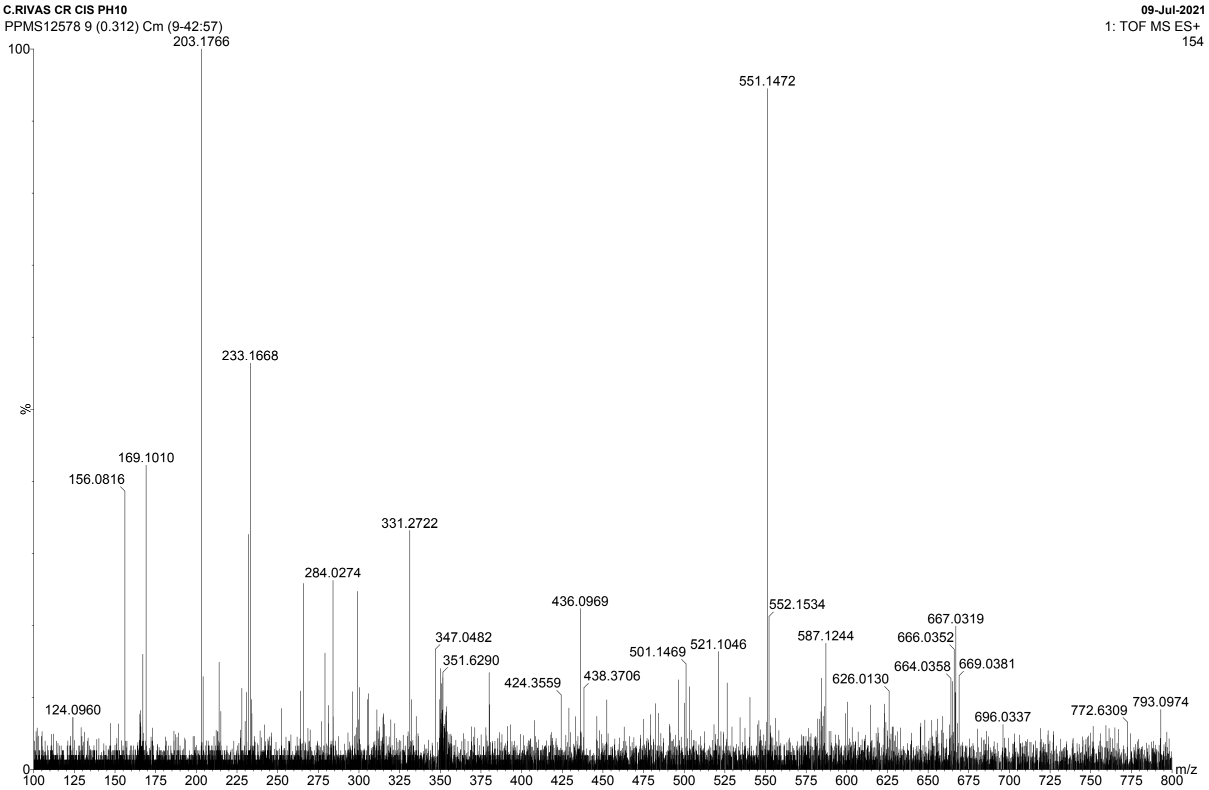

Figure S15. HR-MS of compound ***In-L**[1B]*.

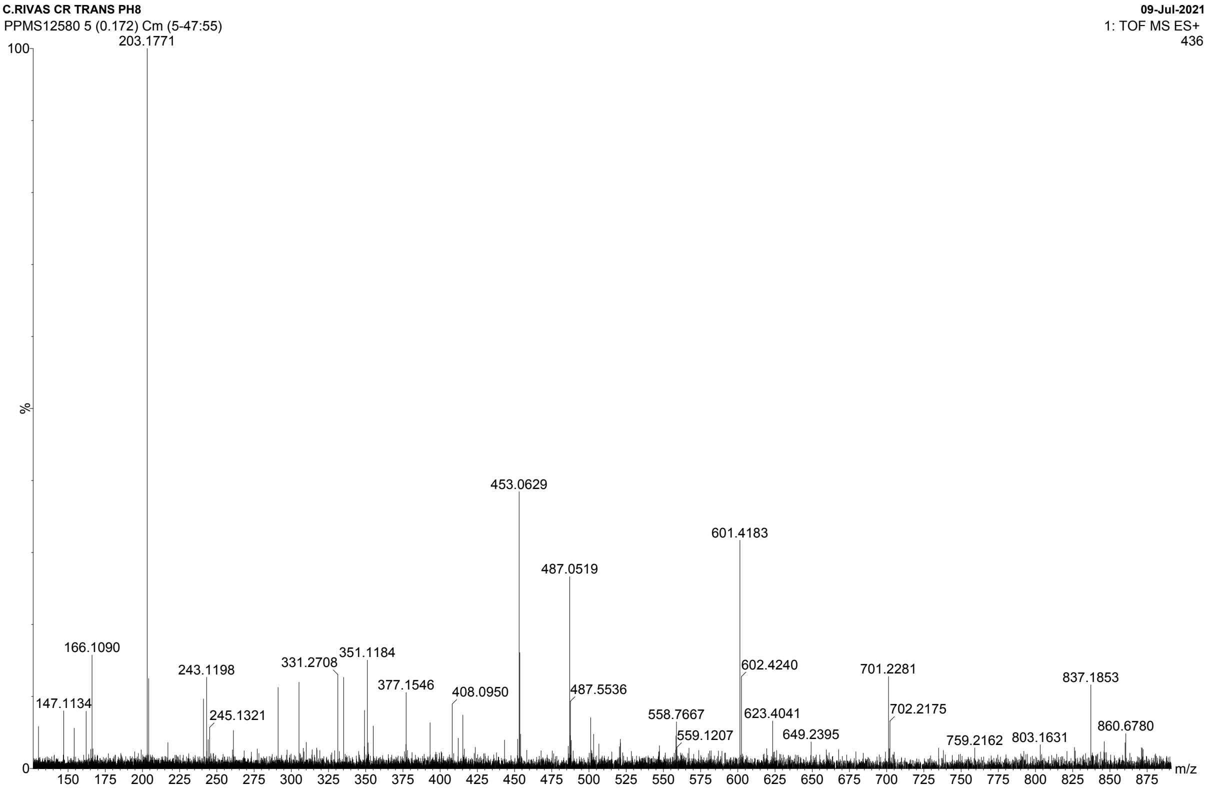

Figure S16. HR-MS of compound ***In-L^2A^***.

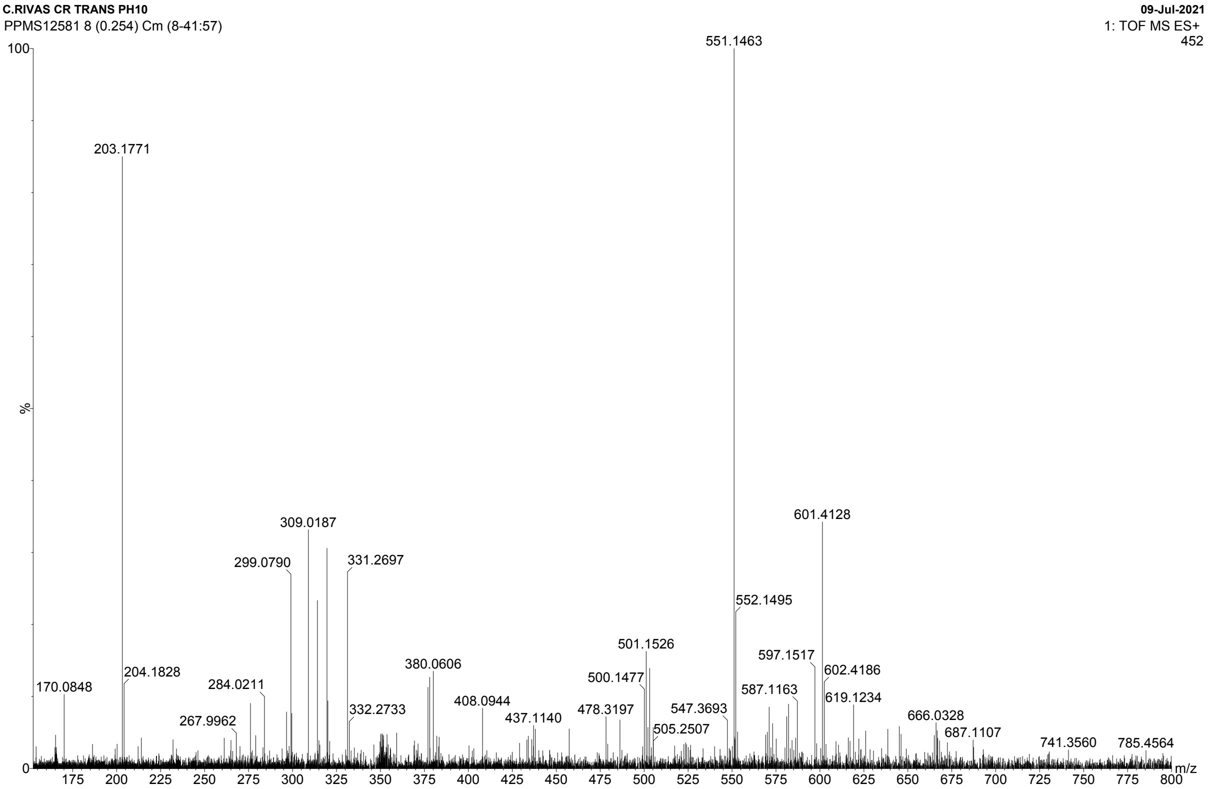

Figure S17. HR-MS of compound ***In-L^2B^***.

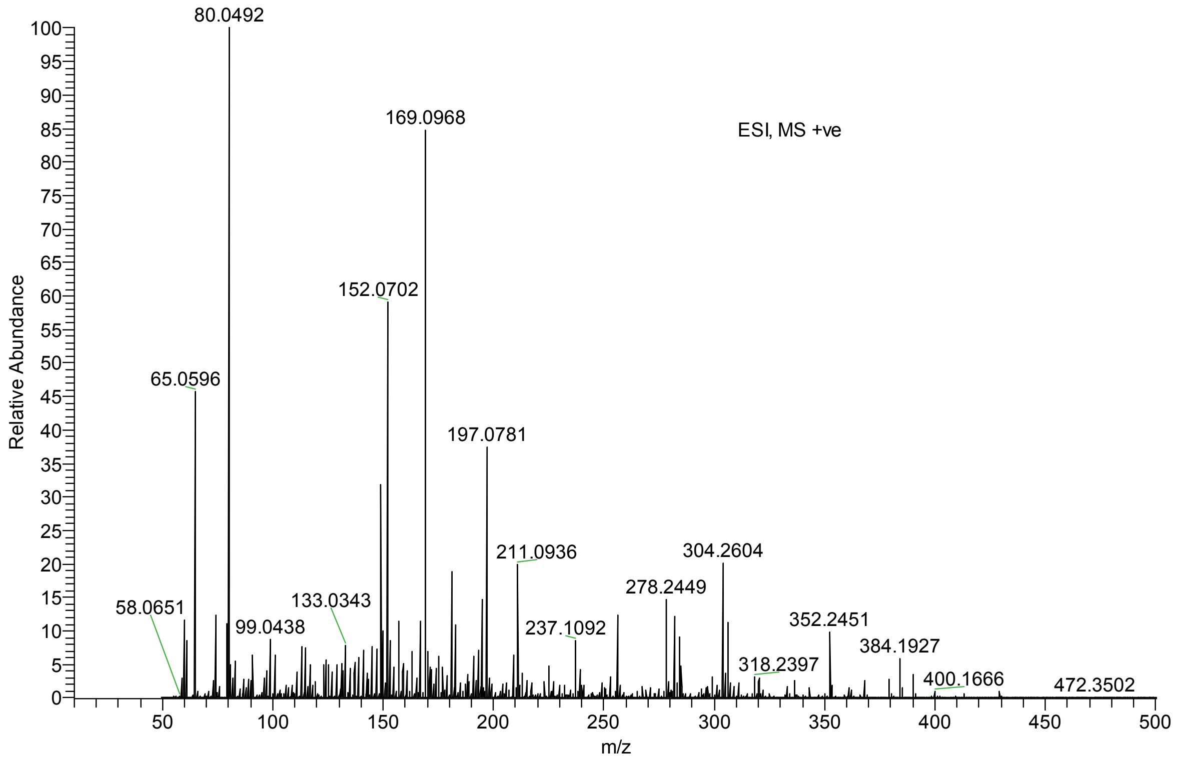

*Figure S18. HR-MS of compound **HOPO-NH₂**: found 169.0968; calc. for [$C_8H_{12}N_2O_2$ + H]$^+$ 169.0972.*

4. X-ray Crystallography

The X-ray crystal structure of 2

Crystal data for **2**: $C_{17}H_{19}ClN_2O_3$, M = 334.79, triclinic, P-1 (no. 2), a = 7.6829(6), b = 9.1538(8), c = 12.4738(14) Å, α = 71.692(9), β = 85.455(8), γ = 71.875(7)°, V = 791.35(14) Å^3, Z = 2, D_c = 1.405 g cm^{-3}, μ(Cu-Kα) = 2.284 mm^{-1}, T = 173 K, colourless blocks, Agilent Xcalibur PX Ultra A diffractometer; 2986 independent measured reflections (R_{int} = 0.0440), F^2 refinement,[1-3] R_1(obs) = 0.0514, wR_2(all) = 0.1401, 2180 independent observed absorption-corrected reflections [$|F_o| > 4\sigma(|F_o|)$, completeness to θ_{full}(67.7°) = 97.4%], 215 parameters. CCDC 2202474.

The N9–H hydrogen atom in the structure of **2** was located from a ΔF map and refined freely subject to an N–H distance constraint of 0.90 Å.

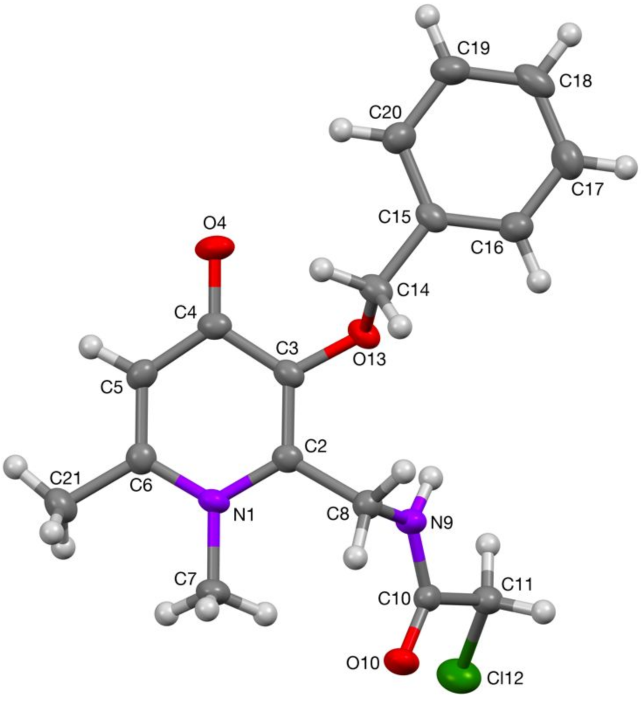

Figure S19. Representation of ***2*** *(50% probability ellipsoids).*

5. Hydrolysis mechanism

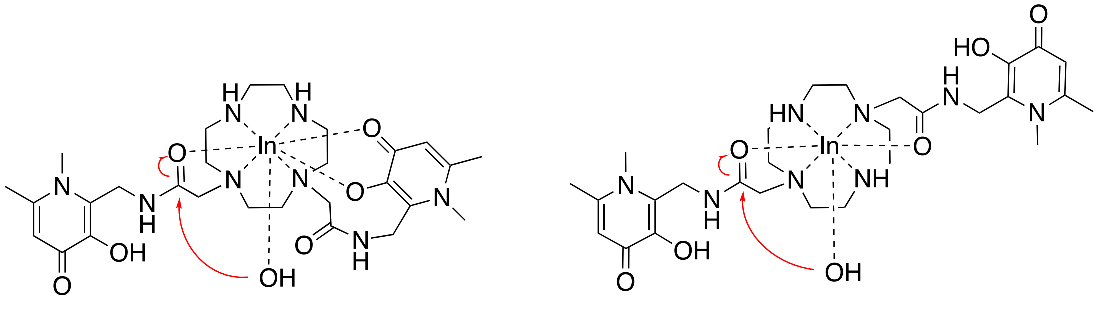

Figure S20. Proposed intramolecular amide hydrolysis mechanism.

6. Gallium Complexation

We elected to further probe the reactivity of **L^1** with Ga^{3+}, which has exceptionally high affinity for hydroxypyridinone ligands. **L^1** was reacted with [^{nat}Ga]$Ga(NO_3)_3$ to form [^{nat}Ga][Ga(**L^1**)]$^+$, and characterised by ^{1}H NMR and MS, which indicated that [Ga(**L^1**)]$^+$ likely consists of a complex in which the hydroxypyridinone groups coordinate Ga^{3+}, and the cyclen ring does not (*Figure S21*). *Bis*(hydroxypyridinone) complexation would furnish an O_4 tetradentate complex, and it is possible that solvent molecules or ions occupy vacant coordination sites on Ga^{3+}. *Importantly, no hydrolysis of [^{nat}Ga][Ga(**L^1**)]$^+$ was observed.* We also reacted **L^1** with $^{68}Ga^{3+}$: [^{68}Ga]Ga^{3+} complexation proceeds at both ambient temperature in 30 s, and also at 90 °C in 40 min, to yield a single product, which co-eluted with non-radioactive [^{nat}Ga][Ga(**L^1**)]$^+$ in HPLC experiments (*Figure S22*). Based on this data, we hypothesise that cyclen coordination to a metal ion is important for hydrolytic activity of this ligand system, although extensive further experiments are required to assess reactivity of these compounds with other metal ions.

[natGa][Ga(**L^1**)]$^+$: A sample of **L^1** (5 mg, 8.5 mmol) was dissolved in deionised water (0.7 ml) and $Ga(NO_3)_3$ (2.2 mg, 9 mmol) was added. The reaction was heated to 80 °C for 1 h. The solution was analysed by reverse-phase HPLC as previously described. HPLC retention time: 13.5 minutes. LR-MS for $[M + H]^{2+}$ (M = $GaC_{28}H_{42}N_8O_6^+$): 328.2 (obs), 328.1 (calc). ^{1}H NMR (400 MHz, D2O) δ 2.39-2.51 (m, 6H, HP-C***H$_3$***), 3.11-3.26 (m, 16H, cyclen-C***H$_2$***), 3.67-3.87 (m, 10H, HP-NC***H$_3$***/NHCOC***H$_2$***), 4.67-4.76 (m, 4H, HP-C***H$_2$***), 6.59-6.77 (m, 2H, HP-***H***).

[^{68}Ga][Ga(**L^1**)]$^+$: An aliquot of **L^1** (100 µl of a 100 µM solution) was combined with a solution of generator-produced [^{68}Ga]Ga^{3+} (100 µl of in 0.1 M aqueous HCl solution, approx. 10 MBq) and ammonium acetate solution (40 µl, 1 M) to give a solution with a final chelator concentrations of 40 µM, respectively, at pH 6. The reaction solutions were incubated at either 25, 40 or 90 °C for 30 s or 40 minutes, prior to reverse-phase HPLC analysis. All reactions were analysed by reverse-phase HPLC to confirm radiolabelling and calculate yields. HPLC retention time: 14.5 mins.

Reverse-phase HPLC analysis: 0 to 25 % B linear gradient 30 min, 25 to 0 % B linear gradient 5 min, A: 0.1 % TFA in H2O, B: 0.1 % TFA in MeCN.

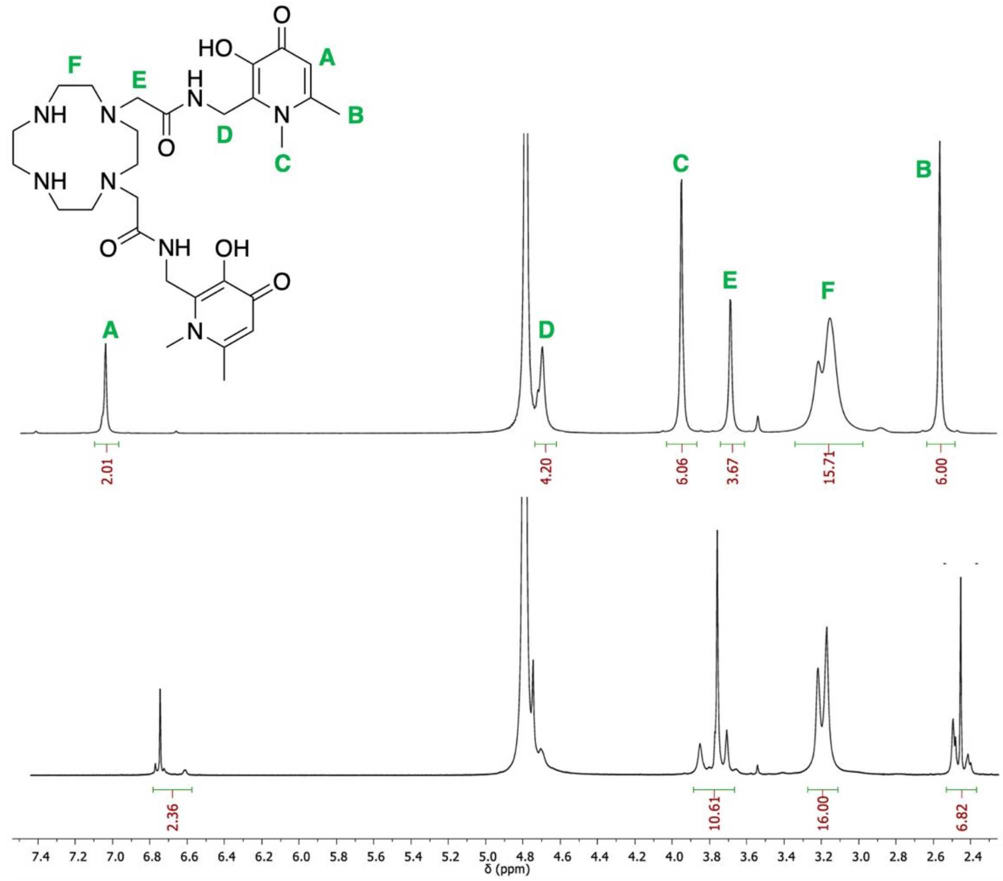

Figure S21. ${}^{1}H$ *NMR spectrum (400 MHz, D_2O, 298 K) of* ***L*[1]** *(top) and* $[Ga(\mathbf{L^1})]^+$ *(bottom).*

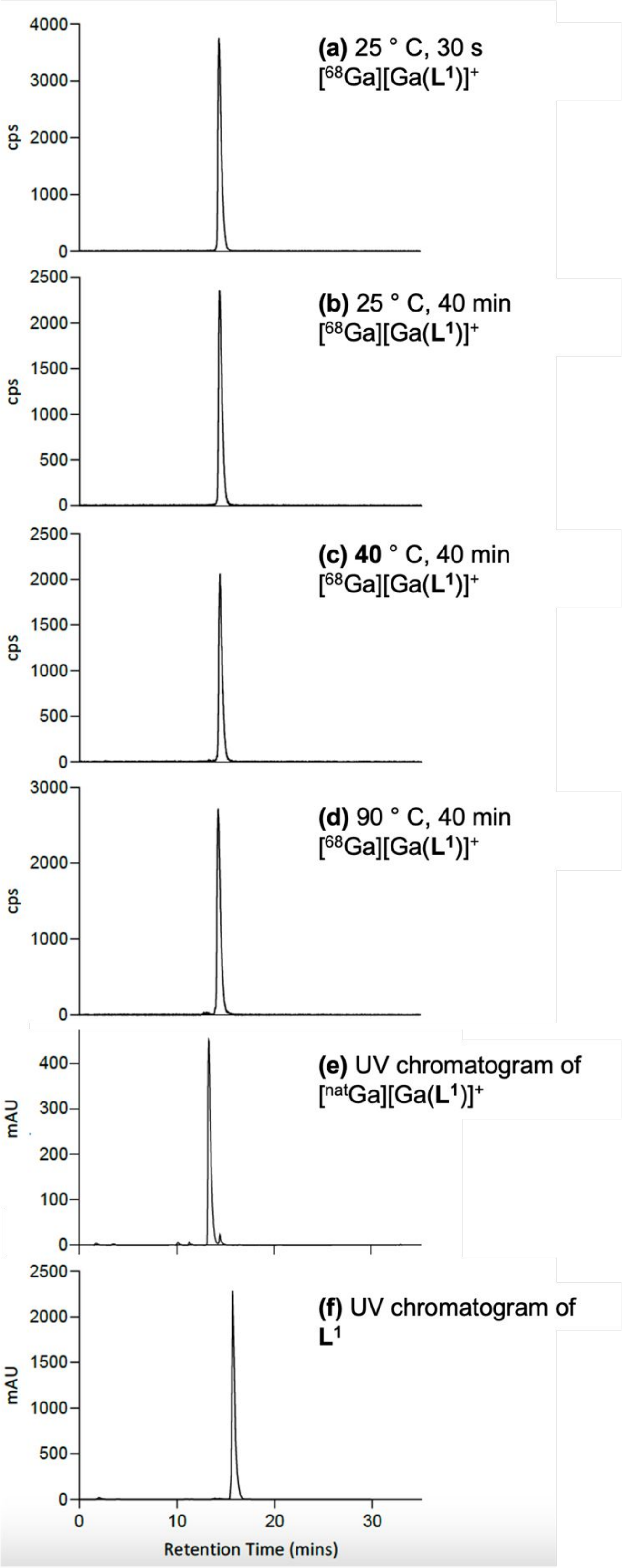

Figure S22. Reverse-phase HPLC chromatograms of ***(a-d)*** *[^{68}Ga][Ga(**L**^{1})]$^{+}$,* ***(e)*** *[^{nat}Ga][Ga(**L**^{1})]$^{+}$ and* ***(f) L^{1}****.*

7. Kinetic analysis

We have *qualitatively* shown that both In^{3+} coordination and In^{3+}-mediated hydrolysis is dependent on the concentration of OH^-. The kinetics of these consecutive reactions are complicated: the rate laws are likely of a high order, complexation will be dependent on [OH^-], [In^{3+}], [**L**^{1}] or [**L**^{2}] and possibly solvent ions (which will influence speciation of simple, precursor In^{3+} complexes in solution), and hydrolysis is likely dependent on [OH^-] and [In-**L**^{1A}] or [In-**L**^{2A}]. In combination, these factors mean that determining experimental rate constants and activation energies for each process is difficult.

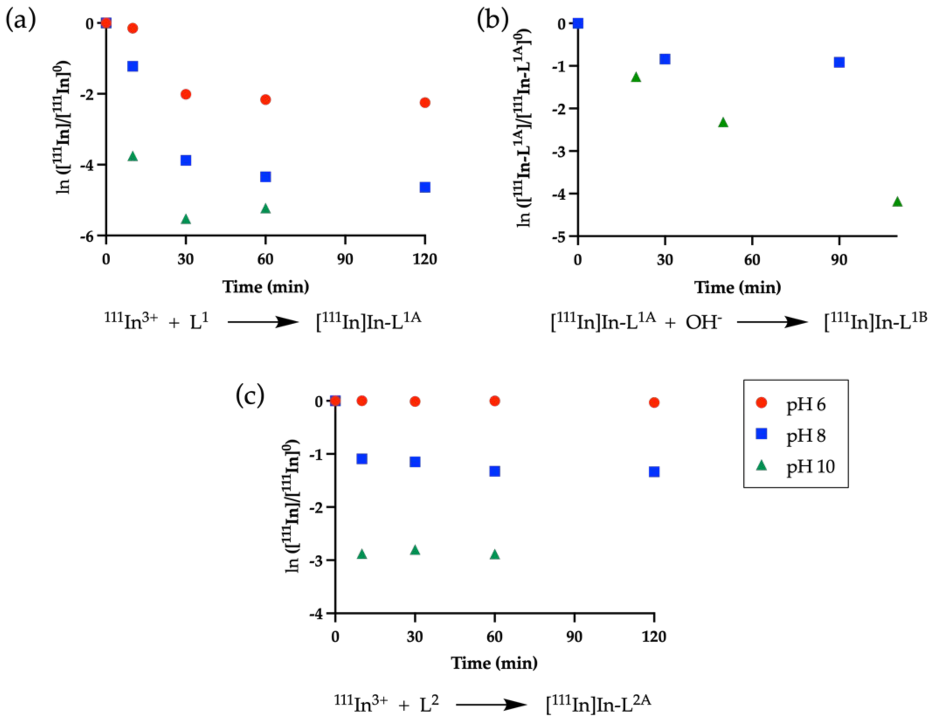

*Figure S23. Graphs of **ln(A/A^0) vs time** for **(a)** complexation of $^{111}In^{3+}$ by **L^1**; **(b)** Hydrolysis of [^{111}In]In-**L^{1A}**; **(c)** Complexation of $^{111}In^{3+}$ by **L^2**.*

However, in radiochemical reactions, due to the large excess of ligand, OH^- and solvent ions relative to $^{111}In^{3+}$, pseudo-first order reactions with respect to $^{111}In^{3+}$ can be approximated.

Using radiochemical HPLC data, we have plotted $\ln(A/A^0)$ versus time (*Figure X*) for the following reactions:

- **Complexation of $^{111}In^{3+}$ by L^1**: plots of $\underline{\ln([^{111}In]/[^{111}In]^0)}$ <u>versus time</u> are not linear, indicative of the intricacy of the kinetic parameters of this reaction.
- **Hydrolysis of $[^{111}In]In\text{-}L^{1A}$** (using radio-HPLC data at timepoints where the amount of unchelated $^{111}In^{3+}$ is less than 5%/negligible): at pH 10, a plot of $\underline{\ln(\ln([^{111}In]In\text{-}L^{1A}/[^{111}In]In\text{-}L^{1A})}$ <u>versus time</u> is linear. Under these experimental conditions, hydrolysis is a first order process; we hypothesise that it is an intramolecular process. However, at pH 8, a plot of $\underline{\ln(\ln([^{111}In]In\text{-}L^{1A}/[^{111}In]In\text{-}L^{1A})}$ <u>versus time</u> is non-linear.
- **Complexation of $^{111}In^{3+}$ by L^2**: plots of $\underline{\ln([^{111}In]/[^{111}In]^0)}$ <u>versus time</u> indicate little change over time: this system likely either rapidly reaches equilibrium under these conditions or the kinetics of complexation are extremely slow.

Importantly, overall, this data is consistent with qualitative observations that both In^{3+} coordination and In^{3+}-mediated hydrolysis is dependent on the concentration of OH^-.

8. Computational Modelling

General details

DFT calculations were run using Gaussian 09 (Revision D.01)[4] and Gaussian 16[5] using the ωB97X-D hybrid exchange-correlation functional, which includes D2 dispersion corrections described by Grimme.[6,7] A functional screening was undertaken, and all structures including transition states were re-optimised using ωB97X[6] and PBE0[8,9] with and without Grimme's D3 dispersion correction including Becke-Johnson damping (GD3BJ).[10,11] This allowed trends to be compared across two functionals, with and without additional dispersion corrections.

NBO analysis was performed using NBO 6.0.[12]

Geometry optimisations were performed without symmetry constraints (nosymm), and all intermediates and transition states were characterised by normal coordinate analysis revealing either precisely zero or one imaginary frequency, respectively. In the case of transition states, the imaginary frequency corresponds to the mode of the intended reaction step. The default numerical integration grid was improved using a pruned grid with 99 radial shells and 590 angular points per shell (int=ultrafine). All structures are optimised using the self-consistent reaction field (SCRF) approach with conductor like polarisable continuum model (CPCM).[13] The CPCM model was implemented using the built-in parameters for H_2O (solvent=water).

Different basis sets (BS1, BS2 and BS3) were used as detailed. The coordination environment of $\mathbf{L^{1B}}$, $\mathbf{L^{2B}}$, $\mathbf{L^{1A}}$ and $\mathbf{L^{2A}}$ with In^{3+} was explored starting from various starting geometries using BS1. Geometry optimisations on each stable conformation of In-**L** (and additionally each associated transition state for the hydrolysis step) with four functionals were performed using

BS2. NBO calculations were performed at this level. Higher level single point calculations were performed on each ωB97X-D optimised structure using the larger basis set, BS3.

BS1 was built as follows. The SDD basis set and corresponding effective core potential was used for In. The split-valence 6-31(d) basis set was used for C and H atoms. The triple-ξ 6-311+G(d) basis set was used for N and O.

BS2 was built as follows. The SDD basis set and corresponding effective core potential was used for In. The split-valence 6-31+G(d,p) basis set was used for C and H atoms. The triple-ξ 6-311+G(d) basis set was used for N and O.

BS3 was built as follows. The SDD basis set and corresponding effective core potential was used for In. All other elements (C, H, N and O) were described using Ahlrichs quadruple-ξ basis set def2-QZVPP.[14]

Full coordinates for all the calculated stationary points are included as part of the supplementary information (.xyz).

For simplicity, the coordination environment of $\mathbf{L^{1B}}$ with In^{3+} was chosen as a starting point. These structures were modelled using the ωB97X functional and BS1 with no solvent corrections. The input geometries were pre-selected based on the possible coordination groups of each ligand *i.e.* each carboxylic acid on one arm, and amide or hydroxypyridinone (hopo) on the other. Due to the basic conditions of the reaction being modelled, both the carboxylic acid and hopo moieties were deprotonated. This led to five possible permutations. All five permutations were explored, with and without an additional bound hydroxide to In (Tables S1 and S2). Note that several structures were calculated where the amide proton had migrated to the hopo–O^-, or where the exposed hopo–O^- was bound to the electrophilic amide carbon.

Input Geometry		Output Geometry		G (hartrees)	ΔG (kcal mol^{-1})
Arm 1	*Arm 2*	*Arm 1*	*Arm 2*		
$In(OH)_3$				-229.421404	
OH^-				-75.79776	
H_2O				-76.413949	
$\mathbf{L^{1B}}$				-1486.566605	
carboxylate	–	carboxylate	amide*	-1487.174838	45.48
–	amide	carboxylate	amide*	-1487.175584	45.01
–	hopo	carboxylate	hopo	-1487.247250	0.04
carboxylate	amide	carboxylate	amide**	-1487.201173	28.95
carboxylate	hopo	carboxylate	hopo	-1487.247312	0.00

* amide proton migrated to hopo–O$^-$
**hopo–O– bound to amide carbon forming six-membered ring

Table S1. Coordination environment of ***L^{1B}*** *with In^{3+} (unbound hydroxide) showing input and output geometries, and calculated absolute and relative free energies.*

Input Geometry		Output Geometry		G (hartrees)	ΔG (kcal mol^{-1})
Arm 1	*Arm 2*	*Arm 1*	*Arm 2*		
carboxylate	–	carboxylate	amide*	-1563.222473	2.46
–	amide	carboxylate	amide*	-1563.223113	2.06
–	hopo	carboxylate	hopo	-1563.226394	0.00
carboxylate	amide	carboxylate	amide	-1563.214494	7.47
carboxylate	hopo	not found		–	–

* amide proton migrated to hopo–O$^-$

Table S2. Coordination environment of ***L^{1B}*** *with In^{3+} (bound hydroxide) showing input and output geometries, and calculated absolute and relative free energies.*

For In-**L^{1B}**, in each case the output geometry showed the carboxylate arm was coordinated, and stationary points were found with either amide or hopo coordination from the other arm. In all cases, coordination of hydroxide was favoured over a cationic complex by > 100 kcal mol^{-1} *i.e.* [In(**L^{1B}**)(OH)] favoured over [In(**L^{1B}**)]$^+$[OH]$^-$ (Table S3). In both cases, the hopo-carb coordination geometry was favoured at BS1.

Structure	G (hartrees)	ΔG (kcal mol^{-1})
[In(**L^1B**)]$^+$[OH]$^-$ (hopo-carb)	-1563.045072	113.78
[In(**L^1B**)]$^+$[OH]$^-$ (amide-carb)	-1562.973344	158.79
[In(**L^1B**)(OH)] (hopo-carb)	-1563.226394	0.00
[In(**L^1B**)(OH)] (amide-carb)	-1563.214494	7.47

Table S3. Comparison of [In($\mathbf{L^{1B}}$)(OH)] and [In($\mathbf{L^{1B}}$)]$^+$[OH]$^-$

Subsequently, corresponding geometries for [In($\mathbf{L^{2B}}$)(OH)] and [In($\mathbf{L^{2B}}$)]$^+$[OH]$^-$ were found at this level of theory (Table S4). Similar to $\mathbf{L^{1B}}$, hydroxide coordination and the hopo-carb geometry is favoured.

Structure	G (hartrees)	ΔG (kcal mol^{-1})
[In($\mathbf{L^{2B}}$)]$^+$[OH]$^-$ (hopo-carb)	-1563.047513	130.78
[In($\mathbf{L^{2B}}$)]$^+$[OH]$^-$ (amide-carb)	-1563.046399	131.48
[In($\mathbf{L^{2B}}$)(OH)] (hopo-carb)	-1563.255920	0.00
[In($\mathbf{L^{2B}}$)(OH)] (amide-carb)	-1563.214950	25.71

Table S4. Comparison of [In($\mathbf{L^{2B}}$)(OH)] and [In($\mathbf{L^{2B}}$)]$^+$[OH]$^-$

Exploring the coordination geometries of $\mathbf{L^{B}}$ with In^{3+} allows the following conditions to be applied for understanding the coordination of $\mathbf{L^{A}}$:

- The complex likely has a bound hydroxide and takes the form [In($\mathbf{L^{A}}$)(OH)]
- Either hopo or amide moieties on each arm can coordinate, leading to three possible coordination modes: hopo-hopo, hopo-amide, amide-amide

Consequently, a complete energy surface for the hydrolysis of each ligand was calculated using BS2 across four different functionals (ωB97X, ωB97X-D, PBE0, PBE0-GD3BJ). The energy surface consists of i) initial coordination of **L** to $In(OH)_3$ forming three possible conformers (**I** = hopo-hopo; **II** = hopo-amide; **III** = amide-amide) with liberation of 2 H_2O molecules, ii) hydrolysis of a/the bound amide moiety via a transition state, with each conformer having an

associated barrier, and iii) formation of the hydrolyzed complex **IV** (*i.e.* In-**L**$^{\mathbf{B}}$) with liberation of a cleaved hydroxypyridinone moiety terminated with an amine (**HOPO**). The data for each stationary point with each functional are tabulated in Tables S5-8, and energy level diagrams are presented as Figures S20–S23.

**Structure**	**G (hartrees)**	**ΔG (kcal mol^{-1})**	**ΔΔG (kcal mol^{-1})**
L^{1}			
**L**^{1}	-1982.078657		
H_2O	-76.437450		
$In(OH)_3$	-229.464062		
**I** (hopo-hopo)	-2058.721244	-33.52	
**II** (hopo-amide)	-2058.721277	-33.54	
**III** (amide-amide)	-2058.733787	-41.39	
TS (**I-IV**)	-2058.690184	-14.03	19.49
TS (**II-IV**)	-2058.706742	-24.42	9.12
TS (**III-IV**)	-2058.687070	-12.08	29.31
**IV** (hopo-carb)	-1563.348355	-49.35	
**IV** (amide-carb)	-1563.367393	-61.30	
**HOPO**	-571.835558		
L^{2}			
**L**^{2}	-1982.080275		
**Ia** (hopo-hopo)	-2058.721643	-32.76	
**Ib** (hopo-hopo)	-2058.738497	-43.34	
**II** (hopo-amide)	-2058.736816	-42.28	
**III** (amide-amide)	-2058.732859	-39.80	
TS (**Ib-IV**)	-2058.693762	-15.26	28.07
TS (**II-IV**)	-2058.695510	-16.36	25.92
TS (**III-IV**)	-2058.694908	-15.98	23.81
**IV** (hopo-carb)	-1563.376959	-66.28	
**IV** (amide-carb)	-1563.363567	-57.88	

Table S5. Calculated free energies for the coordination and hydrolysis of ***L***$^{\mathbf{1}}$ *and* ***L***$^{\mathbf{2}}$ *(ωB97X)*

Structure	G (hartrees)	ΔG (kcal mol^{-1})	ΔΔG (kcal mol^{-1})
L^1			
**L^1**	-1981.961978		
H_2O	-76.435381		
$In(OH)_3$	-229.455021		
**I** (hopo-hopo)	-2058.606582	-37.87	
**II** (hopo-amide)	-2058.612669	-41.69	
**III** (amide-amide)	-2058.621913	-47.49	
TS (**I-IV**)	-2058.581516	-22.14	15.73
TS (**II-IV**)	-2058.588746	-26.67	15.01
TS (**III-IV**)	-2058.578449	-20.21	27.27
**IV** (hopo-carb)	-1563.266574	-51.87	
**IV** (amide-carb)	-1563.282030	-61.57	
**HOPO**	-571.797712		
L^2			
**L^2**	-1981.962630		
**Ia** (hopo-hopo)	-2058.609128	-39.05	
**Ib** (hopo-hopo)	-2058.624037	-48.41	
**II** (hopo-amide)	-2058.624312	-48.58	
**III** (amide-amide)	-2058.616234	-43.51	
TS (**Ib-IV**)	-2058.591342	-27.89	20.52
TS (**II-IV**)	-2058.584627	-23.68	24.90
TS (**III-IV**)	-2058.584594	-23.66	19.85
**IV** (hopo-carb)	-1563.296563	-70.28	
**IV** (amide-carb)	-1563.280750	-60.36	

*Table S6. Calculated free energies for the coordination and hydrolysis of **L^1** and **L^2** (ωB97X-D)*

Structure	G (hartrees)	ΔG (kcal mol^{-1})	ΔΔG (kcal mol^{-1})
L¹			
**L¹**	-1980.293429		
H_2O	-76.374813		
$In(OH)_3$	-229.274463		
**I** (hopo-hopo)	-2056.861900	-27.38	
**II** (hopo-amide)	-2056.873667	-34.76	
**III** (amide-amide)	-2056.893241	-47.05	
TS (**I-IV**)	-2056.839328	-13.22	14.16
TS (**II-IV**)	-2056.861721	-27.27	7.50
TS (**III-IV**)	-2056.853677	-22.22	24.83
**IV** (hopo-carb)	-1561.941623	-49.56	
**IV** (amide-carb)	-1561.970898	-67.93	
**HOPO**	-571.330430		
L²			
**L²**	-1980.293045		
**Ia** (hopo-hopo)			
**Ib** (hopo-hopo)	-2056.888544	-44.34	
**II** (hopo-amide)	-2056.889933	-45.21	
**III** (amide-amide)	-2056.892435	-46.78	
TS (**Ib-IV**)			
TS (**II-IV**)	-2056.851764	-21.26	23.95
TS (**III-IV**)	-2056.856582	-24.28	22.50
**IV** (hopo-carb)	-1561.972134	-68.94	
**IV** (amide-carb)	-1561.970053	-67.64	

*Table S7. Calculated free energies for the coordination and hydrolysis of **L¹** and **L²** (PBE0)*

Structure	G (hartrees)	ΔG (kcal mol^{-1})	ΔΔG (kcal mol^{-1})
L^1			
**L^1**	-1980.404754		
H_2O	-76.375090		
$In(OH)_3$	-229.277530		
**I** (hopo-hopo)	-2056.991872	-37.50	
**II** (hopo-amide)	-2057.003733	-44.95	
**III** (amide-amide)	-2057.011683	-49.94	
TS (**I-IV**)	-2056.972244	-25.19	12.32
TS (**II-IV**)	-2056.988346	-35.29	9.66
TS (**III-IV**)	-2056.973046	-25.69	24.24
**IV** (hopo-carb)	-1562.039568	-53.82	
**IV** (amide-carb)	-1562.055944	-64.10	
**HOPO**	-571.353400		
L^2			
**L^2**	-1980.401929		
**Ia** (hopo-hopo)			
**Ib** (hopo-hopo)	-2057.011062	-51.32	
**II** (hopo-amide)	-2057.014454	-53.45	
**III** (amide-amide)	-2057.009918	-50.60	
TS (**Ib-IV**)	-2056.977163	-30.05	21.27
TS (**II-IV**)	-2056.978146	-30.66	22.78
TS (**III-IV**)	-2056.977163	-30.05	20.55
**IV** (hopo-carb)	-1562.068396	-73.69	
**IV** (amide-carb)	-1562.057418	-66.80	

*Table S8. Calculated free energies for the coordination and hydrolysis of **L^1** and **L^2** (PBE0-GD3BJ)*

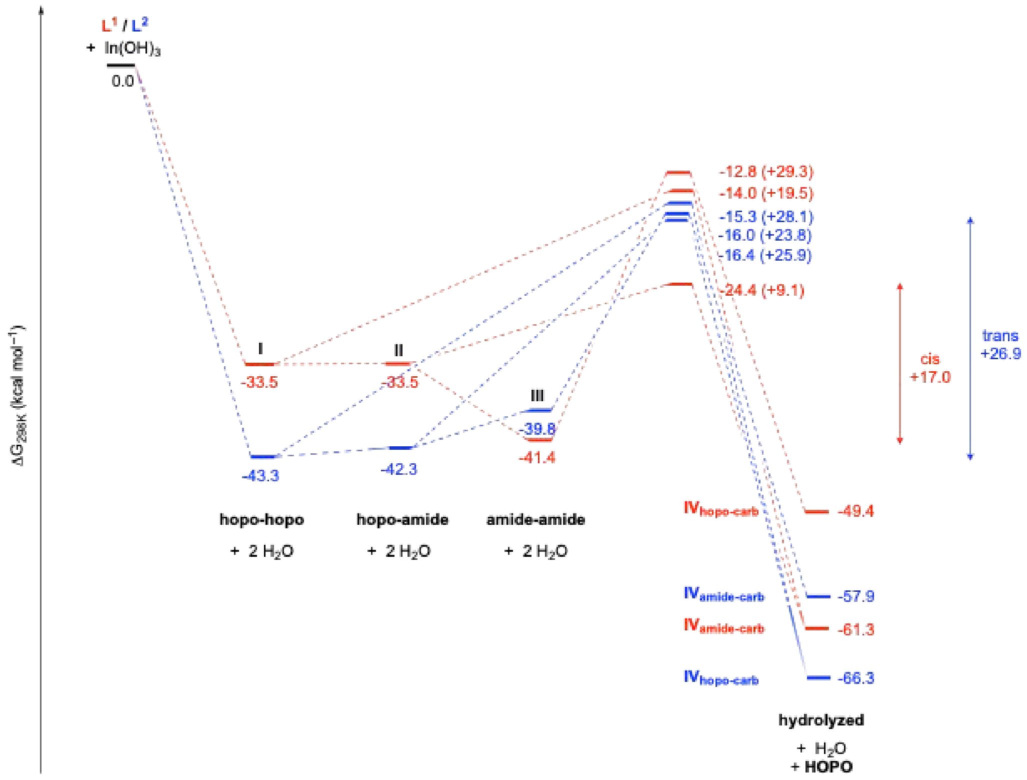

*Figure S24. Energy level diagram for the coordination and hydrolysis of **L¹** and **L²** (ωB97X)*

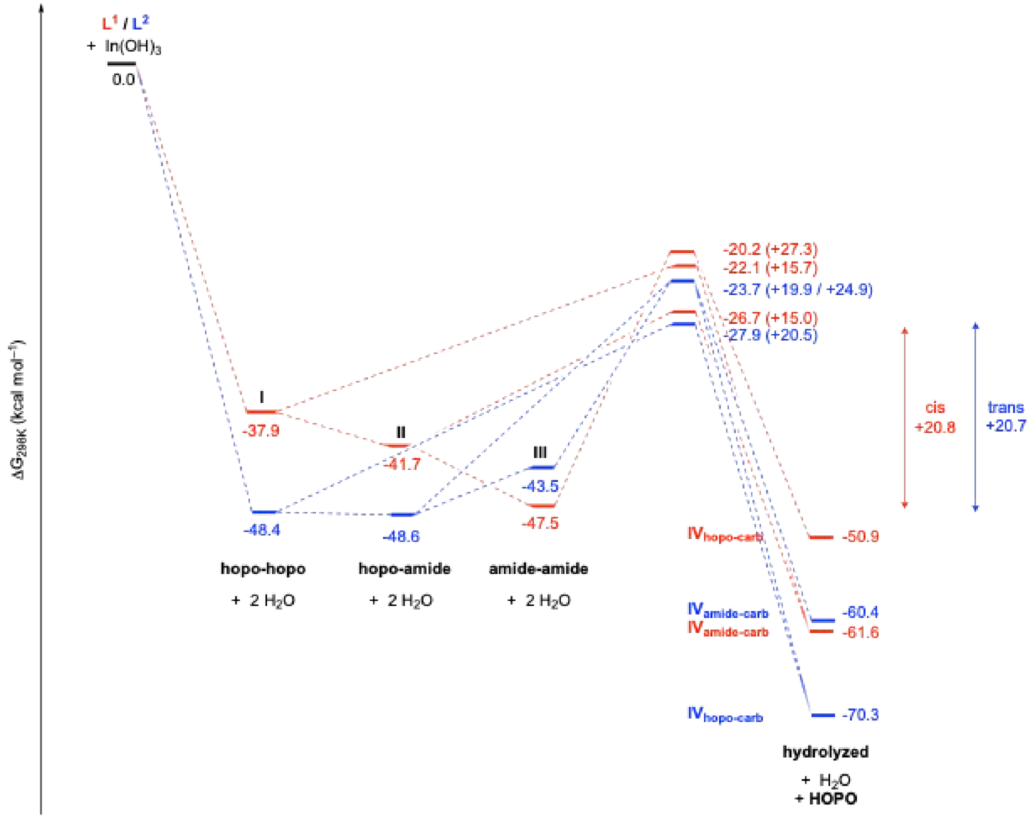

*Figure S25. Energy level diagram for the coordination and hydrolysis of **L¹** and **L²** (ωB97X-D)*

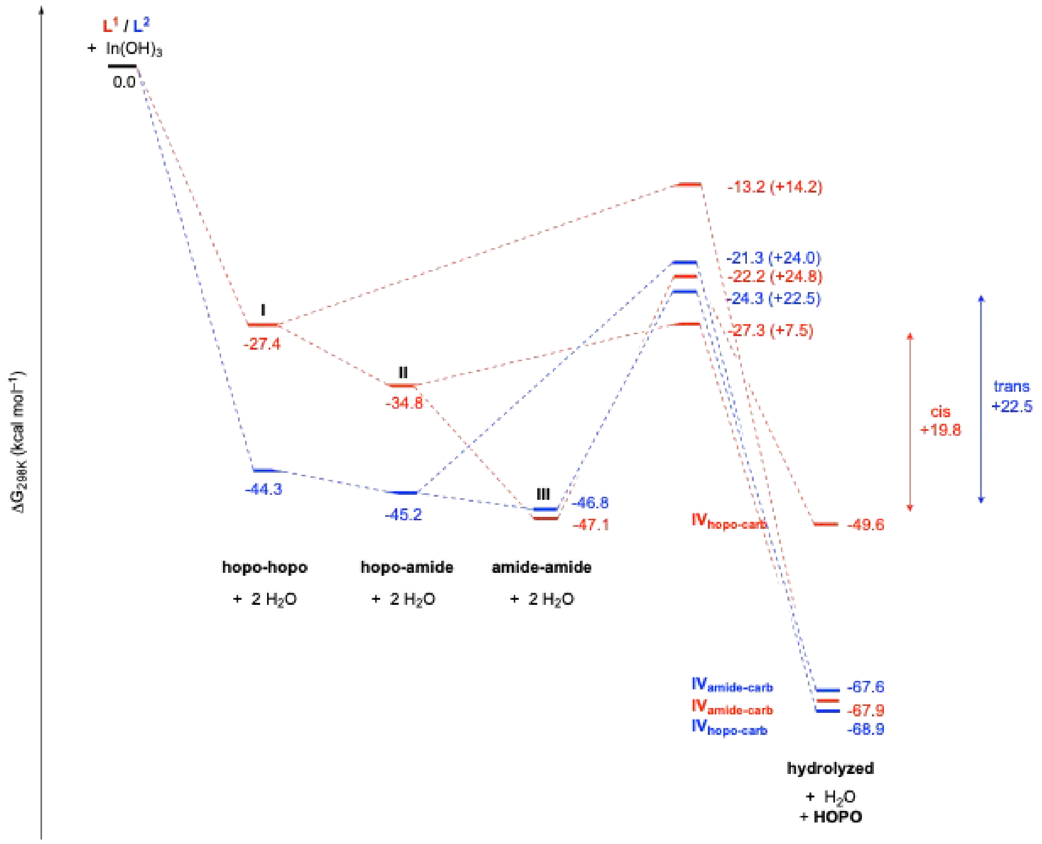

Figure S26. Energy level diagram for the coordination and hydrolysis of ***L¹*** *and* ***L²*** *(PBE0)*

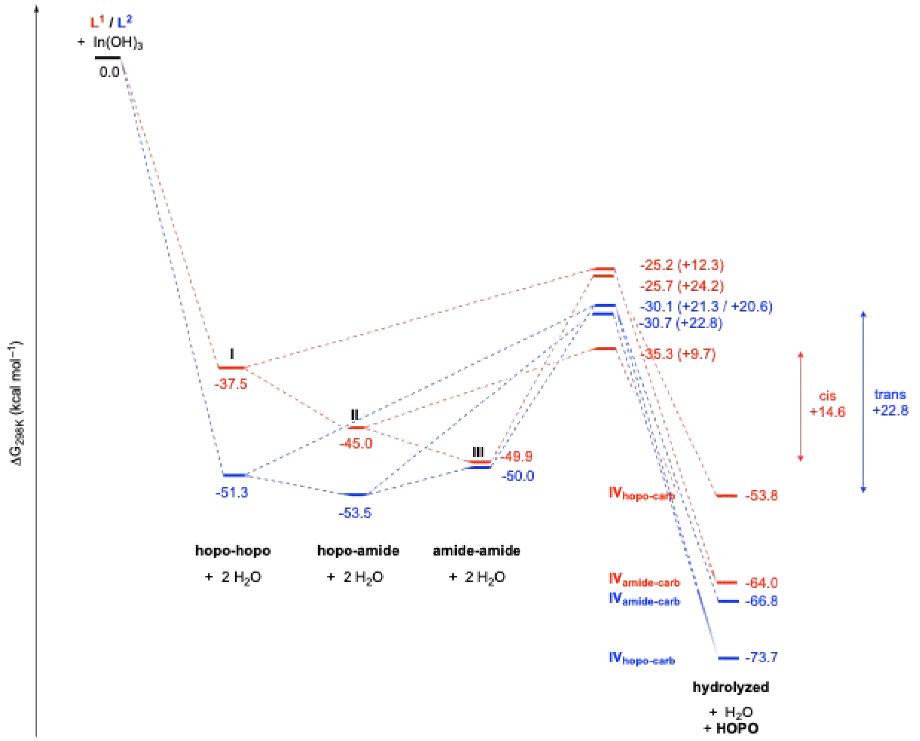

Figure S27. Energy level diagram for the coordination and hydrolysis of ***L¹*** *and* ***L²*** *(PBE0-GD3BJ)*

Finally, each optimised structure at the ωB97X-D/BS2 level was single point corrected at BS3.

The tabulated data for the correction are presented in Table S9.

**Structure**	**E^{elec}(BS2)**	**G_{sol}(BS2) corr.**	**G_{sol}(BS2)**	**E^{elec}(BS3)**	**E^{elec}(BS3) + [G_{sol}(BS2)-E^{elec}(BS2)]**	**ΔG (kcal mol^{-1})**	**ΔΔG (kcal mol^{-1})**
L^1							
**L^1**	-1982.637308	0.675330	-1981.961978	-1983.162126	-1244010.4645		
H_2O	-76.4387237	0.003342	-76.435381	-76.45375678	-47972.6348		
$In(OH)_3$	-229.4622283	0.007207	-229.455021	-417.7830266	-262154.3267		
I (hopo-hopo)	-2059.278337	0.671755	-2058.606582	-2248.070538	-1410242.7367	-23.22	
II (hopo-amide)	-2059.281595	0.668926	-2058.612669	-2248.073960	-1410246.6593	-27.14	
III (amide-amide)	-2059.286394	0.664481	-2058.621913	-2248.077012	-1410251.3631	-31.84	
TS (I-IV)	-2059.253679	0.672163	-2058.581516	-2248.041554	-1410224.2933	-4.77	18.44
TS (II-IV)	-2059.258992	0.670246	-2058.588746	-2248.048147	-1410229.6331	-10.11	17.03
TS (III-IV)	-2059.248830	0.670381	-2058.578449	-2248.038316	-1410223.3791	-3.86	27.98
IV (hopo-carb)	-1563.770014	0.503440	-1563.266574	-1752.426248	-1099331.5619	-39.65	
IV (amide-carb)	-1563.782792	0.500763	-1563.282030	-1752.436533	-1099339.6959	-47.78	
**HOPO**	-571.9634579	0.165746	-572.1183199	-572.1183199	-358900.2401		
L^2							
**L^2**	-1982.635285	0.672655	-1981.96263	-1983.160569	-1244011.1662		
**I** (hopo-hopo)	-2059.295438	0.671401	-2058.624037	-2248.085622	-1410252.4235	-32.20	
**II** (hopo-amide)	-2059.294345	0.670032	-2058.624312	-2248.085764	-1410253.3714	-33.15	
**III** (amide-amide)	-2059.285209	0.668975	-2058.616234	-2248.074661	-1410247.0681	-26.84	
TS (**Ib-IV**)	-2059.265561	0.674219	-2058.591342	-2248.051072	-1410228.9751	-8.75	23.45
TS (**II-IV**)	-2059.254735	0.670108	-2058.584627	-2248.041002	-1410225.2362	-5.01	28.14
TS (**III-IV**)	-2059.253659	0.669065	-2058.584594	-2248.039755	-1410225.1078	-4.88	21.96
**IV** (hopo-carb)	-1563.798498	0.501935	-1563.296563	-1752.447591	-1099345.8988	-53.28	
**IV** (amide-carb)	-1563.782996	0.502246	-1563.28075	-1752.435663	-1099338.2195	-45.60	

Table S9. Single point corrections for optimised structures at the ωB97X-D/BS2 level using BS3.

Coordinates

pbe-gd3bj_H2O.log

SCF (wB97x) = -76.378281413
E(SCF)+ZPE(0 K)= -76.356800
H(298 K)= -76.353020
G(298 K)= -76.375090
Lowest Frequency = 1606.6740cm-1

O	-0.210168	1.497761	0.000000
H	0.748979	1.534217	0.000000
H	-0.495978	2.414067	0.000000

pbe-gd3bj_HOPO.log

SCF (wB97x) = -571.518066605
E(SCF)+ZPE(0 K)= -571.316342
H(298 K)= -571.303582
G(298 K)= -571.353400
Lowest Frequency = 80.4669cm-1

C	-2.173062	-2.336541	-0.122303
C	-0.811247	-2.242815	-0.197869
C	-0.112337	-0.978874	-0.134647
C	-0.952821	0.154202	0.007849
C	-2.324941	0.038345	0.091680
H	-0.506496	1.141838	0.049552
N	-2.920175	-1.185220	0.046784
C	-4.377696	-1.303782	0.105565
H	-4.631801	-2.234542	0.611285
H	-4.790478	-0.480455	0.682872
H	-4.805857	-1.285508	-0.901394
C	-3.181824	1.258914	0.227377
H	-2.553027	2.147183	0.159251
H	-3.938764	1.314345	-0.560875
H	-3.699284	1.287850	1.192375
C	-2.856882	-3.671244	-0.205768
H	-2.138263	-4.372924	-0.644411
H	-3.704119	-3.612506	-0.897214
O	-0.021180	-3.326547	-0.344810
H	0.878356	-2.945106	-0.373227
O	1.143894	-0.989431	-0.214827
N	-3.359846	-4.091869	1.103267
H	-3.856992	-4.970761	1.012588
H	-2.583506	-4.258598	1.733863

pbe-gd3bj_InOH3.log

SCF (wB97x) = -229.284431929
E(SCF)+ZPE(0 K)= -229.245848
H(298 K)= -229.237772
G(298 K)= -229.277530
Lowest Frequency = 123.8612cm-1

In	0.745296	-0.634778	0.063509
O	0.065217	1.113708	-0.461876
H	-0.878755	1.187447	-0.620632
O	2.649502	-0.881394	0.392144
H	3.216650	-0.115448	0.276315
O	-0.483270	-2.133818	0.261526
H	-0.108298	-2.978705	0.520985

pbe-gd3bj_L1_III.log

SCF (wB97x) = -205767277497
E(SCF)+ZPE(0 K)= -2056.936054
H(298 K)= -2056.892157
G(298 K)= -2057.011683
Lowest Frequency = 11.5559cm-1

O	-1.920967	2.968222	5.662797
N	-0.360752	2.267592	3.554788
C	-2.668692	1.856176	2.749306
H	-2.987576	2.835134	3.114825
H	-3.258611	1.642031	1.850544
C	-1.193702	1.892453	2.395441
H	-0.860337	0.917804	2.031453
H	-1.040135	2.601612	1.571480
C	1.061623	1.935854	3.317385
H	1.647493	2.471678	4.069546
H	1.383657	2.292989	2.330988
C	1.331585	0.448416	3.450681
H	0.847659	-0.125298	2.655182
H	2.410172	0.273717	3.356810
C	0.812745	-1.465175	4.930185
H	0.877745	-1.659374	6.004836
H	1.677875	-1.939156	4.452067
C	-0.474287	-2.050740	4.378664
H	-0.536628	-1.884871	3.298699
H	-0.500610	-3.133687	4.540393
C	-2.883906	-1.582678	4.265754
H	-3.720222	-1.571448	4.967653
H	-2.910872	-2.562016	3.775219
C	-3.044785	-0.498932	3.214258
H	-2.258645	-0.596566	2.461123
H	-4.004523	-0.616291	2.698144
C	-1.370209	3.928107	5.076164
C	-0.501083	3.695513	3.868395
H	0.474450	4.155250	4.109965
H	-0.911737	4.244354	3.011973
N	-1.533239	5.164126	5.517747
H	-2.100035	5.239636	6.352726
C	0.254547	6.798151	6.017244
C	1.508989	6.181660	5.887182
C	2.579610	6.624649	6.813699
C	0.933611	8.166546	7.845952
C	2.199121	7.616670	7.760819
H	2.954210	7.960994	8.462111
O	3.737737	6.140006	6.756627
N	-0.029815	7.756570	6.988321
C	-1.365557	8.339296	7.030509
H	-1.546491	8.968343	6.153753
H	-2.127207	7.557822	7.075532
H	-1.475325	8.952102	7.919661
C	-0.823051	6.368976	5.069282
H	-1.570346	7.141922	4.885210
H	-0.349087	6.142986	4.113842
O	1.742218	5.276574	5.011283
N	-2.950688	0.856961	3.802592
N	0.823475	-0.010634	4.741547
H	1.362743	0.412942	5.494301
C	-4.192976	1.189445	4.524164
H	-5.083589	0.897427	3.948279
H	-4.228948	2.275541	4.666810
C	-4.165432	0.633006	5.929888
O	-3.077228	0.563478	6.541818
C	0.631945	9.213837	8.877335
H	1.538541	9.414928	9.449918
H	0.305128	10.157938	8.428613
H	-0.140966	8.892082	9.584183
N	-1.628709	-1.393549	5.011858
H	-1.746786	-1.779287	5.945035
In	-1.226123	0.898611	5.374501
O	-0.133416	0.988033	7.064383
H	-0.567502	1.420377	7.801920
C	-6.573431	0.012927	5.833339
H	-6.851538	0.867317	5.213422
H	-7.327198	-0.068993	6.620196
N	-5.299887	0.285339	6.517551
H	-5.205945	0.002418	7.484050
C	-6.506942	-1.170338	4.915534
C	-6.606728	-0.931912	3.533055
C	-6.435667	-2.098597	2.630094

C	-6.086248	-3.502650	4.639688
C	-6.154544	-3.338432	3.269927
H	-5.981583	-4.206990	2.640699
C	-6.235267	-2.613863	6.900119
H	-5.220413	-2.464627	7.283879
H	-6.913831	-1.915968	7.386820
H	-6.562426	-3.616421	7.166099
O	-6.778520	0.239754	3.047026
O	-6.505027	-1.970618	1.382389
C	-5.785003	-4.843904	5.237164
H	-4.931827	-4.807253	5.922620
H	-6.638478	-5.253731	5.789455
H	-5.544690	-5.543723	4.435519
N	-6.288756	-2.439015	5.452727

pbe-gd3bj_L1_II.log

SCF (wB97x) = -2057.66989305
E(SCF)+ZPE(0 K)= -2056.932395
H(298 K)= -2056.889210
G(298 K)= -2057.003733
Lowest Frequency = 20.9544cm-1

O	-0.440800	4.424657	6.312444
N	0.390171	2.012133	4.485776
C	-2.080182	2.352160	4.527062
H	-1.979256	2.919931	5.453115
H	-2.904752	2.776298	3.936476
C	-0.787455	2.489260	3.744238
H	-0.849465	1.939303	2.800138
H	-0.675174	3.547936	3.472557
C	1.328846	1.317041	3.585713
H	2.305274	1.290672	4.075127
H	1.454463	1.865470	2.640068
C	0.929408	-0.113063	3.295521
H	-0.023432	-0.163799	2.761675
H	1.684903	-0.561510	2.636072
C	0.613631	-2.296111	4.375318
H	0.772744	-2.771814	5.348344
H	1.330649	-2.723847	3.662633
C	-0.808664	-2.535878	3.903087
H	-0.936928	-2.147799	2.888151
H	-1.017725	-3.611028	3.859265
C	-2.941587	-1.331277	4.135145
H	-3.802091	-1.471250	4.792631
H	-3.171637	-1.901566	3.227350
C	-2.797172	0.136485	3.760271
H	-2.025154	0.242201	2.994869
H	-3.738629	0.501354	3.328325
C	0.567764	3.726434	6.386159
C	1.161532	3.087511	5.143281
H	2.133477	2.672032	5.387096
H	1.311123	3.920193	4.436767
N	1.220003	3.608093	7.575995
H	0.776195	4.149410	8.306846
C	2.125274	1.591259	8.675177
C	1.582254	0.601161	7.880161
C	0.932169	-0.538942	8.483452
C	1.789107	0.267175	10.621175
C	1.125010	-0.694105	9.865361
H	0.713453	-1.557993	10.376765
O	0.203282	-1.273231	7.719378
N	2.246927	1.405164	10.036929
C	2.894409	2.455623	10.822581
H	3.900384	2.641354	10.441132
H	2.308335	3.376248	10.778229
H	2.973173	2.151121	11.860600
C	2.435204	2.892206	7.974918
H	3.028925	3.573145	8.585258
H	3.024484	2.669901	7.084781
O	1.471828	0.739015	6.599216
N	-2.414329	0.962945	4.919181
N	0.824370	-0.860651	4.550207
H	1.665070	-0.692738	5.101124
C	-3.533308	1.035901	5.878117
H	-4.475507	1.259410	5.361456
H	-3.303867	1.847623	6.576417
C	-3.610349	-0.179959	6.768887
O	-2.578515	-0.787695	7.093403
C	1.981204	0.053782	12.092530
H	1.555138	-0.911510	12.368110
H	3.040370	0.040899	12.370421
H	1.481602	0.821735	12.691870
N	-1.731269	-1.833491	4.792300
H	-1.995224	-2.436885	5.562221
In	-0.610074	0.077527	6.112781
O	-1.199558	1.519616	7.453124
H	-0.487008	2.076142	7.766088
C	-6.108198	-0.144218	6.714721
H	-6.183053	0.944180	6.670252
H	-6.853460	-0.480944	7.440924
N	-4.801726	-0.537058	7.257498
H	-4.768937	-1.307721	7.910257
C	-6.349450	-0.634723	5.317546
C	-6.454158	0.323743	4.292165
C	-6.551306	-0.184450	2.900318
C	-6.443138	-2.483770	3.810603
C	-6.517998	-1.599180	2.752613
H	-6.551035	-2.005529	1.745563
C	-6.336058	-2.943744	6.194630
H	-5.304449	-3.224430	6.432651
H	-6.803857	-2.503760	7.073227
H	-6.895067	-3.844990	5.951444
O	-6.411060	1.582798	4.516965
O	-6.627257	0.603082	1.923080
C	-6.414152	-3.963679	3.574922
H	-5.553911	-4.440404	4.056660
H	-7.318749	-4.461914	3.942490
H	-6.347699	-4.151252	2.502172
N	-6.388806	-2.007859	5.076838

pbe-gd3bj_L1_I.log

SCF (wB97x) = -2057.65879422
E(SCF)+ZPE(0 K)= -2056.921320
H(298 K)= -2056.878066
G(298 K)= -2056.991872
Lowest Frequency = 24.9213cm-1

O	-1.489760	3.723583	6.841803
N	0.596998	2.758663	4.869148
C	-1.671946	2.573262	3.830602
H	-2.138251	2.686318	4.808109
H	-2.320020	3.077849	3.094745
C	-0.324327	3.274160	3.841359
H	0.160896	3.214702	2.862581
H	-0.521157	4.340143	4.010082
C	1.951522	2.516970	4.341040
H	2.607803	2.368117	5.200170
H	2.320691	3.390846	3.781509
C	2.071289	1.296610	3.457244
H	1.487538	1.406689	2.545981
H	3.120057	1.205142	3.144698
C	1.898417	-1.150576	3.327192
H	1.576332	-2.011201	3.926559
H	2.980157	-1.227144	3.149787
C	1.194151	-1.187394	1.976320
H	1.429692	-0.293540	1.384287
H	1.665448	-2.020573	1.432032
C	-1.102188	-0.393591	1.464643
H	-2.109354	-0.818944	1.389108
H	-0.791660	-0.218260	0.425677
C	-1.170036	1.002540	2.095637
H	-0.194467	1.472745	2.000294
H	-1.858145	1.603804	1.475415
C	-0.292052	3.572974	7.097322
C	0.749814	3.685303	6.006518
H	1.738327	3.540651	6.428763
H	0.697843	4.727371	5.652962
N	0.119550	3.436048	8.378214

H	-0.656747	3.421030	9.027517
C	1.303031	1.441830	9.135683
C	1.195850	0.655652	8.004981
C	0.758079	-0.714848	8.128139
C	0.892203	-0.433724	10.548343
C	0.669958	-1.231138	9.434623
H	0.391543	-2.269043	9.585434
O	0.461959	-1.323985	7.050675
N	1.177633	0.885676	10.397375
C	1.377520	1.752071	11.557853
H	2.342056	2.258052	11.485751
H	0.577608	2.493753	11.625703
H	1.373401	1.161613	12.467799
C	1.383272	2.928538	8.912678
H	1.608824	3.490405	9.818640
H	2.183320	3.141028	8.203547
O	1.338636	1.120795	6.803307
N	-1.602597	1.130764	3.513112
N	1.651879	0.064498	4.146595
H	2.264706	-0.018530	4.959902
C	-2.957166	0.540014	3.570764
H	-2.864465	-0.472975	3.200417
H	-3.611200	1.094031	2.877099
C	-3.705782	0.541095	4.898082
O	-4.132517	1.589591	5.381827
C	0.801388	-1.028122	11.921917
H	0.551245	-2.085932	11.832153
H	1.749754	-0.956532	12.465171
H	0.025407	-0.551838	12.529758
N	-0.241665	-1.388907	2.054792
H	-0.533240	-1.592036	3.013026
In	-0.175072	0.203693	5.462087
O	-1.642136	0.756782	6.696616
H	-1.874603	1.687295	6.698783
C	-3.785689	-1.998840	4.970904
H	-3.477963	-1.946604	3.927511
H	-4.738395	-2.533478	4.968090
N	-4.038549	-0.650497	5.440309
H	-4.557874	-0.555721	6.301571
C	-2.732547	-2.822137	5.672510
C	-1.406458	-2.614043	5.342543
C	-0.428353	-3.671252	5.603023
C	-2.221713	-4.821196	6.860930
C	-0.919145	-4.732793	6.425576
H	-0.225384	-5.518286	6.710160
C	-4.518543	-3.971981	6.913305
H	-4.577836	-4.547171	7.833590
H	-4.909509	-2.977513	7.127330
H	-5.146082	-4.455026	6.157466
O	-1.062753	-1.539134	4.665198
O	0.721604	-3.655661	5.107721
C	-2.659361	-5.953386	7.740711
H	-2.961655	-5.606622	8.735406
H	-3.497690	-6.510997	7.311235
H	-1.825374	-6.644225	7.871189
N	-3.128199	-3.881467	6.477663

pbe-gd3bj_L1_IVac.log

SCF (wB97x) = -1562.55354366
E(SCF)+ZPE(0 K)= -1561.994153
H(298 K)= -1561.961032
G(298 K)= -1562.055944
Lowest Frequency = 18.5474cm-1

O	-1.301362	-1.463545	6.024751
N	-1.157299	1.169175	5.624141
C	-2.213521	2.178208	3.622056
H	-3.200851	1.772036	3.858738
H	-2.383392	3.135619	3.112892
C	-1.437579	2.421218	4.901259
H	-0.476481	2.892241	4.678784
H	-1.993361	3.120934	5.538812
C	-0.044162	1.356207	6.585226
H	-0.155429	0.631258	7.398249

H	-0.101689	2.350533	7.043552
C	1.300088	1.156191	5.917474
H	1.477099	1.934821	5.171896
H	2.095192	1.242858	6.666744
C	2.482121	-0.332257	4.349348
H	2.573664	-1.404379	4.155915
H	3.411487	0.014241	4.816140
C	2.251683	0.403041	3.039706
H	2.211432	1.485036	3.198945
H	3.091852	0.212273	2.361547
C	0.486959	0.833883	1.372634
H	-0.009851	0.203632	0.630929
H	1.307128	1.342980	0.853621
C	-0.478291	1.871249	1.928946
H	0.061770	2.568581	2.575182
H	-0.910906	2.462615	1.111319
C	-2.020301	-0.764855	6.771160
C	-2.330085	0.657505	6.340343
H	-2.579786	1.304624	7.184040
H	-3.193177	0.615778	5.669189
C	-1.692079	0.196714	9.680153
C	-0.627092	-0.679349	9.933684
C	0.654192	-0.077456	10.365963
C	-0.455784	2.141309	10.263590
C	0.655422	1.345817	10.464795
H	1.587574	1.831712	10.739406
O	1.667993	-0.781185	10.602231
N	-1.629168	1.567827	9.899538
C	-2.855333	2.356182	9.827054
H	-3.129335	2.594447	8.793837
H	-3.673944	1.814300	10.302803
H	-2.724716	3.291887	10.363668
C	-2.913767	-0.415926	9.043708
H	-3.421762	-1.095801	9.734992
H	-3.657373	0.309287	8.717228
O	-0.742290	-1.945771	9.746651
N	-1.535829	1.226233	2.722907
N	1.332921	-0.151014	5.250390
H	1.363831	-0.876403	5.962233
C	-2.537081	0.572251	1.871992
H	-2.041211	0.072265	1.034496
H	-3.240650	1.298747	1.448279
O	-4.403135	-0.860581	2.236243
C	-3.301832	-0.504004	2.645304
O	-2.702978	-0.975371	3.675280
C	-0.378627	3.627908	10.442675
H	0.663394	3.911346	10.598366
H	-0.748867	4.169382	9.566033
H	-0.946661	3.972507	11.314656
N	0.981832	-0.027176	2.448325
H	1.077279	-0.987009	2.121900
In	-0.630119	-0.567532	4.087371
O	0.005895	-2.374468	3.425498
H	-0.534385	-3.118465	3.696810
N	-2.500006	-1.244673	7.907366
H	-2.003559	-2.073796	8.245538

pbe-gd3bj_L1_IVhc.log

SCF (wB97x) = -1562.54005579
E(SCF)+ZPE(0 K)= -1561.980596
H(298 K)= -1561.947830
G(298 K)= -1562.039568
Lowest Frequency = 31.2781cm-1

O	-1.171513	4.263512	6.767606
N	0.724100	2.810860	4.722656
C	-1.659043	2.728307	4.009337
H	-1.955197	2.951532	5.035883
H	-2.374741	3.196457	3.317331
C	-0.283560	3.324851	3.781050
H	0.059181	3.124506	2.761081
H	-0.371671	4.417033	3.863996
C	2.022938	2.636223	4.044712
H	2.799831	2.604965	4.811956

H	2.246357	3.491132	3.388994
C	2.116691	1.349288	3.255604
H	1.391565	1.325481	2.437522
H	3.113168	1.290896	2.796194
C	2.116375	-1.089363	3.520118
H	2.125905	-1.838773	4.318253
H	3.086816	-1.121510	3.007355
C	0.989873	-1.383150	2.544193
H	1.045381	-0.700184	1.690659
H	1.091412	-2.399214	2.143724
C	-1.371661	-0.694691	2.376680
H	-2.301803	-1.184659	2.673035
H	-1.207619	-0.959609	1.324567
C	-1.514147	0.817993	2.478069
H	-0.611638	1.291712	2.085277
H	-2.353462	1.154712	1.851983
C	-0.060665	3.779881	6.979537
C	0.975511	3.706639	5.870632
H	1.925643	3.404966	6.296734
H	1.077266	4.743063	5.507704
N	0.324402	3.483051	8.246460
H	-0.436738	3.592518	8.902709
C	1.353892	1.343833	8.957168
C	1.328830	0.593573	7.796767
C	0.839419	-0.765913	7.832111
C	0.792910	-0.566796	10.262971
C	0.649291	-1.325069	9.105602
H	0.337891	-2.360236	9.201662
O	0.577270	-1.325989	6.704150
N	1.101325	0.753135	10.180054
C	1.202235	1.581736	11.380731
H	2.210183	1.992541	11.471480
H	0.477404	2.397512	11.339899
H	0.993055	0.987455	12.263665
C	1.520288	2.831412	8.778880
H	1.781927	3.342274	9.706519
H	2.348476	2.995775	8.089068
O	1.579115	1.099882	6.636408
N	-1.709098	1.257451	3.868035
N	1.883484	0.210494	4.143568
H	2.462424	0.316857	4.975963
C	-3.014406	0.782295	4.363985
H	-3.776947	0.870833	3.579950
H	-3.306695	1.423850	5.198664
O	-4.124031	-1.218497	4.977514
C	-3.026084	-0.644901	4.923360
O	-1.921358	-1.113691	5.332531
C	0.592816	-1.208459	11.603513
H	0.372142	-2.266663	11.458119
H	1.487882	-1.139209	12.230750
H	-0.243538	-0.767998	12.156237
N	-0.282391	-1.177967	3.224614
H	-0.574796	-2.021144	3.703375
In	-0.115134	0.328865	5.325998
O	-1.276843	1.195453	6.782868
H	-1.865295	0.515220	7.115033

pbe-gd3bj_L1.log

SCF (wB97x) = -1981.07358978
E(SCF)+ZPE(0 K)= -1980.331172
H(298 K)= -1980.289101
G(298 K)= -1980.404754
Lowest Frequency = 11.8385cm-1

O	2.158814	0.434638	6.634653
N	0.619584	1.959550	4.934349
C	-1.849219	1.750007	4.440447
H	-2.194214	1.991116	5.451544
H	-2.599770	2.199087	3.763289
C	-0.522731	2.449491	4.173733
H	-0.266832	2.355913	3.113942
H	-0.687851	3.530236	4.350947
C	1.852969	2.591431	4.458945
H	2.590621	2.577557	5.267441
H	1.680157	3.650110	4.200160
C	2.438961	1.849753	3.268158
H	1.675665	1.785728	2.477436
H	3.261954	2.445751	2.848786
C	2.805723	-0.518225	2.694925
H	3.241643	-1.425262	3.131544
H	3.417795	-0.259354	1.820477
C	1.393382	-0.844942	2.210284
H	0.983640	0.010401	1.642101
H	1.462816	-1.677013	1.497671
C	-0.854703	-1.453784	2.894174
H	-1.326989	-2.213531	3.532766
H	-0.866974	-1.867541	1.879387
C	-1.677466	-0.170292	2.937993
H	-1.160954	0.595424	2.350969
H	-2.666246	-0.314631	2.468347
C	1.441683	1.283907	7.155877
C	0.440395	2.106373	6.356578
H	0.480145	3.162636	6.682562
H	-0.549869	1.726590	6.629762
N	1.524699	1.523539	8.484821
H	2.179222	0.928191	8.976707
C	-0.322689	1.298624	10.069008
C	0.234638	0.296543	10.810763
C	-0.544202	-0.748104	11.431416
C	-2.499634	0.444379	10.556761
C	-1.945497	-0.615063	11.245385
H	-2.606893	-1.358637	11.675984
O	0.063910	-1.658012	12.046695
N	-1.698670	1.387192	9.971454
C	-2.275964	2.544267	9.284613
H	-1.866937	3.466573	9.700019
H	-2.081267	2.496418	8.211205
H	-3.350532	2.554740	9.426282
C	0.594715	2.250106	9.329835
H	0.045491	2.970427	8.729598
H	1.172255	2.826810	10.059701
O	1.571934	0.197883	10.956962
H	1.691872	-0.607367	11.497686
N	-1.809331	0.302317	4.314300
N	2.921368	0.541383	3.681044
H	2.467991	0.282444	4.553660
C	-2.946684	-0.313983	4.973391
H	-2.962187	-1.386767	4.752748
H	-3.909427	0.106074	4.638001
O	-3.802608	0.444465	7.083384
C	-2.902851	-0.139474	6.482119
N	-1.817509	-0.604737	7.142497
H	-1.871351	-0.488823	8.146228
C	-0.821193	-1.544859	6.649892
H	0.174178	-1.102106	6.694631
H	-1.019143	-1.735922	5.598020
C	-0.917219	-2.841192	7.407085
C	-1.952754	-3.689550	7.140906
C	-0.185554	-4.273190	9.169677
C	-2.174440	-4.907747	7.883836
C	-1.231907	-5.137682	8.919655
H	-1.336321	-6.021510	9.539424
O	-2.849053	-3.418097	6.173361
H	-3.462757	-4.177515	6.205910
O	-3.155314	-5.628364	7.569556
N	-0.026550	-3.145559	8.419231
C	1.122723	-2.263517	8.616447
H	1.782298	-2.292697	7.746188
H	0.788922	-1.239141	8.779278
H	1.680694	-2.576670	9.491467
N	0.529055	-1.249210	3.309695
H	0.528429	-0.516862	4.020956
C	-3.986870	0.562868	10.431537
H	-4.449405	-0.306112	10.900605
H	-4.365491	1.456242	10.939349
H	-4.292195	0.595107	9.380848
C	0.775554	-4.556650	10.282832
H	0.476969	-5.479606	10.781124
H	1.799416	-4.689276	9.918630

H	0.767658	-3.748377	11.023275

pbe-gd3bj_L1_TS(III).log

SCF (wB97x) = -2057.63934907
E(SCF)+ZPE(0 K)= -2056.901325
H(298 K)= -2056.859494
G(298 K)= -2056.973046
Lowest Frequency = -316.4147cm-1

O	-2.076078	2.845513	5.480287
N	-0.340238	1.988452	3.427206
C	-2.712650	1.893697	2.697615
H	-2.936535	2.839014	3.194667
H	-3.352956	1.810156	1.812160
C	-1.248958	1.887252	2.270199
H	-1.009108	0.963246	1.735037
H	-1.082109	2.713079	1.567149
C	1.024470	1.498461	3.128548
H	1.696563	1.936864	3.872118
H	1.348193	1.839697	2.137763
C	1.128437	-0.009514	3.224153
H	0.510045	-0.504536	2.471353
H	2.164378	-0.311333	3.030904
C	0.562206	-1.906661	4.685804
H	0.607869	-2.158617	5.749652
H	1.384214	-2.429091	4.183206
C	-0.768788	-2.344843	4.103428
H	-0.798526	-2.159946	3.025487
H	-0.908791	-3.421115	4.249042
C	-3.119391	-1.638257	3.978057
H	-3.953371	-1.611171	4.683157
H	-3.211807	-2.587147	3.439003
C	-3.223913	-0.488844	2.988673
H	-2.471082	-0.594336	2.202529
H	-4.205996	-0.511654	2.501271
C	-0.890634	3.339727	5.435564
C	-0.277934	3.339695	4.027941
H	0.751583	3.693461	4.009692
H	-0.885460	4.038864	3.443773
N	-0.615897	4.450340	6.198966
H	-1.195262	4.459151	7.028176
C	1.573879	4.409942	7.492641
C	2.419243	3.332255	7.215434
C	3.343333	2.881255	8.272053
C	2.358750	4.617812	9.737051
C	3.243389	3.584857	9.507716
H	3.897187	3.279048	10.319497
O	4.153441	1.937810	8.086281
N	1.528855	5.020622	8.742613
C	0.615889	6.139357	8.953684
H	-0.359802	5.925959	8.517313
H	0.466242	6.299043	10.017548
H	1.010410	7.060804	8.513597
C	0.736949	4.983245	6.380242
H	0.653090	6.068353	6.479994
H	1.294806	4.801400	5.461902
O	2.392804	2.719153	6.070354
N	-3.019042	0.809980	3.662141
N	0.696923	-0.449977	4.561036
H	1.381777	-0.119054	5.236526
C	-4.179460	1.145679	4.508623
H	-5.126824	0.916298	4.001356
H	-4.149695	2.221502	4.709680
C	-4.068532	0.515793	5.882681
O	-2.944429	0.335939	6.416001
C	2.317486	5.301190	11.071735
H	3.087081	4.869494	11.713271
H	2.510022	6.376288	10.993142
H	1.353679	5.169215	11.576370
N	-1.852919	-1.578764	4.730999
H	-2.019904	-1.960114	5.658390
In	-1.261904	0.707211	5.112962
O	-0.030820	1.929826	6.202215
H	0.959200	2.075086	6.120443
C	-6.514761	0.064922	5.955842
H	-6.778850	0.968968	5.403955
H	-7.206180	-0.006987	6.798933
N	-5.172554	0.211057	6.543135
H	-5.023175	-0.126155	7.485132
C	-6.605539	-1.068192	4.978899
C	-6.803048	-0.748994	3.622971
C	-6.759286	-1.870502	2.649974
C	-6.347248	-3.401457	4.551623
C	-6.507271	-3.157929	3.201498
H	-6.431838	-3.997743	2.516566
C	-6.267216	-2.636667	6.858495
H	-6.830391	-1.907009	7.436844
H	-6.670656	-3.617167	7.102549
H	-5.214132	-2.606885	7.158152
O	-6.950015	0.454081	3.212965
O	-6.904532	-1.668155	1.418953
C	-6.083655	-4.787762	5.056264
H	-5.183404	-4.834845	5.677900
H	-6.918070	-5.179187	5.649529
H	-5.942608	-5.455167	4.204922
N	-6.422079	-2.375032	5.431502

pbe-gd3bj_L1_TS(II).log

SCF (wB97x) = -2057.65658591
E(SCF)+ZPE(0 K)= -2056.918631
H(298 K)= -2056.876746
G(298 K)= -2056.988346
Lowest Frequency = -247.2933cm-1

O	-1.823163	3.114290	6.208263
N	0.424290	2.037452	4.391331
C	-1.848978	1.464506	3.558245
H	-2.307012	2.009341	4.383278
H	-2.501073	1.570314	2.679513
C	-0.505264	2.102355	3.259931
H	-0.035374	1.614059	2.401361
H	-0.688284	3.143114	2.951813
C	1.808830	1.872725	3.919206
H	2.475941	2.095971	4.755016
H	2.043326	2.583628	3.111412
C	2.125194	0.467971	3.451519
H	1.483410	0.166445	2.618461
H	3.157495	0.448871	3.076518
C	2.339769	-1.854413	4.215222
H	2.457970	-2.406184	5.153363
H	3.295375	-1.888422	3.676843
C	1.237450	-2.482712	3.380187
H	1.171917	-1.988424	2.406187
H	1.458428	-3.538537	3.188034
C	-1.209258	-2.254202	3.189670
H	-2.055674	-2.680709	3.729524
H	-1.050382	-2.855626	2.286558
C	-1.515667	-0.822451	2.783881
H	-0.665680	-0.404551	2.235831
H	-2.375879	-0.808416	2.100239
C	-0.609267	3.170908	6.438948
C	0.382148	3.211834	5.287686
H	1.382086	3.372683	5.682512
H	0.114016	4.113555	4.714261
N	-0.193707	3.353333	7.714161
H	-0.976712	3.384265	8.355195
C	1.155694	1.689852	8.888074
C	1.070040	0.697540	7.936808
C	0.910197	-0.682904	8.310971
C	1.131133	0.068088	10.623731
C	0.981318	-0.951210	9.687852
H	0.900291	-1.971880	10.046498
O	0.699766	-1.527096	7.364882
N	1.198756	1.366565	10.227023
C	1.326214	2.455177	11.196126
H	2.219390	3.043922	10.979261
H	0.443253	3.097433	11.159256
H	1.417372	2.053735	12.199551

C	1.097031	3.099035	8.350594
H	1.245350	3.858579	9.118113
H	1.889791	3.229070	7.611658
O	1.048787	0.983514	6.674295
N	-1.779262	0.038168	3.951402
N	1.958206	-0.483257	4.553466
H	2.504905	-0.152635	5.344552
C	-3.052166	-0.337479	4.622468
H	-3.601977	-1.059026	4.006191
H	-3.686611	0.547116	4.739067
C	-2.776643	-1.002895	5.965103
O	-1.841094	-1.881967	5.998895
C	1.208092	-0.269337	12.082250
H	1.137674	-1.351240	12.198965
H	2.154339	0.052339	12.529580
H	0.390710	0.181519	12.654207
N	-0.038425	-2.311546	4.075065
H	-0.178091	-3.067702	4.737058
In	-0.137347	-0.438735	5.591171
O	-1.805346	0.339233	6.720503
H	-2.090808	1.254455	6.587852
C	-4.921152	-0.226793	6.960383
H	-4.483223	0.776375	6.980604
H	-5.339166	-0.415588	7.954822
N	-3.878516	-1.236715	6.754404
H	-3.602470	-1.727808	7.593819
C	-6.001487	-0.193746	5.918299
C	-6.096940	0.929401	5.075697
C	-7.197487	0.936010	4.081011
C	-7.908645	-1.267498	4.961702
C	-8.051346	-0.201886	4.092564
H	-8.866035	-0.231991	3.373969
C	-6.766773	-2.327414	6.844377
H	-7.103537	-1.988073	7.829748
H	-7.367256	-3.183209	6.550179
H	-5.725558	-2.643092	6.905389
O	-5.266308	1.904180	5.126156
O	-7.353649	1.896344	3.279632
C	-8.871266	-2.417684	4.919029
H	-8.378137	-3.365939	4.676702
H	-9.403064	-2.551223	5.867378
H	-9.615745	-2.225779	4.144652
N	-6.897338	-1.258782	5.859778

pbe-gd3bj_L1_TS(I).log

SCF (wB97x) = -2057.63908113
E(SCF)+ZPE(0 K)= -2056.902273
H(298 K)= -2056.860026
G(298 K)= -2056.972244
Lowest Frequency = -188.4934cm-1

O	-1.989483	3.332101	6.734908
N	0.501957	2.504593	5.332100
C	-1.526266	2.437809	3.845913
H	-2.192452	2.524843	4.701622
H	-1.990103	2.978796	3.005725
C	-0.202991	3.105371	4.171895
H	0.467411	3.084060	3.308618
H	-0.405962	4.163436	4.372355
C	1.945637	2.342708	5.044064
H	2.445933	2.110577	5.990128
H	2.377636	3.282865	4.672428
C	2.249712	1.224173	4.068335
H	1.853077	1.437451	3.075347
H	3.339759	1.147592	3.961759
C	2.055665	-1.226038	3.763259
H	1.678649	-2.089857	4.320871
H	3.152017	-1.290846	3.722679
C	1.520041	-1.257972	2.343034
H	1.799685	-0.339030	1.810826
H	2.069976	-2.064282	1.834346
C	-0.674674	-0.489679	1.538026
H	-1.672433	-0.893391	1.337318
H	-0.224365	-0.337274	0.548320
C	-0.783643	0.905257	2.157733
H	0.210905	1.348649	2.205920
H	-1.367451	1.532126	1.462585
C	-0.900064	3.307953	7.299555
C	0.411426	3.354793	6.537656
H	1.224099	3.070783	7.204783
H	0.584701	4.407953	6.264415
N	-0.824839	3.414029	8.652327
H	-1.732511	3.353843	9.090148
C	0.934779	1.740095	9.347029
C	0.582132	0.850788	8.346516
C	1.428200	-0.287456	8.075264
C	2.745363	0.320108	10.021878
C	2.485215	-0.535361	8.964968
H	3.120912	-1.402061	8.820383
O	1.162150	-0.956166	7.020631
N	1.997793	1.442686	10.187390
C	2.269776	2.360738	11.294138
H	2.479281	3.362887	10.916211
H	1.414797	2.397558	11.973413
H	3.136231	2.024910	11.852436
C	0.306563	3.113560	9.504601
H	-0.045423	3.263081	10.528789
H	1.086298	3.871670	9.349135
O	-0.416537	1.007096	7.528174
N	-1.399546	1.007640	3.502855
N	1.689571	-0.034833	4.562625
H	2.117033	-0.225850	5.471761
C	-2.738873	0.398422	3.418498
H	-2.592925	-0.625731	3.091688
H	-3.319765	0.914492	2.637477
C	-3.648367	0.408498	4.662663
O	-4.328306	1.434949	4.921820
C	3.857782	0.010117	10.976745
H	4.322366	-0.931175	10.681139
H	4.634939	0.781316	10.971994
H	3.497639	-0.104325	12.004328
N	0.088678	-1.485347	2.249636
H	-0.318761	-1.709184	3.157396
In	-0.363318	0.102026	5.577534
O	-2.382804	0.345100	5.973067
H	-2.546301	1.168156	6.449638
C	-3.687733	-2.134714	4.759087
H	-3.053956	-2.163128	3.871775
H	-4.491646	-2.853354	4.566658
N	-4.308048	-0.825343	4.831421
H	-5.024537	-0.724107	5.537194
C	-2.821560	-2.703869	5.865252
C	-1.442399	-2.657192	5.741208
C	-0.624666	-3.645995	6.445968
C	-2.683848	-4.259154	7.671323
C	-1.327542	-4.356038	7.473607
H	-0.758893	-5.031369	8.105979
C	-4.881473	-3.431839	6.984432
H	-5.359211	-4.159373	6.320052
H	-5.165683	-3.655282	8.009806
H	-5.255313	-2.435843	6.756050
O	-0.865673	-1.812947	4.898132
O	0.571403	-3.865294	6.153851
C	-3.360638	-5.036844	8.759379
H	-3.818432	-4.380727	9.508334
H	-4.142845	-5.696747	8.370650
H	-2.619881	-5.655138	9.268189
N	-3.428727	-3.463287	6.853811

pbe-gd3bj_L2_III.log

SCF (wB97x) = -2057.67085838
E(SCF)+ZPE(0 K)= -2056.934373
H(298 K)= -2056.890552
G(298 K)= -2057.009918
Lowest Frequency = 12.0490cm-1

O	0.193761	1.475546	7.910147
N	0.932968	2.669002	5.555564

C	-1.537935	2.729581	5.486496
H	-1.595078	3.060216	6.526506
H	-2.392911	3.161086	4.955386
C	-0.246016	3.199928	4.843136
H	-0.203657	2.854515	3.807148
H	-0.216470	4.296234	4.814565
C	2.144719	2.729545	4.707111
H	3.007985	2.581060	5.362962
H	2.245347	3.720988	4.249126
C	2.149088	1.649009	3.638610
H	1.357256	1.797979	2.898721
H	3.102246	1.694156	3.097738
C	1.775090	-0.802411	3.405385
H	2.232584	-1.669206	3.891198
H	2.288392	-0.664234	2.447152
C	0.296353	-1.049564	3.151796
H	-0.120922	-0.221524	2.572799
H	0.159752	-1.960713	2.556170
C	-1.887388	-0.818001	4.215357
H	-2.460317	-1.278188	5.024529
H	-2.248734	-1.248663	3.274342
C	-2.125305	0.681939	4.231783
H	-1.628278	1.172854	3.390468
H	-3.197236	0.882239	4.130965
C	1.006338	2.427750	8.001624
C	1.156838	3.373286	6.832438
H	2.110652	3.915111	6.827965
H	0.382330	4.137068	6.981776
N	1.687591	2.641760	9.112129
H	1.480928	1.998692	9.865127
C	4.026509	2.993704	8.373071
C	4.430954	3.806147	7.298433
C	5.470820	3.259309	6.390088
C	5.506524	1.217398	7.788936
C	5.933562	1.949145	6.697986
H	6.665892	1.499610	6.033176
O	5.875436	3.914068	5.397197
N	4.588847	1.745241	8.632116
C	4.141060	0.970736	9.783048
H	3.843434	1.635993	10.591280
H	3.301828	0.318798	9.519014
H	4.956056	0.355597	10.158713
C	2.892342	3.480971	9.224759
H	3.121497	3.540272	10.291743
H	2.671498	4.496109	8.888653
O	3.893798	4.942278	7.053634
N	-1.602914	1.257705	5.482956
N	1.962817	0.352947	4.288067
H	2.753246	0.164560	4.903368
C	-0.287034	-2.438447	5.060604
H	0.770112	-2.723395	5.035194
H	-0.835096	-3.239931	4.546301
C	-0.681788	-2.333499	6.517744
O	-0.812984	-1.202059	7.042595
C	6.041791	-0.158265	8.048136
H	6.676111	-0.457161	7.212348
H	6.649796	-0.203448	8.959021
H	5.240596	-0.897887	8.146547
N	-0.457990	-1.136577	4.415991
H	-2.206187	0.958330	6.245405
In	0.411683	0.366911	6.012050
O	2.048559	-0.540862	6.778549
H	1.975011	-0.834996	7.687684
C	-0.587678	-4.811133	6.753222
H	-1.259767	-5.073895	5.930806
H	-0.837431	-5.463681	7.594597
N	-0.865663	-3.441120	7.214789
H	-1.101772	-3.298644	8.188051
C	0.800408	-4.972952	6.217207
C	0.938090	-5.309170	4.856852
C	2.301385	-5.187308	4.271609
C	3.110060	-4.444088	6.491496
C	3.314800	-4.711331	5.150915
H	4.310982	-4.561291	4.743993
C	1.677175	-4.400196	8.453550
H	1.508402	-3.344627	8.690315
H	0.825117	-4.984390	8.794857
H	2.550152	-4.748348	9.003716
O	-0.055921	-5.619511	4.119427
O	2.513296	-5.442913	3.060282
C	4.234175	-3.989421	7.371333
H	3.973285	-3.089434	7.937438
H	4.531856	-4.759305	8.093001
H	5.103819	-3.762160	6.752820
N	1.876800	-4.602970	7.022608

pbe-gd3bj_L2_II.log

SCF (wB97x) = -2057.67918700
E(SCF)+ZPE(0 K)= -2056.941411
H(298 K)= -2056.898216
G(298 K)= -2057.014454
Lowest Frequency = 13.8899cm-1

O	0.396535	1.892676	8.110645
N	1.199417	2.684073	5.643404
C	-1.228400	2.845253	5.374642
H	-1.297568	3.217833	6.400571
H	-2.026249	3.326231	4.795426
C	0.125164	3.185830	4.777913
H	0.209259	2.718172	3.794632
H	0.220804	4.269437	4.621840
C	2.464216	2.486067	4.927743
H	3.239257	2.285458	5.676012
H	2.765119	3.395045	4.386306
C	2.383280	1.322472	3.965342
H	1.679186	1.524637	3.153571
H	3.365125	1.174554	3.498606
C	1.660439	-0.991229	3.715355
H	1.894118	-1.928952	4.218532
H	2.310320	-0.905789	2.835378
C	0.209915	-0.979074	3.265027
H	-0.020880	-0.002626	2.832875
H	0.076990	-1.709824	2.451053
C	-2.033624	-0.649124	4.135273
H	-2.696254	-1.020779	4.920287
H	-2.455777	-0.965310	3.165383
C	-2.000890	0.870324	4.172116
H	-1.444887	1.277162	3.322056
H	-3.029968	1.240168	4.071234
C	1.225113	2.817445	8.075256
C	1.402546	3.575936	6.786062
H	2.350709	4.116670	6.719374
H	0.621165	4.346537	6.807358
N	1.899078	3.201363	9.154297
H	1.690477	2.676658	9.992104
C	4.223692	3.596442	8.347617
C	4.633957	4.376726	7.250312
C	5.680065	3.801752	6.367526
C	5.715119	1.808542	7.837367
C	6.146292	2.505793	6.724008
H	6.882002	2.034963	6.077636
O	6.087738	4.420229	5.350159
N	4.792516	2.364015	8.654568
C	4.338540	1.644501	9.839094
H	4.113743	2.349424	10.638700
H	3.452358	1.049317	9.600165
H	5.129382	0.990794	10.200991
C	3.067822	4.092658	9.166636
H	3.309568	4.255886	10.220966
H	2.801831	5.066516	8.748670
O	4.094838	5.502420	6.966491
N	-1.396319	1.390128	5.403462
N	1.949164	0.106995	4.659865
H	2.714247	-0.198416	5.257978
C	-0.793273	-2.695056	4.589364
H	0.178253	-3.126602	4.361805
H	-1.518693	-3.142554	3.887335
C	-1.239578	-3.116252	5.980145
O	-2.295146	-2.679272	6.448205

C	6.248044	0.440417	8.140414
H	6.904810	0.127027	7.327358
H	6.833255	0.414032	9.066868
H	5.445472	-0.299220	8.231750
N	-0.717177	-1.240371	4.369593
H	-1.970916	1.109040	6.201464
In	0.387353	0.278696	6.426300
O	-1.199479	-0.229658	7.633643
H	-1.586291	-1.071173	7.371263
C	0.830238	-4.469494	6.612801
H	1.201980	-4.345583	5.592780
H	0.900575	-5.532400	6.843077
N	-0.582087	-4.102013	6.648407
H	-1.045086	-4.317668	7.522521
C	1.655094	-3.610978	7.542942
C	1.610929	-2.256054	7.295557
C	2.327257	-1.313633	8.112191
C	3.110731	-3.248695	9.384672
C	3.078257	-1.875016	9.160257
H	3.650526	-1.241331	9.827507
C	2.432941	-5.543696	8.809296
H	1.424020	-5.908743	9.013281
H	2.836477	-6.048354	7.929047
H	3.061647	-5.782200	9.660178
O	0.922921	-1.785588	6.311767
O	2.211815	-0.073079	7.804764
C	3.931556	-3.793197	10.514914
H	3.320201	-4.327974	11.249045
H	4.715627	-4.472749	10.165307
H	4.417741	-2.963903	11.029839
N	2.407129	-4.097697	8.591020

pbe-gd3bj_L2_I.log

SCF (wB97x) = -2057.67992944
E(SCF)+ZPE(0 K)= -2056.941913
H(298 K)= -2056.899128
G(298 K)= -2057.011062
Lowest Frequency = 29.1329cm-1

O	-0.038240	4.091347	6.551459
N	1.132580	2.016679	4.602298
C	-1.151767	2.838124	3.990698
H	-1.207394	3.646004	4.721173
H	-1.741321	3.125604	3.112500
C	0.298495	2.644053	3.562727
H	0.311511	1.993175	2.685512
H	0.724968	3.607114	3.248826
C	2.187560	1.184736	3.981345
H	2.875415	0.890394	4.776478
H	2.752903	1.764586	3.236455
C	1.655894	-0.075178	3.328160
H	0.945739	0.155277	2.528630
H	2.494807	-0.602670	2.857650
C	0.190973	-2.008861	3.625212
H	0.289695	-2.933923	4.205822
H	0.593782	-2.210381	2.624708
C	-1.255686	-1.584236	3.489545
H	-1.320886	-0.717214	2.830437
H	-1.841817	-2.386687	3.020487
C	-3.070364	-0.399981	4.556557
H	-3.531143	-0.232271	5.531263
H	-3.778745	-0.964876	3.932974
C	-2.761213	0.935952	3.885557
H	-2.466281	0.812737	2.839522
H	-3.684912	1.530839	3.879587
C	1.009847	3.466195	6.705561
C	1.804963	2.989695	5.497853
H	2.740191	2.545660	5.826493
H	2.050483	3.900405	4.931360
N	1.531922	3.335750	7.952421
H	0.924401	3.748094	8.648912
C	1.990117	1.181021	9.012167
C	1.452574	0.351996	8.055311
C	0.559492	-0.722307	8.417525
C	1.047032	-0.148592	10.742557
C	0.407586	-0.941321	9.800069
H	-0.245647	-1.731327	10.151277
O	-0.055654	-1.314357	7.470652
N	1.818823	0.902100	10.353741
C	2.490958	1.756119	11.331488
H	3.546038	1.855868	11.071860
H	2.025258	2.744681	11.364067
H	2.428853	1.311971	12.319719
C	2.579305	2.469218	8.491766
H	3.113291	3.036219	9.254879
H	3.295614	2.239149	7.702133
O	1.593209	0.610204	6.787892
N	-1.690359	1.621121	4.602715
N	0.981242	-0.959042	4.291782
H	1.688094	-1.454075	4.851746
C	-2.157408	-2.463162	5.519110
H	-1.293508	-3.126948	5.427728
H	-2.995850	-2.975919	5.016645
C	-2.555817	-2.334822	6.978400
O	-3.124358	-1.339516	7.432216
C	0.876227	-0.442971	12.202512
H	0.186503	-1.280449	12.316367
H	1.821744	-0.725273	12.678670
H	0.462153	0.408573	12.751420
N	-1.855089	-1.214107	4.792139
H	-2.026612	1.829497	5.551289
In	-0.234632	0.213181	5.800742
O	-1.484247	1.120510	7.179721
H	-2.012446	0.420735	7.579651
C	-1.559598	-4.625816	7.476365
H	-1.729888	-4.966540	6.453478
H	-1.944121	-5.407120	8.137273
N	-2.381024	-3.436017	7.739604
H	-2.731998	-3.314939	8.678868
C	-0.077713	-4.406955	7.603772
C	0.668042	-4.263763	6.424729
C	1.986729	-3.612546	6.554694
C	1.776264	-3.859564	8.999539
C	2.507191	-3.538042	7.871534
H	3.516502	-3.158175	8.003911
C	-0.323923	-4.583520	10.025361
H	-1.209266	-3.946449	10.085233
H	-0.639167	-5.629510	9.980024
H	0.247950	-4.439102	10.936764
O	0.190986	-4.553490	5.268445
O	2.590243	-3.142448	5.545145
C	2.407519	-3.786335	10.357512
H	1.905717	-3.067384	11.013353
H	2.417385	-4.757956	10.864044
H	3.442977	-3.460499	10.245674
N	0.483912	-4.240202	8.862931

pbe-gd3bj_L2_IVac.log

SCF (wB97x) = -1562.55444645
E(SCF)+ZPE(0 K)= -1561.994858
H(298 K)= -1561.961852
G(298 K)= -1562.057418
Lowest Frequency = 16.1241cm-1

C	-1.806807	1.905118	3.380734
H	-1.909823	2.504512	4.290158
H	-2.714196	2.054968	2.782067
C	-0.583089	2.390448	2.618258
H	-0.485151	1.896203	1.647094
H	-0.691448	3.463746	2.419051
C	1.899160	2.361286	2.790435
H	2.590600	2.675356	3.577328
H	1.841989	3.181369	2.065234
C	2.413627	1.111226	2.095427
H	1.751579	0.855425	1.264068
H	3.408118	1.294379	1.668863
C	2.375643	-1.319047	2.280638
H	2.777139	-2.105411	2.925443

H	3.004086	-1.278780	1.382627
C	0.946858	-1.668755	1.909934
H	0.524206	-0.930550	1.222448
H	0.929050	-2.635467	1.394812
C	-1.328025	-1.788087	2.858083
H	-1.800108	-2.283221	3.709144
H	-1.527579	-2.404248	1.974251
C	-1.909188	-0.402128	2.642146
H	-1.450353	0.047671	1.757883
H	-2.985455	-0.479668	2.440234
N	-1.669832	0.492980	3.791065
N	2.441328	-0.039285	3.015599
C	-2.544717	0.153701	4.922681
H	-3.528733	-0.188991	4.582145
H	-2.707068	1.053821	5.524195
O	-2.722576	-1.466052	6.645767
C	-1.954205	-0.887029	5.882008
O	-0.684736	-1.050919	5.848466
N	0.117690	-1.701364	3.122645
H	0.393723	-2.507298	3.678232
C	3.607070	-0.010084	3.893561
N	4.335692	-1.072631	5.976429
H	4.512609	-0.344148	3.370655
H	3.776585	1.015305	4.239550
C	5.560394	-0.271204	6.011782
C	5.263281	1.077706	6.613998
H	5.989956	-0.221478	5.011965
H	6.267036	-0.831996	6.631832
C	4.536048	1.068579	7.811415
N	5.633671	2.239218	5.946003
C	4.082032	2.374975	8.335183
O	4.234538	-0.031418	8.406299
C	5.213209	3.445344	6.401367
C	6.548586	2.141967	4.813075
C	4.449392	3.507821	7.550442
O	3.406661	2.463802	9.391849
H	4.039856	-1.304909	6.928963
C	5.578436	4.694655	5.656257
H	7.381662	1.481580	5.061380
H	6.045188	1.768554	3.915767
H	6.959358	3.120975	4.584745
H	4.110701	4.484186	7.885652
H	5.059804	5.540252	6.110505
H	6.653000	4.907181	5.698217
H	5.286263	4.647697	4.601987
N	0.602355	2.108135	3.422723
H	0.564379	2.640104	4.290855
C	3.345033	-0.853157	5.128298
O	2.195136	-1.303223	5.329457
In	0.572567	0.047213	4.529174
O	1.225137	1.404929	5.887595
H	1.524777	1.066746	6.733371

pbe-gd3bj_L2_IVhc.log

SCF (wB97x) = -1562.56837977
E(SCF)+ZPE(0 K)= -1562.008781
H(298 K)= -1561.976045
G(298 K)= -1562.068396
Lowest Frequency = 23.8111cm-1

C	-1.885482	2.445926	3.961026
H	-2.300864	2.615917	4.960389
H	-2.542040	2.957378	3.242625
C	-0.485735	3.030967	3.918029
H	-0.035518	2.918523	2.925818
H	-0.537549	4.108075	4.123579
C	1.770093	2.691568	4.865875
H	2.146110	2.649457	5.886241
H	1.907126	3.715930	4.496368
C	2.570426	1.746335	3.979069
H	2.237525	1.870147	2.944700
H	3.629787	2.017753	4.020575
C	2.530308	-0.525263	3.130596
H	2.624936	-1.559025	3.477626

H	3.455743	-0.268599	2.596131
C	1.343662	-0.450902	2.188859
H	1.214492	0.551746	1.769904
H	1.525626	-1.126237	1.342219
C	-1.106986	-0.716585	2.147999
H	-1.800265	-1.474346	2.524300
H	-0.942918	-0.932509	1.084878
C	-1.702654	0.672704	2.305322
H	-1.024924	1.407846	1.862628
H	-2.653579	0.745794	1.758821
N	-1.874007	0.996683	3.723393
N	2.373139	0.325018	4.329436
C	-3.061606	0.374432	4.299514
H	-3.237486	-0.594033	3.819386
H	-3.962348	0.980360	4.135853
O	-1.704416	-0.017850	6.227538
C	-2.899819	0.087263	5.794688
O	-3.916685	-0.063143	6.474985
N	0.132465	-0.822844	2.915226
H	0.239381	-1.768241	3.282968
O	4.344968	1.692851	6.281481
C	3.755053	0.635318	6.500253
N	3.756541	0.114757	7.759580
H	4.232978	0.742742	8.395567
C	2.736153	-0.710085	8.408613
H	2.285907	-1.365515	7.661290
H	3.231582	-1.339937	9.147182
C	1.669151	0.179039	8.994671
C	0.931074	0.886028	8.061502
C	0.072064	1.981129	8.487076
C	0.671329	1.345173	10.801278
C	-0.036850	2.118270	9.900297
H	-0.691460	2.890455	10.292746
O	1.131558	0.634987	6.805069
O	-0.490106	2.741210	7.650516
C	0.497062	1.555323	12.275743
H	-0.220084	2.361355	12.435671
H	0.109347	0.661349	12.775967
H	1.433548	1.838598	12.767247
C	2.316178	-0.426529	11.273228
H	2.133648	-1.485601	11.079311
H	3.383751	-0.214961	11.169967
H	2.024599	-0.215598	12.297001
N	1.536247	0.395664	10.351960
C	3.285721	-0.243163	5.353890
H	2.804273	-1.153751	5.713807
H	4.229483	-0.529558	4.862731
N	0.342282	2.344211	4.904259
H	-0.000879	2.560782	5.858767
In	0.093666	0.095429	5.074811
O	0.427473	-1.924622	5.319313
H	0.271424	-2.245800	6.208758

pbe-gd3bj_L2.log

SCF (wB97x) = -1981.07224657
E(SCF)+ZPE(0 K)= -1980.330184
H(298 K)= -1980.288356
G(298 K)= -1980.401929
Lowest Frequency = 17.8412cm-1

O	2.657073	3.377056	6.055100
O	3.290130	-0.285263	5.038350
N	0.208979	2.118563	5.178772
N	1.773825	-1.064185	2.701084
C	-0.937663	-0.030259	5.648710
H	-0.593045	-0.003069	6.689887
H	-1.931160	-0.495701	5.666668
C	-1.061301	1.395643	5.111952
H	-1.375629	1.354808	4.065463
H	-1.859525	1.938160	5.651184
C	0.352105	3.086645	4.091251
H	1.136722	3.791983	4.370250
H	-0.585112	3.654449	3.944758
C	0.752365	2.416990	2.783758

H	-0.006962	1.662152	2.512306
H	0.728827	3.172584	1.986830
C	2.519843	1.089657	1.727950
H	3.593833	0.893618	1.840032
H	2.416061	1.694211	0.817008
C	1.783422	-0.237795	1.510625
H	0.739151	-0.034490	1.251299
H	2.227122	-0.759456	0.640730
C	0.627694	-1.949189	2.827855
H	0.933663	-2.840791	3.382840
H	0.272257	-2.295825	1.844325
C	-0.506437	-1.277307	3.588381
H	-0.888999	-0.434978	2.984138
H	-1.338124	-1.985108	3.703477
C	1.817992	3.021243	6.873163
C	3.243532	-1.435270	4.596581
C	3.038301	-1.643385	3.095317
H	3.137360	-2.693328	2.784155
H	3.857502	-1.099156	2.615789
C	0.379787	2.692558	6.497575
H	-0.211559	3.620395	6.635977
H	-0.016179	1.976350	7.219233
N	2.128073	2.975781	8.194096
H	3.110797	3.119129	8.376275
N	3.342784	-2.496078	5.418256
H	3.492994	-2.272202	6.408289
C	3.354895	-3.907222	5.062840
H	4.346469	-4.215042	4.716493
H	2.652664	-4.103917	4.250340
C	3.015687	-4.752563	6.255727
C	4.010754	-5.310090	7.005702
C	3.741492	-6.143062	8.157127
C	1.373725	-5.766639	7.659982
C	2.363799	-6.342736	8.428969
H	2.076905	-6.975008	9.262057
C	1.330000	0.830661	9.128116
C	2.460051	0.132164	8.800152
C	2.441881	-1.286765	8.552133
C	0.064194	-1.198257	9.098235
C	1.180823	-1.906726	8.706147
H	1.095633	-2.972263	8.528938
O	5.312248	-5.118819	6.721909
H	5.774706	-5.636257	7.409988
O	3.524066	-1.840562	8.208207
O	4.718877	-6.614446	8.791103
N	1.694397	-4.961483	6.608345
N	0.143879	0.145251	9.320860
C	0.654709	-4.330334	5.795210
H	-0.297227	-4.374602	6.314968
H	0.889276	-3.276057	5.630390
H	0.548793	-4.850438	4.838371
C	-1.019902	0.886725	9.812976
H	-0.729770	1.518881	10.652392
H	-1.462934	1.498047	9.021294
H	-1.772168	0.191163	10.169018
C	1.366076	2.340432	9.249567
H	0.361805	2.762135	9.273075
H	1.834051	2.624635	10.197307
O	3.657821	0.726752	8.663075
H	4.245979	-0.005292	8.390018
N	-0.027703	-0.876897	4.898720
H	0.845388	-0.368913	4.767275
N	2.093636	1.870730	2.872269
H	2.167514	1.291675	3.705761
C	-1.249531	-1.894734	9.271659
H	-1.141762	-2.935390	8.967914
H	-1.583020	-1.889184	10.314592
H	-2.035292	-1.444073	8.657844
C	-0.067144	-6.045322	7.961322
H	-0.585499	-6.472646	7.097537
H	-0.126342	-6.761390	8.781238
H	-0.612576	-5.147146	8.267536

pbe-gd3bj_L2_TS(III).log

SCF (wB97x) = -2057.64263208
E(SCF)+ZPE(0 K)= -2056.905059
H(298 K)= -2056.862854
G(298 K)= -2056.977163
Lowest Frequency = -245.2659cm-1

O	-0.290581	2.067549	7.742858
N	0.952631	2.650544	5.280170
C	-1.469905	3.012760	4.985494
H	-1.551019	3.541613	5.937631
H	-2.222387	3.423450	4.303970
C	-0.080773	3.211225	4.397167
H	-0.007773	2.711022	3.426642
H	0.090360	4.280516	4.219427
C	2.206718	2.340863	4.564363
H	2.997657	2.279112	5.314188
H	2.463141	3.144507	3.863118
C	2.123992	1.016493	3.823416
H	1.392961	1.049912	3.010696
H	3.095610	0.800622	3.365237
C	1.411504	-1.336735	4.104465
H	1.621211	-2.148102	4.807684
H	2.038319	-1.513266	3.223973
C	-0.052662	-1.351295	3.705257
H	-0.248950	-0.564143	2.972442
H	-0.299369	-2.308586	3.230196
C	-2.263907	-0.657146	4.472194
H	-2.941731	-0.814609	5.316002
H	-2.643663	-1.245740	3.628250
C	-2.264297	0.820306	4.122045
H	-1.646333	1.030794	3.245799
H	-3.284860	1.130970	3.873611
C	0.896953	2.628513	7.734015
C	1.202575	3.468049	6.491270
H	2.229836	3.846889	6.492341
H	0.516748	4.321338	6.511723
N	1.320576	3.154909	8.930743
H	0.942290	2.649069	9.719485
C	3.800164	2.861937	8.711862
C	4.517174	3.205937	7.551606
C	5.450365	2.190309	7.004185
C	4.807433	0.698471	8.869594
C	5.514052	0.956747	7.711843
H	6.155425	0.173627	7.315886
O	6.105058	2.405509	5.950242
N	3.981043	1.648154	9.372996
C	3.324877	1.406427	10.655612
H	3.000433	2.348629	11.088847
H	2.457732	0.744816	10.552531
H	4.025766	0.949002	11.354354
C	2.652534	3.743498	9.113275
H	2.692882	4.089747	10.150877
H	2.741503	4.635755	8.489664
O	4.341321	4.313982	6.932768
N	-1.744053	1.586763	5.263233
N	1.745933	-0.063146	4.755241
H	2.526064	-0.200615	5.393923
C	-0.980708	-2.331226	5.714660
H	-0.716174	-3.239058	5.153574
H	-2.008294	-2.485705	6.065275
C	-0.174596	-2.161169	6.978193
O	-0.015810	-1.010315	7.462022
C	4.948122	-0.607628	9.591460
H	5.522178	-1.298096	8.971199
H	5.478145	-0.499010	10.545312
H	3.979186	-1.070658	9.806555
N	-0.921789	-1.122342	4.879192
H	-2.435762	1.551040	6.008703
In	0.125985	0.670569	6.173570
O	1.879524	1.255875	7.275414
H	2.175077	0.822286	8.080053
C	0.476936	-4.556323	7.020244
H	-0.475776	-4.912592	6.622749
H	0.745702	-5.212806	7.851701
N	0.303697	-3.215909	7.604597

H	0.758195	-3.014912	8.485915
C	1.447481	-4.585172	5.878646
C	0.941604	-4.882295	4.599426
C	1.908944	-4.878717	3.471430
C	3.666742	-4.213333	5.084317
C	3.243957	-4.515801	3.804481
H	3.969058	-4.454450	2.997870
C	3.231162	-3.952612	7.464260
H	3.084399	-2.889744	7.683902
H	2.690258	-4.555258	8.192076
H	4.286623	-4.187891	7.577253
O	-0.300381	-5.102864	4.384770
O	1.543917	-5.148929	2.300817
C	5.084528	-3.802124	5.346098
H	5.144257	-2.836360	5.859513
H	5.625723	-4.536666	5.953381
H	5.608932	-3.710892	4.393879
N	2.788604	-4.278236	6.113011

pbe-gd3bj_L2_TS(II).log

SCF (wB97x) = -2057.64434260
E(SCF)+ZPE(0 K)= -2056.907050
H(298 K)= -2056.864925
G(298 K)= -2056.978146
Lowest Frequency = -273.5800cm-1

N	0.046356	1.574574	4.881530
C	-2.124328	1.533208	3.755825
H	-2.623098	1.989637	4.614976
H	-2.667672	1.850055	2.855885
C	-0.681557	2.027798	3.694193
H	-0.170530	1.652314	2.801187
H	-0.682384	3.122359	3.624990
C	1.504765	1.760558	4.870245
H	1.817758	1.930741	5.899269
H	1.773719	2.650857	4.288275
C	2.252165	0.566021	4.291764
H	1.966344	0.452637	3.241880
H	3.327568	0.774243	4.315820
C	2.133305	-1.828070	4.007368
H	2.202836	-2.756196	4.584503
H	3.078350	-1.720182	3.456414
C	0.975927	-1.953546	3.045602
H	0.908124	-1.093998	2.373665
H	1.126062	-2.837927	2.414368
C	-1.466042	-2.056628	2.953528
H	-2.248248	-2.603839	3.481854
H	-1.265504	-2.577291	2.009854
C	-1.914845	-0.636393	2.671978
H	-1.130200	-0.097907	2.132932
H	-2.802071	-0.642538	2.024526
N	-2.187332	0.071315	3.927393
C	-3.431988	-0.394914	4.569331
H	-3.985946	-1.047912	3.882996
H	-4.076986	0.455959	4.811574
C	-3.121408	-1.202079	5.827341
O	-2.089681	-1.977313	5.830043
N	-0.267503	-2.051887	3.809031
H	-0.260865	-2.925665	4.329478
In	-0.531810	-0.457110	5.539261
O	-2.204908	0.178257	6.725351
H	-2.435215	1.108383	6.820008
C	-5.307716	-0.684819	6.874294
H	-4.932527	0.322520	7.082716
H	-5.764224	-1.063622	7.794276
N	-4.183375	-1.582402	6.585562
H	-3.883491	-2.149030	7.367215
C	-6.325629	-0.538645	5.782656
C	-6.441128	0.703142	5.131453
C	-7.479778	0.819172	4.079316
C	-8.101553	-1.534582	4.535422
C	-8.260133	-0.347246	3.845535
H	-9.026306	-0.298523	3.076362
C	-7.011255	-2.834276	6.294085
H	-7.455191	-2.691523	7.285165
H	-7.512018	-3.665998	5.806906
H	-5.956824	-3.087284	6.401483
O	-5.670008	1.694734	5.387553
O	-7.643927	1.889552	3.436465
C	-8.985908	-2.709997	4.240236
H	-8.420921	-3.575974	3.877085
H	-9.559302	-3.029794	5.117039
H	-9.697024	-2.429659	3.461550
N	-7.146905	-1.625035	5.489322
N	1.953715	-0.718615	4.963570
O	3.820966	1.024662	6.730368
C	3.233361	0.010599	7.099682
N	3.178317	-0.294431	8.428503
H	3.625463	0.435848	8.970211
C	2.095953	-0.964977	9.152349
H	1.684251	-1.761624	8.532336
H	2.523087	-1.424660	10.042590
C	1.024412	0.052377	9.452270
C	0.377767	0.558576	8.337015
C	-0.351478	1.813315	8.443486
C	0.060665	1.677781	10.879683
C	-0.516577	2.277316	9.779654
H	-1.105372	3.175903	9.934205
O	0.619434	-0.032543	7.193128
O	-0.771669	2.452502	7.436043
C	-0.167210	2.230391	12.253931
H	-0.805299	3.111706	12.179473
H	-0.670248	1.509604	12.907463
H	0.766970	2.532232	12.738325
C	1.517234	-0.058212	11.844357
H	1.301467	-1.127972	11.847041
H	2.598332	0.098586	11.800406
H	1.147600	0.360775	12.774692
N	0.853330	0.584055	10.711801
C	2.827875	-1.062775	6.109686
H	2.365836	-1.904828	6.624653
H	3.796994	-1.413621	5.716965
H	-0.311068	2.052538	5.730062

pbe-gd3bj_L2_TS(I).log

SCF (wB97x) = -2057.65221527
E(SCF)+ZPE(0 K)= -2056.914792
H(298 K)= -2056.873134
G(298 K)= -2056.981736
Lowest Frequency = -234.4677cm-1

O	0.457900	4.211498	6.792327
N	1.118046	1.751806	4.630063
C	-1.162385	2.725887	4.503889
H	-0.992467	3.363895	5.373734
H	-1.858350	3.227602	3.821810
C	0.163318	2.503431	3.794277
H	0.002691	1.941789	2.869466
H	0.590651	3.474150	3.509457
C	2.119121	1.052423	3.795748
H	2.926763	0.732999	4.459438
H	2.548082	1.731270	3.046673
C	1.544046	-0.175200	3.117628
H	0.759227	0.095210	2.404714
H	2.336152	-0.669078	2.542514
C	0.148983	-2.153097	3.482709
H	0.272609	-3.072065	4.069107
H	0.508059	-2.362920	2.467330
C	-1.303315	-1.733550	3.410982
H	-1.410908	-0.869998	2.750705
H	-1.899082	-2.541653	2.962679
C	-3.063189	-0.551805	4.549296
H	-3.571238	-0.499364	5.513498
H	-3.745107	-1.035042	3.833913
C	-2.721783	0.847488	4.068451
H	-2.304147	0.853762	3.057977
H	-3.639507	1.446390	4.032744
C	1.089529	3.109789	6.865062

C	1.835793	2.601989	5.604983
H	2.695228	2.018485	5.924975
H	2.202646	3.504255	5.096711
N	1.971947	3.045126	8.000577
H	1.589953	3.612349	8.745777
C	1.994799	0.778706	9.011838
C	1.410177	-0.065928	8.081888
C	0.146175	-0.660736	8.436804
C	0.535377	0.009125	10.737057
C	-0.220616	-0.662912	9.788919
H	-1.137257	-1.151065	10.100115
O	-0.620221	-1.027118	7.464703
N	1.599846	0.750247	10.334251
C	2.352438	1.561409	11.291462
H	3.421624	1.412109	11.139809
H	2.108876	2.620734	11.173138
H	2.109914	1.259986	12.305611
C	2.797504	1.929756	8.459749
H	3.507040	2.336535	9.182542
H	3.389443	1.546681	7.627228
O	1.767933	-0.101984	6.830498
N	-1.740879	1.450205	4.974766
N	0.972973	-1.103770	4.106238
H	1.736476	-1.595749	4.596374
C	-2.150855	-2.641856	5.441917
H	-1.255281	-3.265246	5.407152
H	-2.925336	-3.185673	4.871303
C	-2.695361	-2.569016	6.860195
O	-3.519455	-1.728577	7.221619
C	0.160574	-0.049450	12.185913
H	-0.731005	-0.668314	12.294671
H	0.952158	-0.499438	12.795390
H	-0.062561	0.939579	12.598174
N	-1.852739	-1.377055	4.737927
H	-2.198346	1.646139	5.861574
In	-0.069511	0.076558	5.735496
O	-0.088590	1.895424	7.029879
H	-0.417273	1.978896	7.928003
C	-1.364701	-4.653960	7.494898
H	-1.493885	-5.114903	6.513101
H	-1.610984	-5.418419	8.237324
N	-2.357459	-3.589431	7.678620
H	-2.839713	-3.552369	8.564875
C	0.073248	-4.223873	7.558083
C	0.800149	-4.183110	6.357537
C	2.097222	-3.481288	6.391564
C	1.895650	-3.389486	8.847169
C	2.610206	-3.197367	7.680877
H	3.596081	-2.747311	7.751582
C	-0.153771	-4.086635	9.984895
H	-1.127621	-3.604204	9.901340
H	-0.293207	-5.157837	10.159925
H	0.351921	-3.656779	10.844017
O	0.331510	-4.634525	5.250131
O	2.693232	-3.156334	5.321478
C	2.512183	-3.067011	10.174535
H	1.970186	-2.271279	10.696979
H	2.555019	-3.937750	10.837931
H	3.534027	-2.720381	10.012595
N	0.627278	-3.859535	8.776482

pbe_H2O.log

SCF (wB97x) = -76.3780512860
E(SCF)+ZPE(0 K)= -76.356523
H(298 K)= -76.352743
G(298 K)= -76.374813
Lowest Frequency = 1606.6623cm-1

O	-0.210167	1.497763	0.000000
H	0.748980	1.534215	0.000000
H	-0.495980	2.414068	0.000000

pbe_HOPO.log

SCF (wB97x) = -571.494963568
E(SCF)+ZPE(0 K)= -571.293370
H(298 K)= -571.280601
G(298 K)= -571.330430
Lowest Frequency = 80.9659cm-1

C	-2.172321	-2.336715	-0.117852
C	-0.809929	-2.241695	-0.195551
C	-0.111380	-0.976890	-0.135827
C	-0.952527	0.156200	0.005519
C	-2.325010	0.040206	0.090914
H	-0.506159	1.144148	0.044879
N	-2.920295	-1.184304	0.048809
C	-4.378737	-1.302386	0.107854
H	-4.634157	-2.233243	0.613049
H	-4.791646	-0.479060	0.685567
H	-4.807987	-1.283594	-0.898873
C	-3.181286	1.262622	0.225544
H	-2.551731	2.150416	0.155325
H	-3.939235	1.318664	-0.561897
H	-3.697911	1.294682	1.191074
C	-2.855217	-3.673600	-0.202850
H	-2.135206	-4.373527	-0.642466
H	-3.701451	-3.615144	-0.895809
O	-0.018620	-3.324950	-0.342027
H	0.880695	-2.942986	-0.372577
O	1.144810	-0.986777	-0.217854
N	-3.361172	-4.101006	1.103392
H	-3.864991	-4.975448	1.005219
H	-2.586885	-4.279656	1.733398

pbe_InOH3.log

SCF (wB97x) = -229.281357172
E(SCF)+ZPE(0 K)= -229.242782
H(298 K)= -229.234704
G(298 K)= -229.274463
Lowest Frequency = 124.1656cm-1

In	0.745292	-0.634756	0.063508
O	0.064718	1.113882	-0.461965
H	-0.879181	1.188339	-0.620932
O	2.649879	-0.881091	0.392113
H	3.217669	-0.115569	0.276486
O	-0.483173	-2.134295	0.261652
H	-0.108862	-2.979497	0.521108

pbe_L1_III.log

SCF (wB97x) = -2057.54954444
E(SCF)+ZPE(0 K)= -2056.814468
H(298 K)= -2056.769995
G(298 K)= -2056.893241
Lowest Frequency = 8.4129cm-1

O	-1.617234	2.755594	5.641934
N	-0.051715	2.322753	3.464367
C	-2.329733	1.793142	2.622504
H	-2.722239	2.722960	3.041501
H	-2.896908	1.593584	1.705283
C	-0.860273	1.963041	2.280952
H	-0.457993	1.040825	1.855050
H	-0.761814	2.732711	1.503432
C	1.390919	2.113559	3.202392
H	1.942751	2.637629	3.987904
H	1.679518	2.559794	2.242012
C	1.774033	0.644489	3.233143
H	1.333822	0.091993	2.398282
H	2.862829	0.562212	3.126341
C	1.417324	-1.402993	4.585348
H	1.511998	-1.657361	5.645228
H	2.311756	-1.775906	4.072179
C	0.174784	-2.061507	4.008577
H	0.098620	-1.862443	2.934892
H	0.233750	-3.148855	4.129228

C	-2.273793	-1.749807	3.921302
H	-3.094039	-1.850353	4.635704
H	-2.231021	-2.691439	3.361823
C	-2.536884	-0.611644	2.946247
H	-1.758910	-0.598078	2.178373
H	-3.492031	-0.777092	2.432922
C	-1.172845	3.796542	5.103157
C	-0.298252	3.712567	3.876351
H	0.637969	4.229465	4.149246
H	-0.752838	4.285797	3.059089
N	-1.450885	4.983941	5.616094
H	-2.009958	4.955108	6.459473
C	0.087499	6.842735	6.179684
C	1.402859	6.359964	6.081951
C	2.406443	6.929813	7.014515
C	0.590698	8.318860	7.981502
C	1.908171	7.899768	7.929809
H	2.610879	8.337747	8.633550
O	3.609001	6.564324	6.986047
N	-0.312159	7.791136	7.121019
C	-1.701039	8.238352	7.127871
H	-1.928090	8.827818	6.234116
H	-2.382497	7.386246	7.178551
H	-1.887218	8.856837	8.000516
C	-0.911028	6.288770	5.208157
H	-1.757345	6.954394	5.031852
H	-0.401090	6.153930	4.253558
O	1.740056	5.468741	5.227106
N	-2.546982	0.713038	3.613400
N	1.312195	0.058600	4.491095
H	1.824550	0.469705	5.269139
C	-3.817967	0.917730	4.336274
H	-4.682400	0.538291	3.762973
H	-3.972861	1.994021	4.474272
C	-3.753813	0.349842	5.736245
O	-2.646231	0.203386	6.300816
C	0.167468	9.355522	8.982273
H	1.039700	9.661864	9.561898
H	-0.246092	10.251123	8.506089
H	-0.579961	8.974870	9.687525
N	-1.025494	-1.521397	4.669841
H	-1.113793	-1.963466	5.581143
In	-0.808557	0.758361	5.195117
O	0.267168	0.752566	6.899632
H	-0.194195	1.082761	7.672708
C	-6.241236	0.070147	5.813758
H	-6.330845	0.902655	5.113633
H	-6.907232	0.290089	6.652205
N	-4.879169	0.061576	6.370973
H	-4.755142	-0.251992	7.324792
C	-6.630239	-1.160633	5.049665
C	-6.723131	-1.052963	3.649144
C	-7.141589	-2.267044	2.902068
C	-7.274939	-3.459358	5.070651
C	-7.392173	-3.422820	3.693786
H	-7.687336	-4.333783	3.180056
C	-6.718759	-2.386632	7.186197
H	-5.664637	-2.554505	7.433287
H	-7.057377	-1.460572	7.649108
H	-7.306578	-3.192237	7.618356
O	-6.453267	0.029265	3.019284
O	-7.253732	-2.256881	1.650183
C	-7.538331	-4.731701	5.822231
H	-6.676537	-5.048194	6.419670
H	-8.398379	-4.646761	6.496364
H	-7.758840	-5.525736	5.107047
N	-6.916980	-2.338847	5.741703

pbe_L1_II.log

SCF (wB97x) = -2057.53430155
E(SCF)+ZPE(0 K)= -2056.799077
H(298 K)= -2056.755058
G(298 K)= -2056.873667
Lowest Frequency = 13.3146cm-1

O	-0.459454	4.391630	6.398192
N	0.561611	2.079383	4.505413
C	-1.925743	2.314375	4.424060
H	-1.896200	2.865990	5.365774
H	-2.734886	2.718568	3.798306
C	-0.600734	2.525808	3.713692
H	-0.589454	1.997041	2.755413
H	-0.518690	3.594594	3.472258
C	1.585467	1.482238	3.618317
H	2.541937	1.505438	4.145642
H	1.707292	2.076572	2.700851
C	1.303276	0.038263	3.258292
H	0.382971	-0.060195	2.675326
H	2.120748	-0.329696	2.622830
C	1.048203	-2.196046	4.262033
H	1.208507	-2.693129	5.224375
H	1.799356	-2.572501	3.555062
C	-0.350703	-2.489604	3.748566
H	-0.467881	-2.093133	2.735266
H	-0.509003	-3.572721	3.683613
C	-2.553674	-1.408826	3.929913
H	-3.422425	-1.622012	4.557504
H	-2.709177	-1.976110	3.003986
C	-2.494624	0.074349	3.583743
H	-1.702285	0.242775	2.850096
H	-3.439978	0.383314	3.115351
C	0.587364	3.751677	6.482997
C	1.243899	3.174774	5.236963
H	2.229694	2.805551	5.498723
H	1.370739	4.035005	4.559367
N	1.269360	3.715036	7.657276
H	0.751365	4.165461	8.399831
C	2.153706	1.668915	8.743930
C	1.694223	0.669475	7.906500
C	1.033982	-0.487094	8.463894
C	1.717656	0.324956	10.660685
C	1.132838	-0.647289	9.854790
H	0.710033	-1.526465	10.330442
O	0.377414	-1.240952	7.650526
N	2.183115	1.477473	10.111450
C	2.752635	2.536676	10.946103
H	3.784540	2.735659	10.648668
H	2.162534	3.451124	10.854023
H	2.749400	2.234218	11.987926
C	2.467341	2.990295	8.083693
H	3.035959	3.660562	8.729937
H	3.092679	2.793691	7.212020
O	1.686432	0.793157	6.619992
N	-2.215224	0.901397	4.770989
N	1.190717	-0.757875	4.482644
H	2.002074	-0.571129	5.070424
C	-3.387615	0.901086	5.668067
H	-4.316229	1.055258	5.098511
H	-3.267829	1.740601	6.361038
C	-3.447517	-0.312158	6.568748
O	-2.409632	-0.903842	6.905922
C	1.815482	0.104076	12.141565
H	1.399785	-0.875718	12.380526
H	2.852651	0.118362	12.493221
H	1.253569	0.850710	12.712557
N	-1.332820	-1.842891	4.619270
H	-1.583466	-2.464923	5.379318
In	-0.350143	0.102470	5.999196
O	-1.030102	1.500907	7.336843
H	-1.421182	1.053158	8.089327
C	-5.959216	-0.191666	6.644723
H	-5.928195	0.895598	6.548877
H	-6.631597	-0.413133	7.479151
N	-4.637385	-0.685602	7.049729
H	-4.596922	-1.455331	7.703412
C	-6.463320	-0.704165	5.326586
C	-6.593166	0.220689	4.271886
C	-7.091255	-0.300150	2.973180
C	-7.232997	-2.544672	4.013102

C	-7.372202	-1.694644	2.931580
H	-7.718270	-2.114663	1.990797
C	-6.634579	-2.944517	6.339854
H	-5.600981	-3.299929	6.411086
H	-6.906655	-2.435433	7.263766
H	-7.291879	-3.805093	6.243189
O	-6.284642	1.456909	4.392611
O	-7.238224	0.458295	1.980494
C	-7.548511	-4.006069	3.879801
H	-6.703376	-4.641820	4.164937
H	-8.409546	-4.304283	4.489321
H	-7.793629	-4.221835	2.838556
N	-6.807817	-2.050682	5.199998

pbe_L1_I.log

SCF (wB97x) = -2057.52585036
E(SCF)+ZPE(0 K)= -2056.789753
H(298 K)= -2056.746134
G(298 K)= -2056.861900
Lowest Frequency = 15.0910cm-1

O	-1.464821	3.741024	6.893310
N	0.585055	2.742724	4.896315
C	-1.697595	2.565374	3.826963
H	-2.170650	2.659232	4.804368
H	-2.328677	3.115211	3.105450
C	-0.340175	3.253005	3.864632
H	0.151627	3.202008	2.887893
H	-0.538133	4.318713	4.036210
C	1.932358	2.483033	4.355276
H	2.600717	2.336282	5.206155
H	2.303890	3.348572	3.783201
C	2.025821	1.256902	3.476383
H	1.398386	1.357510	2.593504
H	3.060617	1.170957	3.118246
C	1.856888	-1.191802	3.346502
H	1.535266	-2.053394	3.944164
H	2.934736	-1.282412	3.152338
C	1.141429	-1.205400	1.999048
H	1.404332	-0.319043	1.406891
H	1.588476	-2.051995	1.453625
C	-1.140078	-0.384693	1.434051
H	-2.141652	-0.817673	1.322803
H	-0.788470	-0.228667	0.405188
C	-1.244987	1.020245	2.046813
H	-0.281206	1.516167	1.944686
H	-1.947929	1.598801	1.418228
C	-0.262048	3.599615	7.129455
C	0.759731	3.689497	6.015868
H	1.756699	3.553439	6.422401
H	0.703313	4.726327	5.646631
N	0.174018	3.492749	8.406555
H	-0.589586	3.501819	9.071208
C	1.368073	1.515612	9.205385
C	1.225357	0.691949	8.104228
C	0.784276	-0.670429	8.286006
C	1.009808	-0.314555	10.690618
C	0.742884	-1.144398	9.610741
H	0.469785	-2.176714	9.804199
O	0.447780	-1.315908	7.239655
N	1.290621	0.999610	10.488075
C	1.536631	1.899797	11.615353
H	2.493312	2.409488	11.486530
H	0.735001	2.638008	11.701235
H	1.579249	1.333821	12.540009
C	1.446398	2.996526	8.933214
H	1.688363	3.584053	9.818668
H	2.235806	3.187292	8.205763
O	1.342996	1.118649	6.883085
N	-1.671589	1.134288	3.463376
N	1.644955	0.021731	4.186584
H	2.307309	-0.065386	4.959365
C	-3.047419	0.595865	3.514501
H	-3.027485	-0.379555	3.040247
H	-3.702282	1.236413	2.898781
C	-3.756955	0.507887	4.862980
O	-4.207469	1.521594	5.397148
C	0.971374	-0.868675	12.084336
H	0.717813	-1.928470	12.035535
H	1.939797	-0.783068	12.589053
H	0.219240	-0.374265	12.707672
N	-0.300306	-1.365425	2.079177
H	-0.599095	-1.533212	3.041133
In	-0.134200	0.145043	5.587516
O	-1.658163	0.759831	6.714571
H	-1.850127	1.699721	6.705561
C	-3.722686	-2.035347	4.857625
H	-3.310495	-1.919673	3.856971
H	-4.664124	-2.577908	4.734633
N	-4.022242	-0.714011	5.377003
H	-4.527358	-0.667970	6.251237
C	-2.726873	-2.887651	5.608160
C	-1.378726	-2.668647	5.385968
C	-0.417987	-3.728924	5.698923
C	-2.303932	-4.927022	6.764609
C	-0.969544	-4.818043	6.444638
H	-0.297185	-5.611484	6.757783
C	-4.603683	-4.090370	6.654997
H	-4.733610	-4.673660	7.563343
H	-5.027494	-3.103946	6.840759
H	-5.156446	-4.575592	5.843915
O	-0.987522	-1.580923	4.753732
O	0.769799	-3.692021	5.305015
C	-2.807543	-6.094082	7.561266
H	-3.192853	-5.787924	8.540614
H	-3.605187	-6.635884	7.042857
H	-1.984286	-6.788896	7.733459
N	-3.181334	-3.975431	6.341723

pbe_L1_IVac.log

SCF (wB97x) = -1562.46524843
E(SCF)+ZPE(0 K)= -1561.908562
H(298 K)= -1561.875306
G(298 K)= -1561.970898
Lowest Frequency = 18.4160cm-1

O	-0.033404	-0.550199	6.133359
N	-0.278748	1.718348	4.792402
C	-2.053326	2.002366	3.078744
H	-2.861389	1.792657	3.785435
H	-2.456643	2.720084	2.351968
C	-0.882270	2.636323	3.808804
H	-0.100594	2.917587	3.098130
H	-1.216207	3.562474	4.295101
C	1.094143	2.149821	5.150227
H	1.335205	1.736317	6.133477
H	1.135582	3.242460	5.239569
C	2.125988	1.661820	4.150641
H	1.973030	2.121212	3.170366
H	3.124577	1.962830	4.489210
C	2.773681	-0.340673	2.863101
H	2.901498	-1.412355	3.039317
H	3.768556	0.113714	2.783132
C	1.998183	-0.126070	1.573253
H	1.905181	0.940637	1.345472
H	2.543890	-0.584147	0.739042
C	-0.286097	-0.276663	0.658663
H	-0.965062	-1.109891	0.458707
H	0.230426	-0.059847	-0.284092
C	-1.062969	0.956163	1.102377
H	-0.382953	1.808599	1.188090
H	-1.813716	1.221780	0.345798
C	-0.666719	0.338285	6.764780
C	-1.080389	1.597546	6.015658
H	-0.973699	2.492309	6.642067
H	-2.140431	1.498739	5.761731
C	-1.646528	0.731999	10.298559
C	-1.185205	-0.524922	10.703542

C	-1.098121	-0.797427	12.151569
C	-2.005290	1.468572	12.545176
C	-1.527270	0.259894	13.004160
H	-1.475547	0.099104	14.077187
O	-0.672855	-1.897639	12.583917
N	-2.076196	1.695275	11.208506
C	-2.581937	2.968628	10.701515
H	-1.763902	3.615140	10.367124
H	-3.272157	2.802007	9.873525
H	-3.131537	3.485281	11.482632
C	-1.733197	1.130243	8.838230
H	-2.782431	1.150236	8.501107
H	-1.340285	2.145013	8.705333
O	-0.818491	-1.454597	9.867721
N	-1.695522	0.734978	2.412960
N	2.027113	0.203435	4.008681
H	2.378245	-0.227895	4.859860
C	-2.892013	-0.109527	2.304676
H	-2.720834	-0.902601	1.570092
H	-3.761239	0.465429	1.962124
O	-4.354977	-1.212562	3.825235
C	-3.214746	-0.797822	3.633038
O	-2.235885	-0.911270	4.451279
C	-2.441730	2.530890	13.511072
H	-2.247393	2.184002	14.527141
H	-1.898887	3.471264	13.368408
H	-3.513471	2.747596	13.436226
N	0.651776	-0.691202	1.704585
H	0.719790	-1.706755	1.739820
In	-0.189781	-0.494782	3.917920
O	0.320770	-2.453967	3.765602
H	-0.006104	-3.014868	4.471203
N	-0.971326	0.197863	8.029610
H	-0.772463	-0.705395	8.600635

pbe_L1_IVhc.log

SCF (wB97x) = -1562.44138600
E(SCF)+ZPE(0 K)= -1561.882651
H(298 K)= -1561.849839
G(298 K)= -1561.941623
Lowest Frequency = 32.9830cm-1

O	-1.179442	4.281358	6.779788
N	0.722275	2.832880	4.725845
C	-1.659794	2.730918	3.983054
H	-1.971595	2.952520	5.005885
H	-2.366347	3.201279	3.282374
C	-0.281728	3.336046	3.773948
H	0.073795	3.142617	2.756486
H	-0.383435	4.427866	3.855699
C	2.024529	2.637366	4.060167
H	2.795590	2.606583	4.833759
H	2.265283	3.483295	3.397431
C	2.120239	1.343649	3.278531
H	1.413163	1.323808	2.444554
H	3.125686	1.279042	2.838448
C	2.110101	-1.096506	3.531553
H	2.116418	-1.850702	4.325524
H	3.086911	-1.122459	3.029728
C	0.997410	-1.395963	2.539574
H	1.063759	-0.715545	1.684496
H	1.111971	-2.412422	2.142533
C	-1.361792	-0.695912	2.339565
H	-2.299633	-1.183164	2.616889
H	-1.185747	-0.951608	1.286463
C	-1.498951	0.818594	2.449413
H	-0.590382	1.291625	2.069681
H	-2.328174	1.159785	1.811526
C	-0.067465	3.797441	6.986340
C	0.966833	3.734248	5.871833
H	1.923451	3.448671	6.296830
H	1.055587	4.772616	5.509442
N	0.315665	3.488617	8.252280
H	-0.444716	3.603322	8.908926

C	1.331642	1.342845	8.976138
C	1.269936	0.590735	7.817798
C	0.790017	-0.770817	7.867508
C	0.820700	-0.569984	10.300095
C	0.640676	-1.328693	9.147472
H	0.339871	-2.366150	9.254345
O	0.509639	-1.338541	6.747438
N	1.124372	0.751215	10.207973
C	1.269934	1.577916	11.406694
H	2.278072	1.995552	11.456822
H	0.538238	2.388829	11.399686
H	1.102978	0.979391	12.296000
C	1.507608	2.830548	8.788570
H	1.771143	3.344052	9.714528
H	2.338023	2.988333	8.099129
O	1.496980	1.095879	6.651569
N	-1.714196	1.257597	3.838727
N	1.865267	0.201278	4.159480
H	2.437332	0.302408	4.997090
C	-3.033269	0.787815	4.308029
H	-3.779235	0.877793	3.507929
H	-3.344548	1.431893	5.134272
O	-4.164350	-1.208203	4.903134
C	-3.063000	-0.640381	4.867553
O	-1.967407	-1.116256	5.295768
C	0.666498	-1.214617	11.646515
H	0.438288	-2.272192	11.507059
H	1.583124	-1.149674	12.242610
H	-0.148769	-0.774013	12.230024
N	-0.288516	-1.197926	3.198424
H	-0.592057	-2.048891	3.655960
In	-0.157053	0.304983	5.344186
O	-1.343059	1.184862	6.777967
H	-1.979743	0.528887	7.067769

pbe_L1.log

SCF (wB97x) = -1980.95615120
E(SCF)+ZPE(0 K)= -1980.215851
H(298 K)= -1980.172967
G(298 K)= -1980.293429
Lowest Frequency = 10.5527cm-1

O	2.004166	0.394409	6.737086
N	0.533730	1.948199	4.999739
C	-1.921889	1.564883	4.523280
H	-2.253103	1.744995	5.551313
H	-2.715926	1.994839	3.883509
C	-0.646815	2.358703	4.249062
H	-0.394936	2.286413	3.186718
H	-0.888069	3.425199	4.428084
C	1.743586	2.580777	4.468727
H	2.505681	2.583986	5.255504
H	1.555459	3.635514	4.200447
C	2.306599	1.832555	3.269883
H	1.530341	1.763801	2.491572
H	3.120428	2.429398	2.833746
C	2.761516	-0.513968	2.666472
H	3.220745	-1.413059	3.095987
H	3.390246	-0.210085	1.818783
C	1.377770	-0.885619	2.128173
H	0.946951	-0.030247	1.575853
H	1.503578	-1.690597	1.392074
C	-0.882579	-1.544518	2.775957
H	-1.336277	-2.358757	3.357326
H	-0.904017	-1.871890	1.729905
C	-1.720050	-0.276559	2.930553
H	-1.238529	0.530169	2.368816
H	-2.723712	-0.412375	2.488728
C	1.352197	1.305960	7.239293
C	0.395850	2.166722	6.418978
H	0.519516	3.231556	6.690929
H	-0.614074	1.881002	6.732246
N	1.460703	1.576686	8.560327
H	2.052764	0.935320	9.072642

C	-0.340039	1.749100	10.230292
C	0.090958	0.736699	11.039806
C	-0.802154	-0.058807	11.847812
C	-2.585739	1.354451	10.939159
C	-2.169756	0.303876	11.730307
H	-2.912668	-0.254951	12.289111
O	-0.311532	-0.975081	12.553896
N	-1.683002	2.078209	10.211758
C	-2.108922	3.249530	9.443370
H	-1.506908	4.117525	9.717452
H	-2.030206	3.064839	8.369543
H	-3.143911	3.480871	9.671013
C	0.672137	2.483473	9.373881
H	0.203491	3.225504	8.733222
H	1.355245	3.031596	10.033051
O	1.398625	0.409735	11.117356
H	1.416021	-0.337377	11.747331
N	-1.810119	0.123506	4.335957
N	2.806473	0.529037	3.678127
H	2.332648	0.244401	4.531707
C	-2.894809	-0.580153	5.005917
H	-2.859832	-1.640685	4.734324
H	-3.885904	-0.195379	4.711961
O	-3.723423	0.106571	7.150864
C	-2.834515	-0.469848	6.525111
N	-1.775109	-1.006436	7.172892
H	-1.810605	-0.883578	8.176451
C	-0.722860	-1.851623	6.630761
H	0.251523	-1.384331	6.794304
H	-0.853901	-1.904829	5.551924
C	-0.768689	-3.255299	7.178290
C	-1.562538	-4.193427	6.581812
C	-0.043109	-4.880073	8.773106
C	-1.642814	-5.559918	7.046267
C	-0.833273	-5.841661	8.176870
H	-0.832294	-6.846043	8.586510
O	-2.322214	-3.900495	5.508570
H	-2.753781	-4.750664	5.292822
O	-2.393076	-6.356141	6.427205
N	-0.018925	-3.605053	8.288504
C	0.839473	-2.588263	8.898349
H	1.710652	-2.386968	8.269119
H	0.282670	-1.663417	9.049621
H	1.178954	-2.927759	9.871505
N	0.505112	-1.359604	3.193100
H	0.499603	-0.674105	3.947745
C	-4.041594	1.706380	10.871141
H	-4.609140	0.974790	11.447260
H	-4.246106	2.694222	11.297620
H	-4.417044	1.691676	9.843312
C	0.799163	-5.236170	9.961317
H	0.678835	-6.298681	10.175125
H	1.862227	-5.046766	9.782732
H	0.500572	-4.680480	10.856656

pbe_L1_TS(III).log

SCF (wB97x) = -2057.51631873
E(SCF)+ZPE(0 K)= -2056.780374
H(298 K)= -2056.738063
G(298 K)= -2056.853677
Lowest Frequency = -300.6737cm-1

O	-1.600059	2.341323	5.274201
N	0.021276	2.431049	3.003155
C	-2.395903	2.256850	2.407888
H	-2.679474	2.947988	3.203139
H	-3.095118	2.381927	1.572148
C	-0.985171	2.598064	1.936212
H	-0.693469	1.957224	1.098750
H	-0.985849	3.627944	1.555893
C	1.377937	2.205998	2.459028
H	2.092751	2.474677	3.239823
H	1.565544	2.854846	1.594503
C	1.607542	0.754107	2.083820
H	0.939599	0.440818	1.275291
H	2.633412	0.634050	1.715137
C	1.432674	-1.540366	2.972479
H	1.600667	-2.062488	3.919447
H	2.270159	-1.780040	2.306841
C	0.122568	-1.996087	2.351139
H	-0.008756	-1.549284	1.360804
H	0.129425	-3.082754	2.212564
C	-2.294302	-1.530104	2.496291
H	-3.102085	-1.792391	3.185893
H	-2.321932	-2.270765	1.688802
C	-2.544524	-0.148357	1.913479
H	-1.780106	0.093136	1.168242
H	-3.515457	-0.132689	1.403575
C	-0.473812	2.964276	5.355280
C	0.017596	3.516774	4.014996
H	1.006943	3.972643	4.085129
H	-0.691000	4.300032	3.722869
N	-0.282280	3.779688	6.459009
H	-0.833028	3.428886	7.233046
C	1.312551	3.876451	8.321600
C	1.639399	2.562354	8.675990
C	1.924573	2.282214	10.098241
C	1.416247	4.663587	10.569440
C	1.796515	3.398772	10.974828
H	1.999595	3.237988	12.030047
O	2.257990	1.135186	10.491319
N	1.160269	4.894855	9.258692
C	0.728206	6.213451	8.802776
H	-0.094908	6.112235	8.093445
H	0.369689	6.797092	9.646111
H	1.550587	6.756987	8.326658
C	1.078296	4.168876	6.870134
H	1.263228	5.217158	6.622750
H	1.798937	3.567703	6.305526
O	1.720917	1.609733	7.806788
N	-2.500574	0.885513	2.969537
N	1.366765	-0.101636	3.257069
H	2.044861	0.143008	3.975003
C	-3.654789	0.753498	3.883500
H	-4.492913	0.248802	3.381855
H	-3.970557	1.747503	4.208723
C	-3.337242	-0.051809	5.133032
O	-2.158438	-0.385617	5.425219
C	1.287008	5.776634	11.568320
H	1.607672	5.413503	12.546092
H	1.908411	6.640720	11.310768
H	0.253147	6.126491	11.669916
N	-0.998066	-1.572363	3.200971
H	-1.081028	-2.220434	3.979567
In	-0.633273	0.558470	4.242276
O	0.660424	1.590919	5.481291
H	1.024477	1.550575	6.427602
C	-5.772654	-0.275540	5.716047
H	-5.925048	0.336657	4.826350
H	-6.189046	0.274750	6.560489
N	-4.325685	-0.399323	5.936935
H	-4.033349	-0.933234	6.746226
C	-6.415106	-1.609965	5.489295
C	-6.209059	-2.190590	4.227286
C	-6.822050	-3.519031	3.983892
C	-7.713442	-3.432993	6.300507
C	-7.554652	-4.061358	5.077925
H	-8.018351	-5.034552	4.940582
C	-7.316686	-1.518009	7.774345
H	-7.920030	-0.615270	7.639062
H	-7.821484	-2.160043	8.489390
H	-6.348727	-1.244063	8.200651
O	-5.514963	-1.606835	3.324881
O	-6.689740	-4.112898	2.883665
C	-8.517309	-4.088620	7.386570
H	-7.921621	-4.290785	8.283755
H	-9.382554	-3.487299	7.686457
H	-8.891807	-5.045084	7.018425
N	-7.143312	-2.222002	6.507977

pbe_L1_TS(II).log

SCF (wB97x) = -2057.52772566
E(SCF)+ZPE(0 K)= -2056.791187
H(298 K)= -2056.748997
G(298 K)= -2056.861721
Lowest Frequency = -248.2594cm-1

O	-1.935462	3.078855	6.398680
N	0.287406	2.228229	4.428642
C	-1.983655	1.562346	3.638982
H	-2.440185	2.039000	4.506681
H	-2.668915	1.684849	2.787363
C	-0.684340	2.289606	3.333509
H	-0.218277	1.874690	2.434554
H	-0.941295	3.331148	3.082893
C	1.662922	2.136647	3.914275
H	2.344877	2.353800	4.740174
H	1.850800	2.887911	3.129850
C	2.027508	0.767362	3.374929
H	1.383222	0.480234	2.538667
H	3.051700	0.814717	2.978994
C	2.369960	-1.582075	4.000420
H	2.549759	-2.177087	4.901887
H	3.307970	-1.536985	3.431992
C	1.276283	-2.226908	3.164903
H	1.156706	-1.690292	2.218652
H	1.548960	-3.257940	2.912477
C	-1.184796	-2.104484	3.045253
H	-1.997740	-2.602905	3.575295
H	-1.022147	-2.642388	2.103052
C	-1.567755	-0.666207	2.731522
H	-0.754608	-0.177936	2.185463
H	-2.445404	-0.657879	2.069558
C	-0.717088	3.192231	6.578187
C	0.215440	3.351969	5.384775
H	1.221500	3.561816	5.741726
H	-0.136479	4.263184	4.873967
N	-0.252660	3.339712	7.843767
H	-1.008177	3.303117	8.517136
C	1.209726	1.669503	8.879687
C	1.097275	0.715971	7.890458
C	1.033181	-0.685471	8.208857
C	1.392145	-0.027955	10.533311
C	1.219968	-1.010862	9.562248
H	1.219051	-2.049871	9.875096
O	0.803480	-1.499475	7.239321
N	1.366759	1.289195	10.195477
C	1.524289	2.338599	11.204127
H	2.415430	2.933152	10.990812
H	0.644150	2.985000	11.212449
H	1.632515	1.897372	12.189120
C	1.073235	3.100575	8.412851
H	1.230124	3.827756	9.209843
H	1.828404	3.297081	7.648859
O	0.981766	1.057009	6.646380
N	-1.841300	0.118311	3.951613
N	1.929672	-0.251553	4.426251
H	2.485296	0.059950	5.218956
C	-3.084732	-0.351239	4.624015
H	-3.613234	-1.061372	3.975823
H	-3.758578	0.493829	4.802835
C	-2.758812	-1.081567	5.922501
O	-1.783149	-1.917929	5.891483
C	1.599532	-0.427388	11.964302
H	1.600682	-1.515954	12.031132
H	2.556630	-0.069252	12.357824
H	0.803067	-0.055688	12.617438
N	0.010717	-2.164609	3.900203
H	-0.075874	-2.973964	4.506414
In	-0.143651	-0.393062	5.545325
O	-1.825684	0.257738	6.740524
H	-2.139680	1.170916	6.672199
C	-4.917115	-0.469006	7.020405
H	-4.523126	0.550250	7.088213
H	-5.281742	-0.738680	8.017482
N	-3.835679	-1.413545	6.712578
H	-3.523393	-1.957785	7.505469
C	-6.046771	-0.415523	6.030424
C	-6.218821	0.754003	5.264591
C	-7.364624	0.782285	4.321611
C	-7.977277	-1.485302	5.112251
C	-8.191322	-0.375978	4.314386
H	-9.042094	-0.387205	3.637967
C	-6.705934	-2.625135	6.861758
H	-7.008440	-2.363316	7.881575
H	-7.288926	-3.483064	6.538635
H	-5.653089	-2.906990	6.857218
O	-5.419908	1.753421	5.337568
O	-7.579845	1.778204	3.579712
C	-8.915648	-2.656069	5.053448
H	-8.417473	-3.575869	4.725949
H	-9.389482	-2.860239	6.019940
H	-9.707922	-2.437611	4.335325
N	-6.918093	-1.501436	5.954482

pbe_L1_TS(I).log

SCF (wB97x) = -2057.50557858
E(SCF)+ZPE(0 K)= -2056.769718
H(298 K)= -2056.727396
G(298 K)= -2056.839328
Lowest Frequency = -155.1362cm-1

O	-1.816165	3.749129	6.666704
N	0.579472	2.624789	5.249821
C	-1.537589	2.460276	3.897147
H	-2.144477	2.560031	4.796072
H	-2.069049	2.975126	3.080056
C	-0.205457	3.163202	4.115295
H	0.410158	3.127001	3.211252
H	-0.426925	4.223455	4.286901
C	1.996661	2.423999	4.887903
H	2.558692	2.276132	5.815748
H	2.415370	3.319956	4.403622
C	2.241842	1.224485	3.994381
H	1.806667	1.371340	3.006186
H	3.325550	1.124835	3.846105
C	2.036910	-1.229312	3.815358
H	1.655137	-2.074728	4.398772
H	3.132166	-1.307951	3.765606
C	1.493119	-1.299819	2.394451
H	1.794013	-0.414515	1.819353
H	2.027966	-2.140115	1.924560
C	-0.718844	-0.507704	1.601900
H	-1.713746	-0.924676	1.410588
H	-0.283179	-0.348357	0.606057
C	-0.845032	0.894254	2.211816
H	0.138818	1.361511	2.222573
H	-1.460682	1.495701	1.520980
C	-0.725444	3.608618	7.209521
C	0.569750	3.525276	6.419535
H	1.374770	3.211917	7.083123
H	0.806980	4.555291	6.101069
N	-0.619269	3.695803	8.563102
H	-1.520005	3.741224	9.016890
C	0.983695	1.888454	9.320692
C	0.578527	1.006155	8.331053
C	1.288614	-0.239590	8.156069
C	2.572386	0.291653	10.146800
C	2.256865	-0.575543	9.113182
H	2.780980	-1.522649	9.042564
O	0.992077	-0.929392	7.118014
N	1.965790	1.505308	10.223094
C	2.309460	2.437179	11.299567
H	2.584131	3.409115	10.887261
H	1.465918	2.555789	11.984813
H	3.158801	2.061746	11.859550
C	0.496366	3.325859	9.408897

H	0.180234	3.559673	10.429124
H	1.343213	3.996422	9.206077
O	-0.355472	1.240792	7.459121
N	-1.427590	1.019412	3.574452
N	1.695166	-0.004388	4.585841
H	2.168410	-0.146588	5.479569
C	-2.779154	0.423238	3.521638
H	-2.653205	-0.590653	3.156104
H	-3.377818	0.969334	2.774941
C	-3.662721	0.397240	4.788195
O	-4.331608	1.416883	5.094691
C	3.588332	-0.109591	11.174224
H	3.924538	-1.124629	10.958822
H	4.469538	0.540685	11.163073
H	3.174498	-0.103398	12.187858
N	0.058243	-1.502617	2.300137
H	-0.348116	-1.744817	3.204102
In	-0.357613	0.109829	5.619951
O	-2.364163	0.296304	6.079947
H	-2.536487	1.079865	6.611844
C	-3.713725	-2.154056	4.803939
H	-3.106696	-2.155698	3.897284
H	-4.531883	-2.854716	4.605165
N	-4.317558	-0.840423	4.940781
H	-5.021694	-0.759123	5.661682
C	-2.820850	-2.778897	5.861574
C	-1.442569	-2.701334	5.722635
C	-0.599969	-3.722875	6.349749
C	-2.634641	-4.463297	7.546358
C	-1.279329	-4.525285	7.322916
H	-0.692720	-5.241051	7.891670
C	-4.852755	-3.606071	6.972295
H	-5.341961	-4.301659	6.282187
H	-5.109111	-3.886811	7.991421
H	-5.241526	-2.603044	6.807100
O	-0.890703	-1.805326	4.920791
O	0.599832	-3.892694	6.034885
C	-3.284059	-5.333545	8.581237
H	-3.731226	-4.745446	9.390941
H	-4.069680	-5.966893	8.156480
H	-2.528736	-5.984753	9.023635
N	-3.402131	-3.616049	6.804540

pbe_L2_III.log

SCF (wB97x) = -2057.55114223
E(SCF)+ZPE(0 K)= -2056.815755
H(298 K)= -2056.771526
G(298 K)= -2056.892435
Lowest Frequency = 12.2172cm-1

O	1.206112	1.319768	7.446157
N	1.092010	2.450810	4.960822
C	-1.205270	2.552493	5.914099
H	-0.859498	2.911547	6.886059
H	-2.198679	2.983792	5.747128
C	-0.270906	3.002678	4.803768
H	-0.664194	2.670167	3.840126
H	-0.236046	4.099432	4.771527
C	1.822619	2.482333	3.671914
H	2.880891	2.317216	3.893401
H	1.733026	3.467766	3.197332
C	1.349233	1.396618	2.714659
H	0.320069	1.560859	2.382565
H	1.979741	1.424941	1.817213
C	0.856883	-1.044804	2.672157
H	1.466059	-1.923281	2.904023
H	0.900792	-0.906009	1.585564
C	-0.588869	-1.270181	3.090629
H	-1.201174	-0.430575	2.750033
H	-0.986779	-2.173053	2.609995
C	-2.095423	-1.003982	4.986987
H	-2.267621	-1.437991	5.976661
H	-2.835878	-1.446285	4.309197
C	-2.289363	0.498962	5.070823
H	-2.197205	0.967293	4.086695
H	-3.300243	0.715575	5.433654
C	1.761144	2.441005	7.347530
C	1.837794	3.153223	6.014037
H	2.914683	3.209209	5.785545
H	1.477941	4.183911	6.118067
N	2.307899	3.007965	8.409886
H	2.258118	2.450388	9.253670
C	4.582327	3.918370	8.720177
C	5.343915	3.472876	7.626849
C	6.781623	3.197335	7.871122
C	6.411157	3.827566	10.243973
C	7.224189	3.401372	9.208899
H	8.270430	3.206593	9.429124
O	7.541658	2.806421	6.949021
N	5.101514	4.075263	10.004805
C	4.225598	4.557618	11.067334
H	3.953183	5.605292	10.905845
H	3.316602	3.954731	11.121715
H	4.727757	4.479045	12.026727
C	3.138280	4.219801	8.454786
H	2.695087	4.901018	9.182599
H	3.077100	4.700894	7.478251
O	4.832272	3.309027	6.465243
N	-1.275068	1.079906	5.966388
N	1.432437	0.099557	3.386274
H	2.406720	-0.103883	3.608789
C	-0.361390	-2.680916	5.072705
H	0.556204	-3.030749	4.584886
H	-1.133468	-3.440489	4.865645
C	-0.056240	-2.563756	6.549825
O	0.252714	-1.446405	7.032418
C	6.979610	4.018087	11.620826
H	8.046034	3.788368	11.595878
H	6.869906	5.047279	11.979385
H	6.516722	3.354092	12.359617
N	-0.723475	-1.354799	4.557581
H	-1.503100	0.806499	6.919693
In	0.807992	0.163915	5.626356
O	2.713263	-0.532424	5.623800
H	3.072034	-0.773089	6.479788
C	-0.539282	-5.001303	6.951688
H	-1.587341	-4.962442	6.642656
H	-0.488722	-5.568159	7.884650
N	-0.100210	-3.638613	7.317470
H	0.131307	-3.468399	8.287912
C	0.197717	-5.668296	5.832838
C	-0.490768	-5.858032	4.619162
C	0.239604	-6.563856	3.533567
C	2.171591	-6.775341	5.074187
C	1.563562	-6.980574	3.848687
H	2.129882	-7.492846	3.075589
C	2.073086	-5.964876	7.382632
H	1.999333	-4.923493	7.700260
H	1.562000	-6.603039	8.110616
H	3.124809	-6.234531	7.371208
O	-1.677955	-5.425303	4.423151
O	-0.293849	-6.764401	2.413740
C	3.567595	-7.271276	5.318274
H	4.262069	-6.458982	5.560481
H	3.613134	-8.004796	6.130615
H	3.932330	-7.757935	4.412282
N	1.495977	-6.131258	6.053600

pbe_L2_II.log

SCF (wB97x) = -2057.55202337
E(SCF)+ZPE(0 K)= -2056.815615
H(298 K)= -2056.772081
G(298 K)= -2056.889933
Lowest Frequency = 10.7860cm-1

O	0.794229	1.787538	7.604908
N	1.243220	2.339838	5.016846
C	-1.158411	2.808345	5.563599

H	-0.926545	3.199010	6.555843
H	-2.030195	3.361143	5.191528
C	0.004412	3.031625	4.608123
H	-0.282467	2.664475	3.620291
H	0.189464	4.109798	4.498779
C	2.096746	2.070705	3.847395
H	3.072370	1.743540	4.220404
H	2.262028	2.981938	3.253898
C	1.520179	0.982292	2.964597
H	0.597281	1.303469	2.473070
H	2.237765	0.759608	2.163839
C	0.521029	-1.251458	3.001693
H	0.866361	-2.218910	3.367233
H	0.778726	-1.184232	1.936560
C	-0.986110	-1.135093	3.154147
H	-1.304977	-0.148310	2.810834
H	-1.475878	-1.861907	2.485684
C	-2.679701	-0.609913	4.810039
H	-3.027828	-0.922825	5.796969
H	-3.462002	-0.885819	4.081550
C	-2.515174	0.906597	4.781383
H	-2.284029	1.265575	3.774003
H	-3.478134	1.358630	5.054804
C	1.636238	2.677466	7.416973
C	1.991053	3.104937	6.014745
H	3.078667	2.990364	5.883491
H	1.796252	4.182633	5.910938
N	2.233255	3.290363	8.442083
H	1.963360	2.937792	9.350617
C	4.716990	3.521076	8.567894
C	5.475588	3.247224	7.413781
C	6.807266	2.620798	7.610698
C	6.351927	2.605021	10.046678
C	7.160228	2.331825	8.959008
H	8.120970	1.857158	9.140810
O	7.558679	2.359999	6.635715
N	5.153685	3.207188	9.855780
C	4.263372	3.399591	10.996047
H	3.601871	4.245659	10.825854
H	3.661275	2.502535	11.182068
H	4.841543	3.621141	11.890947
C	3.387973	4.191240	8.372806
H	3.209179	5.010844	9.074445
H	3.421545	4.630045	7.374804
O	5.057381	3.516678	6.233608
N	-1.458964	1.375609	5.688793
N	1.232472	-0.208672	3.764605
H	2.119203	-0.609414	4.061178
C	-1.510497	-2.769873	4.809103
H	-0.681339	-3.253677	4.298492
H	-2.443597	-3.169679	4.373862
C	-1.536021	-3.188084	6.272908
O	-2.418605	-2.750153	7.019007
C	6.785965	2.236002	11.435023
H	7.739039	1.707430	11.380531
H	6.931215	3.115133	12.073585
H	6.065587	1.579475	11.935123
N	-1.423227	-1.317969	4.544569
H	-1.733352	1.153857	6.648426
In	0.424187	0.080406	6.017888
O	-0.695267	-0.407138	7.685479
H	-1.216315	-1.208098	7.590802
C	0.614484	-4.604993	6.301212
H	0.673892	-4.506767	5.214885
H	0.689017	-5.669006	6.527893
N	-0.702341	-4.150396	6.746413
H	-0.912882	-4.365194	7.713404
C	1.722308	-3.786787	6.924619
C	1.723980	-2.453651	6.574811
C	2.636653	-1.518889	7.176996
C	3.579111	-3.435366	8.364978
C	3.574858	-2.075101	8.062646
H	4.315887	-1.440476	8.537964
C	2.635891	-5.699472	8.131410
H	1.673400	-5.971966	8.569836
H	2.806749	-6.286864	7.226397
H	3.415688	-5.936207	8.847374
O	0.870878	-1.994537	5.722617
O	2.501595	-0.281611	6.855470
C	4.609939	-3.980703	9.308982
H	4.159843	-4.419761	10.205548
H	5.239701	-4.742419	8.837432
H	5.260681	-3.166782	9.630915
N	2.656938	-4.270458	7.815576

pbe_L2_I.log

SCF (wB97x) =	-2057.55038788
E(SCF)+ZPE(0 K)=	-2056.814354
H(298 K)=	-2056.770670
G(298 K)=	-2056.888544

Lowest Frequency = 9.7706cm-1

O	-0.496415	4.488660	6.540825
N	0.772313	2.641481	4.434364
C	-1.676030	2.957115	4.093043
H	-1.781707	3.618802	4.954651
H	-2.414518	3.253979	3.337204
C	-0.274431	3.124488	3.521388
H	-0.199054	2.563803	2.584331
H	-0.118258	4.181464	3.261923
C	1.850178	1.971307	3.682036
H	2.702277	1.849668	4.355270
H	2.186239	2.586136	2.832530
C	1.466669	0.595379	3.166713
H	0.593354	0.640003	2.509480
H	2.296813	0.202301	2.566246
C	0.754134	-1.666760	3.851309
H	0.961013	-2.374559	4.661600
H	1.323961	-1.990244	2.971473
C	-0.726020	-1.654839	3.522386
H	-0.918679	-0.944411	2.715945
H	-1.038453	-2.639182	3.150940
C	-2.820737	-0.671785	4.249995
H	-3.463165	-0.593324	5.130232
H	-3.311254	-1.340029	3.527543
C	-2.645200	0.708414	3.627658
H	-2.099141	0.668892	2.679793
H	-3.642243	1.106895	3.396508
C	0.642893	4.024367	6.581381
C	1.376430	3.693409	5.285112
H	2.398436	3.397548	5.507171
H	1.422867	4.643559	4.729644
N	1.289445	3.955995	7.776206
H	0.699852	4.277023	8.533653
C	2.198043	1.881120	8.742237
C	1.637174	1.006889	7.840120
C	1.105518	-0.264568	8.254123
C	1.983800	0.266195	10.472457
C	1.345343	-0.607030	9.597609
H	1.013858	-1.567533	9.978940
O	0.460540	-0.947528	7.379755
N	2.382338	1.498717	10.054143
C	3.020510	2.446475	10.969166
H	4.009644	2.720223	10.596068
H	2.404378	3.342884	11.071977
H	3.137596	1.997871	11.949878
C	2.502940	3.259385	8.198331
H	3.012602	3.896395	8.921938
H	3.172232	3.158195	7.341394
O	1.487895	1.349012	6.594748
N	-1.929384	1.584402	4.554366
N	1.168532	-0.321602	4.279851
H	2.004788	-0.396063	4.855235
C	-1.774955	-2.510049	5.466519
H	-0.830181	-3.067683	5.546280
H	-2.461281	-3.156806	4.892949
C	-2.367640	-2.351966	6.851072
O	-2.904039	-1.312864	7.242834
C	2.227738	-0.153293	11.891988

H	1.849177	-1.166667	12.031144
H	3.294176	-0.160144	12.141088
H	1.715038	0.494789	12.610308
N	-1.540749	-1.270148	4.701345
H	-2.453639	1.640551	5.432081
In	-0.272542	0.534039	5.803997
O	-1.587697	1.201520	7.218605
H	-2.084112	0.437945	7.535454
C	-1.713923	-4.752854	7.369502
H	-1.650735	-4.898282	6.290035
H	-2.406813	-5.508535	7.750058
N	-2.334620	-3.448842	7.631545
H	-2.790927	-3.314784	8.523189
C	-0.319529	-4.937699	7.899958
C	0.754436	-4.789741	7.003790
C	2.122376	-4.974528	7.550652
C	1.096806	-5.431391	9.760752
C	2.198059	-5.283835	8.937728
H	3.185083	-5.412939	9.374030
C	-1.327602	-5.494153	10.066962
H	-1.991869	-4.628148	10.045897
H	-1.875043	-6.376756	9.723106
H	-1.039184	-5.655598	11.100970
O	0.591984	-4.486504	5.769303
O	3.140490	-4.851241	6.821906
C	1.278864	-5.785226	11.208904
H	0.856722	-5.029683	11.881041
H	0.827473	-6.750667	11.463343
H	2.346948	-5.855117	11.421464
N	-0.144647	-5.263951	9.245708

pbe_L2_IVac.log

SCF (wB97x) = -1562.46540631
E(SCF)+ZPE(0 K)= -1561.907179
H(298 K)= -1561.874024
G(298 K)= -1561.970053
Lowest Frequency = 16.1757cm-1

C	-2.480011	1.317398	2.584149
H	-2.711247	2.134407	3.274423
H	-3.373353	1.143986	1.969876
C	-1.312154	1.745151	1.704844
H	-1.098614	1.011120	0.921913
H	-1.579339	2.680643	1.196650
C	1.135970	2.174492	1.849936
H	1.735213	2.827117	2.491578
H	0.975052	2.714027	0.908739
C	1.880618	0.878963	1.562645
H	1.312516	0.283950	0.842207
H	2.853400	1.093110	1.100835
C	2.194483	-1.366612	2.453653
H	2.681704	-1.864252	3.297483
H	2.848015	-1.494279	1.581408
C	0.851152	-2.028793	2.205301
H	0.348195	-1.595222	1.335889
H	1.005396	-3.092033	1.988388
C	-1.415525	-2.245173	3.166134
H	-1.831062	-2.552655	4.128137
H	-1.478611	-3.111086	2.496898
C	-2.215075	-1.096977	2.570561
H	-1.824992	-0.859149	1.577407
H	-3.258668	-1.410744	2.433822
N	-2.154121	0.125958	3.396458
N	2.044017	0.067288	2.782814
C	-3.010356	0.010249	4.587623
H	-3.930168	-0.545760	4.369988
H	-3.308532	1.014611	4.905964
O	-3.028290	-1.057484	6.706301
C	-2.317445	-0.623184	5.802324
O	-1.037553	-0.618684	5.806907
N	-0.012266	-1.849061	3.381594
H	0.364134	-2.410980	4.141769
C	3.169011	0.519650	3.603036
N	4.006760	0.011643	5.859290

H	4.124533	0.189219	3.176640
H	3.182251	1.614903	3.635743
C	5.360921	0.503469	5.645743
C	5.751146	1.395979	6.802362
H	5.418496	1.018650	4.690123
H	6.034279	-0.364577	5.587943
C	5.412241	0.973891	8.093581
N	6.439690	2.576838	6.544208
C	5.767160	1.859893	9.220883
O	4.790304	-0.140642	8.302747
C	6.777929	3.405316	7.564670
C	6.803364	2.899502	5.167583
C	6.446568	3.059876	8.860045
O	5.485853	1.560871	10.408991
H	3.902468	-0.284905	6.853374
C	7.505345	4.688954	7.285980
H	7.334920	2.063572	4.707030
H	5.916945	3.141519	4.572728
H	7.465984	3.759133	5.152135
H	6.722557	3.742877	9.658712
H	7.638742	5.228250	8.225104
H	8.500500	4.521027	6.858769
H	6.952226	5.342222	6.602683
N	-0.126580	1.908220	2.544513
H	-0.287522	2.652474	3.222329
C	2.992916	0.012003	5.029063
O	1.857988	-0.398492	5.386233
In	0.099307	0.287039	4.236501
O	0.256195	2.096457	5.155727
H	0.417142	2.063404	6.100437

pbe_L2_IVhc.log

SCF (wB97x) = -1562.47095737
E(SCF)+ZPE(0 K)= -1561.912213
H(298 K)= -1561.879291
G(298 K)= -1561.972134
Lowest Frequency = 23.4230cm-1

C	-1.899324	2.454826	3.943055
H	-2.334976	2.615338	4.935561
H	-2.536484	2.984179	3.219302
C	-0.494803	3.034005	3.935076
H	-0.030122	2.941294	2.947513
H	-0.547836	4.107463	4.160158
C	1.755220	2.672595	4.892419
H	2.126448	2.620955	5.914850
H	1.896776	3.701083	4.535416
C	2.560480	1.735087	3.999448
H	2.230761	1.865563	2.964679
H	3.618616	2.011747	4.045428
C	2.528728	-0.536821	3.137937
H	2.617827	-1.572387	3.481525
H	3.459582	-0.282115	2.610936
C	1.352379	-0.456577	2.181606
H	1.238776	0.544336	1.754138
H	1.542483	-1.137690	1.340755
C	-1.102644	-0.691629	2.101332
H	-1.804747	-1.454066	2.451893
H	-0.920411	-0.891949	1.037711
C	-1.702595	0.696926	2.266257
H	-1.021796	1.438572	1.838690
H	-2.645465	0.772001	1.705195
N	-1.896742	1.008143	3.685383
N	2.367509	0.309702	4.341182
C	-3.097283	0.385401	4.236065
H	-3.281312	-0.568692	3.730249
H	-3.990692	1.004640	4.079352
O	-1.768055	-0.052966	6.176676
C	-2.956930	0.058121	5.725844
O	-3.984145	-0.115438	6.385070
N	0.124458	-0.815537	2.888954
H	0.214643	-1.765308	3.250585
O	4.395144	1.659706	6.261519
C	3.780003	0.620193	6.500059

N	3.765101	0.128390	7.769058
H	4.260819	0.752806	8.393784
C	2.759345	-0.700177	8.436722
H	2.313122	-1.373598	7.703209
H	3.270975	-1.313304	9.178882
C	1.682522	0.178733	9.023735
C	0.910086	0.852981	8.091320
C	0.049200	1.948298	8.518943
C	0.696610	1.355959	10.832725
C	-0.037127	2.105371	9.931927
H	-0.695023	2.875795	10.322944
O	1.082056	0.578623	6.834526
O	-0.533047	2.693691	7.682855
C	0.541022	1.585702	12.307486
H	-0.184031	2.385131	12.466211
H	0.171298	0.695005	12.827190
H	1.480276	1.888194	12.782274
C	2.373205	-0.385993	11.303507
H	2.203145	-1.450421	11.127146
H	3.436818	-0.161927	11.185262
H	2.091729	-0.165564	12.328346
N	1.569093	0.413718	10.381491
C	3.283048	-0.261718	5.365202
H	2.796533	-1.164955	5.736844
H	4.219201	-0.562668	4.867883
N	0.323442	2.329953	4.921425
H	-0.024284	2.541447	5.875080
In	0.063899	0.075671	5.073895
O	0.385891	-1.949308	5.284164
H	0.245225	-2.286743	6.170269

pbe_L2.log

SCF (wB97x) = -1980.95795439
E(SCF)+ZPE(0 K)= -1980.217548
H(298 K)= -1980.175179
G(298 K)= -1980.293045
Lowest Frequency = 8.7980cm-1

O	3.041314	3.521237	6.241359
O	2.174242	-0.716898	5.380469
N	0.493514	2.756408	4.948889
N	1.121499	-1.111419	2.869082
C	-1.451462	1.212481	5.245652
H	-1.324753	1.224820	6.335730
H	-2.534348	1.166976	5.068035
C	-0.921828	2.521218	4.654190
H	-1.040314	2.489085	3.567363
H	-1.545630	3.365739	5.002213
C	1.159564	3.487239	3.867396
H	2.095890	3.884575	4.261951
H	0.541098	4.340788	3.530906
C	1.479970	2.594580	2.674715
H	0.543837	2.197059	2.242885
H	1.930176	3.222486	1.893655
C	2.709429	0.582703	2.003865
H	3.600526	0.016324	2.305905
H	2.983870	1.109421	1.080242
C	1.574571	-0.401529	1.688431
H	0.718190	0.149227	1.285701
H	1.907639	-1.090881	0.888041
C	-0.286020	-1.479429	2.882379
H	-0.415924	-2.322071	3.571061
H	-0.626871	-1.818221	1.888527
C	-1.171850	-0.337230	3.363839
H	-1.065020	0.513352	2.666211
H	-2.222962	-0.652929	3.306717
C	2.023644	3.237654	6.869560
C	2.360815	-1.823135	4.878713
C	2.025161	-2.091967	3.406942
H	1.630290	-3.111863	3.288271
H	2.977036	-2.083677	2.858231
C	0.638199	3.393438	6.252117
H	0.437388	4.481875	6.205128
H	-0.120877	2.973501	6.912426
N	2.136017	2.854378	8.165571
H	3.100249	2.745997	8.449191
N	2.866732	-2.838606	5.613871
H	3.087997	-2.598285	6.584669
C	3.300077	-4.143265	5.133463
H	4.287587	-4.080779	4.665081
H	2.608756	-4.517669	4.373197
C	3.402938	-5.125472	6.265522
C	4.623464	-5.458971	6.781711
C	4.775967	-6.409230	7.863242
C	2.336132	-6.604488	7.809178
C	3.559161	-6.959708	8.340806
H	3.588985	-7.685024	9.146791
C	1.053916	0.805261	9.063050
C	2.145329	0.009834	8.839492
C	2.039123	-1.421872	8.701409
C	-0.338771	-1.139468	9.168441
C	0.737425	-1.948103	8.865755
H	0.579555	-3.016269	8.764973
O	5.770402	-4.926940	6.318371
H	6.457602	-5.355847	6.865652
O	3.093276	-2.066790	8.433970
O	5.937846	-6.659515	8.272328
N	2.258338	-5.693690	6.798505
N	-0.176606	0.209553	9.285218
C	0.966533	-5.357545	6.197905
H	0.170098	-5.524917	6.917627
H	0.953087	-4.302360	5.928482
H	0.781872	-5.971958	5.311308
C	-1.299686	1.054417	9.700780
H	-0.966819	1.775071	10.447836
H	-1.739379	1.580425	8.848352
H	-2.067796	0.440237	10.160326
C	1.153856	2.320453	9.082683
H	0.186782	2.772945	8.870322
H	1.427095	2.659354	10.089557
O	3.391814	0.500350	8.699439
H	3.926578	-0.296799	8.508049
N	-0.842673	-0.003598	4.737598
H	0.171549	0.049252	4.825457
N	2.425219	1.555566	3.044146
H	2.071926	1.055561	3.858040
C	-1.697439	-1.739531	9.367803
H	-1.655213	-2.800898	9.120680
H	-2.034229	-1.655238	10.406905
H	-2.451197	-1.271321	8.727649
C	1.076641	-7.224900	8.333127
H	0.505519	-7.720534	7.541946
H	1.332518	-7.972013	9.085130
H	0.423673	-6.483879	8.806981

pbe_L2_TS(III).log

SCF (wB97x) = -2057.51820602
E(SCF)+ZPE(0 K)= -2056.782037
H(298 K)= -2056.739387
G(298 K)= -2056.856582
Lowest Frequency = -207.6880cm-1

O	-0.161227	1.702170	7.807791
N	0.367923	3.107948	5.437281
C	-2.090529	3.005728	5.722696
H	-2.036601	3.272426	6.780472
H	-3.035569	3.392170	5.325153
C	-0.925149	3.637357	4.972219
H	-1.012739	3.439182	3.899707
H	-0.966909	4.727708	5.094923
C	1.432018	3.249909	4.422611
H	2.389563	3.198762	4.945959
H	1.372694	4.227811	3.927717
C	1.360909	2.145878	3.379662
H	0.437682	2.207424	2.795832
H	2.192196	2.257669	2.674505
C	1.101822	-0.297728	3.107440
H	1.592426	-1.204084	3.480242

H	1.497572	-0.097354	2.105302
C	-0.402315	-0.502493	3.029541
H	-0.883424	0.397639	2.636612
H	-0.634675	-1.318540	2.333981
C	-2.417078	-0.546699	4.426599
H	-2.796192	-1.043262	5.325492
H	-2.917961	-1.003685	3.563259
C	-2.759283	0.931821	4.504423
H	-2.470507	1.462448	3.593679
H	-3.844979	1.042792	4.603312
C	0.897790	2.492728	7.754872
C	0.781125	3.649067	6.756929
H	1.731137	4.191247	6.671023
H	0.009727	4.326658	7.137801
N	1.418706	2.826482	8.987888
H	1.265221	2.096821	9.668776
C	3.914979	3.246743	8.683676
C	4.403973	3.844054	7.506422
C	5.727099	3.385850	7.016114
C	5.832767	1.843027	8.953394
C	6.361670	2.375623	7.791678
H	7.325456	2.006454	7.450458
O	6.239560	3.865427	5.969706
N	4.627904	2.279426	9.392498
C	4.093055	1.821205	10.669743
H	4.101074	2.630333	11.406606
H	3.072372	1.453967	10.553130
H	4.692622	1.002350	11.055600
C	2.577168	3.697962	9.203308
H	2.601646	3.894427	10.279574
H	2.382363	4.655938	8.715638
O	3.753313	4.734877	6.856415
N	-2.061265	1.528453	5.652297
N	1.411742	0.816307	4.023721
H	2.349602	0.690063	4.397785
C	-0.618669	-2.147048	4.796446
H	0.070502	-2.638017	4.091241
H	-1.518798	-2.766837	4.861295
C	0.106793	-2.162392	6.120542
O	0.371923	-1.087465	6.723793
C	6.593059	0.801784	9.723823
H	7.519273	0.577846	9.191771
H	6.864052	1.139759	10.730384
H	6.035063	-0.135970	9.824713
N	-0.957897	-0.782958	4.369427
H	-2.502013	1.182708	6.501721
In	0.104784	0.830665	5.884427
O	1.966503	1.536409	6.848530
H	2.441862	0.985940	7.479224
C	0.465901	-4.631308	5.999244
H	-0.109009	-4.544294	5.076313
H	-0.074189	-5.316839	6.652481
N	0.479953	-3.311707	6.645532
H	0.993587	-3.232434	7.514808
C	1.849397	-5.092628	5.652040
C	2.460435	-4.467118	4.553617
C	3.817109	-4.931013	4.175092
C	3.711432	-6.510770	6.087916
C	4.363806	-5.952483	5.003162
H	5.359728	-6.315820	4.764715
C	1.743833	-6.668604	7.536956
H	1.393640	-5.890461	8.219081
H	0.887928	-7.253759	7.186997
H	2.399553	-7.326910	8.098285
O	1.877723	-3.534883	3.897110
O	4.426714	-4.442413	3.190650
C	4.375650	-7.590087	6.893590
H	4.532469	-7.295507	7.937321
H	3.803830	-8.524383	6.890755
H	5.354934	-7.800272	6.460760
N	2.468888	-6.081592	6.414708

pbe_L2_TS(II).log

SCF (wB97x) = -2057.51651930

E(SCF)+ZPE(0 K)= -2056.780263
H(298 K)= -2056.737947
G(298 K)= -2056.851764
Lowest Frequency = -275.7053cm-1

N	0.084273	1.589823	4.882270
C	-2.080195	1.563141	3.724687
H	-2.593040	2.018087	4.576385
H	-2.604017	1.889300	2.816031
C	-0.634563	2.057746	3.691871
H	-0.111582	1.699258	2.799209
H	-0.636982	3.153553	3.639355
C	1.546615	1.768327	4.877766
H	1.855772	1.941042	5.908107
H	1.821125	2.657821	4.296608
C	2.298799	0.571642	4.304669
H	2.022595	0.458302	3.251955
H	3.373682	0.784288	4.336317
C	2.178570	-1.827894	4.022862
H	2.238412	-2.754632	4.603769
H	3.129451	-1.728745	3.479222
C	1.031572	-1.953872	3.046585
H	0.981869	-1.102546	2.362628
H	1.185611	-2.846898	2.427806
C	-1.412098	-2.028324	2.910059
H	-2.203867	-2.582911	3.416513
H	-1.194400	-2.538864	1.964224
C	-1.859671	-0.605165	2.631814
H	-1.068702	-0.062549	2.105653
H	-2.736512	-0.611230	1.969652
N	-2.154437	0.099117	3.886616
C	-3.412901	-0.369266	4.504921
H	-3.946074	-1.028631	3.807807
H	-4.074326	0.475410	4.729615
C	-3.124709	-1.177664	5.769839
O	-2.095303	-1.958932	5.780234
N	-0.227978	-2.035567	3.788366
H	-0.241137	-2.910653	4.306998
In	-0.521092	-0.444087	5.532220
O	-2.221174	0.183282	6.687497
H	-2.454479	1.111333	6.797460
C	-5.335415	-0.682163	6.800427
H	-4.969684	0.332663	6.988456
H	-5.766190	-1.052284	7.736565
N	-4.202304	-1.570085	6.504643
H	-3.912274	-2.152201	7.278851
C	-6.385634	-0.558843	5.734179
C	-6.524177	0.673935	5.067431
C	-7.607159	0.778278	4.057286
C	-8.229438	-1.560545	4.590107
C	-8.409778	-0.384175	3.885586
H	-9.213060	-0.341216	3.154565
C	-7.063144	-2.844737	6.311243
H	-7.479127	-2.696851	7.313984
H	-7.571632	-3.685153	5.846493
H	-6.004850	-3.092026	6.390791
O	-5.746104	1.669357	5.282853
O	-7.791339	1.837146	3.400327
C	-9.140516	-2.731638	4.360785
H	-8.606101	-3.608534	3.977457
H	-9.666704	-3.035104	5.272475
H	-9.892227	-2.454519	3.619732
N	-7.229349	-1.643973	5.497999
N	1.998422	-0.713939	4.975667
O	3.888000	1.015348	6.749641
C	3.281672	0.011550	7.118423
N	3.204263	-0.282308	8.447741
H	3.658274	0.441575	8.992451
C	2.125295	-0.961752	9.169429
H	1.723287	-1.763749	8.549849
H	2.556145	-1.417761	10.060207
C	1.040584	0.043240	9.470947
C	0.379522	0.539962	8.358169
C	-0.353417	1.794051	8.469155
C	0.066559	1.659947	10.905064

C	-0.517741	2.256292	9.806702
H	-1.110916	3.152000	9.962945
O	0.612810	-0.051762	7.211301
O	-0.774763	2.433555	7.462797
C	-0.163548	2.210815	12.280877
H	-0.805973	3.089294	12.207368
H	-0.663493	1.488038	12.934884
H	0.768842	2.517342	12.766091
C	1.536137	-0.067768	11.865656
H	1.332124	-1.140027	11.864074
H	2.615961	0.100460	11.827995
H	1.158395	0.342837	12.796768
N	0.868200	0.572735	10.733105
C	2.869004	-1.059667	6.126279
H	2.402594	-1.900010	6.640440
H	3.838212	-1.414889	5.736701
H	-0.275229	2.066957	5.729325

wb97xd_H2O.log

SCF (wB97x) = -76.4387237009
E(SCF)+ZPE(0 K)= -76.417095
H(298 K)= -76.413315
G(298 K)= -76.435381
Lowest Frequency = 1610.4044cm-1

O	-0.209293	1.499000	0.000000
H	0.749010	1.533266	0.000000
H	-0.496884	2.413779	0.000000

wb97xd_HOPO.log

SCF (wB97x) = -571.963457949
E(SCF)+ZPE(0 K)= -571.760766
H(298 K)= -571.748099
G(298 K)= -571.797712
Lowest Frequency = 82.1320cm-1

C	-2.174003	-2.332556	-0.121207
C	-0.813958	-2.241941	-0.187952
C	-0.112640	-0.977138	-0.122478
C	-0.954775	0.158069	0.011131
C	-2.325890	0.043950	0.089371
H	-0.504664	1.143512	0.049741
N	-2.923994	-1.179478	0.047211
C	-4.387235	-1.297041	0.111546
H	-4.643821	-2.222104	0.623976
H	-4.798953	-0.471939	0.685717
H	-4.815339	-1.285046	-0.894412
C	-3.182606	1.272730	0.216374
H	-2.553422	2.158874	0.132400
H	-3.943210	1.317797	-0.567886
H	-3.690066	1.312379	1.185277
C	-2.858986	-3.672415	-0.213178
H	-2.149745	-4.367424	-0.674149
H	-3.719682	-3.606519	-0.884821
O	-0.031083	-3.336372	-0.330236
H	0.874005	-2.980479	-0.358800
O	1.142065	-0.979462	-0.194659
N	-3.331196	-4.115392	1.103249
H	-3.838522	-4.987665	1.009904
H	-2.540624	-4.298386	1.710842

wb97xd_InOH3.log

SCF (wB97x) = -229.462228258
E(SCF)+ZPE(0 K)= -229.423425
H(298 K)= -229.415386
G(298 K)= -229.455021
Lowest Frequency = 126.2271cm-1

In	0.745365	-0.634567	0.063341
O	0.058255	1.105365	-0.461050
H	-0.881480	1.200812	-0.623884
O	2.645618	-0.872012	0.389031
H	3.229916	-0.120118	0.279576
O	-0.472461	-2.135147	0.262562
H	-0.118871	-2.987321	0.522394

wb97xd_L1_III.log

SCF (wB97x) = -2059.28639442
E(SCF)+ZPE(0 K)= -2058.546039
H(298 K)= -2058.502397
G(298 K)= -2058.621913
Lowest Frequency = 10.9596cm-1

O	-1.753505	2.873077	5.750239
N	-0.302206	2.184924	3.562524
C	-2.646888	1.871208	2.796828
H	-2.926658	2.854116	3.181521
H	-3.260328	1.690797	1.907542
C	-1.175126	1.866296	2.410415
H	-0.884018	0.890121	2.016184
H	-1.016301	2.588565	1.600315
C	1.103187	1.806324	3.281541
H	1.728709	2.295473	4.032325
H	1.415317	2.173356	2.296508
C	1.317807	0.302895	3.375542
H	0.788237	-0.234209	2.584519
H	2.384405	0.087632	3.248778
C	0.766028	-1.630478	4.818330
H	0.845727	-1.861503	5.883730
H	1.600697	-2.117982	4.303525
C	-0.556988	-2.149073	4.275923
H	-0.638218	-1.937761	3.205633
H	-0.620123	-3.234559	4.398426
C	-2.955688	-1.601223	4.242572
H	-3.772189	-1.584371	4.966221
H	-3.025396	-2.562794	3.723931
C	-3.104690	-0.479701	3.223693
H	-2.344024	-0.588290	2.447547
H	-4.080427	-0.549220	2.730985
C	-1.242440	3.841053	5.141863
C	-0.389688	3.611295	3.914211
H	0.596912	4.031317	4.162082
H	-0.786688	4.195190	3.076419
N	-1.436691	5.074865	5.575306
H	-1.981177	5.139896	6.425612
C	0.245878	6.820627	6.056983
C	1.513781	6.229725	6.004767
C	2.547446	6.762052	6.930581
C	0.831420	8.314327	7.821002
C	2.114348	7.800233	7.803849
H	2.841101	8.209509	8.499168
O	3.719609	6.311753	6.932749
N	-0.096893	7.826808	6.966707
C	-1.455834	8.370277	6.939813
H	-1.616512	8.971377	6.040708
H	-2.192022	7.565850	6.973245
H	-1.624697	8.998448	7.808034
C	-0.787411	6.306204	5.096116
H	-1.573116	7.026956	4.871694
H	-0.282270	6.078659	4.158488
O	1.793920	5.275496	5.195281
N	-2.941317	0.860884	3.841920
N	0.827238	-0.167486	4.674215
H	1.417996	0.204371	5.414862
C	-4.155639	1.230518	4.599757
H	-5.067029	0.992718	4.037190
H	-4.140554	2.311171	4.774833
C	-4.123736	0.631592	5.993588
O	-3.026655	0.514188	6.580117
C	0.472635	9.416736	8.782961
H	1.360082	9.690038	9.354788
H	0.117686	10.314239	8.267281
H	-0.298121	9.106544	9.495962
N	-1.674289	-1.473910	4.963985
H	-1.782759	-1.894789	5.882828
In	-1.175731	0.792746	5.401929

O	-0.062897	0.741809	7.074856
H	-0.456313	1.133806	7.854865
C	-6.553255	0.066373	5.934611
H	-6.835731	0.948095	5.359351
H	-7.285721	-0.036046	6.737428
N	-5.254855	0.296790	6.596095
H	-5.146484	-0.012506	7.552591
C	-6.520770	-1.089804	4.972146
C	-6.666912	-0.812149	3.605474
C	-6.489641	-1.948259	2.659487
C	-6.044776	-3.403583	4.608820
C	-6.152868	-3.200028	3.247911
H	-5.967113	-4.038888	2.584177
C	-6.172576	-2.604611	6.906588
H	-5.145501	-2.488448	7.266341
H	-6.824455	-1.913242	7.435079
H	-6.515523	-3.608127	7.146593
O	-6.874861	0.369232	3.154651
O	-6.593742	-1.782940	1.420364
C	-5.678123	-4.756941	5.154093
H	-4.820448	-4.703651	5.831597
H	-6.508299	-5.220978	5.696950
H	-5.415006	-5.415794	4.325891
N	-6.258116	-2.375654	5.462585

wb97xd_L1_II.log

SCF (wB97x) = -2059.28159455
E(SCF)+ZPE(0 K)= -2058.541067
H(298 K)= -2058.497973
G(298 K)= -2058.612669
Lowest Frequency = 21.4995cm-1

O	-0.402634	4.399770	6.366043
N	0.372792	2.001530	4.475432
C	-2.096137	2.377981	4.552948
H	-1.975688	2.944418	5.476810
H	-2.926901	2.804816	3.974248
C	-0.810959	2.511304	3.751976
H	-0.894411	1.977829	2.801371
H	-0.683322	3.571221	3.498700
C	1.299287	1.328077	3.533911
H	2.286004	1.294223	3.999053
H	1.396998	1.900424	2.601407
C	0.896466	-0.101300	3.221311
H	-0.069788	-0.143433	2.712693
H	1.636993	-0.535389	2.538131
C	0.584242	-2.304470	4.288729
H	0.773702	-2.790067	5.250525
H	1.269050	-2.731708	3.546421
C	-0.859614	-2.527389	3.866475
H	-1.017537	-2.134884	2.857820
H	-1.079916	-3.599593	3.827706
C	-2.978314	-1.311160	4.166301
H	-3.818287	-1.450575	4.847719
H	-3.236102	-1.877452	3.264397
C	-2.835848	0.160731	3.790838
H	-2.078234	0.259610	3.011000
H	-3.782877	0.530630	3.376641
C	0.607760	3.701757	6.412920
C	1.165175	3.063565	5.144787
H	2.133589	2.631266	5.364309
H	1.313360	3.900131	4.444533
N	1.349509	3.649394	7.550641
H	0.892182	4.124297	8.316421
C	2.180414	1.568170	8.620103
C	1.653806	0.571015	7.828030
C	0.946061	-0.528953	8.440939
C	1.724071	0.308580	10.590422
C	1.070614	-0.656190	9.831572
H	0.607214	-1.493778	10.341092
O	0.210926	-1.255647	7.671760
N	2.233546	1.416845	9.994910
C	2.875670	2.478291	10.781126
H	3.908709	2.609432	10.455300
H	2.329068	3.415358	10.664003
H	2.879775	2.218416	11.833462
C	2.530293	2.862895	7.919065
H	3.171902	3.509366	8.517350
H	3.091763	2.617257	7.018846
O	1.595286	0.659519	6.537784
N	-2.423450	0.983781	4.946907
N	0.821764	-0.869566	4.470632
H	1.688879	-0.730609	4.986828
C	-3.525812	1.058687	5.929505
H	-4.473280	1.280394	5.426246
H	-3.293217	1.876170	6.617392
C	-3.597165	-0.157776	6.830509
O	-2.560933	-0.746201	7.168361
C	1.842761	0.129898	12.080137
H	1.386691	-0.819858	12.360233
H	2.886997	0.106279	12.405932
H	1.328084	0.922214	12.631718
N	-1.748275	-1.817234	4.789437
H	-1.988828	-2.419915	5.567135
In	-0.525493	0.117952	6.065874
O	-1.012475	1.597483	7.388084
H	-1.331525	1.235413	8.214767
C	-6.107014	-0.155183	6.770002
H	-6.203217	0.930638	6.743841
H	-6.845045	-0.516331	7.490192
N	-4.790408	-0.529775	7.311404
H	-4.749432	-1.295303	7.969228
C	-6.339491	-0.638206	5.362198
C	-6.475710	0.322154	4.348408
C	-6.547553	-0.176383	2.947925
C	-6.342505	-2.475804	3.833296
C	-6.447642	-1.586588	2.782895
H	-6.451378	-1.980783	1.771176
C	-6.240219	-2.965563	6.215235
H	-5.199748	-3.218716	6.441402
H	-6.713726	-2.549574	7.101248
H	-6.778025	-3.877583	5.966819
O	-6.478245	1.582520	4.580366
O	-6.654588	0.615879	1.979296
C	-6.233554	-3.954621	3.578725
H	-5.360587	-4.389142	4.075630
H	-7.120464	-4.498879	3.920109
H	-6.132717	-4.125809	2.506354
N	-6.323591	-2.014629	5.104980

wb97xd_L1_I.log

SCF (wB97x) = -2059.27833658
E(SCF)+ZPE(0 K)= -2058.536624
H(298 K)= -2058.493889
G(298 K)= -2058.606582
Lowest Frequency = 26.6785cm-1

O	-1.475521	3.746957	6.860159
N	0.599653	2.776556	4.873472
C	-1.680430	2.582125	3.827922
H	-2.141306	2.681292	4.808536
H	-2.328862	3.105028	3.105074
C	-0.330114	3.289433	3.847511
H	0.153201	3.244121	2.867653
H	-0.537698	4.351311	4.024526
C	1.950982	2.516732	4.339262
H	2.611833	2.363467	5.193200
H	2.327287	3.382257	3.772409
C	2.053569	1.289811	3.455681
H	1.451149	1.401464	2.558910
H	3.095122	1.195723	3.124022
C	1.878419	-1.163550	3.319461
H	1.554323	-2.024547	3.914833
H	2.957750	-1.246006	3.136314
C	1.171782	-1.188619	1.964267
H	1.421583	-0.297351	1.375598
H	1.630793	-2.028020	1.421866
C	-1.117245	-0.368485	1.425159

H	-2.123745	-0.789284	1.329597
H	-0.783052	-0.193962	0.394431
C	-1.189420	1.028435	2.064404
H	-0.214551	1.497588	1.970822
H	-1.876564	1.630102	1.443872
C	-0.277743	3.591815	7.108237
C	0.760272	3.707699	6.007784
H	1.751522	3.567523	6.422923
H	0.704786	4.749842	5.657784
N	0.138999	3.442952	8.388109
H	-0.632211	3.426925	9.042768
C	1.319189	1.441597	9.153875
C	1.206453	0.642638	8.037357
C	0.765320	-0.725060	8.183450
C	0.910424	-0.414930	10.595867
C	0.682419	-1.226645	9.494756
H	0.401920	-2.261743	9.655501
O	0.458011	-1.347937	7.117116
N	1.197532	0.901275	10.426986
C	1.402214	1.789561	11.577509
H	2.373356	2.280335	11.500743
H	0.609779	2.539403	11.623689
H	1.385715	1.219947	12.499603
C	1.407682	2.930607	8.914494
H	1.645795	3.500980	9.810646
H	2.203134	3.128178	8.197841
O	1.343440	1.087958	6.823965
N	-1.620657	1.142813	3.485045
N	1.645584	0.051916	4.152540
H	2.285222	-0.035653	4.943873
C	-2.978446	0.553996	3.539789
H	-2.892033	-0.451157	3.149390
H	-3.640396	1.118470	2.862848
C	-3.719633	0.526925	4.875863
O	-4.153994	1.564066	5.375400
C	0.820961	-0.995752	11.981582
H	0.572164	-2.054485	11.905888
H	1.770458	-0.914930	12.519527
H	0.044095	-0.511622	12.580461
N	-0.271153	-1.371263	2.033400
H	-0.571664	-1.566355	2.987608
In	-0.153007	0.154289	5.512126
O	-1.632079	0.750819	6.704020
H	-1.862303	1.680333	6.706510
C	-3.763232	-2.019685	4.931722
H	-3.427903	-1.957131	3.898918
H	-4.713517	-2.555887	4.898994
N	-4.029325	-0.674556	5.414171
H	-4.542015	-0.594610	6.280463
C	-2.720643	-2.845671	5.655629
C	-1.392160	-2.636644	5.355319
C	-0.415002	-3.691985	5.637787
C	-2.231615	-4.849253	6.850833
C	-0.920708	-4.759445	6.445556
H	-0.231064	-5.544252	6.739681
C	-4.533038	-3.993406	6.875856
H	-4.605032	-4.538303	7.812931
H	-4.929369	-2.995412	7.055692
H	-5.144754	-4.500873	6.124532
O	-1.030146	-1.557664	4.687535
O	0.744960	-3.671342	5.171159
C	-2.688706	-5.991663	7.716972
H	-2.992862	-5.649767	8.711779
H	-3.529545	-6.531809	7.272459
H	-1.864624	-6.693993	7.845500
N	-3.131989	-3.908722	6.454567

wb97xd_L1_IVac.log

SCF (wB97x) = -1563.78279233
E(SCF)+ZPE(0 K)= -1563.220755
H(298 K)= -1563.187840
G(298 K)= -1563.282030
Lowest Frequency = 18.4217cm-1

O	-1.348725	-1.499911	6.010903
N	-1.146887	1.146300	5.676117
C	-2.187047	2.227070	3.692272
H	-3.182960	1.836648	3.915521
H	-2.332635	3.199767	3.207014
C	-1.411786	2.423930	4.984466
H	-0.445865	2.889862	4.776552
H	-1.962466	3.110612	5.638999
C	-0.027253	1.299828	6.641169
H	-0.141986	0.558014	7.436337
H	-0.072418	2.283005	7.122628
C	1.316672	1.098168	5.965614
H	1.501163	1.890213	5.236678
H	2.112531	1.156138	6.715337
C	2.480797	-0.377118	4.362295
H	2.560769	-1.445685	4.149289
H	3.411679	-0.046886	4.835337
C	2.257917	0.386391	3.063269
H	2.229291	1.464838	3.243677
H	3.093676	0.197454	2.381112
C	0.499601	0.886068	1.405685
H	-0.005491	0.286395	0.645734
H	1.329941	1.395874	0.905905
C	-0.449541	1.926928	1.993040
H	0.105923	2.593696	2.657549
H	-0.869237	2.548067	1.191809
C	-2.043433	-0.805517	6.781767
C	-2.327559	0.637613	6.388426
H	-2.557666	1.267181	7.249466
H	-3.196347	0.628690	5.724774
C	-1.687860	0.161979	9.677050
C	-0.592212	-0.672503	9.920346
C	0.695014	-0.014161	10.261673
C	-0.477126	2.165981	10.113057
C	0.666116	1.412305	10.290652
H	1.598472	1.932594	10.488332
O	1.737715	-0.679249	10.473651
N	-1.650748	1.549072	9.836617
C	-2.909551	2.299543	9.831589
H	-3.247208	2.525058	8.815443
H	-3.679379	1.730316	10.352599
H	-2.784038	3.239166	10.361723
C	-2.910862	-0.496852	9.080551
H	-3.363157	-1.201552	9.782552
H	-3.694107	0.200572	8.788323
O	-0.672095	-1.946914	9.787240
N	-1.522743	1.281082	2.771051
N	1.330470	-0.199329	5.269361
H	1.362068	-0.936101	5.969294
C	-2.536472	0.666019	1.899701
H	-2.050496	0.185374	1.046459
H	-3.232555	1.413623	1.503132
O	-4.421565	-0.756067	2.237550
C	-3.310469	-0.429197	2.643517
O	-2.705656	-0.947957	3.647027
C	-0.431590	3.666420	10.220663
H	0.609502	3.987331	10.272265
H	-0.894062	4.153167	9.356831
H	-0.933962	4.029557	11.123711
N	0.978787	-0.016464	2.460147
H	1.074549	-0.961972	2.096575
In	-0.640461	-0.582540	4.096341
O	-0.015793	-2.385319	3.425248
H	-0.545869	-3.138525	3.686656
N	-2.521576	-1.302668	7.912119
H	-2.086039	-2.179577	8.186712

wb97xd_L1_IVhc.log

SCF (wB97x) = -1563.77001382
E(SCF)+ZPE(0 K)= -1563.207847
H(298 K)= -1563.175398
G(298 K)= -1563.266574
Lowest Frequency = 30.9161cm-1

O	-1.163448	4.290531	6.784865
N	0.722121	2.834287	4.730593
C	-1.663930	2.725461	4.003653
H	-1.969984	2.941082	5.028047
H	-2.378932	3.187755	3.307990
C	-0.290906	3.345152	3.788634
H	0.056166	3.166573	2.766869
H	-0.399801	4.433252	3.887190
C	2.019517	2.631332	4.052805
H	2.796182	2.590667	4.818652
H	2.258501	3.475935	3.390026
C	2.094224	1.336299	3.266576
H	1.371371	1.323857	2.448188
H	3.090325	1.262031	2.811449
C	2.092969	-1.107345	3.517593
H	2.083767	-1.864248	4.307737
H	3.076258	-1.129347	3.031562
C	0.991532	-1.399334	2.507190
H	1.063739	-0.706500	1.663315
H	1.116292	-2.409187	2.099318
C	-1.371390	-0.688742	2.314627
H	-2.312704	-1.170963	2.586597
H	-1.203548	-0.922884	1.256081
C	-1.490687	0.827642	2.454876
H	-0.575440	1.291499	2.084328
H	-2.314384	1.192338	1.824854
C	-0.054368	3.802056	6.992032
C	0.980629	3.735897	5.874915
H	1.934115	3.440567	6.295876
H	1.077052	4.772376	5.513405
N	0.327905	3.490200	8.258249
H	-0.432529	3.592674	8.915766
C	1.351391	1.342304	8.973280
C	1.309479	0.588795	7.820947
C	0.825420	-0.771727	7.868070
C	0.813384	-0.565465	10.295533
C	0.655259	-1.329177	9.144211
H	0.348911	-2.364657	9.244654
O	0.548093	-1.334676	6.745733
N	1.117355	0.754296	10.205299
C	1.230581	1.594376	11.403957
H	2.241979	1.996435	11.485028
H	0.510342	2.412645	11.357281
H	1.021006	1.012012	12.293881
C	1.526132	2.833564	8.787967
H	1.793080	3.346893	9.711619
H	2.352806	2.991113	8.096445
O	1.537584	1.089132	6.650460
N	-1.699951	1.249672	3.854634
N	1.843859	0.191340	4.152362
H	2.436066	0.289767	4.975594
C	-3.015948	0.764195	4.326537
H	-3.760618	0.834527	3.524971
H	-3.339974	1.407717	5.146623
O	-4.107876	-1.254409	4.933522
C	-3.019471	-0.663087	4.896721
O	-1.916346	-1.114751	5.328959
C	0.630937	-1.207216	11.645036
H	0.418045	-2.267477	11.506818
H	1.532390	-1.126318	12.259907
H	-0.204168	-0.769880	12.200332
N	-0.299557	-1.214993	3.159972
H	-0.601466	-2.069220	3.609675
In	-0.136107	0.317036	5.369690
O	-1.308353	1.207109	6.799226
H	-1.950277	0.575550	7.124026

wb97xd_L1.log

SCF (wB97x) = -1982.63730812
E(SCF)+ZPE(0 K)= -1981.890503
H(298 K)= -1981.849233
G(298 K)= -1981.961978
Lowest Frequency = 12.2682cm-1

O	2.180586	0.417763	6.629080
N	0.641537	1.953269	4.915949
C	-1.837033	1.783844	4.401692
H	-2.193117	2.047234	5.402775
H	-2.567389	2.233299	3.704535
C	-0.491837	2.461290	4.145790
H	-0.231106	2.362798	3.088099
H	-0.641199	3.543319	4.323935
C	1.885966	2.589724	4.463268
H	2.611668	2.573302	5.281482
H	1.715738	3.648593	4.207483
C	2.494104	1.858759	3.271553
H	1.745789	1.805212	2.467416
H	3.325091	2.458552	2.876785
C	2.832825	-0.515578	2.677444
H	3.268802	-1.427413	3.101858
H	3.432144	-0.252914	1.796215
C	1.408138	-0.830502	2.208762
H	0.998155	0.028132	1.647871
H	1.464909	-1.662280	1.495839
C	-0.839734	-1.448693	2.905253
H	-1.306315	-2.199621	3.556576
H	-0.850207	-1.877502	1.897339
C	-1.673175	-0.166481	2.926172
H	-1.161221	0.591712	2.326546
H	-2.657411	-0.329095	2.455817
C	1.446578	1.253358	7.147238
C	0.444442	2.076965	6.341962
H	0.470335	3.128307	6.681512
H	-0.542035	1.678471	6.599604
N	1.507798	1.474929	8.482645
H	2.173192	0.890717	8.972686
C	-0.349789	1.226421	10.066038
C	0.214217	0.241061	10.819750
C	-0.556695	-0.813429	11.438162
C	-2.521670	0.348214	10.542873
C	-1.959655	-0.698429	11.241392
H	-2.611110	-1.447769	11.675586
O	0.049684	-1.713722	12.064698
N	-1.728831	1.296465	9.952059
C	-2.312896	2.444600	9.243942
H	-1.939774	3.374723	9.673918
H	-2.079266	2.397878	8.178806
H	-3.391259	2.430416	9.342917
C	0.567994	2.190968	9.332325
H	0.019528	2.915048	8.737423
H	1.140567	2.761032	10.069167
O	1.555310	0.171757	10.975407
H	1.704991	-0.619477	11.521495
N	-1.817003	0.331066	4.295880
N	2.970242	0.539263	3.671339
H	2.523586	0.274857	4.544769
C	-2.965508	-0.266458	4.958478
H	-2.997711	-1.339358	4.743765
H	-3.921171	0.166399	4.622082
O	-3.822407	0.486698	7.070789
C	-2.917847	-0.087628	6.470254
N	-1.820492	-0.532268	7.132036
H	-1.876853	-0.412499	8.135121
C	-0.835503	-1.497957	6.654327
H	0.163343	-1.063562	6.675560
H	-1.042968	-1.719074	5.611173
C	-0.940483	-2.778531	7.447520
C	-1.992654	-3.611449	7.211377
C	-0.184424	-4.212295	9.200256
C	-2.217233	-4.821173	7.971261
C	-1.247498	-5.062296	8.981103
H	-1.346344	-5.947223	9.599204
O	-2.899653	-3.329407	6.251874
H	-3.533838	-4.066140	6.287092
O	-3.212480	-5.533180	7.692948
N	-0.032800	-3.083365	8.448277
C	1.129153	-2.204891	8.625735
H	1.816973	-2.303303	7.783460
H	0.804278	-1.169033	8.706999

H	1.649038	-2.457750	9.542496
N	0.546726	-1.231086	3.317294
H	0.544861	-0.494097	4.023491
C	-4.015629	0.447721	10.413532
H	-4.469579	-0.422269	10.887888
H	-4.401028	1.340946	10.914662
H	-4.318332	0.470041	9.362692
C	0.814694	-4.519541	10.280543
H	0.539593	-5.458018	10.762166
H	1.825872	-4.631250	9.878767
H	0.819436	-3.731777	11.041580

wb97xd_L1_TS(III).log

SCF (wB97x) = -2059.24882997
E(SCF)+ZPE(0 K)= -2058.507116
H(298 K)= -2058.465673
G(298 K)= -2058.578449
Lowest Frequency = -334.0082cm-1

O	-2.067212	2.845399	5.491939
N	-0.351988	1.973242	3.426812
C	-2.730014	1.882763	2.686592
H	-2.957082	2.834753	3.166938
H	-3.364813	1.785513	1.799792
C	-1.259876	1.871769	2.264026
H	-1.024171	0.944909	1.733676
H	-1.088692	2.693799	1.559317
C	1.012936	1.469420	3.134166
H	1.688212	1.907798	3.873775
H	1.339260	1.796793	2.140650
C	1.104844	-0.042155	3.245466
H	0.483946	-0.537351	2.495706
H	2.138049	-0.354066	3.060817
C	0.523575	-1.929790	4.721572
H	0.561253	-2.174497	5.786481
H	1.346384	-2.454857	4.225537
C	-0.808526	-2.369322	4.133452
H	-0.832305	-2.190506	3.054919
H	-0.952255	-3.443007	4.286669
C	-3.160153	-1.650802	3.991865
H	-3.996199	-1.619256	4.692930
H	-3.252642	-2.598422	3.452285
C	-3.253718	-0.499076	2.997360
H	-2.496743	-0.614849	2.217396
H	-4.232647	-0.517612	2.504714
C	-0.874701	3.331995	5.441574
C	-0.279820	3.331789	4.020261
H	0.748834	3.684772	3.987785
H	-0.895752	4.028718	3.444702
N	-0.600164	4.454447	6.197814
H	-1.173084	4.461971	7.031189
C	1.595599	4.416955	7.493144
C	2.437833	3.339921	7.221214
C	3.360476	2.889532	8.283642
C	2.382190	4.634465	9.739289
C	3.265846	3.600241	9.516346
H	3.922646	3.298242	10.326367
O	4.167450	1.943833	8.103883
N	1.549051	5.032696	8.746463
C	0.627702	6.151824	8.958832
H	-0.337648	5.945282	8.499536
H	0.452039	6.293103	10.020778
H	1.033365	7.078513	8.542788
C	0.755796	4.989936	6.375021
H	0.669176	6.074060	6.472195
H	1.312082	4.809776	5.456450
O	2.415350	2.720112	6.077636
N	-3.041020	0.806734	3.665405
N	0.663442	-0.469600	4.588907
H	1.356445	-0.145709	5.259265
C	-4.202362	1.159738	4.510399
H	-5.147969	0.936463	4.003547
H	-4.164276	2.234263	4.709425
C	-4.098134	0.530552	5.891711
O	-2.975030	0.355402	6.427635
C	2.346849	5.328242	11.075334
H	3.130044	4.913973	11.711166
H	2.521625	6.404437	10.983738
H	1.390911	5.182104	11.588968
N	-1.894917	-1.593595	4.753565
H	-2.069258	-1.976496	5.678609
In	-1.290833	0.710542	5.132200
O	-0.033917	1.941064	6.194764
H	0.953728	2.069921	6.124420
C	-6.548863	0.067145	5.965880
H	-6.825865	0.977854	5.434682
H	-7.233316	-0.026894	6.810951
N	-5.201706	0.220586	6.550758
H	-5.051729	-0.110841	7.494373
C	-6.630210	-1.058557	4.970840
C	-6.853165	-0.731243	3.625197
C	-6.787380	-1.842513	2.636053
C	-6.289245	-3.378373	4.509561
C	-6.474633	-3.126595	3.165045
H	-6.373278	-3.951742	2.466792
C	-6.220928	-2.648815	6.832331
H	-6.792580	-1.940204	7.426673
H	-6.602731	-3.639226	7.068564
H	-5.165504	-2.600340	7.117934
O	-7.038579	0.471502	3.225430
O	-6.962319	-1.633007	1.411676
C	-5.956037	-4.764167	4.990814
H	-5.054902	-4.773551	5.611494
H	-6.771721	-5.203705	5.574463
H	-5.780716	-5.408925	4.128995
N	-6.396541	-2.369890	5.405348

wb97xd_L1_TS(II).log

SCF (wB97x) = -2059.25899187
E(SCF)+ZPE(0 K)= -2058.518057
H(298 K)= -2058.476353
G(298 K)= -2058.588746
Lowest Frequency = -277.2191cm-1

O	-0.947472	3.902821	6.519141
N	0.604848	2.190420	4.470678
C	-1.785851	2.521856	3.855269
H	-1.932922	3.136569	4.741688
H	-2.428575	2.911012	3.053905
C	-0.329069	2.660375	3.439999
H	-0.142194	2.102285	2.519081
H	-0.154272	3.717699	3.192392
C	1.784846	1.547750	3.863717
H	2.548547	1.449713	4.637629
H	2.207819	2.167003	3.058104
C	1.507565	0.155984	3.321728
H	0.735004	0.173920	2.548465
H	2.422355	-0.220609	2.846826
C	0.903321	-2.138122	3.973081
H	0.889166	-2.761268	4.872045
H	1.731809	-2.472582	3.337903
C	-0.420847	-2.269600	3.233125
H	-0.382292	-1.719071	2.288869
H	-0.614187	-3.317902	2.983610
C	-2.620566	-1.150259	3.302790
H	-3.526157	-1.268358	3.900509
H	-2.772352	-1.699720	2.366767
C	-2.386124	0.319970	2.978200
H	-1.484595	0.420993	2.367934
H	-3.225017	0.704187	2.383249
C	0.210261	3.488969	6.643340
C	1.067959	3.238515	5.406748
H	2.079056	2.991667	5.717792
H	1.117774	4.211560	4.894308
N	0.745472	3.407206	7.886288
H	0.077908	3.682536	8.595141
C	1.471266	1.285524	8.881353
C	0.971393	0.450638	7.911947

C	0.344302	-0.798360	8.255908
C	0.980003	-0.324123	10.560955
C	0.406625	-1.162167	9.609524
H	-0.017802	-2.104852	9.936082
O	-0.230539	-1.446079	7.305539
N	1.485996	0.885000	10.203479
C	2.068116	1.799403	11.194521
H	3.102022	2.023637	10.928425
H	1.485634	2.721426	11.236930
H	2.060688	1.343797	12.177979
C	1.895243	2.657459	8.398003
H	2.362604	3.262032	9.173958
H	2.628766	2.537402	7.600019
O	0.965647	0.797437	6.662355
N	-2.208728	1.138781	4.198796
N	1.081111	-0.745461	4.402443
H	1.781526	-0.703072	5.138210
C	-3.477322	1.207854	4.970156
H	-4.310606	0.864340	4.351303
H	-3.693723	2.240385	5.244064
C	-3.411541	0.355179	6.234606
O	-2.816848	-0.767146	6.186350
C	1.026664	-0.758856	12.000832
H	0.576123	-1.747576	12.087720
H	2.053256	-0.826790	12.372856
H	0.468489	-0.079202	12.651342
N	-1.487380	-1.701932	4.064468
H	-1.843984	-2.411660	4.695056
In	-0.742802	-0.013382	5.678271
O	-1.917908	1.229240	6.991107
H	-1.859420	2.191697	6.970277
C	-5.840791	0.612634	6.573956
H	-5.980761	1.575011	6.078371
H	-6.511885	0.612027	7.438684
N	-4.470345	0.533527	7.120411
H	-4.385150	-0.148471	7.861073
C	-6.194103	-0.429778	5.534808
C	-6.552616	0.012090	4.250255
C	-6.662543	-1.017767	3.183739
C	-6.114347	-2.727936	4.880596
C	-6.401059	-2.358536	3.581345
H	-6.435533	-3.131838	2.819387
C	-5.813183	-2.194904	7.231172
H	-6.238499	-1.460569	7.912043
H	-6.314623	-3.140158	7.429941
H	-4.742554	-2.307337	7.426041
O	-6.712433	1.251747	3.960255
O	-6.937205	-0.702587	1.996394
C	-5.875786	-4.170582	5.235728
H	-4.938525	-4.306623	5.783898
H	-6.684834	-4.583404	5.848156
H	-5.821748	-4.759913	4.319418
N	-6.047855	-1.784230	5.846122

wb97xd_L1_TS(I).log

SCF (wB97x) = -2059.25367871
E(SCF)+ZPE(0 K)= -2058.512713
H(298 K)= -2058.471059
G(298 K)= -2058.581516
Lowest Frequency = -193.9406cm-1

O	-1.892804	3.658823	6.683194
N	0.546534	2.650014	5.252326
C	-1.545401	2.464164	3.867138
H	-2.162718	2.559498	4.757078
H	-2.071457	2.968617	3.042047
C	-0.223435	3.185394	4.105996
H	0.404848	3.159470	3.211739
H	-0.461650	4.241451	4.275839
C	1.972158	2.451739	4.919736
H	2.512046	2.289104	5.857294
H	2.403455	3.349962	4.453429
C	2.233532	1.256435	4.020701
H	1.808386	1.407197	3.030712
H	3.317586	1.157272	3.886305
C	2.043548	-1.207078	3.849768
H	1.650343	-2.049312	4.429038
H	3.138595	-1.281323	3.824020
C	1.531760	-1.284673	2.413743
H	1.838080	-0.397186	1.846534
H	2.080415	-2.121562	1.958354
C	-0.670493	-0.497876	1.577676
H	-1.662550	-0.912492	1.374406
H	-0.220166	-0.332456	0.590541
C	-0.799963	0.901683	2.197095
H	0.186508	1.357642	2.232008
H	-1.396003	1.510636	1.498050
C	-0.798378	3.549514	7.223847
C	0.501776	3.537047	6.431652
H	1.317442	3.245265	7.089701
H	0.696008	4.579949	6.132229
N	-0.697067	3.592951	8.580713
H	-1.596942	3.567718	9.036756
C	0.964840	1.813123	9.293067
C	0.570824	0.937523	8.300834
C	1.282393	-0.307354	8.118985
C	2.575184	0.226888	10.099373
C	2.260309	-0.640259	9.066883
H	2.788146	-1.583587	8.987722
O	0.977493	-0.994528	7.085381
N	1.959610	1.434233	10.188397
C	2.300797	2.365350	11.272962
H	2.532969	3.349086	10.866109
H	1.470832	2.443407	11.978764
H	3.176814	2.014662	11.805577
C	0.441710	3.241530	9.408789
H	0.131445	3.449204	10.434886
H	1.264578	3.936810	9.201509
O	-0.365559	1.168991	7.427383
N	-1.409551	1.021134	3.550545
N	1.681207	0.023917	4.606514
H	2.149496	-0.110894	5.502911
C	-2.751133	0.398363	3.479996
H	-2.593270	-0.623677	3.156781
H	-3.342457	0.907133	2.703781
C	-3.656402	0.395579	4.734960
O	-4.333091	1.421296	5.008475
C	3.606195	-0.171913	11.119768
H	3.943888	-1.185378	10.902623
H	4.482236	0.483157	11.095486
H	3.198806	-0.164881	12.134827
N	0.098411	-1.499085	2.281805
H	-0.327609	-1.753550	3.171084
In	-0.380248	0.090316	5.593387
O	-2.391298	0.310403	6.033706
H	-2.564481	1.096049	6.560765
C	-3.717842	-2.158407	4.792462
H	-3.123374	-2.193154	3.879420
H	-4.537948	-2.864203	4.630046
N	-4.323093	-0.840420	4.893690
H	-5.029947	-0.746173	5.609652
C	-2.811097	-2.747044	5.865464
C	-1.440637	-2.682965	5.703666
C	-0.590697	-3.693885	6.337334
C	-2.600106	-4.371198	7.605188
C	-1.251914	-4.455418	7.356482
H	-0.657987	-5.155858	7.935123
C	-4.828976	-3.508762	7.057731
H	-5.338297	-4.247151	6.431546
H	-5.061024	-3.709437	8.100645
H	-5.211501	-2.516604	6.831511
O	-0.897454	-1.801365	4.875889
O	0.593914	-3.891578	5.990240
C	-3.237067	-5.200291	8.686964
H	-3.643865	-4.575980	9.489351
H	-4.048145	-5.824385	8.300639
H	-2.484293	-5.856325	9.125088
N	-3.379403	-3.545819	6.851146

wb97xd_L2_Ia.log

SCF (wB97x) = -2059.28308859
E(SCF)+ZPE(0 K)= -2058.540748
H(298 K)= -2058.498546
G(298 K)= -2058.609128
Lowest Frequency = 34.6978cm-1

O	0.893717	4.802833	5.996703
N	1.335682	2.136922	4.373321
C	-0.939594	3.046557	3.885211
H	-0.935951	3.626951	4.808005
H	-1.504538	3.592643	3.117859
C	0.502070	2.878705	3.418139
H	0.514522	2.353040	2.457063
H	0.916874	3.878276	3.229602
C	2.161436	1.128850	3.699146
H	2.945991	0.816545	4.392225
H	2.659026	1.535313	2.803673
C	1.376735	-0.110035	3.313182
H	0.603014	0.119976	2.577957
H	2.063091	-0.822401	2.837552
C	0.203949	-2.056622	4.228694
H	0.079353	-2.571067	5.180935
H	0.888241	-2.642893	3.601751
C	-1.146592	-1.941540	3.540729
H	-1.020937	-1.472088	2.560357
H	-1.545140	-2.946418	3.357766
C	-2.868103	-0.214260	3.485517
H	-3.712798	0.143189	4.078069
H	-3.271826	-0.737446	2.605983
C	-2.085564	1.008999	3.024508
H	-1.239399	0.734084	2.391133
H	-2.752481	1.627749	2.410045
C	1.608253	3.843824	6.285574
C	2.221192	2.967914	5.196866
H	2.935423	2.291670	5.652657
H	2.782308	3.674446	4.559630
N	2.083787	3.706292	7.555881
H	1.646700	4.361898	8.188510
C	1.927443	1.556548	8.820063
C	1.372805	0.621325	7.964951
C	0.240539	-0.154910	8.425917
C	0.591888	0.775875	10.640755
C	-0.061207	-0.108319	9.790974
H	-0.877326	-0.705329	10.182747
O	-0.451395	-0.768020	7.528606
N	1.533548	1.618459	10.148163
C	2.182488	2.611928	11.011772
H	3.251854	2.405277	11.085851
H	2.029191	3.614703	10.610743
H	1.755038	2.582326	12.007655
C	2.815421	2.609653	8.198796
H	3.500559	3.060637	8.917573
H	3.434969	2.112974	7.454342
O	1.670448	0.527884	6.712988
N	-1.599559	1.762153	4.185426
N	0.757639	-0.725390	4.498169
H	1.475028	-0.798384	5.220950
C	-3.050771	-1.976385	5.081634
H	-3.865283	-1.326855	5.376845
H	-3.459002	-2.763654	4.428795
C	-2.473848	-2.686856	6.301278
O	-1.612127	-3.558574	6.172310
C	0.224447	0.812930	12.099907
H	-0.503600	0.025093	12.297274
H	1.087357	0.634258	12.748283
H	-0.229384	1.767009	12.386664
N	-2.091998	-1.138449	4.339125
H	-2.403446	1.952286	4.788226
In	-0.565394	0.598672	5.838091
O	-0.460371	2.313006	6.879653
H	-1.314206	2.753591	6.933734
C	-3.990211	-1.433037	7.906403
H	-4.556785	-1.152938	7.020779
H	-4.700304	-1.942183	8.561517
N	-2.981548	-2.405798	7.515948
H	-2.501900	-2.889746	8.261485
C	-3.523381	-0.143089	8.536160
C	-3.131030	0.894144	7.701800
C	-3.166971	2.258233	8.248423
C	-3.549022	1.251753	10.468997
C	-3.342415	2.346969	9.658956
H	-3.306012	3.331410	10.114930
C	-3.882578	-1.155593	10.757362
H	-4.949046	-1.380648	10.847453
H	-3.362940	-2.019584	10.346638
H	-3.475143	-0.980894	11.749798
O	-2.842872	0.681370	6.448302
O	-3.052545	3.273211	7.508700
C	-3.685206	1.411936	11.958643
H	-4.622260	0.989621	12.333292
H	-2.861016	0.934806	12.498911
H	-3.669338	2.473990	12.205642
N	-3.654287	0.019319	9.911711

wb97xd_L2_Ib.log

SCF (wB97x) = -2059.29543845
E(SCF)+ZPE(0 K)= -2058.554451
H(298 K)= -2058.511797
G(298 K)= -2058.624037
Lowest Frequency = 27.9728cm-1

O	-0.001020	4.117380	6.525161
N	1.147430	2.024411	4.569004
C	-1.146563	2.845525	3.971488
H	-1.195227	3.653087	4.701367
H	-1.742812	3.132848	3.098569
C	0.302901	2.648326	3.531079
H	0.304388	1.993550	2.657567
H	0.724280	3.609706	3.207623
C	2.193638	1.179645	3.943379
H	2.890101	0.888308	4.731061
H	2.750934	1.747585	3.184450
C	1.648510	-0.089508	3.308630
H	0.929996	0.133085	2.515471
H	2.480084	-0.626324	2.838183
C	0.195634	-2.034342	3.648165
H	0.296173	-2.943727	4.247922
H	0.600558	-2.255957	2.653905
C	-1.256916	-1.618744	3.500776
H	-1.320257	-0.768805	2.821148
H	-1.836975	-2.434035	3.049340
C	-3.073284	-0.398488	4.543266
H	-3.541130	-0.216238	5.511194
H	-3.779196	-0.962317	3.917599
C	-2.750317	0.934794	3.863656
H	-2.438435	0.802617	2.824623
H	-3.670684	1.531959	3.840980
C	1.039054	3.479210	6.675578
C	1.828583	3.001592	5.457895
H	2.765938	2.559378	5.780656
H	2.070111	3.912354	4.891695
N	1.555115	3.331986	7.925038
H	0.952260	3.746789	8.623481
C	2.002575	1.166895	8.987339
C	1.465574	0.338108	8.035719
C	0.566226	-0.729552	8.401352
C	1.052167	-0.154944	10.722101
C	0.407680	-0.944953	9.782036
H	-0.251477	-1.731187	10.129222
O	-0.054032	-1.318365	7.456193
N	1.830009	0.890181	10.332680
C	2.510559	1.747432	11.310474
H	3.565405	1.836695	11.049414
H	2.049076	2.737066	11.335669
H	2.447842	1.311039	12.301361
C	2.600082	2.456726	8.464221
H	3.135195	3.022461	9.226132

H	3.316551	2.222587	7.677390
O	1.601125	0.590249	6.764210
N	-1.684894	1.627200	4.591841
N	0.979171	-0.961396	4.293312
H	1.693136	-1.434132	4.858098
C	-2.178999	-2.461804	5.542567
H	-1.318990	-3.127910	5.461880
H	-3.019670	-2.974336	5.046097
C	-2.578130	-2.319850	7.005336
O	-3.148260	-1.322052	7.449115
C	0.878118	-0.451590	12.186975
H	0.184442	-1.285453	12.300296
H	1.823254	-0.739438	12.658586
H	0.468365	0.402183	12.733896
N	-1.862119	-1.220451	4.798222
H	-2.039867	1.854127	5.525208
In	-0.234587	0.210817	5.808419
O	-1.465813	1.133447	7.184546
H	-1.999803	0.456785	7.611292
C	-1.589024	-4.617572	7.530715
H	-1.776445	-4.978027	6.519066
H	-1.974046	-5.380547	8.210949
N	-2.401951	-3.413775	7.779886
H	-2.759375	-3.284459	8.715182
C	-0.098884	-4.408382	7.644428
C	0.645961	-4.303329	6.465380
C	1.976856	-3.665053	6.579763
C	1.763064	-3.842206	9.028784
C	2.498067	-3.556312	7.895095
H	3.511171	-3.184132	8.014979
C	-0.340425	-4.535636	10.079258
H	-1.238454	-3.916499	10.110850
H	-0.630170	-5.589286	10.068766
H	0.219215	-4.344558	10.988974
O	0.171197	-4.615383	5.311966
O	2.590576	-3.237072	5.560785
C	2.400978	-3.738411	10.388218
H	1.901063	-3.002356	11.025031
H	2.406231	-4.698620	10.914191
H	3.437019	-3.418856	10.268388
N	0.468156	-4.214020	8.904589

wb97xd_L2_III.log

SCF (wB97x) = -2059.28520924
E(SCF)+ZPE(0 K)= -2058.543893
H(298 K)= -2058.501121
G(298 K)= -2058.616234
Lowest Frequency = 15.1008cm-1

O	0.197238	1.461493	8.004035
N	0.966756	2.721060	5.690629
C	-1.502836	2.714143	5.473595
H	-1.638876	3.016651	6.514149
H	-2.334675	3.128740	4.896264
C	-0.185754	3.244686	4.925954
H	-0.069321	2.937872	3.884215
H	-0.190732	4.340732	4.935493
C	2.217775	2.803664	4.901050
H	3.050266	2.687035	5.598582
H	2.314525	3.787668	4.427652
C	2.316048	1.695609	3.862092
H	1.551367	1.786104	3.085121
H	3.290806	1.762425	3.366212
C	2.074517	-0.778408	3.684348
H	2.479691	-1.620932	4.251244
H	2.689868	-0.660786	2.786378
C	0.631779	-1.048358	3.272785
H	0.271268	-0.227983	2.646836
H	0.573740	-1.964660	2.674165
C	-1.662730	-0.821220	4.098177
H	-2.320076	-1.302921	4.825221
H	-1.911449	-1.229315	3.112967
C	-1.913112	0.679986	4.122615
H	-1.338789	1.186608	3.343004
H	-2.970473	0.876364	3.922335
C	1.013136	2.409297	8.125136
C	1.120999	3.408437	6.989758
H	2.034757	4.006105	7.022558
H	0.291151	4.105940	7.152088
N	1.725569	2.558388	9.224965
H	1.526621	1.878460	9.946814
C	4.014901	2.983004	8.359500
C	4.445215	3.963300	7.454652
C	5.373201	3.524238	6.372925
C	5.150820	1.213347	7.228849
C	5.615082	2.122326	6.297162
H	6.196559	1.751216	5.458474
O	5.840305	4.342020	5.543973
N	4.418494	1.646009	8.278919
C	4.055068	0.706410	9.340891
H	3.930288	1.239873	10.280784
H	3.137973	0.171154	9.085213
H	4.855671	-0.015651	9.488379
C	2.955659	3.365781	9.359341
H	3.261968	3.266704	10.403173
H	2.733727	4.420468	9.189004
O	4.015531	5.169572	7.484999
N	-1.519473	1.237183	5.434454
N	2.150882	0.410437	4.547309
H	2.923551	0.276746	5.197858
C	-0.158471	-2.445211	5.107051
H	0.894551	-2.729811	5.181347
H	-0.667449	-3.235866	4.540435
C	-0.692035	-2.343362	6.526621
O	-0.877477	-1.213080	7.035805
C	5.412449	-0.259337	7.076744
H	5.884897	-0.442260	6.110279
H	6.079348	-0.651652	7.851659
H	4.468354	-0.814125	7.114754
N	-0.258513	-1.137734	4.451299
H	-2.191681	0.908682	6.124038
In	0.453545	0.356461	6.129073
O	1.977286	-0.672377	6.967444
H	1.799932	-1.060738	7.823885
C	-0.638574	-4.830822	6.778341
H	-1.275342	-5.100935	5.932593
H	-0.927911	-5.468684	7.616670
N	-0.935351	-3.450108	7.208456
H	-1.264674	-3.303807	8.153157
C	0.779319	-5.003215	6.311171
C	0.985013	-5.370447	4.973451
C	2.370438	-5.220030	4.438534
C	3.055248	-4.368101	6.659111
C	3.325318	-4.660118	5.335475
H	4.326526	-4.471876	4.959566
C	1.557381	-4.383736	8.580548
H	1.337173	-3.338077	8.813606
H	0.717478	-5.001367	8.890344
H	2.422292	-4.700654	9.160445
O	0.032514	-5.723708	4.198205
O	2.643432	-5.515542	3.250373
C	4.128712	-3.837259	7.568589
H	3.795413	-2.950963	8.116466
H	4.450153	-4.585523	8.301224
H	4.999629	-3.559916	6.973213
N	1.813924	-4.573266	7.151241

wb97xd_L2_II.log

SCF (wB97x) = -2059.29434460
E(SCF)+ZPE(0 K)= -2058.552865
H(298 K)= -2058.510228
G(298 K)= -2058.624312
Lowest Frequency = 17.7424cm-1

O	0.410430	1.913363	8.158762
N	1.207385	2.724276	5.692982
C	-1.212801	2.856031	5.331999
H	-1.324810	3.229599	6.353329

H	-1.993532	3.325467	4.722658
C	0.161378	3.213850	4.783381
H	0.286859	2.745791	3.805620
H	0.246969	4.297516	4.628345
C	2.500815	2.500197	5.034744
H	3.238119	2.296922	5.818890
H	2.838962	3.396626	4.495142
C	2.453858	1.319615	4.084719
H	1.783440	1.509860	3.243142
H	3.453527	1.164972	3.662029
C	1.751600	-1.008825	3.838702
H	1.957151	-1.937110	4.369719
H	2.448087	-0.937017	2.994919
C	0.324675	-1.009888	3.303549
H	0.120633	-0.042500	2.840488
H	0.246365	-1.753500	2.495126
C	-1.966294	-0.648677	4.057298
H	-2.671029	-1.006179	4.811053
H	-2.347799	-0.957305	3.068743
C	-1.916258	0.873835	4.095477
H	-1.313564	1.272011	3.274870
H	-2.934164	1.254299	3.943992
C	1.225566	2.850519	8.125148
C	1.370305	3.625838	6.836020
H	2.293676	4.205459	6.771525
H	0.553713	4.356461	6.863416
N	1.906054	3.227759	9.202800
H	1.710841	2.688985	10.034759
C	4.214460	3.636137	8.349351
C	4.633499	4.455074	7.290761
C	5.626494	3.879438	6.343097
C	5.563531	1.790093	7.665081
C	6.003994	2.528401	6.582876
H	6.672064	2.053064	5.870676
O	6.054198	4.543471	5.365464
N	4.722492	2.351644	8.560175
C	4.324422	1.606509	9.756412
H	4.148008	2.296390	10.579603
H	3.425416	1.020736	9.548734
H	5.130292	0.943758	10.065167
C	3.079923	4.119266	9.215930
H	3.347387	4.245657	10.267946
H	2.804841	5.106222	8.840182
O	4.145249	5.621493	7.079994
N	-1.369027	1.394124	5.359665
N	1.992446	0.109436	4.781229
H	2.742039	-0.182212	5.405577
C	-0.775140	-2.711216	4.588165
H	0.193417	-3.160761	4.387021
H	-1.490458	-3.158721	3.877366
C	-1.257502	-3.111811	5.978920
O	-2.316509	-2.659151	6.418837
C	5.996505	0.361515	7.851196
H	6.548630	0.036975	6.968174
H	6.653560	0.235195	8.718344
H	5.135296	-0.301573	7.979525
N	-0.669940	-1.259403	4.354665
H	-1.997366	1.127163	6.118997
In	0.372014	0.292890	6.506753
O	-1.261806	-0.174768	7.657329
H	-1.646573	-1.016706	7.396552
C	0.790617	-4.475920	6.666663
H	1.183020	-4.375607	5.652526
H	0.844688	-5.534718	6.915086
N	-0.622933	-4.100497	6.671530
H	-1.102010	-4.303969	7.539251
C	1.618543	-3.612022	7.600316
C	1.562032	-2.256881	7.376204
C	2.308463	-1.325649	8.179324
C	3.134241	-3.270837	9.401211
C	3.093007	-1.894737	9.196428
H	3.687447	-1.265246	9.847453
C	2.446231	-5.566007	8.815344
H	1.445370	-5.935112	9.044901
H	2.825515	-6.052650	7.915430
H	3.100427	-5.817986	9.641890
O	0.848442	-1.768090	6.417549
O	2.190666	-0.082540	7.883978
C	3.996118	-3.827645	10.502212
H	3.408049	-4.374365	11.245121
H	4.770941	-4.496286	10.115679
H	4.494010	-3.003813	11.013684
N	2.408514	-4.111015	8.619672

wb97xd_L2_IVac.log

SCF (wB97x) = -1563.78299566
E(SCF)+ZPE(0 K)= -1563.220062
H(298 K)= -1563.187658
G(298 K)= -1563.280750
Lowest Frequency = 17.9551cm-1

C	-1.835157	1.859052	3.627749
H	-1.989197	2.349904	4.592164
H	-2.718331	2.059095	3.009546
C	-0.588146	2.452716	2.979795
H	-0.432853	2.065897	1.968330
H	-0.711912	3.538243	2.894437
C	1.885934	2.479395	3.269629
H	2.539762	2.675778	4.124167
H	1.831416	3.401581	2.681054
C	2.455267	1.362707	2.402844
H	1.821300	1.226824	1.523177
H	3.454223	1.634089	2.040789
C	2.461343	-1.076795	2.217449
H	2.870857	-1.942581	2.743209
H	3.095469	-0.890994	1.342883
C	1.040062	-1.396290	1.779565
H	0.610598	-0.573230	1.201784
H	1.047715	-2.277259	1.130116
C	-1.243878	-1.733557	2.666513
H	-1.704723	-2.356851	3.434588
H	-1.404925	-2.230900	1.704574
C	-1.883478	-0.352268	2.625210
H	-1.446757	0.219498	1.803588
H	-2.954811	-0.450202	2.410696
N	-1.679127	0.408373	3.878439
N	2.492091	0.081711	3.137400
C	-2.567110	-0.075159	4.950105
H	-3.543766	-0.380081	4.558858
H	-2.739767	0.738796	5.660189
O	-2.740488	-1.917725	6.442217
C	-1.972979	-1.227313	5.777105
O	-0.700073	-1.362961	5.747113
N	0.197419	-1.626700	2.967267
H	0.497423	-2.497473	3.398855
C	3.640003	0.006443	4.040803
N	4.255534	-1.141920	6.114849
H	4.554094	-0.299711	3.514940
H	3.821652	0.990289	4.480657
C	5.448235	-0.306414	6.308373
C	5.095375	1.134231	6.598686
H	6.092761	-0.424682	5.437954
H	5.976374	-0.755564	7.153182
C	4.154956	1.368944	7.605495
N	5.640541	2.149639	5.802884
C	3.729873	2.776679	7.821167
O	3.642193	0.416115	8.294672
C	5.195835	3.422812	5.930483
C	6.740701	1.826411	4.891971
C	4.258717	3.726023	6.898032
O	2.937940	3.090496	8.742253
H	3.904631	-1.586683	6.952485
C	5.728984	4.501091	5.024430
H	7.490927	1.228092	5.410995
H	6.385250	1.284324	4.010919
H	7.225249	2.736810	4.554499
H	3.917312	4.753691	6.978503
H	5.163338	5.417812	5.195004
H	6.783233	4.722899	5.221351

H	5.628636	4.234992	3.967952
N	0.570008	2.102997	3.804158
H	0.478102	2.529920	4.724013
C	3.338642	-0.949683	5.184599
O	2.220978	-1.510334	5.243591
In	0.579957	-0.090650	4.630768
O	1.198611	1.063022	6.165638
H	1.730077	0.705172	6.882810

wb97xd_L2_IVhc.log

SCF (wB97x) = -1563.79849848
E(SCF)+ZPE(0 K)= -1563.236371
H(298 K)= -1563.203718
G(298 K)= -1563.296563
Lowest Frequency = 20.5754cm-1

C	-1.880956	2.464363	3.892927
H	-2.304676	2.674324	4.880614
H	-2.509474	2.976451	3.151166
C	-0.461702	3.010984	3.856493
H	0.004216	2.845431	2.879645
H	-0.486906	4.094911	4.021280
C	1.766250	2.698711	4.879253
H	2.119299	2.646665	5.906877
H	1.902423	3.726702	4.522598
C	2.587902	1.764236	3.994959
H	2.272689	1.899229	2.957371
H	3.644208	2.039791	4.055290
C	2.532998	-0.501614	3.118517
H	2.639126	-1.539448	3.447563
H	3.444493	-0.231480	2.568713
C	1.324132	-0.419468	2.198392
H	1.173169	0.592649	1.811294
H	1.496463	-1.071000	1.333023
C	-1.123223	-0.756107	2.174500
H	-1.804968	-1.505684	2.585225
H	-0.961270	-1.008167	1.119911
C	-1.735573	0.634706	2.284860
H	-1.070873	1.360302	1.809457
H	-2.688679	0.672929	1.740021
N	-1.904715	1.007845	3.693431
N	2.390838	0.335580	4.332818
C	-3.100795	0.418147	4.291368
H	-3.313454	-0.544011	3.814964
H	-3.986692	1.049117	4.146477
O	-1.722176	-0.022254	6.200210
C	-2.920744	0.119867	5.785996
O	-3.927987	-0.006698	6.484595
N	0.126530	-0.826976	2.938970
H	0.262011	-1.777230	3.281582
O	4.388741	1.683449	6.300245
C	3.786581	0.631800	6.508257
N	3.764478	0.110727	7.768410
H	4.242891	0.728373	8.412247
C	2.733343	-0.714506	8.407233
H	2.280246	-1.359759	7.654287
H	3.224836	-1.354716	9.138088
C	1.664375	0.176289	9.002664
C	0.918292	0.880597	8.080462
C	0.051035	1.969354	8.513864
C	0.668866	1.330927	10.821386
C	-0.051541	2.101543	9.929271
H	-0.710109	2.867471	10.325790
O	1.110657	0.639374	6.818604
O	-0.520700	2.726682	7.684674
C	0.501164	1.539472	12.302840
H	-0.220533	2.339511	12.469323
H	0.123275	0.641190	12.800804
H	1.439460	1.829462	12.784980
C	2.332822	-0.436444	11.280726
H	2.143883	-1.494398	11.090754
H	3.397476	-0.224617	11.159438
H	2.059696	-0.225688	12.308911
N	1.538034	0.388427	10.365835

C	3.320096	-0.237640	5.345357
H	2.854748	-1.159055	5.696786
H	4.264505	-0.503090	4.846561
N	0.336036	2.339353	4.884004
H	-0.031517	2.578701	5.819236
In	0.097665	0.081664	5.100927
O	0.447112	-1.931226	5.354032
H	0.355822	-2.256237	6.249762

wb97xd_L2.log

SCF (wB97x) = -1982.63528477
E(SCF)+ZPE(0 K)= -1981.889982
H(298 K)= -1981.848295
G(298 K)= -1981.962630
Lowest Frequency = 9.5386cm-1

O	2.711102	3.360333	6.014414
O	3.359399	-0.304354	5.055383
N	0.212627	2.152611	5.178845
N	1.768118	-1.071191	2.726345
C	-0.928887	0.009167	5.702800
H	-0.573878	0.054810	6.739054
H	-1.918875	-0.461491	5.733692
C	-1.064203	1.431271	5.147913
H	-1.403847	1.377070	4.110813
H	-1.848061	1.979947	5.699419
C	0.341521	3.104030	4.070274
H	1.129646	3.814554	4.323601
H	-0.597089	3.669016	3.929079
C	0.721198	2.412230	2.763930
H	-0.035140	1.645686	2.522923
H	0.677980	3.152491	1.954701
C	2.478821	1.074263	1.691080
H	3.556561	0.892594	1.780335
H	2.343357	1.661114	0.773683
C	1.748137	-0.267478	1.515861
H	0.698591	-0.077638	1.270235
H	2.182252	-0.803304	0.651506
C	0.626575	-1.960873	2.895273
H	0.943711	-2.835217	3.469818
H	0.258145	-2.335170	1.927781
C	-0.503818	-1.274447	3.655529
H	-0.893162	-0.445399	3.039001
H	-1.330425	-1.982701	3.794359
C	1.879001	3.014920	6.843432
C	3.268828	-1.446889	4.603858
C	3.047708	-1.640091	3.098854
H	3.155461	-2.685263	2.776851
H	3.853169	-1.080871	2.616268
C	0.418442	2.748997	6.487240
H	-0.123551	3.707047	6.605853
H	-0.006123	2.070047	7.226157
N	2.219005	2.925316	8.156341
H	3.209119	3.032103	8.321081
N	3.332092	-2.519683	5.416474
H	3.493572	-2.315029	6.406335
C	3.285371	-3.929250	5.042471
H	4.246591	-4.256993	4.638620
H	2.533738	-4.098867	4.269723
C	2.983503	-4.779515	6.247394
C	3.996354	-5.391677	6.922484
C	3.760985	-6.237574	8.075065
C	1.385902	-5.747632	7.734059
C	2.392837	-6.382497	8.428061
H	2.133594	-7.018998	9.266229
C	1.367054	0.796052	9.102346
C	2.485340	0.068953	8.809086
C	2.438986	-1.351716	8.563602
C	0.045114	-1.195317	9.021758
C	1.154161	-1.934496	8.674407
H	1.048793	-2.996788	8.491722
O	5.285746	-5.243880	6.553281
H	5.785191	-5.787702	7.186695
O	3.508687	-1.943792	8.256305

O	4.745508	-6.767799	8.643376
N	1.674957	-4.935734	6.677274
N	0.153604	0.144395	9.258233
C	0.610302	-4.241662	5.940014
H	-0.293350	-4.207924	6.540260
H	0.899528	-3.208780	5.736806
H	0.386444	-4.766752	5.007304
C	-1.011087	0.915436	9.718418
H	-0.722917	1.564043	10.544155
H	-1.436371	1.508674	8.904218
H	-1.775275	0.241048	10.088237
C	1.454362	2.309406	9.227170
H	0.469017	2.768813	9.272150
H	1.953655	2.568582	10.164362
O	3.699750	0.645412	8.712306
H	4.296738	-0.079914	8.458269
N	-0.016548	-0.843582	4.957191
H	0.850547	-0.330864	4.812838
N	2.071265	1.874657	2.833854
H	2.163179	1.311648	3.675547
C	-1.300659	-1.855665	9.124330
H	-1.220049	-2.883090	8.771415
H	-1.662690	-1.887681	10.156295
H	-2.048933	-1.349129	8.508860
C	-0.049000	-5.960518	8.130575
H	-0.653176	-6.313883	7.290895
H	-0.093305	-6.709226	8.921093
H	-0.508696	-5.046639	8.519242

wb97xd_L2_TS(Ib).log

SCF (wB97x) = -2059.26556141
E(SCF)+ZPE(0 K)= -2058.524643
H(298 K)= -2058.483298
G(298 K)= -2058.591342
Lowest Frequency = -239.0764cm-1

O	0.491967	4.218601	6.833854
N	1.111640	1.774188	4.632509
C	-1.189210	2.734906	4.527865
H	-1.030437	3.360733	5.405878
H	-1.887694	3.237286	3.850305
C	0.141983	2.534142	3.813283
H	-0.023628	1.983795	2.883102
H	0.558339	3.511685	3.539560
C	2.103835	1.083952	3.773154
H	2.928181	0.767459	4.416035
H	2.509552	1.767648	3.016930
C	1.524986	-0.150705	3.102430
H	0.728161	0.113810	2.401902
H	2.311758	-0.641083	2.519250
C	0.171036	-2.159465	3.488494
H	0.301635	-3.065240	4.089028
H	0.544459	-2.378292	2.481187
C	-1.290968	-1.765054	3.394814
H	-1.400401	-0.914896	2.718652
H	-1.865854	-2.589776	2.951704
C	-3.068788	-0.566056	4.498975
H	-3.599908	-0.513265	5.449215
H	-3.733874	-1.041021	3.763548
C	-2.712425	0.839831	4.035959
H	-2.261843	0.851932	3.040413
H	-3.627682	1.438681	3.976920
C	1.122020	3.116468	6.886114
C	1.848543	2.617272	5.606544
H	2.709796	2.028767	5.908985
H	2.212277	3.521693	5.101335
N	2.006526	3.022759	8.019237
H	1.630171	3.576631	8.776743
C	1.998083	0.738694	9.008800
C	1.406355	-0.093597	8.079462
C	0.133259	-0.669011	8.429530
C	0.521447	-0.012513	10.728885
C	-0.241263	-0.670343	9.777758
H	-1.166810	-1.146476	10.079084
O	-0.637545	-1.018465	7.455319
N	1.598115	0.713246	10.333024
C	2.369250	1.509269	11.296783
H	3.433935	1.330433	11.150115
H	2.151356	2.572484	11.173045
H	2.116769	1.218018	12.310679
C	2.818868	1.886626	8.461238
H	3.537778	2.276017	9.183027
H	3.400571	1.503868	7.623052
O	1.760091	-0.129740	6.823977
N	-1.757171	1.441688	4.977541
N	0.971696	-1.082620	4.104257
H	1.746533	-1.550682	4.593136
C	-2.170859	-2.654445	5.429445
H	-1.278159	-3.279134	5.401224
H	-2.950416	-3.199673	4.869055
C	-2.709613	-2.563552	6.854042
O	-3.538121	-1.722745	7.202441
C	0.139329	-0.075059	12.181427
H	-0.761313	-0.680876	12.284289
H	0.923790	-0.542084	12.785648
H	-0.069462	0.914914	12.596562
N	-1.861865	-1.395023	4.712864
H	-2.247279	1.625224	5.849262
In	-0.070257	0.087415	5.749355
O	-0.072645	1.893504	7.042404
H	-0.489988	2.047004	7.891685
C	-1.369065	-4.639014	7.529770
H	-1.510596	-5.125722	6.563952
H	-1.611984	-5.381781	8.293575
N	-2.362870	-3.566547	7.693065
H	-2.843098	-3.513136	8.579179
C	0.076010	-4.209858	7.576700
C	0.800718	-4.197957	6.378482
C	2.107329	-3.505319	6.399171
C	1.910873	-3.370535	8.851418
C	2.626462	-3.204925	7.683294
H	3.616488	-2.763760	7.742647
C	-0.142278	-4.031669	10.012520
H	-1.124831	-3.573445	9.911260
H	-0.256240	-5.098921	10.222368
H	0.347463	-3.559692	10.858151
O	0.333771	-4.669679	5.276655
O	2.708562	-3.206841	5.327171
C	2.538764	-3.026803	10.174835
H	2.011200	-2.209228	10.676347
H	2.564472	-3.883022	10.856177
H	3.566253	-2.702772	10.005015
N	0.637860	-3.826890	8.793264

wb97xd_L2_TS(III).log

SCF (wB97x) = -2059.25365924
E(SCF)+ZPE(0 K)= -2058.513073
H(298 K)= -2058.471157
G(298 K)= -2058.584594
Lowest Frequency = -238.0318cm-1

O	-0.282696	2.061524	7.750708
N	0.971473	2.637011	5.288130
C	-1.454935	3.003569	4.965448
H	-1.551191	3.535403	5.913660
H	-2.197375	3.408209	4.271176
C	-0.054540	3.203317	4.392820
H	0.026677	2.705636	3.422462
H	0.119284	4.271887	4.220586
C	2.232391	2.317927	4.579983
H	3.020787	2.259145	5.332097
H	2.492807	3.113605	3.872780
C	2.150164	0.982854	3.849082
H	1.426611	1.012685	3.030288
H	3.124520	0.757070	3.404579
C	1.432068	-1.372462	4.144484
H	1.625747	-2.171798	4.864280
H	2.077580	-1.559356	3.280980

C	-0.027025	-1.392901	3.712754
H	-0.204982	-0.614547	2.967059
H	-0.260493	-2.354922	3.241623
C	-2.255667	-0.672821	4.426798
H	-2.956450	-0.829089	5.250535
H	-2.616789	-1.251156	3.568876
C	-2.238250	0.811125	4.086352
H	-1.604684	1.022156	3.222552
H	-3.251516	1.131234	3.825223
C	0.910506	2.616957	7.748414
C	1.217315	3.459215	6.501281
H	2.241068	3.842606	6.501615
H	0.528843	4.308944	6.522647
N	1.328474	3.149736	8.948713
H	0.948304	2.647630	9.738260
C	3.817462	2.873132	8.730080
C	4.534718	3.221693	7.576896
C	5.459868	2.200915	7.016659
C	4.811512	0.698802	8.868517
C	5.514842	0.958064	7.710147
H	6.147042	0.174435	7.302464
O	6.114454	2.419705	5.965288
N	3.997222	1.649849	9.386025
C	3.340691	1.403905	10.673587
H	3.059702	2.347010	11.133296
H	2.446689	0.782199	10.559966
H	4.027387	0.902783	11.354419
C	2.658782	3.748921	9.137071
H	2.695067	4.085307	10.176659
H	2.737482	4.647502	8.523564
O	4.368955	4.336400	6.962648
N	-1.730567	1.572750	5.242963
N	1.756050	-0.089080	4.790492
H	2.534194	-0.225715	5.431684
C	-1.010154	-2.349383	5.715594
H	-0.757766	-3.264626	5.167004
H	-2.038154	-2.480226	6.070108
C	-0.198841	-2.175605	6.981640
O	-0.043099	-1.021433	7.458410
C	4.942747	-0.623795	9.574459
H	5.492285	-1.317233	8.936276
H	5.492604	-0.531913	10.517583
H	3.968985	-1.069364	9.799905
N	-0.924494	-1.149271	4.865929
H	-2.440615	1.540721	5.971092
In	0.125787	0.661398	6.190814
O	1.876194	1.242324	7.310759
H	2.174689	0.809534	8.113432
C	0.470837	-4.572839	7.048689
H	-0.483236	-4.950961	6.680105
H	0.767745	-5.208845	7.884926
N	0.285484	-3.222365	7.616567
H	0.734851	-3.010432	8.497586
C	1.427081	-4.601735	5.887894
C	0.915756	-4.943748	4.626676
C	1.867933	-4.925667	3.481258
C	3.618836	-4.150100	5.047776
C	3.191908	-4.497620	3.781735
H	3.897786	-4.420353	2.960401
C	3.219469	-3.862853	7.435145
H	3.052106	-2.798280	7.626040
H	2.701793	-4.457175	8.185121
H	4.279858	-4.075236	7.542015
O	-0.320134	-5.216848	4.430423
O	1.498585	-5.239083	2.324446
C	5.024139	-3.661538	5.274353
H	5.040250	-2.689193	5.776768
H	5.612649	-4.362179	5.875833
H	5.523082	-3.552173	4.310932
N	2.763697	-4.233248	6.093567

wb97xd_L2_TS(II).log

SCF (wB97x) = -2059.25473494
E(SCF)+ZPE(0 K)= -2058.513856
H(298 K)= -2058.472094
G(298 K)= -2058.584627
Lowest Frequency = -281.2686cm-1

N	0.026257	1.573862	4.893727
C	-2.150915	1.583390	3.764730
H	-2.628015	2.072757	4.617162
H	-2.683867	1.902113	2.860531
C	-0.689064	2.032370	3.695047
H	-0.189738	1.626567	2.810190
H	-0.655001	3.124091	3.611428
C	1.492244	1.739075	4.884844
H	1.802966	1.893556	5.916819
H	1.771720	2.631136	4.312551
C	2.223052	0.536450	4.292117
H	1.927918	0.436785	3.244132
H	3.300228	0.731756	4.308020
C	2.044547	-1.859087	3.992786
H	2.107111	-2.792370	4.560641
H	2.975734	-1.767703	3.416945
C	0.858586	-1.956210	3.056989
H	0.790736	-1.090497	2.393662
H	0.977221	-2.838302	2.417657
C	-1.588828	-2.040393	3.009601
H	-2.370295	-2.569715	3.555386
H	-1.408146	-2.574985	2.070884
C	-2.022375	-0.614270	2.711330
H	-1.238966	-0.100552	2.148405
H	-2.919896	-0.617689	2.080155
N	-2.259945	0.123797	3.961369
C	-3.512197	-0.296783	4.626144
H	-4.086003	-0.959553	3.968174
H	-4.137526	0.572282	4.843413
C	-3.204683	-1.074853	5.913020
O	-2.188571	-1.878371	5.914554
N	-0.373853	-2.039825	3.849544
H	-0.362137	-2.917635	4.362881
In	-0.599894	-0.432850	5.589606
O	-2.268330	0.284811	6.759435
H	-2.517815	1.208736	6.847565
C	-5.395816	-0.521450	6.927449
H	-5.026629	0.497502	7.072795
H	-5.844049	-0.833403	7.875127
N	-4.272913	-1.431369	6.677379
H	-3.997226	-2.007148	7.459521
C	-6.406684	-0.454274	5.814269
C	-6.574726	0.762556	5.135910
C	-7.524665	0.774220	3.993777
C	-7.953734	-1.625185	4.420795
C	-8.157314	-0.465806	3.697894
H	-8.835442	-0.498209	2.850022
C	-6.894588	-2.838352	6.253591
H	-6.752719	-2.597139	7.304884
H	-7.767083	-3.482249	6.183640
H	-6.014043	-3.374909	5.892231
O	-5.909678	1.821614	5.430591
O	-7.732561	1.818256	3.321900
C	-8.661177	-2.897677	4.036624
H	-7.961915	-3.724339	3.878162
H	-9.386651	-3.211153	4.794867
H	-9.206577	-2.734510	3.106197
N	-7.113202	-1.613610	5.479906
N	1.910444	-0.749366	4.960629
O	3.849740	0.935271	6.732453
C	3.236889	-0.064763	7.095263
N	3.177080	-0.377846	8.423968
H	3.652265	0.330355	8.970343
C	2.076307	-1.020677	9.152597
H	1.632864	-1.799201	8.533786
H	2.498762	-1.500521	10.033167
C	1.035429	0.031070	9.470341
C	0.375665	0.547532	8.372677
C	-0.303721	1.832650	8.488171
C	0.152286	1.691228	10.914806
C	-0.433064	2.303150	9.827736

H	-0.992096	3.218765	9.988818
O	0.565319	-0.057892	7.222169
O	-0.708382	2.489286	7.489591
C	-0.031489	2.258926	12.295953
H	-0.657922	3.148972	12.233451
H	-0.527025	1.548318	12.964543
H	0.920595	2.549847	12.749398
C	1.597894	-0.077926	11.854060
H	1.337530	-1.136720	11.883806
H	2.680222	0.035282	11.759735
H	1.288700	0.368689	12.792688
N	0.912544	0.576500	10.734385
C	2.795522	-1.120409	6.093751
H	2.322200	-1.956672	6.606671
H	3.753191	-1.485003	5.688487
H	-0.318497	2.078635	5.727463

wb97x_H2O.log

SCF (wB97x) = -76.4407476727
E(SCF)+ZPE(0 K)= -76.419161
H(298 K)= -76.415381
G(298 K)= -76.437450
Lowest Frequency = 1605.7256cm-1

O	-0.209490	1.498721	0.000000
H	0.749755	1.532949	0.000000
H	-0.497431	2.414375	0.000000

wb97x_HOPO.log

SCF (wB97x) = -572.001780158
E(SCF)+ZPE(0 K)= -571.798577
H(298 K)= -571.785882
G(298 K)= -571.835558
Lowest Frequency = 82.4458cm-1

C	-2.173167	-2.332360	-0.120864
C	-0.815264	-2.242296	-0.189726
C	-0.113496	-0.976944	-0.124839
C	-0.955628	0.158904	0.011517
C	-2.325762	0.043254	0.090216
H	-0.504892	1.145258	0.051129
N	-2.924885	-1.179197	0.046686
C	-4.389073	-1.295130	0.106135
H	-4.648839	-2.223295	0.612541
H	-4.800202	-0.471185	0.684113
H	-4.816127	-1.276745	-0.901098
C	-3.182974	1.271738	0.219483
H	-2.551971	2.158095	0.144443
H	-3.939241	1.320551	-0.569337
H	-3.697000	1.304393	1.185457
C	-2.857580	-3.673133	-0.210171
H	-2.146208	-4.368151	-0.669423
H	-3.718995	-3.607843	-0.882113
O	-0.031011	-3.340587	-0.334103
H	0.875665	-2.988023	-0.363451
O	1.141046	-0.977912	-0.198879
N	-3.328614	-4.112883	1.107027
H	-3.836194	-4.985558	1.017049
H	-2.537932	-4.294999	1.715170

wb97x_InOH3.log

SCF (wB97x) = -229.471268867
E(SCF)+ZPE(0 K)= -229.432478
H(298 K)= -229.424444
G(298 K)= -229.464062
Lowest Frequency = 125.4379cm-1

In	0.745382	-0.634580	0.063364
O	0.055688	1.103083	-0.460701
H	-0.883186	1.206347	-0.625605
O	2.644987	-0.868804	0.388054
H	3.235796	-0.121300	0.280746
O	-0.469265	-2.136078	0.263177
H	-0.123061	-2.991656	0.522936

wb97x_L1_III.log

SCF (wB97x) = -2059.39904468
E(SCF)+ZPE(0 K)= -2058.656849
H(298 K)= -2058.613170
G(298 K)= -2058.733787
Lowest Frequency = 9.6324cm-1

O	-1.717232	2.859972	5.713716
N	-0.257634	2.239271	3.510925
C	-2.602960	1.883472	2.754923
H	-2.905900	2.852971	3.159440
H	-3.217561	1.704548	1.864429
C	-1.133621	1.926829	2.358909
H	-0.820209	0.968919	1.934141
H	-1.000826	2.675493	1.566713
C	1.152312	1.891326	3.210644
H	1.778194	2.390024	3.956982
H	1.445294	2.272739	2.223827
C	1.401432	0.392047	3.291859
H	0.880392	-0.151382	2.497496
H	2.472974	0.201997	3.157581
C	0.909871	-1.564956	4.726549
H	1.000351	-1.798540	5.791803
H	1.758391	-2.026080	4.207644
C	-0.398583	-2.121708	4.184776
H	-0.481835	-1.921710	3.111119
H	-0.431745	-3.208776	4.314079
C	-2.815042	-1.624455	4.146705
H	-3.630467	-1.650447	4.875189
H	-2.853093	-2.578260	3.607588
C	-3.007718	-0.487970	3.147884
H	-2.258684	-0.565912	2.354487
H	-3.992723	-0.574482	2.670735
C	-1.211976	3.850300	5.131731
C	-0.365893	3.657569	3.891478
H	0.616626	4.083392	4.143231
H	-0.779640	4.252977	3.068550
N	-1.410584	5.068859	5.604275
H	-1.954927	5.103943	6.457252
C	0.234447	6.834938	6.141393
C	1.488171	6.221849	6.136378
C	2.509461	6.754935	7.075287
C	0.803664	8.360267	7.882638
C	2.075840	7.821448	7.913747
H	2.792947	8.233027	8.619135
O	3.672534	6.283119	7.114759
N	-0.111691	7.873817	7.015947
C	-1.459556	8.441312	6.941575
H	-1.593325	9.006573	6.014565
H	-2.211551	7.651380	6.994550
H	-1.629490	9.110126	7.779753
C	-0.776681	6.319821	5.155538
H	-1.570791	7.033237	4.933064
H	-0.255406	6.113716	4.220257
O	1.768913	5.241993	5.352745
N	-2.869822	0.848105	3.784041
N	0.929434	-0.099647	4.590139
H	1.513649	0.283024	5.331207
C	-4.094141	1.178645	4.545295
H	-4.999409	0.912906	3.981161
H	-4.114615	2.259786	4.723651
C	-4.046984	0.573069	5.935633
O	-2.941855	0.449748	6.509547
C	0.443155	9.491462	8.810420
H	1.320434	9.755420	9.402953
H	0.124741	10.385164	8.264577
H	-0.356955	9.214241	9.504705
N	-1.535474	-1.473812	4.868822
H	-1.636739	-1.900565	5.786378
In	-1.095807	0.795887	5.336864
O	0.017717	0.734169	7.009452

H	-0.381075	1.090407	7.804703
C	-6.488876	0.049649	5.908857
H	-6.728753	0.924693	5.303842
H	-7.217765	0.018107	6.721940
N	-5.171867	0.235673	6.548481
H	-5.052171	-0.084782	7.500608
C	-6.537068	-1.143490	4.991648
C	-6.655356	-0.914723	3.616701
C	-6.600923	-2.104323	2.722195
C	-6.323131	-3.510848	4.735133
C	-6.411679	-3.360330	3.365993
H	-6.336227	-4.246256	2.741061
C	-6.318827	-2.599226	6.993810
H	-5.277622	-2.560715	7.332044
H	-6.895934	-1.828503	7.500962
H	-6.745205	-3.556891	7.284921
O	-6.753691	0.261360	3.107084
O	-6.692714	-1.983179	1.476952
C	-6.129655	-4.875304	5.340773
H	-5.260681	-4.907674	6.005449
H	-7.005334	-5.194574	5.916043
H	-5.973898	-5.601193	4.541270
N	-6.411210	-2.429451	5.541427

wb97x_L1_II.log

SCF (wB97x) = -2059.39138915
E(SCF)+ZPE(0 K)= -2058.649297
H(298 K)= -2058.606056
G(298 K)= -2058.721277
Lowest Frequency = 22.9274cm-1

O	-0.402867	4.406362	6.390807
N	0.440047	2.041630	4.494114
C	-2.040677	2.378154	4.506816
H	-1.955225	2.941839	5.437938
H	-2.859518	2.800491	3.905623
C	-0.733290	2.532942	3.742518
H	-0.782894	2.002101	2.786398
H	-0.617399	3.597085	3.495266
C	1.394274	1.368079	3.582212
H	2.368903	1.340524	4.074883
H	1.516186	1.937696	2.649137
C	1.011215	-0.065207	3.259908
H	0.063092	-0.116741	2.716421
H	1.778567	-0.494582	2.601969
C	0.690856	-2.275529	4.302739
H	0.855635	-2.770091	5.266099
H	1.408229	-2.685908	3.580421
C	-0.734307	-2.516691	3.829117
H	-0.863579	-2.122089	2.816031
H	-0.936548	-3.592917	3.777473
C	-2.877389	-1.321633	4.064876
H	-3.743488	-1.484713	4.712145
H	-3.090160	-1.878132	3.143334
C	-2.741244	0.158778	3.711112
H	-1.968465	0.277085	2.947196
H	-3.684532	0.522799	3.277399
C	0.621699	3.727722	6.446415
C	1.203983	3.116541	5.175650
H	2.186985	2.714684	5.395640
H	1.330237	3.965204	4.484425
N	1.340966	3.674227	7.598987
H	0.863959	4.143334	8.357108
C	2.184853	1.603260	8.680211
C	1.647646	0.618035	7.884516
C	0.957064	-0.497106	8.490149
C	1.772107	0.310613	10.637311
C	1.107034	-0.645434	9.876566
H	0.659373	-1.495198	10.382430
O	0.222923	-1.224539	7.717785
N	2.268442	1.428501	10.052374
C	2.927073	2.477257	10.843371
H	3.945589	2.630738	10.481725
H	2.365142	3.410839	10.771659
H	2.974880	2.186823	11.887558
C	2.528518	2.905455	7.986225
H	3.149017	3.560088	8.599585
H	3.108984	2.671194	7.093643
O	1.576875	0.722795	6.592511
N	-2.368776	0.977910	4.884188
N	0.902490	-0.838070	4.505573
H	1.754284	-0.692749	5.045355
C	-3.507202	1.035978	5.826717
H	-4.443643	1.226645	5.287506
H	-3.326380	1.868308	6.513692
C	-3.583689	-0.176284	6.734432
O	-2.548689	-0.769432	7.076841
C	1.918764	0.108100	12.121870
H	1.461435	-0.843465	12.395800
H	2.969668	0.073573	12.425640
H	1.421422	0.896576	12.694875
N	-1.664930	-1.825272	4.725371
H	-1.928477	-2.439673	5.486922
In	-0.502515	0.118730	6.078213
O	-1.062932	1.584070	7.395605
H	-1.448191	1.203982	8.186754
C	-6.094546	-0.133919	6.696417
H	-6.146834	0.954177	6.637508
H	-6.825119	-0.442218	7.448956
N	-4.778180	-0.544454	7.212669
H	-4.742171	-1.307340	7.874691
C	-6.391166	-0.652899	5.312836
C	-6.505710	0.271847	4.268881
C	-6.662742	-0.274376	2.892738
C	-6.594832	-2.545231	3.865375
C	-6.676510	-1.693951	2.781726
H	-6.760612	-2.125566	1.787752
C	-6.425700	-2.947934	6.261298
H	-5.396502	-3.256278	6.474130
H	-6.850980	-2.467962	7.140727
H	-7.023393	-3.835473	6.063275
O	-6.426147	1.542376	4.446100
O	-6.752188	0.486689	1.897639
C	-6.632999	-4.037250	3.669114
H	-5.780531	-4.533956	4.143146
H	-7.548678	-4.479860	4.076076
H	-6.604186	-4.257117	2.600729
N	-6.482161	-2.039032	5.113474

wb97x_L1_I.log

SCF (wB97x) = -2059.39087430
E(SCF)+ZPE(0 K)= -2058.648802
H(298 K)= -2058.605467
G(298 K)= -2058.721244
Lowest Frequency = 9.4706cm-1

O	-1.646363	3.336898	7.002825
N	0.433168	2.657262	4.958037
C	-1.858010	2.489389	3.803802
H	-2.310779	2.542913	4.796334
H	-2.456261	3.145489	3.140896
C	-0.470064	3.121040	3.880001
H	0.045111	3.026745	2.919398
H	-0.646784	4.199065	3.998300
C	1.797384	2.440415	4.442296
H	2.463572	2.301815	5.296718
H	2.152148	3.318635	3.877752
C	1.900884	1.228860	3.544945
H	1.153609	1.282337	2.756754
H	2.885053	1.224857	3.058972
C	1.889220	-1.207405	3.326046
H	1.648294	-2.115319	3.883855
H	2.946914	-1.240779	3.035668
C	1.063079	-1.161596	2.038834
H	1.282845	-0.246668	1.472368
H	1.455834	-1.983162	1.420546
C	-1.293278	-0.511211	1.491746
H	-2.260598	-1.030056	1.468183

H	-0.960144	-0.480780	0.446564
C	-1.501575	0.945418	1.941707
H	-0.554551	1.477632	1.835263
H	-2.203463	1.430810	1.236225
C	-0.433706	3.409237	7.212631
C	0.533499	3.628639	6.064292
H	1.554481	3.629287	6.434612
H	0.329253	4.648917	5.703776
N	0.036446	3.423150	8.485323
H	-0.704833	3.333292	9.169236
C	1.519736	1.684643	9.354648
C	1.427130	0.777219	8.325511
C	1.199430	-0.615204	8.621345
C	1.517974	-0.068412	10.969730
C	1.305345	-1.002018	9.966291
H	1.190800	-2.047111	10.234971
O	0.889722	-1.378262	7.637991
N	1.598721	1.252178	10.669001
C	1.782103	2.262018	11.721891
H	2.652284	2.879691	11.494789
H	0.891599	2.889148	11.803899
H	1.951728	1.777880	12.677626
C	1.383256	3.147751	8.991000
H	1.566008	3.815414	9.832129
H	2.118720	3.397302	8.226485
O	1.403062	1.134841	7.064305
N	-1.972622	1.098612	3.325826
N	1.711203	-0.050856	4.266762
H	2.508221	-0.116871	4.901641
C	-3.398243	0.729010	3.390362
H	-3.579947	-0.138797	2.754610
H	-4.027377	1.548113	3.004717
C	-3.871152	0.438574	4.812842
O	-4.332390	1.334073	5.519085
C	1.637423	-0.524962	12.398575
H	1.530816	-1.609725	12.433006
H	2.612514	-0.270862	12.825585
H	0.859753	-0.089686	13.033069
N	-0.366243	-1.327267	2.251867
H	-0.583004	-1.387427	3.240005
In	0.154099	-0.164455	5.946080
O	-1.570637	0.353064	6.785466
H	-1.823415	1.279209	6.744048
C	-3.175730	-1.939533	4.528400
H	-2.395605	-1.544379	3.883622
H	-3.919105	-2.429865	3.888245
N	-3.795691	-0.835658	5.256469
H	-3.839330	-0.908441	6.263548
C	-2.505557	-2.964250	5.402978
C	-1.133889	-2.929473	5.538823
C	-0.439478	-4.103686	6.089244
C	-2.677632	-5.031887	6.576281
C	-1.308117	-5.094900	6.651928
H	-0.850431	-5.957250	7.128064
C	-4.736985	-3.961876	5.790071
H	-5.211182	-4.384787	6.673917
H	-5.088116	-2.936198	5.691369
H	-5.043865	-4.534892	4.909185
O	-0.418856	-1.924479	5.053855
O	0.801338	-4.233124	6.040338
C	-3.532991	-6.119847	7.165029
H	-4.148690	-5.746458	7.990022
H	-4.201165	-6.560529	6.418858
H	-2.889760	-6.909337	7.556081
N	-3.278395	-3.985270	5.948497

wb97x_L1_IVac.log

SCF (wB97x) = -1563.86786113
E(SCF)+ZPE(0 K)= -1563.304430
H(298 K)= -1563.271561
G(298 K)= -1563.367393
Lowest Frequency = 9.7661cm-1

O	-0.477814	-0.877369	6.127851

N	-0.572114	1.532144	5.051147
C	-2.082434	2.054318	3.142992
H	-2.987381	1.772269	3.689039
H	-2.372813	2.860935	2.457015
C	-1.036547	2.578166	4.116055
H	-0.160768	2.941230	3.570523
H	-1.446641	3.435624	4.665840
C	0.726331	1.916424	5.663148
H	0.833495	1.380391	6.610322
H	0.731183	2.988796	5.894043
C	1.901676	1.563970	4.765795
H	1.878168	2.153008	3.844272
H	2.837028	1.812179	5.279964
C	2.763109	-0.249236	3.324957
H	2.895692	-1.333668	3.377964
H	3.744800	0.223456	3.446204
C	2.165568	0.126154	1.975240
H	2.076930	1.213172	1.876993
H	2.829931	-0.215263	1.172678
C	0.035209	0.078218	0.730082
H	-0.597025	-0.722991	0.337720
H	0.677447	0.408201	-0.095042
C	-0.815984	1.251088	1.208544
H	-0.167609	2.087888	1.486875
H	-1.462464	1.604547	0.393872
C	-1.220233	-0.083240	6.757475
C	-1.548414	1.263476	6.117198
H	-1.539708	2.073181	6.856665
H	-2.557178	1.199230	5.698858
C	-1.989792	0.468794	10.197329
C	-1.565034	-0.727111	10.770882
C	-0.955085	-0.659585	12.119034
C	-1.341279	1.787005	12.072033
C	-0.878763	0.644083	12.691551
H	-0.431639	0.735906	13.677800
O	-0.538008	-1.687573	12.707529
N	-1.898183	1.706488	10.840576
C	-2.426475	2.895770	10.169204
H	-1.831192	3.139288	9.284135
H	-3.467969	2.738414	9.879920
H	-2.395214	3.748199	10.840422
C	-2.585540	0.383446	8.809777
H	-3.547631	-0.143281	8.849751
H	-2.775111	1.355241	8.361104
O	-1.674682	-1.856518	10.147453
N	-1.617355	0.872636	2.386797
N	1.848262	0.134230	4.417359
H	2.092872	-0.405031	5.244173
C	-2.777147	0.049294	2.012473
H	-2.497193	-0.649147	1.217737
H	-3.602022	0.663379	1.630785
O	-4.415880	-1.218092	3.194226
C	-3.263542	-0.793503	3.196162
O	-2.394728	-1.014161	4.114839
C	-1.227252	3.119380	12.766188
H	-0.717864	2.977109	13.720468
H	-0.648318	3.840344	12.180494
H	-2.207977	3.559467	12.974574
N	0.825716	-0.465134	1.842618
H	0.923657	-1.473567	1.742520
In	-0.308503	-0.554459	3.921646
O	0.258188	-2.475225	3.631575
H	-0.125227	-3.129542	4.217245
N	-1.706171	-0.394087	7.937551
H	-1.463659	-1.292908	8.372275

wb97x_L1_IVhc.log

SCF (wB97x) = -1563.85322540
E(SCF)+ZPE(0 K)= -1563.289618
H(298 K)= -1563.257179
G(298 K)= -1563.348355
Lowest Frequency = 33.1142cm-1

O	-1.181498	4.276522	6.779523

N	0.723620	2.843617	4.732826
C	-1.657943	2.729855	3.979081
H	-1.978497	2.953043	4.998734
H	-2.363478	3.191307	3.271310
C	-0.279836	3.347375	3.777900
H	0.079095	3.164192	2.759606
H	-0.390587	4.437349	3.868914
C	2.026531	2.626803	4.071620
H	2.793962	2.580011	4.847928
H	2.282331	3.469336	3.410199
C	2.103935	1.331079	3.285177
H	1.396294	1.324317	2.451727
H	3.108240	1.251237	2.846565
C	2.088716	-1.111619	3.517487
H	2.079486	-1.877538	4.300797
H	3.075418	-1.127325	3.035919
C	0.992948	-1.398719	2.499608
H	1.067714	-0.700260	1.659124
H	1.122061	-2.406335	2.085302
C	-1.370135	-0.690199	2.297238
H	-2.314803	-1.171418	2.564417
H	-1.197665	-0.924883	1.238487
C	-1.487488	0.827086	2.436644
H	-0.572838	1.293593	2.063586
H	-2.312460	1.190648	1.805840
C	-0.066458	3.800951	6.991630
C	0.970394	3.751354	5.874514
H	1.931208	3.478311	6.297841
H	1.045754	4.789693	5.511100
N	0.309707	3.489389	8.260237
H	-0.456081	3.591651	8.912418
C	1.333158	1.343831	8.987842
C	1.269153	0.591225	7.838526
C	0.792625	-0.772481	7.894909
C	0.834349	-0.565712	10.321738
C	0.651064	-1.330611	9.174587
H	0.350664	-2.368257	9.282555
O	0.501568	-1.340121	6.776420
N	1.134949	0.752758	10.226851
C	1.282976	1.590362	11.424496
H	2.288053	2.015503	11.460889
H	0.543111	2.393431	11.415083
H	1.129148	0.997030	12.319628
C	1.507091	2.836511	8.798568
H	1.766476	3.351769	9.724482
H	2.337760	2.995372	8.110444
O	1.479039	1.092584	6.660784
N	-1.700259	1.252548	3.835604
N	1.834733	0.182266	4.162668
H	2.423123	0.272979	4.989524
C	-3.023982	0.776875	4.295077
H	-3.761456	0.851774	3.485878
H	-3.353830	1.421847	5.112792
O	-4.136373	-1.235021	4.886016
C	-3.042778	-0.651317	4.859418
O	-1.944811	-1.116501	5.296618
C	0.681337	-1.208568	11.674759
H	0.452294	-2.266403	11.539587
H	1.600602	-1.138242	12.264638
H	-0.132429	-0.760844	12.253359
N	-0.303424	-1.223078	3.146958
H	-0.607906	-2.085931	3.579642
In	-0.162216	0.296914	5.370978
O	-1.357957	1.189058	6.787873
H	-2.018896	0.564366	7.090333

wb97x_L1.log

SCF (wB97x) = -1982.75555026
E(SCF)+ZPE(0 K)= -1982.007008
H(298 K)= -1981.965558
G(298 K)= -1982.078657
Lowest Frequency = 17.1454cm-1

O	2.101729	0.422614	6.597199
N	0.615442	1.994837	4.892484
C	-1.863039	1.735218	4.455222
H	-2.191685	1.991762	5.467980
H	-2.636576	2.152129	3.784316
C	-0.551115	2.456738	4.144458
H	-0.319470	2.341787	3.080732
H	-0.728397	3.538327	4.302654
C	1.849606	2.576284	4.352352
H	2.617215	2.554588	5.133226
H	1.698807	3.635049	4.077853
C	2.374012	1.798692	3.150675
H	1.585985	1.748482	2.383619
H	3.203233	2.362550	2.700965
C	2.699042	-0.576176	2.559823
H	3.147104	-1.487722	2.974191
H	3.291473	-0.300750	1.677026
C	1.273445	-0.901150	2.102149
H	0.843176	-0.037973	1.563109
H	1.328168	-1.721781	1.375122
C	-0.969015	-1.504029	2.863538
H	-1.419276	-2.272132	3.507879
H	-1.032831	-1.892201	1.840109
C	-1.773925	-0.207315	2.971091
H	-1.297666	0.553468	2.343203
H	-2.796643	-0.347928	2.578837
C	1.425764	1.303697	7.122324
C	0.492971	2.207807	6.316611
H	0.668912	3.261388	6.603361
H	-0.526154	1.962925	6.633580
N	1.499712	1.510899	8.459501
H	2.125870	0.885084	8.950513
C	-0.332421	1.439456	10.090990
C	0.181684	0.428526	10.845848
C	-0.644417	-0.550786	11.516725
C	-2.543736	0.733291	10.648915
C	-2.041132	-0.335959	11.358523
H	-2.733550	-1.028395	11.825631
O	-0.087488	-1.485240	12.143201
N	-1.704740	1.616716	10.028770
C	-2.239042	2.779618	9.304515
H	-1.673655	3.672876	9.569699
H	-2.211152	2.606839	8.225331
H	-3.271341	2.947388	9.593705
C	0.628966	2.316427	9.302977
H	0.110178	3.047563	8.688415
H	1.251584	2.879016	10.006131
O	1.524451	0.263969	10.959558
H	1.639629	-0.503721	11.546338
N	-1.798298	0.280099	4.352350
N	2.833967	0.475913	3.558970
H	2.382042	0.218707	4.432223
C	-2.856947	-0.354316	5.126100
H	-2.882883	-1.426979	4.905823
H	-3.852999	0.058297	4.896447
O	-3.434970	0.497666	7.297308
C	-2.647326	-0.166186	6.624730
N	-1.543424	-0.704813	7.199800
H	-1.507720	-0.559488	8.201770
C	-0.634186	-1.697914	6.634019
H	0.391643	-1.319834	6.642207
H	-0.893222	-1.852734	5.589580
C	-0.773600	-3.017286	7.354260
C	-1.677259	-3.930844	6.906459
C	-0.242119	-4.434531	9.202012
C	-1.920330	-5.187021	7.585537
C	-1.149948	-5.373347	8.766175
H	-1.284513	-6.283751	9.341121
O	-2.422663	-3.698681	5.797745
H	-2.970156	-4.496738	5.695029
O	-2.766856	-5.978813	7.105913
N	-0.052060	-3.273036	8.510670
C	1.004660	-2.343233	8.931995
H	1.955934	-2.602903	8.458134
H	0.745467	-1.320675	8.663532
H	1.108667	-2.378007	10.013668

N	0.438875	-1.327551	3.221786
H	0.474909	-0.616994	3.951888
C	-4.029665	0.932839	10.544232
H	-4.534074	0.092609	11.023010
H	-4.348956	1.848198	11.052727
H	-4.341079	0.977693	9.497109
C	0.554239	-4.670837	10.455237
H	0.292887	-5.646618	10.866223
H	1.630460	-4.659889	10.257421
H	0.337531	-3.906412	11.210750

wb97x_L1_TS(III).log

SCF (wB97x) = -2059.35882419
E(SCF)+ZPE(0 K)= -2058.615120
H(298 K)= -2058.573603
G(298 K)= -2058.687070
Lowest Frequency = -355.1778cm-1

O	-1.999205	2.769564	5.486060
N	-0.271416	2.014310	3.392823
C	-2.653335	1.886169	2.658969
H	-2.907483	2.819161	3.164759
H	-3.287831	1.796901	1.769318
C	-1.184067	1.930910	2.231152
H	-0.922790	1.033417	1.660914
H	-1.039759	2.785971	1.558527
C	1.104485	1.556104	3.074654
H	1.775207	1.990389	3.823026
H	1.415861	1.925417	2.089709
C	1.242268	0.043956	3.135338
H	0.638837	-0.447427	2.366454
H	2.286030	-0.229011	2.942358
C	0.708765	-1.913429	4.533593
H	0.766388	-2.204338	5.586943
H	1.538947	-2.396887	4.006124
C	-0.619300	-2.359126	3.940723
H	-0.657018	-2.138341	2.869000
H	-0.737800	-3.442104	4.052223
C	-2.992281	-1.696727	3.856651
H	-3.819845	-1.718189	4.572315
H	-3.062139	-2.627256	3.282084
C	-3.132858	-0.512677	2.903779
H	-2.391874	-0.586027	2.102071
H	-4.123735	-0.536646	2.430824
C	-0.823343	3.299694	5.448158
C	-0.231389	3.355653	4.027825
H	0.787959	3.739254	4.003168
H	-0.866830	4.054199	3.473230
N	-0.584241	4.405143	6.239782
H	-1.160938	4.372398	7.070676
C	1.590262	4.447784	7.564804
C	2.472150	3.401721	7.314092
C	3.390288	2.990019	8.398156
C	2.336518	4.716542	9.819370
C	3.256544	3.709762	9.622207
H	3.913142	3.438476	10.444512
O	4.229015	2.068488	8.240162
N	1.504836	5.076001	8.812808
C	0.540380	6.163257	9.002029
H	-0.403303	5.925537	8.511700
H	0.329042	6.292939	10.060010
H	0.925035	7.106050	8.600218
C	0.751063	4.987012	6.429668
H	0.631122	6.069772	6.518940
H	1.327201	4.815939	5.520615
O	2.499780	2.777423	6.169908
N	-2.935435	0.775671	3.608797
N	0.814005	-0.445368	4.462501
H	1.502218	-0.134561	5.144522
C	-4.100010	1.079194	4.469883
H	-5.045016	0.832196	3.967522
H	-4.097817	2.152344	4.684929
C	-3.969506	0.428173	5.838915
O	-2.835389	0.225459	6.345340
C	2.259195	5.424743	11.146186
H	3.051075	5.048595	11.795713
H	2.391721	6.506026	11.041168
H	1.302653	5.248209	11.649548
N	-1.716734	-1.632991	4.601842
H	-1.868566	-2.051848	5.515734
In	-1.163103	0.664441	5.059297
O	0.063379	1.904275	6.155449
H	1.047574	2.064808	6.132836
C	-6.435275	0.053468	5.984202
H	-6.659437	0.971264	5.439346
H	-7.095013	0.032235	6.854426
N	-5.060584	0.120824	6.519441
H	-4.891187	-0.230828	7.453065
C	-6.651694	-1.085800	5.024085
C	-6.883227	-0.777332	3.679104
C	-7.030367	-1.919632	2.734182
C	-6.679937	-3.445798	4.647570
C	-6.891056	-3.217993	3.302318
H	-6.961306	-4.073994	2.636474
C	-6.369244	-2.651260	6.930940
H	-6.844862	-1.874087	7.526492
H	-6.825452	-3.595067	7.221157
H	-5.299925	-2.691008	7.164921
O	-6.927121	0.428162	3.236050
O	-7.243167	-1.728673	1.512508
C	-6.551490	-4.852107	5.169580
H	-5.627294	-4.994703	5.738270
H	-7.391336	-5.125901	5.817161
H	-6.542177	-5.544975	4.326820
N	-6.590369	-2.403998	5.503949

wb97x_L1_TS(II).log

SCF (wB97x) = -2059.37937819
E(SCF)+ZPE(0 K)= -2058.636547
H(298 K)= -2058.594776
G(298 K)= -2058.706742
Lowest Frequency = -297.5823cm-1

O	-1.978887	3.070579	6.445321
N	0.232925	2.297271	4.436968
C	-2.036930	1.584027	3.669345
H	-2.497102	2.036923	4.547454
H	-2.735828	1.697254	2.827220
C	-0.759532	2.354732	3.357916
H	-0.299823	1.972772	2.440977
H	-1.050744	3.393825	3.137229
C	1.604306	2.224062	3.904902
H	2.295379	2.435229	4.725017
H	1.775091	2.986605	3.127579
C	1.974514	0.862185	3.341169
H	1.317042	0.579079	2.513208
H	2.991164	0.923529	2.929308
C	2.377993	-1.489764	3.930225
H	2.580509	-2.095332	4.820280
H	3.308343	-1.410360	3.353676
C	1.289145	-2.147379	3.094109
H	1.147671	-1.597288	2.158131
H	1.579990	-3.168265	2.823436
C	-1.176588	-2.064051	2.998141
H	-1.979704	-2.587769	3.518764
H	-1.008943	-2.576327	2.042997
C	-1.581567	-0.620976	2.719232
H	-0.778183	-0.111110	2.177517
H	-2.465105	-0.608742	2.065706
C	-0.761674	3.202386	6.616494
C	0.155367	3.404067	5.412477
H	1.161239	3.625545	5.763502
H	-0.220680	4.317709	4.924839
N	-0.288813	3.327902	7.883271
H	-1.036474	3.264121	8.563111
C	1.210754	1.652463	8.874021
C	1.101530	0.715241	7.877725
C	1.072761	-0.692865	8.177746

C	1.453343	-0.063522	10.501268
C	1.286711	-1.037424	9.520861
H	1.310281	-2.081768	9.815334
O	0.844546	-1.497166	7.197470
N	1.397886	1.254954	10.185309
C	1.549490	2.299540	11.208000
H	2.428720	2.908952	10.989043
H	0.658027	2.929116	11.229212
H	1.675063	1.851227	12.187707
C	1.049467	3.094170	8.431820
H	1.207481	3.809564	9.239090
H	1.793150	3.309988	7.662077
O	0.957452	1.066903	6.633881
N	-1.855198	0.135799	3.960767
N	1.907480	-0.174685	4.383029
H	2.471480	0.138367	5.169115
C	-3.089376	-0.367187	4.632595
H	-3.610019	-1.078976	3.980081
H	-3.773383	0.464237	4.828688
C	-2.744028	-1.109471	5.922931
O	-1.753301	-1.930620	5.869323
C	1.688847	-0.481504	11.927961
H	1.714001	-1.570559	11.978579
H	2.643058	-0.105527	12.309485
H	0.891690	-0.133361	12.591863
N	0.027740	-2.125526	3.846483
H	-0.038320	-2.956828	4.425046
In	-0.137701	-0.392500	5.535910
O	-1.828002	0.223826	6.753633
H	-2.160749	1.131143	6.721602
C	-4.891917	-0.534285	7.058324
H	-4.497630	0.482469	7.149572
H	-5.251469	-0.828574	8.049529
N	-3.810812	-1.471676	6.718934
H	-3.479565	-2.018737	7.502191
C	-6.030486	-0.467174	6.071550
C	-6.213742	0.701391	5.323995
C	-7.360601	0.728969	4.378162
C	-7.955151	-1.545224	5.146118
C	-8.180330	-0.434518	4.356484
H	-9.030686	-0.443630	3.679436
C	-6.680974	-2.696283	6.892085
H	-6.979349	-2.435262	7.912763
H	-7.264694	-3.553604	6.569260
H	-5.629069	-2.979444	6.880397
O	-5.423791	1.715219	5.400554
O	-7.583368	1.729144	3.647154
C	-8.891752	-2.723450	5.077166
H	-8.383597	-3.634656	4.744447
H	-9.360744	-2.932243	6.044109
H	-9.684804	-2.503674	4.360505
N	-6.898742	-1.564696	5.987044

wb97x_L1_TS(I).log

SCF (wB97x) = -2059.36365027
E(SCF)+ZPE(0 K)= -2058.621038
H(298 K)= -2058.579206
G(298 K)= -2058.690184
Lowest Frequency = -222.3849cm-1

O	-1.856809	3.709890	6.681510
N	0.562697	2.673801	5.236946
C	-1.545835	2.467266	3.870682
H	-2.157946	2.566584	4.765614
H	-2.079193	2.969670	3.047163
C	-0.221568	3.193196	4.093961
H	0.399073	3.162814	3.192729
H	-0.462983	4.251050	4.256827
C	1.982554	2.464317	4.890403
H	2.535217	2.315386	5.824297
H	2.412637	3.354102	4.404102
C	2.230976	1.255798	4.004739
H	1.794676	1.394987	3.016634
H	3.314287	1.153921	3.858046
C	2.047478	-1.204521	3.843407
H	1.660996	-2.052296	4.421769
H	3.143212	-1.276048	3.810164
C	1.527086	-1.278036	2.409677
H	1.827376	-0.387889	1.841799
H	2.075295	-2.112644	1.948045
C	-0.689180	-0.503601	1.581643
H	-1.678543	-0.930695	1.385473
H	-0.245463	-0.341698	0.590094
C	-0.832053	0.899415	2.191714
H	0.145874	1.378311	2.200736
H	-1.453785	1.490028	1.497622
C	-0.757079	3.597880	7.213637
C	0.533196	3.575439	6.405550
H	1.357635	3.295080	7.059667
H	0.720285	4.615667	6.089008
N	-0.644759	3.644491	8.569947
H	-1.541893	3.634050	9.032974
C	0.999834	1.850833	9.295004
C	0.601180	0.970907	8.310935
C	1.288858	-0.292618	8.156137
C	2.568408	0.245591	10.143619
C	2.247636	-0.632107	9.121741
H	2.756631	-1.588677	9.064697
O	0.979542	-0.991317	7.128749
N	1.977803	1.465232	10.208221
C	2.329425	2.407272	11.281120
H	2.571567	3.383305	10.860038
H	1.500007	2.505018	11.986262
H	3.202775	2.052887	11.817567
C	0.498043	3.288913	9.392535
H	0.199332	3.514558	10.419058
H	1.329921	3.969653	9.170046
O	-0.324033	1.207279	7.421010
N	-1.419108	1.022157	3.555331
N	1.688414	0.024642	4.607970
H	2.174891	-0.105145	5.495819
C	-2.768630	0.413669	3.499296
H	-2.634411	-0.602713	3.144348
H	-3.369972	0.949214	2.747481
C	-3.655057	0.393860	4.767865
O	-4.329950	1.417909	5.059763
C	3.581546	-0.157686	11.180591
H	3.897700	-1.182903	10.984390
H	4.471704	0.478393	11.151724
H	3.165542	-0.120793	12.191804
N	0.092709	-1.494999	2.288448
H	-0.327315	-1.754501	3.178410
In	-0.364327	0.078481	5.618262
O	-2.374125	0.310962	6.055549
H	-2.545483	1.094406	6.588679
C	-3.719158	-2.162697	4.804064
H	-3.121758	-2.183264	3.891731
H	-4.542027	-2.862818	4.626342
N	-4.318407	-0.843394	4.929834
H	-5.019363	-0.752854	5.652737
C	-2.818520	-2.775910	5.869428
C	-1.448510	-2.705159	5.724916
C	-0.602395	-3.722453	6.358360
C	-2.627439	-4.440550	7.574590
C	-1.275277	-4.511961	7.349055
H	-0.686422	-5.224735	7.919663
C	-4.851746	-3.571534	7.020987
H	-5.354168	-4.299514	6.375936
H	-5.093447	-3.791924	8.058599
H	-5.234642	-2.575560	6.807448
O	-0.892670	-1.814508	4.907651
O	0.590167	-3.904748	6.029636
C	-3.279008	-5.302213	8.622083
H	-3.706735	-4.701346	9.431717
H	-4.078573	-5.920403	8.202581
H	-2.529089	-5.964467	9.057095
N	-3.399006	-3.601168	6.829474

wb97x_L2_Ia.log

SCF (wB97x) = -2059.39347108
E(SCF)+ZPE(0 K)= -2058.650246
H(298 K)= -2058.607441
G(298 K)= -2058.721643
Lowest Frequency = 11.0105cm-1

O	0.886958	4.850371	6.209347
N	1.298021	2.335276	4.406197
C	-1.072616	3.085396	4.191911
H	-0.994357	3.649628	5.124507
H	-1.749611	3.621478	3.510203
C	0.313181	3.011150	3.556917
H	0.252766	2.491712	2.592399
H	0.632700	4.038890	3.329495
C	2.058943	1.313148	3.686077
H	2.893988	1.007656	4.322346
H	2.488576	1.707341	2.747719
C	1.261776	0.066855	3.355329
H	0.426382	0.295426	2.690499
H	1.922503	-0.613582	2.799301
C	0.273547	-1.969990	4.243037
H	0.219961	-2.543123	5.169865
H	0.969115	-2.474589	3.557047
C	-1.112683	-1.913413	3.616770
H	-1.067329	-1.379840	2.660523
H	-1.440444	-2.936159	3.382208
C	-2.908576	-0.257543	3.795986
H	-3.719979	0.018615	4.472133
H	-3.357532	-0.696973	2.888924
C	-2.197201	1.030825	3.386842
H	-1.398134	0.831157	2.668488
H	-2.933098	1.656547	2.861163
C	1.725345	3.955725	6.323827
C	2.239127	3.228740	5.085862
H	3.116262	2.643252	5.348843
H	2.575864	4.030720	4.405613
N	2.328596	3.735263	7.521160
H	1.920868	4.294777	8.258208
C	2.536389	1.569905	8.709563
C	1.741308	0.735352	7.956339
C	0.798518	-0.141596	8.617888
C	1.804029	0.537061	10.730381
C	0.913305	-0.252521	10.012213
H	0.267086	-0.936115	10.554588
O	-0.084555	-0.718050	7.884046
N	2.573582	1.452138	10.093273
C	3.488531	2.327657	10.836982
H	4.517366	2.154963	10.514170
H	3.223236	3.374304	10.674408
H	3.423382	2.122959	11.900355
C	3.244019	2.680671	7.965604
H	4.029639	3.152497	8.557845
H	3.729017	2.243276	7.093325
O	1.691721	0.779771	6.661993
N	-1.644735	1.767636	4.535729
N	0.758007	-0.618483	4.560789
H	1.543107	-0.692341	5.206813
C	-2.913826	-2.177995	5.254456
H	-3.852060	-1.678058	5.471713
H	-3.144742	-3.067569	4.646546
C	-2.276880	-2.702754	6.541821
O	-1.267284	-3.409247	6.487611
C	1.896514	0.380336	12.225304
H	1.215886	-0.412017	12.539665
H	2.905319	0.100653	12.544672
H	1.611050	1.295405	12.753433
N	-2.067691	-1.239360	4.501264
H	-2.415427	1.948213	5.189228
In	-0.496588	0.623801	6.148864
O	-0.563476	2.272793	7.339550
H	-1.429926	2.683711	7.260535
C	-4.085775	-1.732313	8.030815
H	-4.706991	-1.718975	7.132527
H	-4.641781	-2.335430	8.748152
N	-2.863653	-2.455703	7.731655
H	-2.345191	-2.820959	8.518165
C	-3.958457	-0.294111	8.505021
C	-3.341185	0.632900	7.680550
C	-3.669437	2.052531	7.866719
C	-4.881768	1.405574	9.906990
C	-4.418502	2.365901	9.032619
H	-4.650014	3.408557	9.230564
C	-5.126537	-0.934635	10.588749
H	-6.051017	-1.405781	10.242532
H	-4.353128	-1.692206	10.718597
H	-5.308190	-0.485979	11.561075
O	-2.634918	0.261466	6.650296
O	-3.328167	2.912194	7.005468
C	-5.642298	1.811636	11.141459
H	-6.619933	1.324107	11.200982
H	-5.088870	1.577293	12.056981
H	-5.802766	2.890541	11.118755
N	-4.664783	0.092281	9.645975

wb97x_L2_Ib.log

SCF (wB97x) = -2059.40715082
E(SCF)+ZPE(0 K)= -2058.664904
H(298 K)= -2058.621538
G(298 K)= -2058.738497
Lowest Frequency = 13.2708cm-1

O	-0.272504	4.415236	6.591530
N	0.893174	2.459705	4.528858
C	-1.545264	2.903637	4.180544
H	-1.639469	3.532200	5.067803
H	-2.259811	3.258098	3.426698
C	-0.127506	3.037519	3.632406
H	-0.067195	2.525053	2.666614
H	0.078582	4.098428	3.434494
C	1.934083	1.762534	3.739637
H	2.792684	1.589179	4.393065
H	2.277191	2.386514	2.901001
C	1.481238	0.411703	3.203052
H	0.602733	0.506880	2.556319
H	2.286026	-0.013209	2.590689
C	0.708627	-1.836934	3.905201
H	0.882125	-2.529412	4.735804
H	1.285179	-2.196056	3.044228
C	-0.768174	-1.793234	3.552472
H	-0.923246	-1.113425	2.711862
H	-1.104946	-2.783521	3.220868
C	-2.825869	-0.684209	4.196776
H	-3.507345	-0.579888	5.044717
H	-3.306802	-1.329267	3.447830
C	-2.559187	0.694322	3.596654
H	-1.954451	0.640106	2.685272
H	-3.520026	1.142301	3.312926
C	0.811696	3.838282	6.679589
C	1.564551	3.450652	5.407712
H	2.543274	3.057219	5.666987
H	1.715510	4.393676	4.860364
N	1.382377	3.678959	7.903979
H	0.788802	4.041288	8.638931
C	2.127740	1.533270	8.872545
C	1.610998	0.689828	7.925202
C	0.938927	-0.531052	8.296423
C	1.626244	-0.038105	10.585091
C	1.016891	-0.875194	9.656847
H	0.557222	-1.794806	10.005619
O	0.303770	-1.158763	7.373258
N	2.159383	1.150407	10.202107
C	2.787932	2.060448	11.169745
H	3.801622	2.300457	10.844754
H	2.200177	2.976820	11.260368
H	2.847967	1.588823	12.144996
C	2.537169	2.904944	8.368454
H	3.042593	3.501781	9.128449
H	3.239976	2.778458	7.543287

O	1.585107	1.028314	6.664474
N	-1.861588	1.522373	4.584992
N	1.153487	-0.495639	4.318654
H	1.984185	-0.584882	4.900251
C	-1.903623	-2.528671	5.505827
H	-1.004888	-3.146351	5.584249
H	-2.638616	-3.145485	4.961077
C	-2.475109	-2.280114	6.890341
O	-2.965519	-1.200487	7.227513
C	1.674715	-0.444384	12.033522
H	1.158402	-1.397869	12.153706
H	2.702894	-0.572569	12.386115
H	1.177686	0.285768	12.678889
N	-1.588453	-1.330478	4.702840
H	-2.440371	1.584147	5.423058
In	-0.248324	0.423362	5.831840
O	-1.442328	1.222975	7.277024
H	-1.998606	0.528050	7.642563
C	-1.886963	-4.665338	7.484090
H	-2.028018	-4.949067	6.439916
H	-2.469891	-5.375097	8.077882
N	-2.488794	-3.345107	7.715126
H	-2.937417	-3.172449	8.603265
C	-0.405069	-4.764183	7.745475
C	0.451000	-4.946274	6.653450
C	1.909368	-5.010700	6.936694
C	1.383260	-4.709479	9.333423
C	2.287381	-4.869464	8.302132
H	3.347443	-4.898793	8.541040
C	-0.908531	-4.486637	10.149733
H	-1.227602	-3.440578	10.216210
H	-1.781275	-5.119621	9.992445
H	-0.469033	-4.776720	11.100322
O	0.034654	-5.029257	5.439142
O	2.747097	-5.172961	6.013392
C	1.872793	-4.596832	10.753910
H	1.517980	-3.686623	11.248276
H	1.559514	-5.451170	11.363582
H	2.963610	-4.571677	10.751625
N	0.058337	-4.673936	9.066301

wb97x_L2_III.log

SCF (wB97x) = -2059.39737311
E(SCF)+ZPE(0 K)= -2058.655387
H(298 K)= -2058.611613
G(298 K)= -2058.732859
Lowest Frequency = 10.4051cm-1

O	0.966424	1.380191	7.521516
N	1.041613	2.533027	5.045431
C	-1.329702	2.605773	5.805224
H	-1.067013	2.958224	6.805878
H	-2.311347	3.026143	5.560927
C	-0.311270	3.073434	4.774512
H	-0.622535	2.739245	3.781281
H	-0.285279	4.170099	4.746518
C	1.882081	2.582793	3.822673
H	2.919611	2.423550	4.132394
H	1.823503	3.572377	3.352139
C	1.500911	1.499757	2.817274
H	0.504027	1.661460	2.395976
H	2.209229	1.534259	1.980824
C	1.031885	-0.947928	2.711761
H	1.628023	-1.823462	2.986164
H	1.169001	-0.792581	1.635577
C	-0.445195	-1.193436	3.002043
H	-1.036337	-0.360384	2.610478
H	-0.787429	-2.100905	2.487864
C	-2.113315	-0.952067	4.766007
H	-2.372561	-1.400932	5.729925
H	-2.786382	-1.387049	4.017150
C	-2.321816	0.551845	4.852988
H	-2.149312	1.031882	3.885120
H	-3.359381	0.760734	5.134715
C	1.556003	2.485928	7.475188
C	1.686642	3.241133	6.164318
H	2.758463	3.414480	5.977501
H	1.248855	4.239749	6.292139
N	2.065617	3.017502	8.575233
H	1.948158	2.453210	9.407357
C	4.403356	3.893613	8.672498
C	5.100074	4.032213	7.468327
C	6.569193	3.797472	7.501846
C	6.344953	3.242570	9.901364
C	7.105512	3.402995	8.760581
H	8.172424	3.203582	8.817029
O	7.274528	3.935862	6.472759
N	5.018235	3.499297	9.871133
C	4.198587	3.202084	11.047793
H	3.331636	3.857526	11.087570
H	3.865481	2.157961	11.037212
H	4.768785	3.376988	11.957400
C	2.932245	4.205204	8.661952
H	2.603245	4.781738	9.528874
H	2.755480	4.823662	7.782266
O	4.527352	4.356436	6.363273
N	-1.386032	1.128538	5.838142
N	1.532715	0.195565	3.488951
H	2.489630	-0.002520	3.778587
C	-0.371333	-2.613236	4.992126
H	0.594975	-2.946772	4.594402
H	-1.106356	-3.380797	4.706610
C	-0.213943	-2.499925	6.498638
O	0.041426	-1.380402	7.008070
C	6.984027	2.773889	11.181744
H	8.040229	2.570344	10.998975
H	6.920100	3.529779	11.971687
H	6.521564	1.855294	11.556980
N	-0.703728	-1.287005	4.454295
H	-1.695038	0.845776	6.765876
In	0.718107	0.235001	5.668046
O	2.612412	-0.456908	5.839004
H	2.902403	-0.721316	6.713357
C	-0.684425	-4.939750	6.807579
H	-1.651271	-4.912552	6.300701
H	-0.816000	-5.520705	7.723584
N	-0.335522	-3.575386	7.255370
H	-0.193449	-3.417249	8.244566
C	0.282882	-5.561166	5.841688
C	-0.163772	-5.807545	4.538303
C	0.816996	-6.408097	3.590990
C	2.478721	-6.402739	5.424094
C	2.114543	-6.664541	4.117534
H	2.859011	-7.085510	3.446970
C	1.955899	-5.571231	7.662752
H	2.202822	-4.510243	7.770481
H	1.144042	-5.824156	8.344290
H	2.817911	-6.164208	7.956269
O	-1.345017	-5.508505	4.133835
O	0.504100	-6.654115	2.400627
C	3.879864	-6.695454	5.892363
H	4.366208	-5.807386	6.308093
H	3.901360	-7.481108	6.655025
H	4.473041	-7.039740	5.043799
N	1.575968	-5.882717	6.283135

wb97x_L2_II.log

SCF (wB97x) = -2059.40784336
E(SCF)+ZPE(0 K)= -2058.665000
H(298 K)= -2058.622129
G(298 K)= -2058.736816
Lowest Frequency = 16.9783cm-1

O	0.499656	2.018310	7.685837
N	1.168986	2.548669	5.132251
C	-1.303171	2.865633	5.539942
H	-1.190108	3.259448	6.550576
H	-2.182187	3.346845	5.094007

C	-0.084931	3.200541	4.685221
H	-0.288104	2.876029	3.662037
H	0.048089	4.290625	4.648033
C	2.087370	2.385468	3.984962
H	3.065737	2.096937	4.383580
H	2.218251	3.334526	3.445588
C	1.616518	1.301949	3.030128
H	0.689891	1.578195	2.517494
H	2.377206	1.159300	2.251733
C	0.799039	-1.014038	2.967960
H	1.217743	-1.957759	3.319669
H	1.081995	-0.892745	1.914348
C	-0.718980	-1.029131	3.074827
H	-1.103737	-0.067358	2.727344
H	-1.128691	-1.789803	2.391506
C	-2.509500	-0.660492	4.670292
H	-2.876210	-1.010776	5.637830
H	-3.228459	-0.993626	3.902676
C	-2.469850	0.869133	4.662480
H	-2.217146	1.262234	3.672753
H	-3.476952	1.238571	4.894983
C	1.406213	2.851259	7.568174
C	1.847463	3.303650	6.193725
H	2.936399	3.201213	6.106454
H	1.644840	4.381829	6.104015
N	1.992247	3.403416	8.638501
H	1.648403	3.050196	9.520994
C	4.478153	3.202559	8.579037
C	5.229376	3.169228	7.398411
C	6.365242	2.208593	7.344268
C	5.771135	1.488320	9.631854
C	6.550673	1.393219	8.497170
H	7.352161	0.659137	8.477064
O	7.097596	2.120546	6.327472
N	4.769154	2.393581	9.687862
C	3.943105	2.472791	10.894818
H	3.524861	3.471112	11.002400
H	3.133090	1.735317	10.867499
H	4.552500	2.289118	11.777938
C	3.278650	4.114236	8.635186
H	3.276309	4.782640	9.500265
H	3.334494	4.748978	7.749856
O	4.962005	3.900977	6.374671
N	-1.496932	1.407019	5.629923
N	1.386124	0.067366	3.783035
H	2.285651	-0.269097	4.117701
C	-1.151383	-2.719128	4.707428
H	-0.251548	-3.119275	4.246303
H	-2.018156	-3.195059	4.217905
C	-1.226289	-3.158549	6.168280
O	-2.205550	-2.833918	6.848467
C	6.031379	0.593821	10.815111
H	6.793187	-0.140514	10.546369
H	6.401350	1.155638	11.679669
H	5.131057	0.055391	11.131454
N	-1.182575	-1.258252	4.454830
H	-1.812804	1.164453	6.569620
In	0.447765	0.249131	5.999788
O	-0.653195	-0.379874	7.628248
H	-1.104370	-1.220739	7.537099
C	1.065836	-4.345019	6.356738
H	1.191289	-4.246708	5.276409
H	1.223454	-5.395445	6.603793
N	-0.318777	-4.014824	6.709071
H	-0.579112	-4.262826	7.655516
C	2.050533	-3.416732	7.043278
C	1.968974	-2.104792	6.652904
C	2.721045	-1.073419	7.319529
C	3.736157	-2.864987	8.625183
C	3.630002	-1.516034	8.292237
H	4.263546	-0.798589	8.806554
C	3.024703	-5.217148	8.380439
H	2.054810	-5.561620	8.744679
H	3.320704	-5.801091	7.506499
H	3.758962	-5.371201	9.164138

O	1.157914	-1.739758	5.711176
O	2.478811	0.142145	6.969477
C	4.734838	-3.292577	9.667357
H	4.249512	-3.744083	10.538093
H	5.459607	-4.008111	9.267472
H	5.286990	-2.416104	10.010203
N	2.947378	-3.793097	8.026893

wb97x_L2_IVac.log

SCF (wB97x) = -1563.86583931
E(SCF)+ZPE(0 K)= -1563.301931
H(298 K)= -1563.269327
G(298 K)= -1563.363567
Lowest Frequency = 18.7352cm-1

C	-1.964572	1.783273	3.539862
H	-2.111175	2.311580	4.486940
H	-2.866366	1.937935	2.933340
C	-0.746399	2.377521	2.838700
H	-0.612510	1.964421	1.833540
H	-0.893330	3.458387	2.723636
C	1.736852	2.447944	3.044729
H	2.407563	2.717688	3.866272
H	1.642273	3.328532	2.398480
C	2.319442	1.292713	2.237773
H	1.676925	1.088533	1.375913
H	3.306428	1.567247	1.842914
C	2.376805	-1.151440	2.188666
H	2.822287	-1.977447	2.750051
H	2.988074	-0.998287	1.290240
C	0.956211	-1.533781	1.798115
H	0.498799	-0.767376	1.164725
H	0.977396	-2.460394	1.213810
C	-1.312097	-1.839780	2.736001
H	-1.754667	-2.429755	3.542391
H	-1.479197	-2.390262	1.803159
C	-1.972947	-0.471225	2.633547
H	-1.549809	0.068059	1.781351
H	-3.045640	-0.594109	2.433131
N	-1.771016	0.348747	3.848892
N	2.399728	0.057243	3.043356
C	-2.634791	-0.109396	4.950524
H	-3.600891	-0.474505	4.582326
H	-2.839627	0.734099	5.617661
O	-2.731471	-1.865994	6.548468
C	-1.994601	-1.191996	5.832493
O	-0.716736	-1.293017	5.790095
N	0.132039	-1.697272	3.010356
H	0.448895	-2.535518	3.492916
C	3.568673	0.045391	3.926601
N	4.247498	-1.055609	6.017239
H	4.479838	-0.258113	3.391762
H	3.737405	1.049112	4.331808
C	5.449978	-0.232450	6.157451
C	5.134512	1.192789	6.565923
H	6.010410	-0.290431	5.223752
H	6.059969	-0.734836	6.915702
C	4.182913	1.395488	7.565628
N	5.816424	2.239924	5.929199
C	3.846498	2.803500	7.903571
O	3.584328	0.422786	8.163153
C	5.497054	3.525033	6.206258
C	6.904175	1.920703	5.000658
C	4.531781	3.802415	7.152665
O	3.008182	3.081635	8.795945
H	3.897842	-1.433179	6.889843
C	6.199014	4.650030	5.491261
H	7.581004	1.191768	5.450496
H	6.518223	1.527102	4.054697
H	7.482233	2.814235	4.784680
H	4.291020	4.842471	7.356281
H	5.738952	5.595072	5.784174
H	7.261369	4.703541	5.752206
H	6.120562	4.558771	4.403528

N	0.443484	2.078936	3.640442
H	0.374434	2.554454	4.538631
C	3.305794	-0.878583	5.108145
O	2.188415	-1.434650	5.219606
In	0.516563	-0.063301	4.579053
O	1.191360	1.167934	6.034409
H	1.710338	0.824280	6.770374

wb97x_L2_IVhc.log

SCF (wB97x) = -1563.88110360
E(SCF)+ZPE(0 K)= -1563.317279
H(298 K)= -1563.284768
G(298 K)= -1563.376959
Lowest Frequency = 21.0384cm-1

C	-1.889444	2.477452	3.907975
H	-2.315012	2.671637	4.899676
H	-2.525126	2.994006	3.173645
C	-0.475609	3.039379	3.875349
H	-0.017588	2.910777	2.888064
H	-0.510452	4.118515	4.072792
C	1.774767	2.697539	4.842587
H	2.146681	2.670539	5.865930
H	1.910587	3.717324	4.459731
C	2.580565	1.739857	3.965998
H	2.251516	1.856673	2.929090
H	3.639758	2.013411	4.007511
C	2.528669	-0.545996	3.135479
H	2.624598	-1.577948	3.488745
H	3.449600	-0.294920	2.589899
C	1.332912	-0.477600	2.197031
H	1.209686	0.518897	1.760479
H	1.505226	-1.169483	1.362119
C	-1.124024	-0.728460	2.151888
H	-1.823522	-1.477392	2.536201
H	-0.949799	-0.964218	1.094436
C	-1.721373	0.668207	2.277783
H	-1.042244	1.394361	1.820699
H	-2.668899	0.726170	1.722503
N	-1.903046	1.023146	3.689864
N	2.384327	0.317428	4.331928
C	-3.103247	0.424294	4.270183
H	-3.310504	-0.534958	3.783543
H	-3.990645	1.054584	4.124188
O	-1.740032	-0.015164	6.192022
C	-2.936976	0.117581	5.763674
O	-3.951469	-0.022070	6.450606
N	0.114511	-0.831140	2.932889
H	0.215502	-1.781302	3.288141
O	4.382709	1.705908	6.268107
C	3.790202	0.652112	6.497127
N	3.781468	0.151377	7.765470
H	4.261133	0.782228	8.396001
C	2.770213	-0.683997	8.422294
H	2.328587	-1.353839	7.682530
H	3.279107	-1.301133	9.162753
C	1.683159	0.189812	9.013144
C	0.922147	0.878409	8.094343
C	0.037510	1.953200	8.535024
C	0.660631	1.313110	10.838337
C	-0.073046	2.075422	9.951285
H	-0.746983	2.827849	10.350697
O	1.111853	0.646749	6.824760
O	-0.540654	2.708434	7.708256
C	0.486546	1.509928	12.320978
H	-0.251485	2.295144	12.489937
H	0.126632	0.600498	12.812561
H	1.419252	1.814317	12.805735
C	2.358101	-0.421598	11.293823
H	2.199217	-1.482934	11.092074
H	3.418891	-0.181621	11.184441
H	2.069019	-0.227503	12.321857
N	1.549091	0.391722	10.379861
C	3.320332	-0.235613	5.349533

H	2.862861	-1.155057	5.718742
H	4.264247	-0.504650	4.849780
N	0.341054	2.348310	4.877465
H	-0.007022	2.583594	5.819754
In	0.090030	0.088237	5.101802
O	0.421707	-1.929001	5.356669
H	0.307219	-2.260754	6.248331

wb97x_L2.log

SCF (wB97x) = -1982.75267091
E(SCF)+ZPE(0 K)= -1982.005832
H(298 K)= -1981.963836
G(298 K)= -1982.080275
Lowest Frequency = 14.2120cm-1

O	3.164604	3.076380	6.583509
O	2.056988	-0.862617	5.491803
N	0.649023	2.689757	5.118971
N	1.180146	-1.071647	2.869993
C	-1.401432	1.261364	5.223031
H	-1.319698	1.202208	6.316449
H	-2.475636	1.290759	4.995128
C	-0.762072	2.566330	4.735436
H	-0.824272	2.603632	3.644252
H	-1.348858	3.427629	5.104215
C	1.421013	3.441459	4.121681
H	2.351256	3.765876	4.590509
H	0.866857	4.341814	3.797542
C	1.769257	2.591917	2.902675
H	0.842563	2.259043	2.402556
H	2.295683	3.229918	2.179783
C	2.935478	0.548812	2.200583
H	3.766176	-0.089025	2.530703
H	3.304648	1.104226	1.328059
C	1.764105	-0.341450	1.757248
H	0.976676	0.283501	1.322261
H	2.110805	-1.016746	0.952084
C	-0.245881	-1.353318	2.761636
H	-0.475258	-2.228964	3.380098
H	-0.530012	-1.603793	1.725600
C	-1.094263	-0.186834	3.259363
H	-0.885068	0.697232	2.630998
H	-2.156942	-0.427584	3.118241
C	2.088065	2.939913	7.157501
C	2.183973	-1.957427	4.945381
C	1.982362	-2.127668	3.432277
H	1.555484	-3.119654	3.223471
H	2.983884	-2.133031	2.980653
C	0.758718	3.236392	6.465761
H	0.631619	4.336111	6.480436
H	-0.061384	2.827662	7.057969
N	2.070451	2.594651	8.471192
H	2.986581	2.353240	8.822997
N	2.519689	-3.054605	5.656995
H	2.565819	-2.914979	6.668388
C	2.771942	-4.385253	5.112906
H	3.471523	-4.323379	4.276224
H	1.852957	-4.847723	4.731898
C	3.414341	-5.262415	6.154512
C	4.742535	-5.553952	6.070076
C	5.423367	-6.381844	7.046320
C	3.258155	-6.533449	8.169941
C	4.596638	-6.847975	8.102920
H	5.034349	-7.464994	8.880812
C	0.552546	0.709495	9.009194
C	1.508969	-0.257142	8.919148
C	1.200724	-1.620771	8.557909
C	-1.128761	-0.892581	8.446566
C	-0.169978	-1.870422	8.296824
H	-0.478324	-2.860383	7.977498
O	5.514628	-5.081832	5.062358
H	6.399755	-5.445192	5.242310
O	2.146944	-2.452157	8.485890
O	6.648032	-6.608848	6.891855

N	2.668540	-5.763434	7.211726
N	-0.780617	0.369296	8.831462
C	1.241885	-5.437518	7.351157
H	1.101427	-4.767705	8.202994
H	0.882042	-4.932884	6.459468
H	0.662935	-6.352017	7.483767
C	-1.812678	1.367817	9.150769
H	-1.574750	1.854283	10.096994
H	-1.901536	2.118328	8.359347
H	-2.773896	0.877238	9.266662
C	0.950406	2.151139	9.284158
H	0.114175	2.835483	9.152694
H	1.259885	2.248865	10.329407
O	2.815509	0.015244	9.144525
H	3.273854	-0.831180	8.997906
N	-0.835033	0.039902	4.673432
H	0.172016	0.028776	4.827696
N	2.640349	1.486170	3.274606
H	2.213906	0.969780	4.041463
C	-2.573978	-1.208434	8.177527
H	-2.643878	-2.210300	7.752507
H	-3.172020	-1.191781	9.094148
H	-3.014553	-0.503985	7.465060
C	2.411544	-7.036714	9.304299
H	1.631616	-7.718200	8.950220
H	3.040590	-7.578387	10.011327
H	1.922602	-6.215341	9.836474

wb97x_L2_TS(Ib).log

SCF (wB97x) = -2059.36978375
E(SCF)+ZPE(0 K)= -2058.626173
H(298 K)= -2058.584828
G(298 K)= -2058.693762
Lowest Frequency = -203.6906cm-1

O	0.232970	4.279568	6.844010
N	1.021757	1.935945	4.587564
C	-1.322644	2.780700	4.473596
H	-1.197402	3.415042	5.353558
H	-2.041915	3.257855	3.797761
C	0.017513	2.640864	3.761709
H	-0.119021	2.077809	2.832556
H	0.385035	3.637049	3.480868
C	2.045386	1.286325	3.732616
H	2.900019	1.045855	4.370143
H	2.394423	1.971360	2.948392
C	1.544923	-0.007169	3.105511
H	0.732877	0.175890	2.394867
H	2.362662	-0.468025	2.539536
C	0.346841	-2.107064	3.598711
H	0.501816	-2.955796	4.273327
H	0.771312	-2.386583	2.626031
C	-1.131843	-1.804759	3.423838
H	-1.260680	-1.006509	2.687496
H	-1.641472	-2.688619	3.014816
C	-3.007361	-0.619257	4.369622
H	-3.611251	-0.570604	5.276871
H	-3.591243	-1.140804	3.596899
C	-2.681986	0.791834	3.904845
H	-2.154421	0.803745	2.946148
H	-3.615272	1.348111	3.759731
C	0.971722	3.235895	6.867531
C	1.705548	2.829873	5.556960
H	2.635055	2.332771	5.822107
H	1.957683	3.777576	5.061743
N	1.898610	3.228788	7.969693
H	1.477343	3.725525	8.744086
C	2.098351	0.934363	8.936874
C	1.541139	0.078261	8.012951
C	0.353788	-0.636725	8.397334
C	0.805361	-0.026068	10.697667
C	0.066864	-0.736766	9.764749
H	-0.783586	-1.322421	10.099442
O	-0.428318	-1.028292	7.443673
N	1.772447	0.828540	10.277761
C	2.499302	1.678576	11.231735
H	3.571087	1.607927	11.044067
H	2.175354	2.718325	11.138650
H	2.311905	1.345565	12.247731
C	2.806339	2.156076	8.387750
H	3.500614	2.596002	9.105933
H	3.407286	1.833200	7.536343
O	1.826474	0.117671	6.732027
N	-1.834716	1.458735	4.909557
N	1.053045	-0.932629	4.146314
H	1.852640	-1.274689	4.682153
C	-2.096792	-2.612237	5.451681
H	-1.205691	-3.240958	5.467315
H	-2.864069	-3.185478	4.900766
C	-2.651778	-2.448672	6.860888
O	-3.368756	-1.505604	7.196986
C	0.510107	-0.174120	12.165310
H	-0.305765	-0.887489	12.291892
H	1.376432	-0.556650	12.715501
H	0.206447	0.772459	12.622031
N	-1.781140	-1.384129	4.691058
H	-2.416453	1.616756	5.728981
In	-0.083992	0.211198	5.748713
O	-0.108219	1.946220	7.088783
H	-0.847877	2.389771	7.512287
C	-1.555848	-4.654122	7.535122
H	-1.716024	-5.093787	6.548980
H	-1.901303	-5.390197	8.266334
N	-2.434725	-3.484624	7.703247
H	-2.917593	-3.383059	8.584272
C	-0.075396	-4.377778	7.641250
C	0.694808	-4.406698	6.476107
C	2.073047	-3.863417	6.570695
C	1.773388	-3.743358	9.017430
C	2.555624	-3.620152	7.886372
H	3.586602	-3.294847	8.000206
C	-0.371521	-4.266907	10.077854
H	-1.303500	-3.710433	9.973421
H	-0.598577	-5.327174	10.226057
H	0.133123	-3.893584	10.964441
O	0.233304	-4.783703	5.333370
O	2.755354	-3.631969	5.537061
C	2.375501	-3.506664	10.377453
H	1.894597	-2.671428	10.898248
H	2.305879	-4.392478	11.017197
H	3.432362	-3.261787	10.258299
N	0.469951	-4.079503	8.895894

wb97x_L2_TS(III).log

SCF (wB97x) = -2059.36414455
E(SCF)+ZPE(0 K)= -2058.621799
H(298 K)= -2058.579616
G(298 K)= -2058.694908
Lowest Frequency = -258.9783cm-1

O	-0.182534	1.821188	7.885457
N	0.474446	3.107948	5.470527
C	-1.996961	3.093798	5.637943
H	-1.987101	3.416408	6.681767
H	-2.911140	3.479911	5.174243
C	-0.776929	3.651791	4.909695
H	-0.817997	3.392609	3.847076
H	-0.788811	4.747802	4.969419
C	1.599937	3.174162	4.511020
H	2.524656	3.104357	5.089275
H	1.602786	4.134000	3.978987
C	1.555147	2.028568	3.505801
H	0.685722	2.103188	2.845230
H	2.443355	2.076486	2.866411
C	1.192635	-0.413076	3.306011
H	1.644717	-1.322884	3.719911
H	1.630117	-0.249614	2.314598
C	-0.315090	-0.587688	3.175140

H	-0.762132	0.298588	2.715371
H	-0.536323	-1.434707	2.513521
C	-2.385994	-0.505089	4.484900
H	-2.824286	-0.942488	5.387730
H	-2.865859	-0.980962	3.619905
C	-2.668386	0.990811	4.482230
H	-2.304152	1.469511	3.568832
H	-3.750899	1.153960	4.518632
C	0.889385	2.591301	7.840585
C	0.830850	3.703599	6.783866
H	1.772727	4.258432	6.714654
H	0.045467	4.396855	7.104126
N	1.402909	2.962530	9.063828
H	1.252859	2.265706	9.779175
C	3.836426	3.399625	8.556284
C	4.301596	4.125649	7.454442
C	5.470005	3.570865	6.720609
C	5.489128	1.686428	8.318594
C	5.984627	2.334303	7.205913
H	6.816967	1.883723	6.671046
O	5.950127	4.153146	5.713563
N	4.450839	2.220999	9.000318
C	3.986989	1.560540	10.223971
H	3.316999	0.722268	10.002911
H	4.838092	1.183194	10.789978
H	3.467931	2.272589	10.860236
C	2.560654	3.850078	9.222855
H	2.670386	4.017345	10.298735
H	2.327525	4.823257	8.787415
O	3.737023	5.205851	7.035066
N	-2.005501	1.611074	5.643595
N	1.497270	0.723103	4.202968
H	2.407075	0.568009	4.631697
C	-0.651375	-2.149148	5.001416
H	-0.018628	-2.707800	4.299469
H	-1.579602	-2.712648	5.142167
C	0.110563	-2.126830	6.310970
O	0.360287	-1.033284	6.889625
C	6.102537	0.397807	8.796661
H	6.824416	0.048893	8.056408
H	6.633763	0.526275	9.746283
H	5.351871	-0.386204	8.937985
N	-0.933792	-0.792305	4.504799
H	-2.510159	1.323088	6.479179
In	0.117913	0.861418	6.007091
O	1.967079	1.547858	6.988993
H	2.423625	1.005217	7.639003
C	0.508320	-4.597155	6.251614
H	-0.096288	-4.552461	5.344965
H	-0.000404	-5.268252	6.943248
N	0.515782	-3.254561	6.857406
H	1.033970	-3.142554	7.720410
C	1.895058	-5.046700	5.879363
C	2.469193	-4.444460	4.759903
C	3.824896	-4.899199	4.357804
C	3.783388	-6.439232	6.297621
C	4.405040	-5.901425	5.186698
H	5.397293	-6.260357	4.926625
C	1.861383	-6.574689	7.816852
H	1.525981	-5.774512	8.480161
H	1.001697	-7.177184	7.509814
H	2.538547	-7.207624	8.381870
O	1.861028	-3.530506	4.090065
O	4.403307	-4.421436	3.351231
C	4.482058	-7.499961	7.108234
H	4.669804	-7.174461	8.136622
H	3.908988	-8.431753	7.145652
H	5.446488	-7.718319	6.647213
N	2.548923	-6.016955	6.650452

wb97x_L2_TS(II).log

SCF (wB97x) = -2059.36734317
E(SCF)+ZPE(0 K)= -2058.624417
H(298 K)= -2058.582620
G(298 K)= -2058.695510
Lowest Frequency = -310.1241cm-1

N	0.052350	1.573845	4.854646
C	-2.096878	1.503624	3.662959
H	-2.626128	1.977744	4.495021
H	-2.610166	1.799026	2.737981
C	-0.651311	2.007610	3.637202
H	-0.112542	1.624128	2.763893
H	-0.655314	3.101083	3.556257
C	1.512837	1.795638	4.875158
H	1.797988	1.968905	5.913141
H	1.766956	2.697548	4.304052
C	2.308404	0.623032	4.303110
H	2.044221	0.508624	3.246881
H	3.376005	0.869343	4.343233
C	2.234111	-1.781339	4.011141
H	2.327357	-2.708730	4.586516
H	3.171049	-1.657044	3.448386
C	1.069419	-1.934019	3.055696
H	0.986778	-1.082678	2.373521
H	1.229847	-2.823154	2.433950
C	-1.368531	-2.099193	2.942188
H	-2.146229	-2.659209	3.464062
H	-1.134851	-2.629208	2.011350
C	-1.849129	-0.691880	2.624523
H	-1.068949	-0.148004	2.083079
H	-2.725942	-0.733832	1.964359
N	-2.158420	0.041026	3.862670
C	-3.420835	-0.420840	4.482294
H	-3.945406	-1.104300	3.802352
H	-4.082563	0.427576	4.683866
C	-3.132751	-1.192649	5.774171
O	-2.094426	-1.966481	5.800860
N	-0.180111	-2.049001	3.820397
H	-0.154210	-2.923804	4.339567
In	-0.530589	-0.454949	5.542713
O	-2.257980	0.185732	6.662028
H	-2.520397	1.105116	6.772482
C	-5.331690	-0.674878	6.815860
H	-4.946731	0.329699	7.016375
H	-5.775409	-1.045794	7.744912
N	-4.212933	-1.583251	6.514952
H	-3.913727	-2.144459	7.301217
C	-6.378471	-0.528377	5.742056
C	-6.489896	0.693794	5.070375
C	-7.567921	0.813606	4.053086
C	-8.245672	-1.498996	4.605455
C	-8.401937	-0.328186	3.889552
H	-9.204378	-0.269684	3.158565
C	-7.110169	-2.806940	6.334080
H	-7.538464	-2.647399	7.329363
H	-7.622090	-3.642609	5.864490
H	-6.056989	-3.068457	6.427260
O	-5.689802	1.682073	5.273443
O	-7.723703	1.868878	3.386259
C	-9.190552	-2.651708	4.387127
H	-8.675651	-3.538841	4.003431
H	-9.710521	-2.935394	5.307751
H	-9.942579	-2.359226	3.652505
N	-7.249906	-1.602336	5.511622
N	2.040739	-0.673345	4.969036
O	3.848659	1.108229	6.778936
C	3.287859	0.072496	7.128755
N	3.220169	-0.246017	8.455670
H	3.644997	0.486576	9.012029
C	2.148101	-0.960150	9.160284
H	1.766540	-1.762231	8.528856
H	2.582506	-1.417007	10.048678
C	1.036886	0.022359	9.466635
C	0.361722	0.508484	8.367435
C	-0.384709	1.757834	8.486473
C	0.040994	1.614557	10.916541
C	-0.559938	2.208234	9.828780
H	-1.166804	3.094223	9.989881

O	0.595874	-0.072311	7.205860	H	2.628200	0.099597	11.817096
O	-0.801758	2.403173	7.485747	H	1.174419	0.301799	12.802190
C	-0.189667	2.157690	12.300832	N	0.861560	0.544237	10.737039
H	-0.847131	3.025587	12.235936	C	2.923791	-1.000922	6.115299
H	-0.671164	1.420181	12.950828	H	2.490030	-1.864900	6.619478
H	0.743446	2.475119	12.776011	H	3.908471	-1.310994	5.726765
C	1.552998	-0.092392	11.864346	H	-0.328993	2.069908	5.674689
H	1.369852	-1.168076	11.854336				

References

[1] SHELXTL v5.1, Bruker AXS, Madison, WI, 1998.

[2] SHELX-2013, G.M. Sheldrick, *Acta Cryst.*, 2015, **C71**, 3-8.

[3] O.V. Dolomanov, L.J. Bourhis, R.J. Gildea, J.A.K. Howard, H. Puschmann, *J. Appl. Cryst.*, 2009, **42**, 339-341.

[4] Frisch, M. J.; Trucks, G. W.; Schlegel, H. B.; Scuseria, G. E.; Robb, M. A.; Cheeseman, J. R.; Scalmani, G.; Barone, V.; Mennucci, G.; Petersson, G. A.; Nakatsuji, H.; Caricato, M.; Li, X.; Hratchian, H. P.; Izmaylov, A. F.; Bloino, J.; Zheng, G.; Sonnenberg, J. L.; Hada, M.; Ehara, M.; Toyota, K.; Fukuda, R.; Hasegawa, J.; Ishida, M.; Nakajima, T.; Honda, Y.; Kitao, O.; Nakai, H.; Vreven, T.; Montgomery, J. A., Jr.; Peralta, J. E.; Ogliaro, F.; Bearpark, M.; Heyd, J. J.; Brothers, E.; Kudin, K. N.; Staroverov, V. N.; Kobayashi, R.; Normand, J.; Raghavachari, K.; Rendell, A.; Burant, J. C.; Iyengar, S. S.; Romasi, J.; Cossi, M.; Rega, N.; Millam, J. M.; Klene, M.; Knox, J. E.; Cross, J. B.; Bakken, V.; Adamo, C.; Jaramillo, J.; Gomperts, R.; Stratmann, R. E.; Yazyev, O.; Austin, A. J.; Cammi, R.; Pomelli, C.; Ochterski, J. W.; Martin, R. L.; Morokuma, K.; Zakrzewski, V. G.; Voth, G. A.; Salvador, P.; Dannenberg, J. J.; Dapprich, S.; Daniels, A. D.; Farkas, Ö.; Foresman, J. B.; Ortiz, J. V.; Cioslowski, J.; Fox, D. J. *Gaussian 09*, *Revision D.01*; Gaussian, Inc., Wallingford, CT, USA (2009).

[5] Gaussian 16, Revision C.01, Frisch, M. J.; Trucks, G. W.; Schlegel, H. B.; Scuseria, G. E.; Robb, M. A.; Cheeseman, J. R.; Scalmani, G.; Barone, V.; Petersson, G. A.; Nakatsuji, H.; Li, X.; Caricato, M.; Marenich, A. V.; Bloino, J.; Janesko, B. G.; Gomperts, R.; Mennucci, B.; Hratchian, H. P.; Ortiz, J. V.; Izmaylov, A. F.; Sonnenberg, J. L.; Williams-Young, D.; Ding, F.; Lipparini, F.; Egidi, F.; Goings, J.; Peng, B.; Petrone, A.; Henderson, T.; Ranasinghe, D.; Zakrzewski, V. G.; Gao, J.; Rega, N.; Zheng, G.; Liang, W.; Hada, M.; Ehara, M.; Toyota, K.; Fukuda, R.; Hasegawa, J.; Ishida, M.; Nakajima, T.; Honda, Y.; Kitao, O.; Nakai, H.; Vreven, T.; Throssell, K.; Montgomery, J. A., Jr.; Peralta, J. E.; Ogliaro, F.; Bearpark, M. J.; Heyd, J. J.; Brothers, E. N.; Kudin, K. N.; Staroverov, V. N.; Keith, T. A.; Kobayashi, R.; Normand, J.; Raghavachari, K.; Rendell, A. P.; Burant, J. C.;

Iyengar, S. S.; Tomasi, J.; Cossi, M.; Millam, J. M.; Klene, M.; Adamo, C.; Cammi, R.; Ochterski, J. W.; Martin, R. L.; Morokuma, K.; Farkas, O.; Foresman, J. B.; Fox, D. J. Gaussian, Inc., Wallingford CT, 2016.

[6] Chai, J.-D.; Head-Gordon, M. Systematic Optimization of Long-Range Corrected Hybrid Density Functionals. *J. Chem. Phys.* **2008**, *128*, 084106.

[7] Alipour, M.; Fallahzadeh, P. First Principles Optimally Tuned Range-Separated Density Functional Theory for Prediction of Phosphour–Hydrogen Spin–Spin Coupling Constants. *Phys. Chem. Che. Phys.* **2016**, *18*, 18431–18440.

8 Perdew, J. P.; Burke, K.; Ernzerhof, M. Generalized Gradient Approximation Made Simple, *Phys. Rev. Lett.* **1996**, *77*, 3865–3868.

9 Adamo, C.; Barone, V. Toward Reliable Density Functional Methods Without Adjustable Parameters: The PBE0 Model. *J. Chem. Phys.* **1999**, *110*, 6158–6170.

10 Grimme, S.; Ehrlich, S.; Goerigk, L. Effect of the Damping Function in Dispersion Corrected Density Functional Theory. *J. Comp. Chem.* **2011**, *32*, 1456–1465.

11 Becke, A. D.; Johnson, E. R. A Density-Functional Model of the Dispersion Interaction. *J. Chem. Phys.* **2005**, *123*, 154101.

12 *NBO 6.0.* Glendening, E. D.; Badenhoop, J. K.; Reed, A. E.; Carpenter, J. E.; Bohmann, J. A.; Morales, C. M.; Landis, C. R.; Weinhold, F. Theoretical Chemistry Institute, University of Misconsin, Madison (2013).

13 Barone, V.; Cossi, M. Quantum Calculation of Molecular Energies and Energy Gradients in Solution by a Conductor Solvent Model. *J. Phys. Chem. A* **1998**, *102*, 1995–2001.

14 Weigend, F.; Ahlrichs, R. Balanced basis sets of split valence, triple zeta valence and quadruple zeta valence quality for H to Rn: Design and assessment of accuracy. *Phys. Chem. Chem. Phys.* **2005**, *7*, 3297-3305.
